# Investigating microbial population structure and function in the chicken caeca and large intestine over time using metagenomics

**DOI:** 10.1186/s13104-025-07441-7

**Published:** 2025-08-15

**Authors:** Banaz Star-Shirko, Gladys Maria Pangga, Aaron McKenna, Nicolae Corcionivoschi, Anne Richmond, Umer Zeeshan Ijaz, Ozan Gundogdu

**Affiliations:** 1 Faculty of Infectious and Tropical Diseases, London School of Hygiene and Tropical Medicine, London, UK; 2 James Watt School of Engineering, University of Glasgow, Glasgow, UK; 3 Food Microbiology, Agri-Food and Biosciences Institute, Newforge Lane, Belfast, UK; 4 Pilgrim’s Europe Ltd, Craigavon, UK

**Keywords:** Chicken gut microbiome, Metagenomics, Gastroenteritis, Functional profile, Shotgun sequencing

## Abstract

**Objectives:**

Although taxonomic variations in chicken gut microbiota have been previously documented, their functional capacity remain poorly understood. To gain a better understanding, we incorporated whole genome shotgun metagenomics to analyse microbial communities of two different organs: the caeca and the large intestine.

**Results:**

Using 24 samples obtained from the caeca and the large intestine of commercial chickens, we assembled Metagenome-Assembled Genomes (MAGs) and characterise their functional profiles. Afterwards, using 8 samples, we integrated this sequencing data with chicken performance metadata body weight (BW), weight gain, feed intake (FI), feed conversion ratio (FCR) and age. MAGs belonging to specific families were found to be positively associated with changes in performance parameters. Functional analyses suggest changes in nutrient geochemical cycles including hydrogen generation within the carbon-cycle. Furthermore, 108 CAZymes were identified for MAGs belonging to two major families – glycoside hydrolase (GH) and polysaccharide lyase (PL), which are important for breakdown of dietary carbohydrates and fibres. A total of 13 polysaccharide lyases were identified functioning on day 20 with enzymes were specific to organs. Overall, our results provide a deeper understanding of microbial-mediated metabolism concerning key performance parameters in chicken production.

**Supplementary Information:**

The online version contains supplementary material available at 10.1186/s13104-025-07441-7.

## Introduction

The chicken gut microbiome is populated with a complex community of microorganisms such as bacteria, archaea and viruses. These microbes play an important role in chicken productivity [1, 2]. The chicken microbiota has a vital role in digestion and absorption of nutrients, immune system development, vitamin and amino acid production and inhibition of pathogen colonisation [3, 4]

We have previously performed a comprehensive day-to-day microbiome analysis of the chicken caeca from day 3 to 35, highlighting changes in population structure [5]. However, this previous study was only performed for a single organ (caeca) and utilised 16S rRNA amplicon sequencing (V3–V4).

To obtain a deeper understanding of the changes of the chicken gut microbiome over time and its impact on the presence of pathogenic bacteria, we utilised resources from our previous study and incorporated shotgun metagenomics for two different organs: the caeca and the large intestine. This approach allows for a more in-depth analysis through shotgun metagenomic sequencing, enabling the recovery of nearly complete Metagenome-Assembled Genomes (MAGs) and their taxonomic annotation and functional profiling.

## Materials and methods

### Genomic DNA, experimental design and shotgun metagenomic sequencing

For completion purposes, it is import to discuss the previous study [5], from which the design of this study arose. The study included caecum microbiome samples of 396 chicken (Ross-308) provided by Moy Park (39 Seagoe Industrial Estate, Portadown, Craigavon, Co. Armagh, BT63 5QE, UK). The birds were allocated on 12 pens (33 broiler chicks/pen). The birds under 250 g were euthanised by dislocation of the neck whereas those over 250 g and up to 1 kg were euthanised by dislocation of the neck following anaesthesia using isoflurane. Birds over 1 kg were euthanised by an overdose of anaesthetic (isoflurane) followed by dislocation of the neck. Anaesthesia was carried out using an anaesthetic mask fitted over the bird's head to deliver the vapourised isoflurane with oxygen with death confirmed in all birds by the onset of rigor mortis. Following this, genomic DNA (gDNA) was extracted from the digesta of the caeca and large intestine of broiler chicken, and analysed in the current study. For shotgun metagenomics, extractions of gDNA from broiler chickens were performed using the QIAamp DNA Stool Mini Kit according to the manufacturer’s instructions and stored in -20 °C freezers. The performance parameters were obtained previously^(5)^ and included bird age, body weight (BW), body weight gain, feed intake (FI) and feed conversion ratio (FCR).

**Fig. 1:**
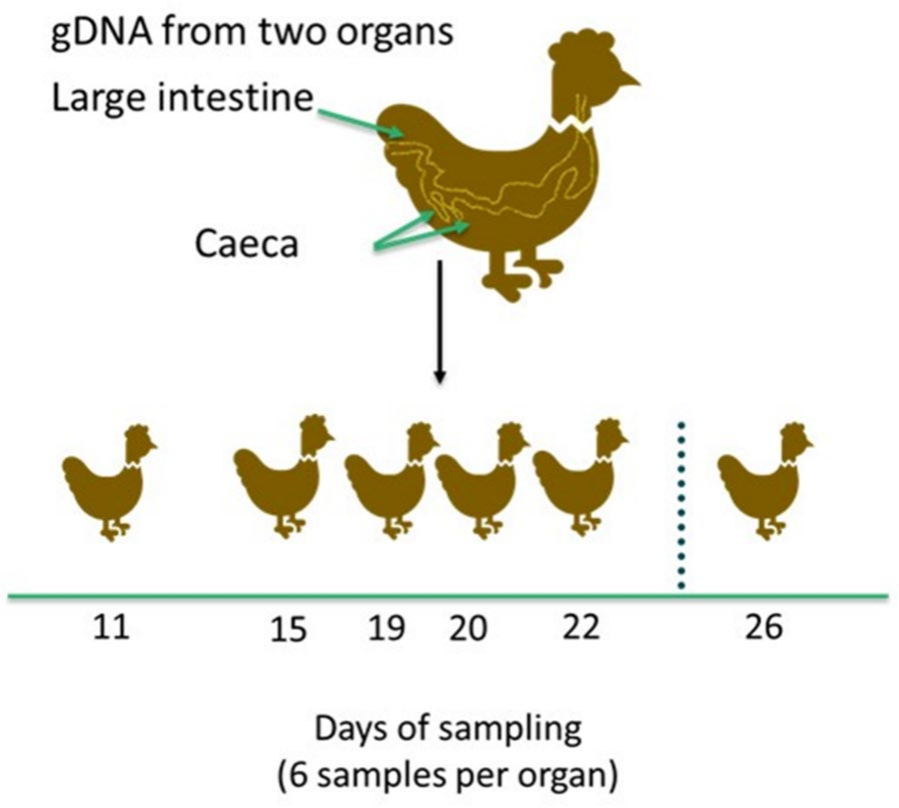
Overview of study design showing age of sampling gDNA from two organs caeca and large intestine followed by shotgun metagenomics sequencing and bioinformatics and statistical analysis

Of the days reported previously [5], days 12 to 20 were deemed important, and therefore, including day 22, and 26, the metagenomic profiles were obtained for the days 11, 15, 19, 20, 22 and 26, whilst also investigating multiple organs (Fig. 1). Quantification of gDNAs was performed using Qubit 3 and short-read shotgun metagenomics sequencing library was constructed using a modified Illumina DNA Prep tagmentation approach (Illumina, Inc., Cambridge, UK) described previously [6]. After library qualification, the library was sequenced using the sequencer (NextSeq 2000).

### Bioinformatic analysis

#### Recovery of metagenomic-assembled genomes (MAGs)

A total of 744,270,746 reads were produced from metagenomic sequencing of 24 samples. Reads were subjected to quality trimming using Sickle v1.200 [7]. Trimming involved removing reads where the average phred below 20 and retaining paired end reads with a post-trimming length exceeding 50 bp. Sixteen samples were excluded due to host contamination and lower DNA yield, resulting in a total of 8 samples which generated 379,415,912 reads. To pre-screen for contaminants, we have used Phyloflash [8] that rapidly screens metagenomics datasets for prokaryotic and eukaryotic species after assembling small-subset rRNA reads. We removed those samples where we did not get enough prokaryotic coverage and the reads were predominantly selected as 18S rRNA reads with details provided in the Supplementary Data 1. We have initially sequenced four organs (as can be seen from the table: Small Intestine, Large Intestine, Caeca, and Duodenum. Majority of the contaminants were in Duodenum and Small Intestine, and therefore, in the publication, we have only proceeded with Caeca and Large Intestine comparison. Forward and reverse reads were then aggregated from all samples to obtain a single assembly (comprising of continguous regions) using MEGAHIT [9]. Assembly parameters used were –k-list 27,47,67,87 –kmin-1pass -m 0.95 –min-contig-len 1000. This gave us a total of 395,830 contigs, with a total of 1,464,921,437 bases (bp), with the maximum contig size of 214,917 bp, average length of 3,701 bp, and an N50 score of 5,371 bp. The contigs were then subjected to binning (clustering at genome level) via the MetaWRAP pipeline [10], wherein three algorithms were utilised, i.e., metabat2 [11] (260 MAGs recovered), MaxBin [12] (220 MAGs recovered), and CONCOCT (221 MAGs, resulting in a total of 154 MAGs. We retained > 50% completed MAGs with < 10% contamination in MetaWRAP which internally uses CheckM to calculate the completion and contamination statistics [13]. For downstream statistical analyses, we have used high quality MAGs, retaining those with > 75% completeness and < 5% contamination, resulting in a total of 54 MAGs. This strategy was previously used [14] and offered reasonable results without biases associated with incompleteness or contamination. The summary statistics of the MAGs are provided in the Supplementary Table S1.

#### Taxonomic and functional annotation

For metabolic function and taxonomic assessment of each MAG, the METABOLIC pipeline was employed [15] Within its framework: taxonomic classification of MAGs was done using GTDB-TK [16]; functional annotations were recovered using Kyoto Encyclopedia of Genes and Genomes (KEGG) at coarser (modules) and finer (submodule) levels [17]; carbohydrate active enzymes (CAZymes) were recovered from dbCAN2 [18]; custom functions were recovered using customised hidden Markov model databases for nutrient cycles [19]; and proteases were recovered using MEROPS [20]. To obtain taxonomic coverages per sample, read coverages (mean number of reads aligned to MAGs on sample-wise basis) were calculated using CoverM (https://github.com/wwood/CoverM). The coverage table was then multiplied with feature tables recovered from METABOLIC to give coverage of functional tables on sample-wise basis.

#### Phylogenetic tree generation

To construct a phylogenetic tree of MAGs, we used GToTree [21] that first recovers Single Copy Genes (SCGs) from MAGs and then aligns them together to generate a phylogenetic tree. In GToTree, we have used a pre-calculated 25 SCGs set covering the bacterial and archaeal domain. Note that for MAGs that had very few hits for these SCG were removed, resulting in a phylogeny recovery of a total of 32 MAGs. For assessment of novelty of MAGs, the Genome Tree Toolkit was utilised [13], wherein phylogenetic gain (PG) for each MAG against all other MAGs was calculated and used as a proxy for novelty.

#### Statistical analyses

Statistical analyses are provided in Supplementary Information.pdf.

## Results

### Performance parameters and their association with key microbes and functions

**Fig. 2:**
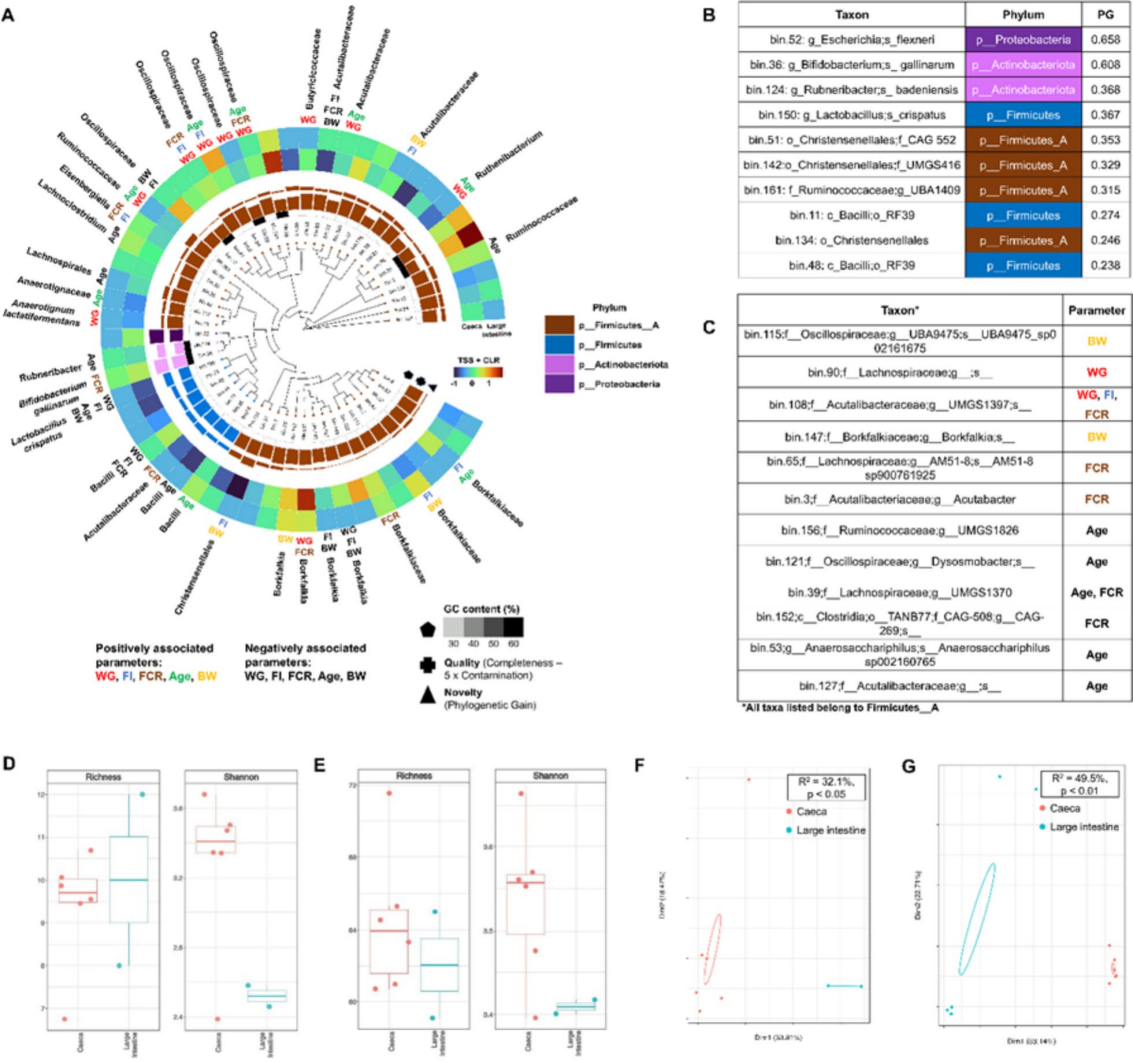
Performance parameters and their association with key microbes and functions. Major bacterial species colonising the chicken caeca and large intestine indicated in phylogenetic tree (**a**). Genomic GC content is shown using grey gradient, quality score (genome completion – 5 × genome contamination), phylogenetic gain (PG) and sample abundances (heatmap; TSS + CLR) for each of the recovered MAGs. Families are shown in colours. Family's positive associations to parameters (weight gain (WG), age, feed conversion rate (FCI), feed intake (FI) and body weight (BW) are colour coded, and the negative associations to parameters are shown in black. Phylogenetic gain (**b**) was calculated using GTDBTK toolkit with higher values representing novelty of a particular genome within the context of the phylogenetic tree; the 10 most novel MAGs are shown. MAGs not recovered in the phylogenetic tree shown in (**c**). Metabolic taxa diversity represented by rarefied Richness and Shannon entropy in both organs are shown in (**d**) and metabolic dbCAN2 diversity in (**e**). A PCA sample analysis represented by Dim1, dimension 1; Dim2, dimension 2 shows taxa differences between both organs in (**f**) and differences in metabolic dbCAN2 are shown in (**g**)

The majority of the MAGs were associated with four different phyla based on GTDB-TK V2.4.0 taxonomy with *Firmicutes A* indicated as the most dominant phylum (Fig. 2) and were resolved at family level as shown in Fig. 2a. Furthermore, using CODA-LASSO algorithm, MAGS with positive associations to performance parameters were identified. These parameters included weight gain, age, FCR, FI, and BW (highlighted with different colours). Negative associations to the same parameters were also identified (highlighted in black). Phylogenetic Gain (PG) was also calculated using the phylogenetic tree, and was used as a proxy for novelty (calculated using genometreetk utilities from GTDTB-TK suite) [22]. Genome Database Taxonomy toolkit (GTDBTK) was performed where higher values represent novelty of a particular genome within the context of the phylogenetic tree. The 10 most novel MAGs are shown in Fig. 2b which came from four distinct phyla. bin.52 (Gammaproteobacteria Burkholderiales) was found to be the most novel bin followed by bin.36 (Bifidobacterium *Bifidobacterium gallinarum*). At family level, *Oscillospiraceae, Ruminococcaceae, Borkfalkiaceae, Acutalibacteraceae* were found to be positively associated with the parameters, age, BW, FI, G and FCR. Changes in the feeding regime resulted in changes in association to the dependent parameters for instance the genus *Borkfalkia* changed from positive association to G, FCR and BW into negative association.

At the genus level *g__UBA5446* (Clostridiales bacterium) abundance was found positively associated with weight gain, *g__UBA11940* (*Borkfalkiaceae*) with FI, BW and FCR, *g__ UBA1417* with FI and BW and *g__ CAG − 180* (*Acutalibacteraceae*) with weight gain and age, *g__ AM07 − 15* (*Ruminococcaceae* bacterium) with weight gain, *Bifidobacterium gallinarum* and *g __ CAG − 822* to FCR and *g__ CAG − 460* (*Bacilli f__UBA660*) with age. Meanwhile, the results showed a negative association of *g__ UBA1375* s *UBA1375* (*Ruminococcaceae*), *g__ UBA1390* s *UBA1390* (Lachnospirales) and *Lactobacillus crispatus* with age. The results showed with advancing ages *Ruminococcaceae*; g__ *UBA1409 UBA1409 Bacilli g__ CAG − 460, f Anaerotignaceae; g__ UBA8514,*

*Ruthenibacterium, Acutalibacteraceae; g__ CAG − 180 CAG − 180* were abundant. Whereas *Ruminococcaceae; g__ UBA1375 UBA1375, Lachnospiraceae; g Lachnoclostridium_A,* Lachnospirales*; f UBA1390; g__ UBA1390 UBA1390, Bacilli; g__ UMGS1217 and Rubneribacter Rubneribacter badeniensis* were negatively associated with age. MAGs missing in the phylogenetic tree (by virtue of not finding enough single copy genes) are shown in Fig. 2c. Metabolic taxa diversity was observed in terms of the alpha diversity in both organs represented by rarefied richness and Shannon entropy Fig. 2d & e. In addition, beta analysis was performed using Bray–Curtis distance in Principal Coordinate. Analysis showed different clusters for both organs suggesting a difference in microbial composition and metabolic dbCAN2 differences between both organs Fig. 2f & g. The performance parameters and their association with key microbes and functions are provided in Supplementary Table S2.

### Nutrient cycles and the role of hydrogen buildup in characterising the chicken gut microbiome

**Fig. 3:**
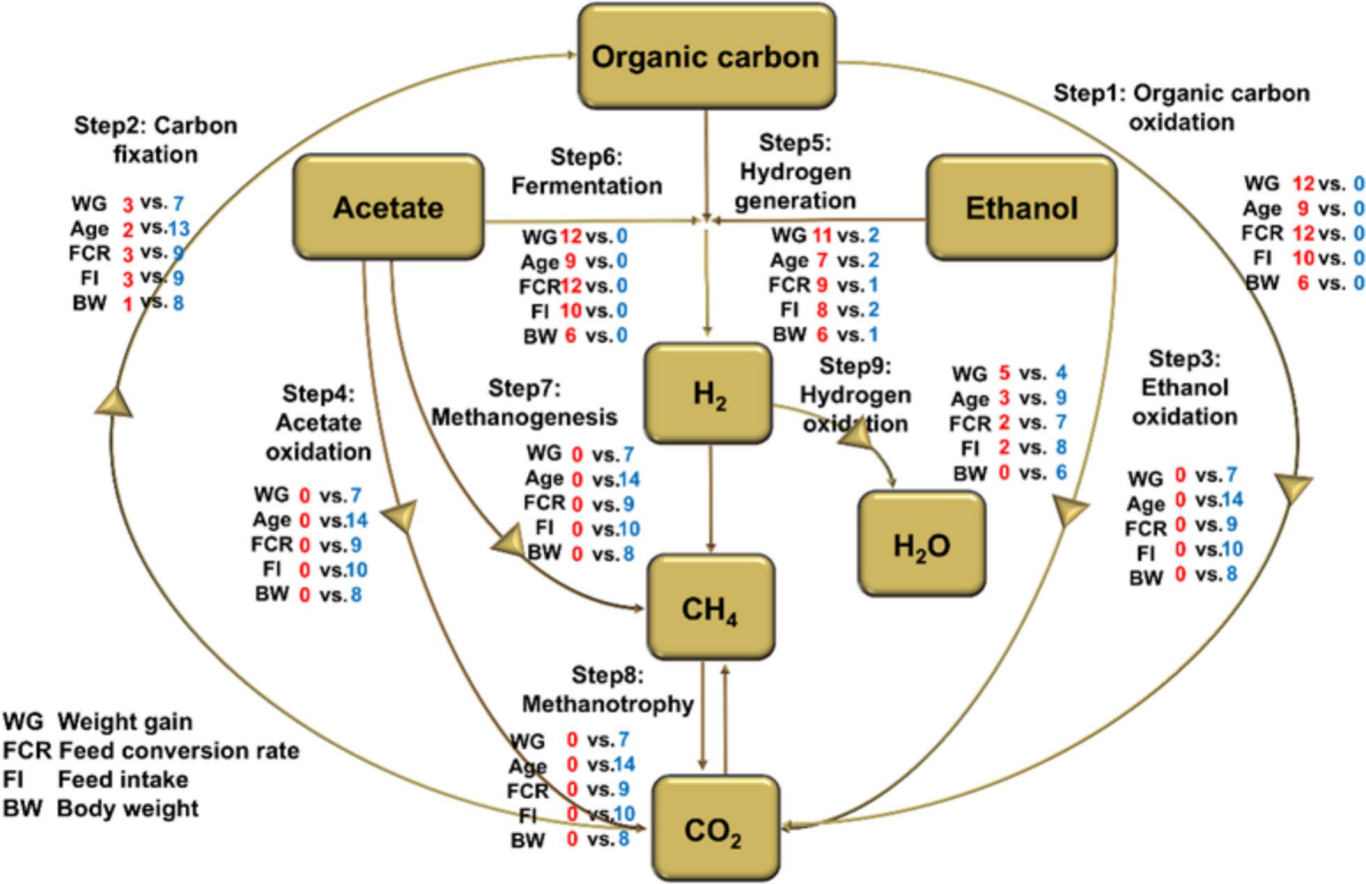
Geochemical cycles recovered from METABOLIC with the count showing the number of genomes that have a particular pathway, along with the coverage information for all genomes recovered in this dataset. Associations to parameters (WG, age, FCR, FI and BW), changes are indicated showing the number of genomes with positive association to parameters changes (red), and the number of genomes with negative associations to parameters changes (blue). The major steps involved in the carbon cycle are shown in this figure. The arrows represent those pathways that were substantially more abundant in the community after the changes. Other cycles (a-i) are shown in Supplementary Fig. S1. These associations are based on CODA LASSO analysis

Using METABOLIC, we recovered major geochemical cycles at both MAGs and community level Fig. 3 (carbon cycle) and Supplementary Fig. S1 (rest of the cycles) with MAGs-wise geochemical cycles provided in Supplementary Data 2. All the recovered MAGs (53, 100% coverage) contained pathways involved in carbon oxidation and fermentation (Step 1 and Step 6), with the figure showing the number of MAGs positively associated with weight gain, age, FCR, FI and BW were 12, 9, 12, 10, and 6, respectively. For hydrogen generation (Step 5), more MAGs (11, 7, 9, 8, 6) were positively associated with the weight gain, age, FCR, FI and BW, respectively, than those MAGs that were negatively associated (2, 2, 1, 2, 1). For Hydrogen oxidation (Step 9), more MAGs (4, 9, 7, 8, 6) were negatively associated with the parameters weight gain, age, FCR, FI, and BW, respectively than those that were positively associated MAGs (5, 3, 2, 2, 0).

Overall nitrogen and sulphur cycles for the recovered MAGs are shown in the Supplementary Fig. S1. For nitrogen fixation and nitrate reduction Steps 1 and 4, more MAGs (7, 12, 8, 9, 8) were negatively associated with the weight gain, age, FCR, FI, and BW, respectively, than those that were positively associated (1, 0, 0, 0, 1). Similar patterns were observed for sulphur oxidation and sulphite reduction Steps 3 and 6 respectively. Furthermore, for pathways involved in arsenate reduction, more MAGs were positively associated with the parameters especially weight gain and FCR. For selenate reduction, more MAGs were negatively associated with the parameters especially age. The association of MAGs with the steps of nutrient cycles are provided in Supplementary Table S3.

### Signature profiles and prediction of KEGG module abundance in association to parameters

**Fig. 4:**
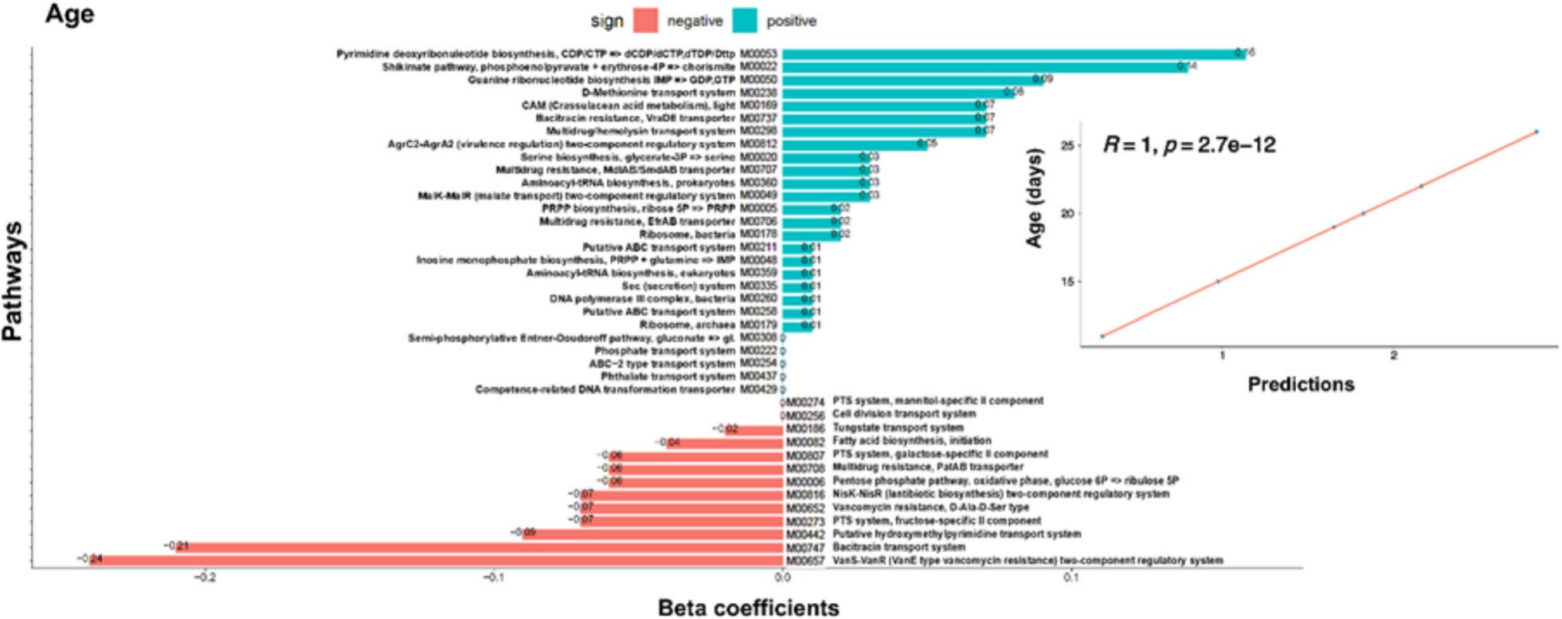
CODA-LASSO regression of age regressed against KEGG modules abundances [17]. Non-zero *β-coefficients* returned from CODA-LASSO procedure are shown as two disjoint sets (those that are increasing with age (positive; green) and those that are decreasing with age (negative; red). The insets show prediction quality of fitting with the predictions from CODA-LASSO procedure shown on the x-axis and the actual values of age shown on the y-axis. Regressions of KEGG modules against other parameters (weight gain, FCR, FI and BW) are provided in the Supplementary Fig. S2

Based on CODA-LASSO analysis, non-zero significant β-coefficients for KEGG modules (using either of the parameters, weight gain, age, FCR, FI, and BW) are shown in Fig. 4 (age) and Supplementary Fig. S2 (rest of the parameters). The positive association to the parameters is represented in blue bars, whilst negative associations are represented in red bars. The most significant associations were M00260 (DNA polymerase III complex, bacteria), M00335 (Sec (secretion) system), M00258 (Putative ABC transport system), M00179 (Ribosome, archaea), M00178 (Ribosome, bacteria). These modules were positively associated with FI, age, and BW, respectively, and negatively associated with FCR. M00731 (Bacitracin transport system) was positive associated with weight gain, FI, age, and BW; and negatively associated with age. M00657 (VanS-VanR (VanE type vancomycin resistance) two-component regulatory system), and M00442 (Putative hydroxymethylpyrimidine transport system) were negatively associated with FI, age, and BW. M00708 (Multidrug resistance, PatAB transporter) was negatively associated with weight gain, and age; M00652 (Vancomycin resistance, D-Ala-D-Ser type) was negatively associated with age whereas M00707 (Multidrug resistance, MdlAB/SmdAB transporter) and M00706 (Multidrug resistance, EfrAB transporter) were positively associated with age. The association of performance parameters with the abundance of KEGG modules abundance is provided in the Supplementary Table S4.

**Fig. 5:**
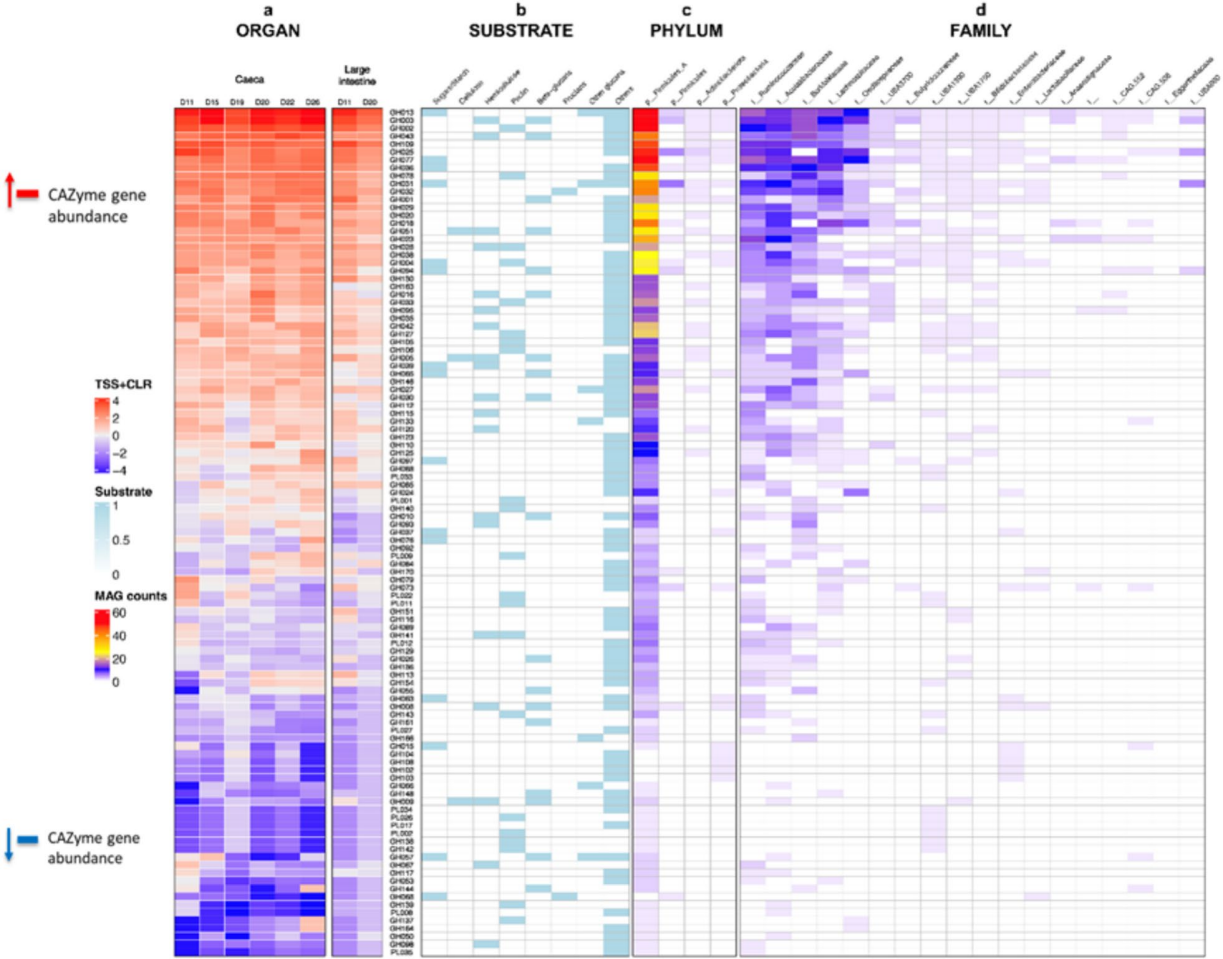
Carbohydrate-active enzymes (CAZyme) gene abundance recovered from Caeca and large intestine: (**a**) Mean normalised abundance of CAZyme gene IDs; Red and blue colour of heatmap cells indicates high and low abundance, respectively); in both organs. CAZyme IDs grouped according to (**b**) substrate/function based on the dbCAN2) across both organs. Number of MAGs containing CAZyme genes, grouped according to (**c**) phylum and (**d**) family taxonomic ranks, comparison of total glycoside hydrolase (GH) and polysaccharide lyase (PL) abundances across both organs

In addition, metabolic functions in both organs were explored. Enzymes including CAZymes are important for breakdown of dietary carbohydrates and fibres, and therefore, they play a vital role in the metabolism and reproduction of chickens. The association of performance parameters with the abundance of CAZymes is provided in the Supplementary Table S5. We detected a total of 108 CAZymes belonging to two major families – glycoside hydrolase (GH) and polysaccharide lyase (PL), with the former being more dominant (Fig. 5). CAZyme families varied significantly in abundance for both organs for the selected age measured in this study [11, 15, 19, 20, 22, 26]. The most common enzyme identified within both organs is GH013 which was detected in 17 bacterial families in our study. GH093, GH076, and Pl001 which metabolise hemicellulose, sugars/starch, and pectin, respectively, were only found in the family *Borkfalkiaceae*. GH077 and GH015 were only found in the caeca on day 11 whereas, GH104, GH108, GH102 and GH103 were found in the large intestine on day 20. In the current study: a total of 13 PLs were found on day 20; seven enzymes (PL033, PL027, PL012, PL034, PL017, PL008 and PL035) were found in the large intestine; and six enzymes (PL001, PL009, PL022, PL011, PL026, PL002) were found in the caeca. None of the enzymes were shared in both organs.

## Discussion

Poultry performance parameters such as BW, FI, FCR, and weight gain are indicators of good farming [4, 23]. Previously, using 16S rRNA sequencing, we highlighted that microbial variation over time is most likely influenced by the diet of chickens whereby significant shifts in taxa abundances and beta dispersion of samples were often associated with changes in feed [5]. In this study, we used the same parameters, and performed shotgun metagenomics on the caeca and large intestine of chickens. Our study corroborates the previous findings that age plays a key role in defining variation in taxonomic composition. These results are in agreement with Li et al. [24] that examined the diversity of gut bacterial communities in chickens, and showed that 90% of amplicon sequence variants belonged to *Firmicutes* and *Proteobacteria*. The chicken gut microbiota is primarily colonised by facultative anaerobes, with a gradual transit from simple to complex and obligate anaerobes occurring with age and ultimately reaching a relatively stable dynamic state [25]. The recovered patterns further substantiate that some facultative anaerobes are negatively associated with the age of bird such as *Lactobacillus crispatus*. Nonetheless, with age, chickens will be predisposed to infectious diseases due to the ability of *L. crispatus* to interfere with pathogenic bacteria via colonisation, competitive exclusion and production of antimicrobial compounds [26].

Carbohydrates are the major content of chicken diet, which approximately include 70% starch, oligosaccharides, and non-starch polysaccharides (NSP) such as cellulose, hemicellulose and pectin [27]. Dietary fibre is referred to as soluble or insoluble non-starch polysaccharides (NSP) and lignin [28]. The lack of enzymatic capacity in chickens to digest NSPs allow NSPs to accumulate, providing the opportunity to modulate the digestive activity via interaction with the gut microbiome which possess a diverse range of CAZymes. This results in changes to the nutrient utilisation and growth performance [29]. Our study revealed differences in functioning CAZymes in both caeca and large intestine especially on day 20 where different polysaccharide lyase enzyme activity was noted in both organs. Pectin was the preferred substrate by caecal microflora on day 20, whereas others were utilised as substrates in the large intestine. This may be due to the reduction of easily digestible growth substrates as they move down the gastrointestinal tract. Based on CAZymes, we hypothesise that the preferred substrates, such as starch and pectin, are digested in the upper intestines. Consequently, other glycans substrates are utilised by caecal microbiota [14]. The working hypothesis is that bacteria in the lower intestine are often better at utilising feed components such as non-starch polysaccharides, resistant starch or resistant protein [30]. Pectin has high water solubility and its ability to form a gel lends itself for easy fermentation by the intestinal microflora [31]. These soluble components act as a source of energy for bacteria, allowing them to use other nutrients such as nitrogen as substrates for the production of metabolites [27].

The breakdown of dietary fibres and other indigestible compounds are necessary to create more accessible products such as SCFAs [32]. SCFAs contribute to the nutrition of the chicken and improve mineral absorption via lowering the pH which can inhibit the growth of acid-sensitive pathogens [33]. As a fermentation byproduct, the production of SCFAs will generates large amounts of free hydrogen and the accumulation of hydrogen has the ability to inhibit fermentation [34] It is well established that some species of bacteria and archaea found in the chicken gut microbiome are also able to provide the enzymes that assist hydrogen consumption, and this in turn allows intestinal fermentation to continue [33]. For example, *Megamonas, Wolinella*, *Helicobacter*, and *Campylobacter* (including *C. jejuni*) are able to produce nickel–iron hydrogenases. The acetyl-coenzyme A synthase is produced by bacteria of the *Lachnospiraceae* family, and methyl-coenzyme M reductase is produced by methanogenic archaea [33]. We have also shown that variation in microbial composition resulted in a community with the ability of hydrogen generation and is positively associated with weight gain, age, FCR, FI and BW respectively, when compared to MAGs that were negatively associated with the mentioned parameters. The majority of the recovered genomes have hydrogen metabolism pathways which lend themselves to hydrogen consumption and thus the hydrogen sick hypothesis (Supplementary Data 2).

## Conclusions

This study explores changes in microbial diversity (and their function) of chicken caeca and large intestine with respect to performance parameters.. Our study highlights association of metabolic pathways with the performance parameters emphasizing on hydrogen generation playing a pivotal role. Further interrogation with a larger sample size as well as controlled experimentation is needed to establish the importance of hydrogen cycling to improve health of chicken. This study advances understanding of the metabolic pathways that influence the type of bacteria present in different organs, which will ultimately lead to developing intervention and control strategies against enteric pathogens.

## Limitations

We acknowledge that the sample size in our study is a limitation. However, this did not hinder the recovery of a significant number of Metagenome-Assembled Genomes (MAGs) compared to other studies with larger sample sizes that recovered fewer MAGs [14, 35].

## Supplementary Information

Supplementary file 1. Samples sequenced from four organs (Small Intestine, Large Intestine, Caeca, and Duodenum) and sample selection based on Phyloflash results.

Supplementary file 2. Recovered nutrient cycles including Carbon, Nitrogen, and Sulphur cycles for all the metagenomic assembled genomes given as PDF images, and identifiable through bin numbers.

Supplementary file 3. Fig. S1. Geochemical cycles (a-i) recovered from METABOLIC with count showing the number of genomes that have a particular pathway, along with the coverage information for all genomes recovered in this dataset. Associations to parameters (weight gain, age, feed conversion rate, feed intake and body weight) changes are indicated showing the number of genomes with positive association to parameters changes (red), and the number of genomes with negative associations to parameters changes (blue)(e). The arrows (a-c) represent those pathways that were substantially more abundant in the community after the changes. These associations are based on CODA LASSO analysis.

Supplementary file 4. Fig. S2: CODA-LASSO regression of the parameters weight gain, feed intake, feed conversion ratio and body weight regressed against KEGG modules abundances [17] were indicated in (a-d) respectively. Non-zero *β-coefficients* returned from CODA-LASSO procedure are shown as two disjoint sets (those that are increasing with parameters (positive; green) and those that are decreasing with parameters (negative; red). The insets show prediction quality of fitting with the predictions from CODA-LASSO procedure shown on the x-axis and the actual values of parameters shown on the y-axis.

Supplementary file 5. Statistical methods used in the study.

Supplementary file 6. Table S1: GTDB-TK taxonomy at different taxonomic levels for the MAGs used in this study

Supplementary file 7. Table S2: Phylogenetic Gain (PG) scores for different MAGs along with their positive/negative association recovered using CODA-LASSO procedure. Here the acronyms denote WG: Weight Gain; FCR: Feed Conversion Ratio; FI: Feed Intake; Age, and BW: Body Weight. The + or - next to these acronyms represent increase or decrease, respectively.

Supplementary file 8. Table S3. Association of MAGs with different geochemical cycles, whether a constituent step is observed or not (highlighted by “None”).

Supplementary file 9. Table S4: Results of Supplementary Fig 2 shown in tabular form with pathway-centric view. Here the acronyms denote WG: Weight Gain; FCR: Feed Conversion Ratio; FI: Feed Intake; Age, and BW: Body Weight. The + or - next to these acronyms represent increase or decrease, respectively.

Supplementary file 10. Table S5: The performance parameters and their association with CAZymes abundance. Here the acronyms denote WG: Weight Gain; FCR: Feed Conversion Ratio; FI: Feed Intake; Age, and BW: Body Weight. The + or - next to these acronyms represent increase or decrease, respectively.

## Data Availability

The sequencing datasets generated and/or analysed during the current study are available in the ENA repository Accession PRJEB88012.

## Abbreviations

ANOVA: Analysis of variance
BW: Body weight
*C. jejuni*: *Campylobacter jejuni*
CAZymes: Carbohydrate active enzyme
FCR: Feed conversion ratio
GC: Guanosine-cytosine
gDNA: Genomic DNA
GH: Glycoside hydrolase
LASSO: Least absolute shrinkage and selection operator
MAGs: Metagenome-assembled genomes
NSP: Non-starch polysaccharide
PCA: Principal component analysis
PCR: Polymerase chain reaction
PG: Phylogenetic gain (Novelty)
PERMANOVA: Permutational multivariate ANOVA
PL: Polysaccharide lyase
R: Richness
SCFAs: Short-chain fatty acids
SCG: Single copy genes
TSS + CLR: Total sum scaling and centralised log ratio
WG: Weight gain
