# Supplementary figures and images for: Investigating microbial population structure and function in the chicken caeca and large intestine over time using metagenomics (part 2 of 2)
Source: BMC Res Notes. 2025 Aug 15;18:355. doi: 10.1186/s13104-025-07441-7 (PMC12357378; doi:10.1186/s13104-025-07441-7)

# Carbon Cycle: bin.40

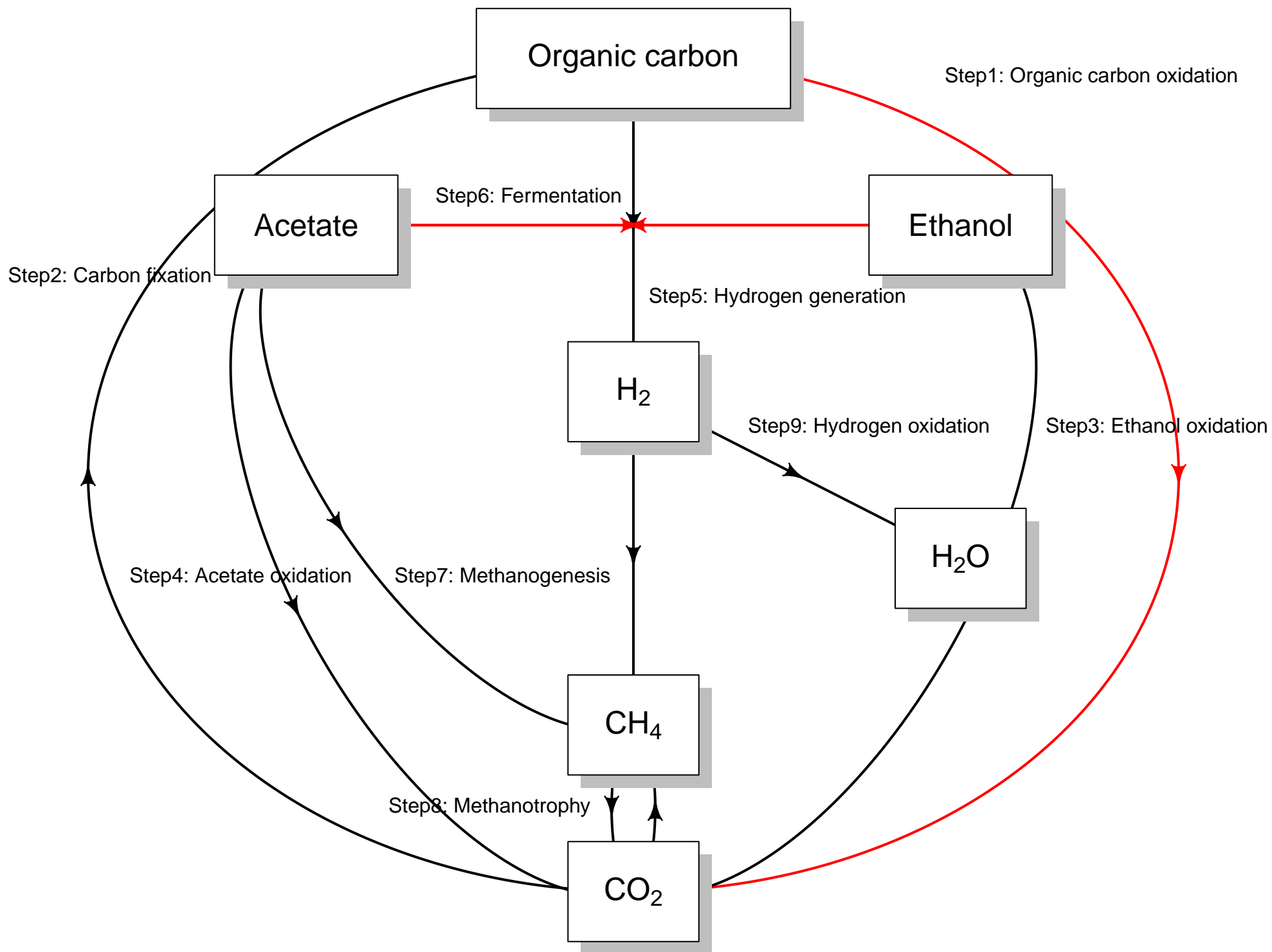

Supplement: Supplementary file 2 — Recovered nutrient cycles including Carbon, Nitrogen, and Sulphur cycles for all the metagenomic assembled genomes given as PDF images, and identifiable through bin numbers. [file 13104_2025_7441_MOESM2_ESM.zip › Supplementary Data2/bin.40.draw_carbon_cycle_single.pdf]

## Nitrogen Cycle: bin.78

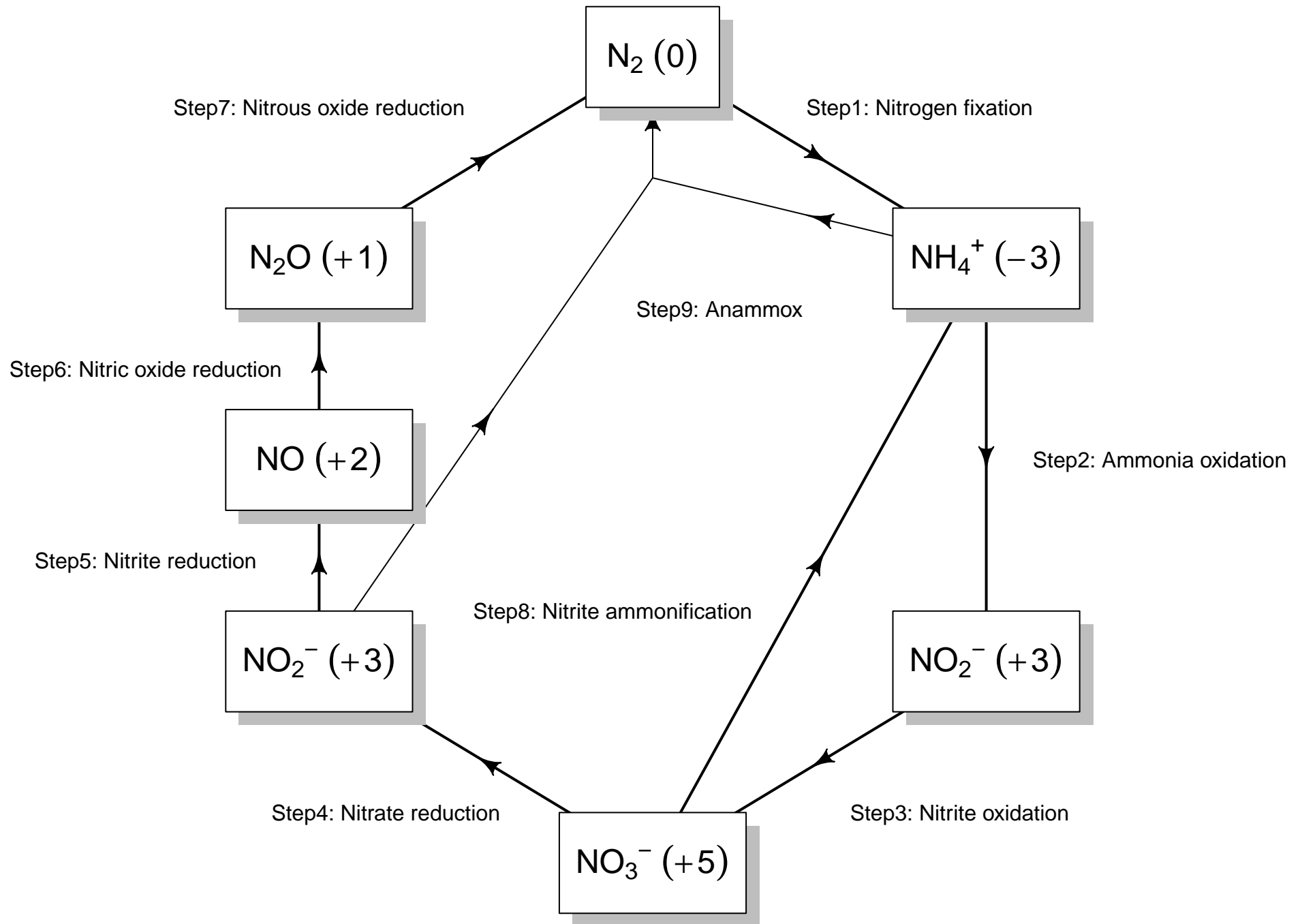

Supplement: Supplementary file 2 — Recovered nutrient cycles including Carbon, Nitrogen, and Sulphur cycles for all the metagenomic assembled genomes given as PDF images, and identifiable through bin numbers. [file 13104_2025_7441_MOESM2_ESM.zip › Supplementary Data2/bin.78.draw_nitrogen_cycle_single.pdf]

# Carbon Cycle: bin.64

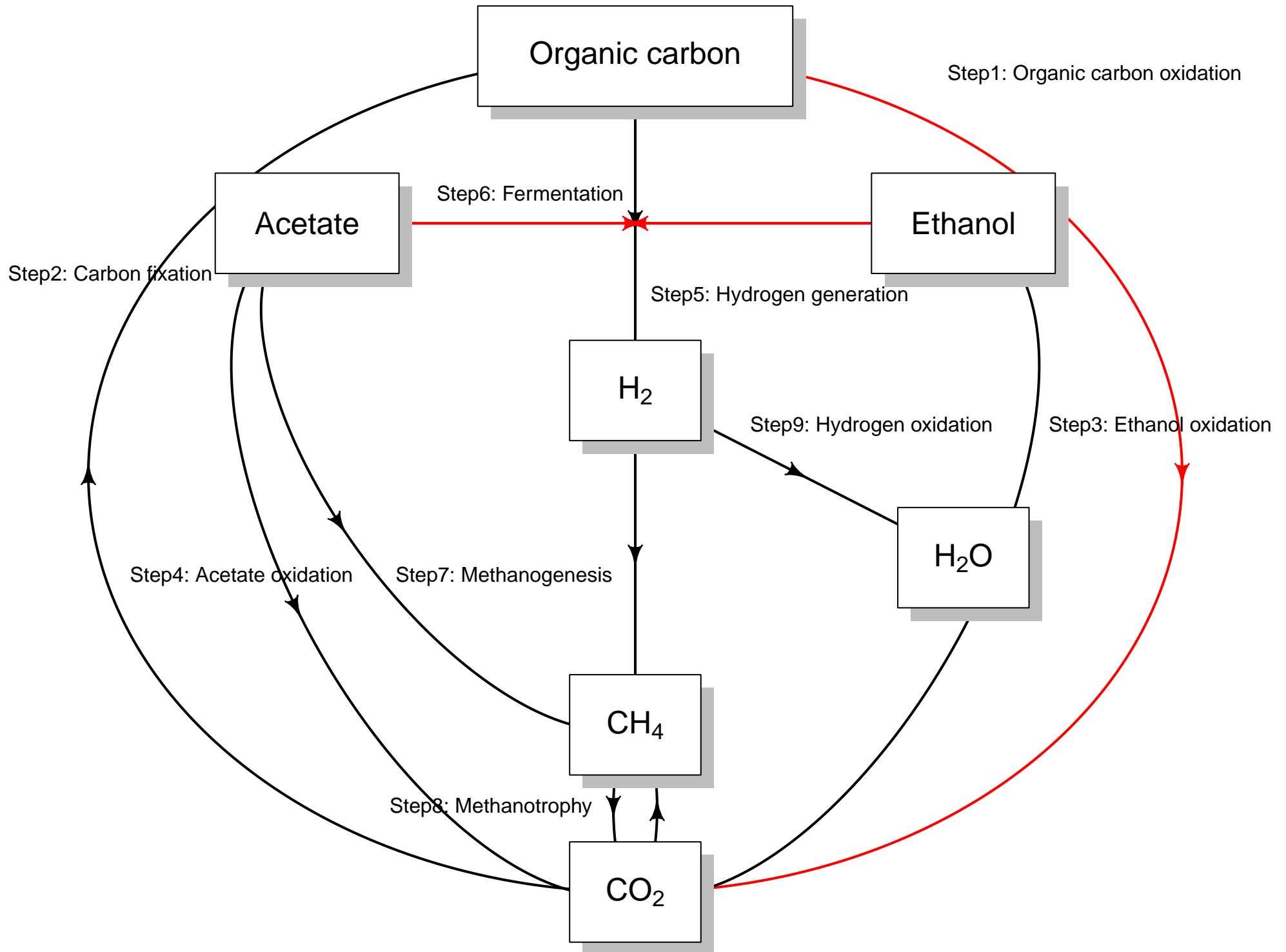

Supplement: Supplementary file 2 — Recovered nutrient cycles including Carbon, Nitrogen, and Sulphur cycles for all the metagenomic assembled genomes given as PDF images, and identifiable through bin numbers. [file 13104_2025_7441_MOESM2_ESM.zip › Supplementary Data2/bin.64.draw_carbon_cycle_single.pdf]

## Nitrogen Cycle: bin.37

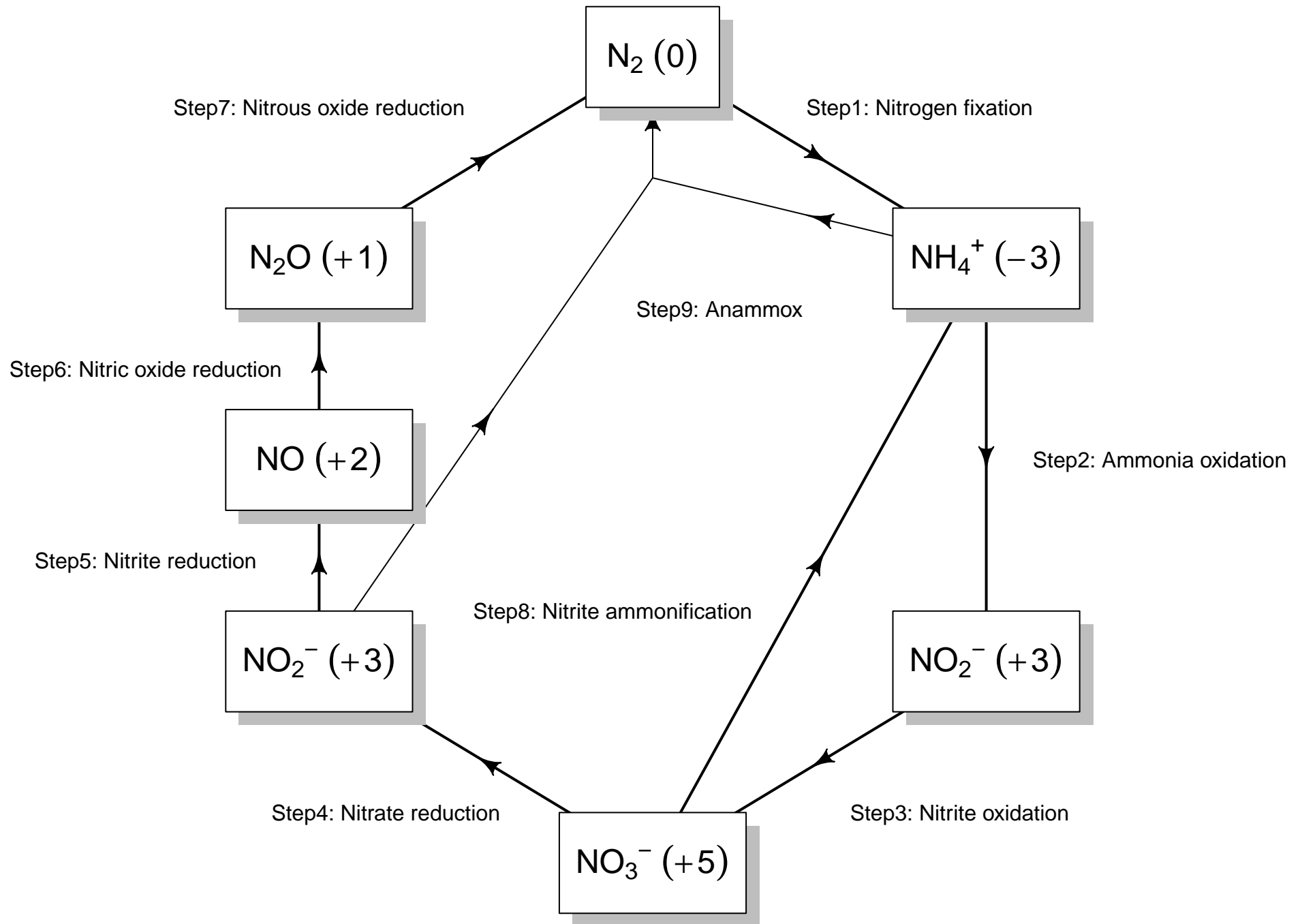

Supplement: Supplementary file 2 — Recovered nutrient cycles including Carbon, Nitrogen, and Sulphur cycles for all the metagenomic assembled genomes given as PDF images, and identifiable through bin numbers. [file 13104_2025_7441_MOESM2_ESM.zip › Supplementary Data2/bin.37.draw_nitrogen_cycle_single.pdf]

# Carbon Cycle: bin.157

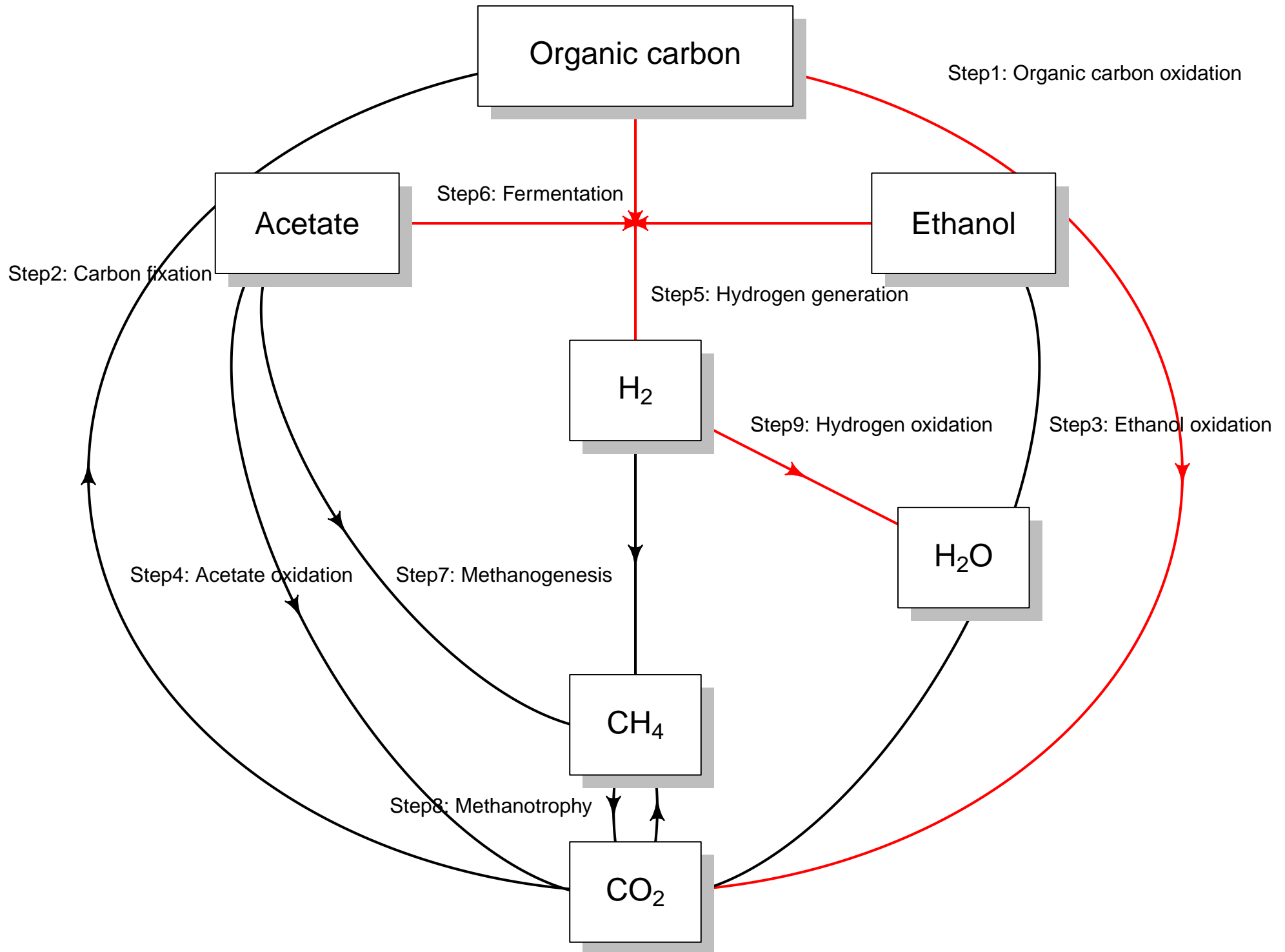

Supplement: Supplementary file 2 — Recovered nutrient cycles including Carbon, Nitrogen, and Sulphur cycles for all the metagenomic assembled genomes given as PDF images, and identifiable through bin numbers. [file 13104_2025_7441_MOESM2_ESM.zip › Supplementary Data2/bin.157.draw_carbon_cycle_single.pdf]

## Nitrogen Cycle: bin.8

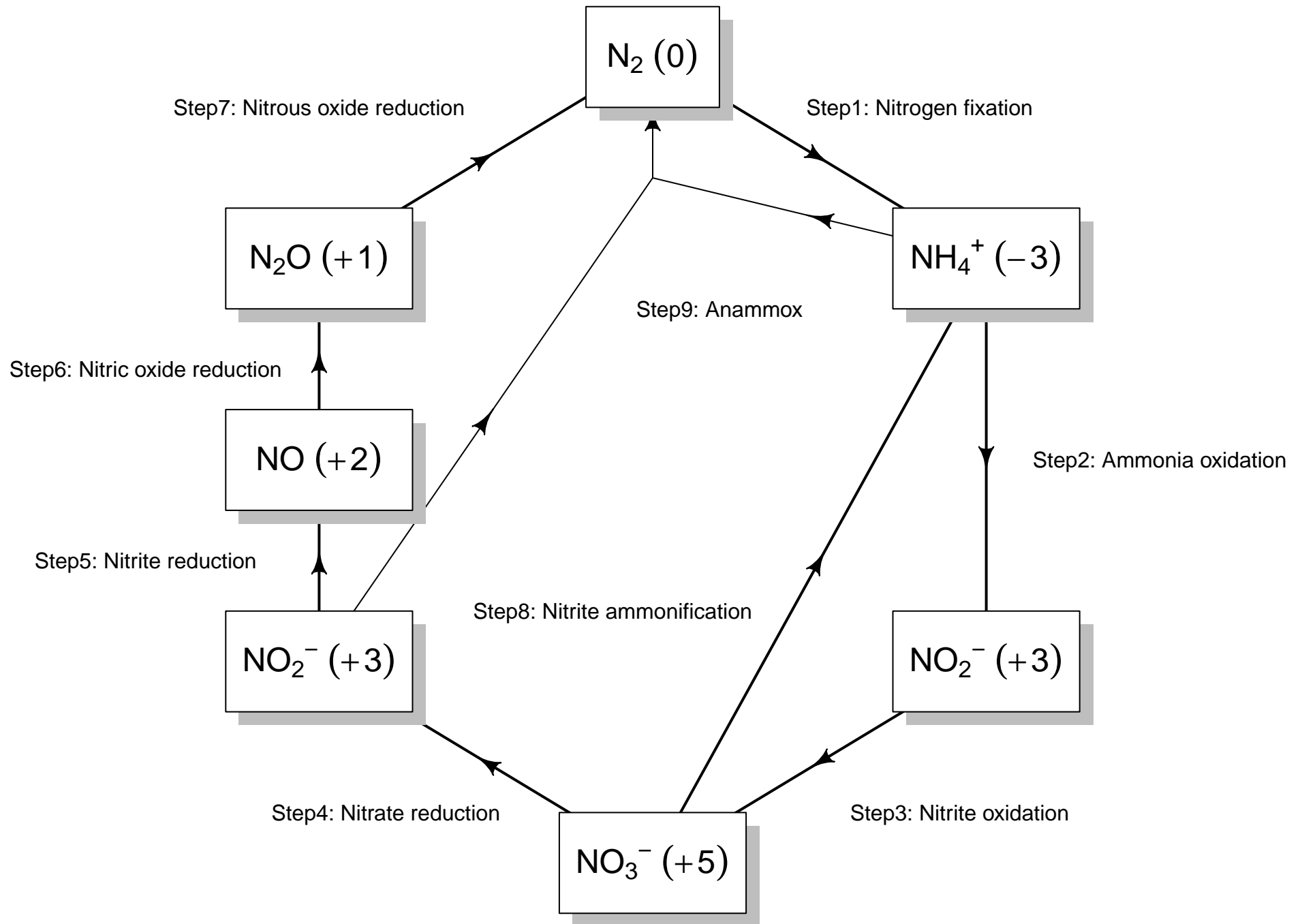

Supplement: Supplementary file 2 — Recovered nutrient cycles including Carbon, Nitrogen, and Sulphur cycles for all the metagenomic assembled genomes given as PDF images, and identifiable through bin numbers. [file 13104_2025_7441_MOESM2_ESM.zip › Supplementary Data2/bin.8.draw_nitrogen_cycle_single.pdf]

## Sulfur Cycle: bin.47

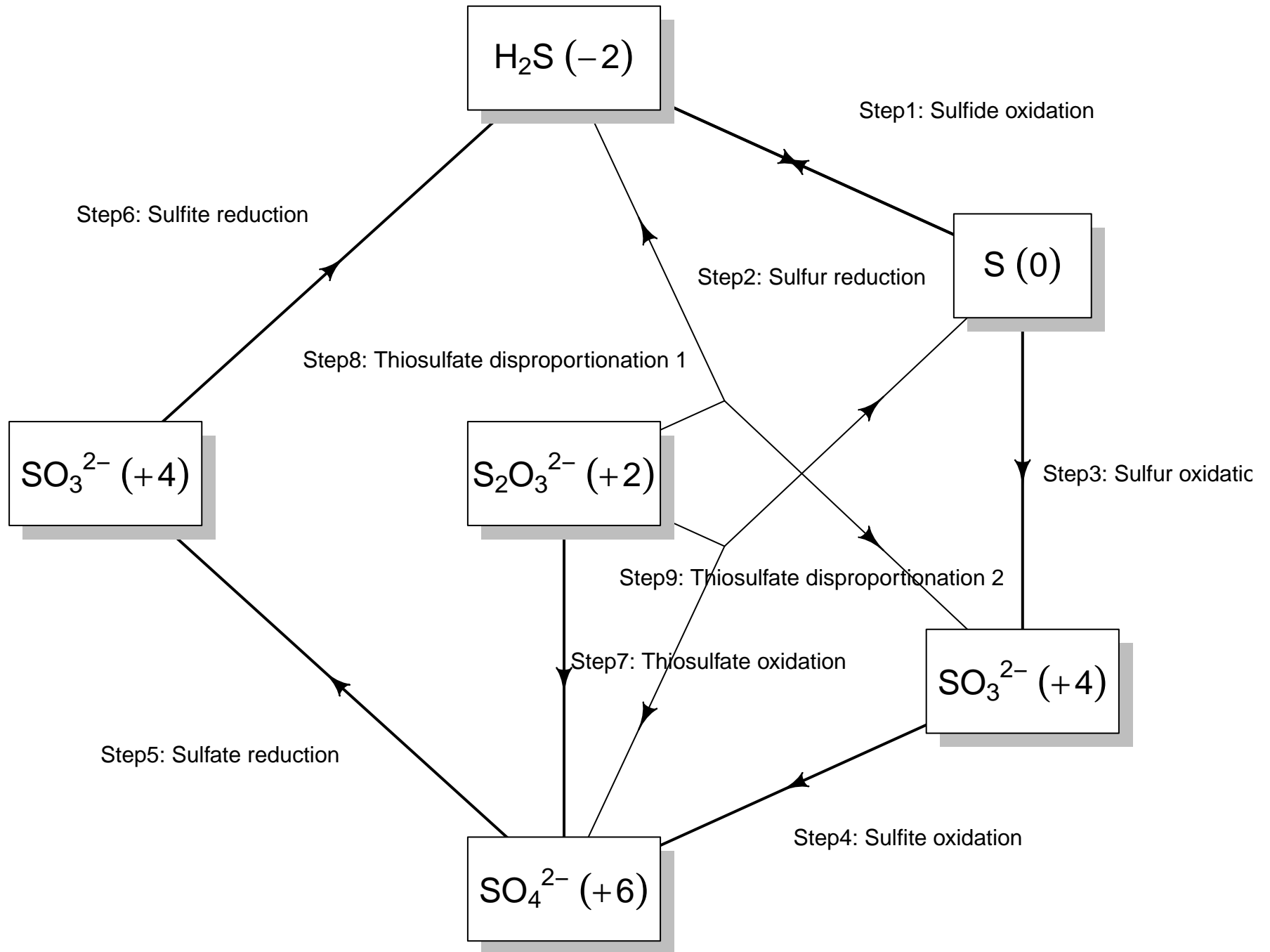

Supplement: Supplementary file 2 — Recovered nutrient cycles including Carbon, Nitrogen, and Sulphur cycles for all the metagenomic assembled genomes given as PDF images, and identifiable through bin numbers. [file 13104_2025_7441_MOESM2_ESM.zip › Supplementary Data2/bin.47.draw_sulfur_cycle_single.pdf]

## Nitrogen Cycle: bin.24

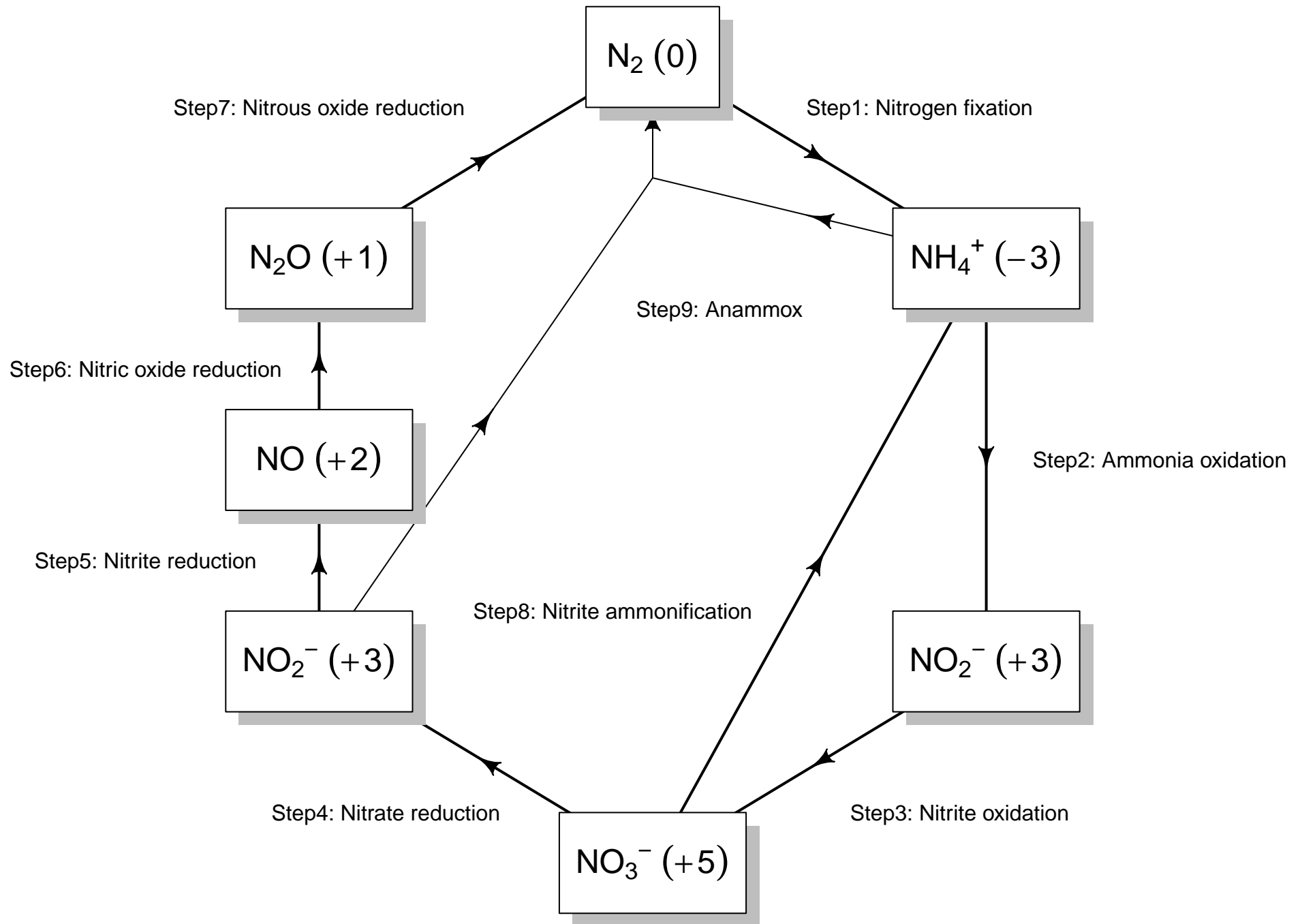

Supplement: Supplementary file 2 — Recovered nutrient cycles including Carbon, Nitrogen, and Sulphur cycles for all the metagenomic assembled genomes given as PDF images, and identifiable through bin numbers. [file 13104_2025_7441_MOESM2_ESM.zip › Supplementary Data2/bin.24.draw_nitrogen_cycle_single.pdf]

# Carbon Cycle: bin.159

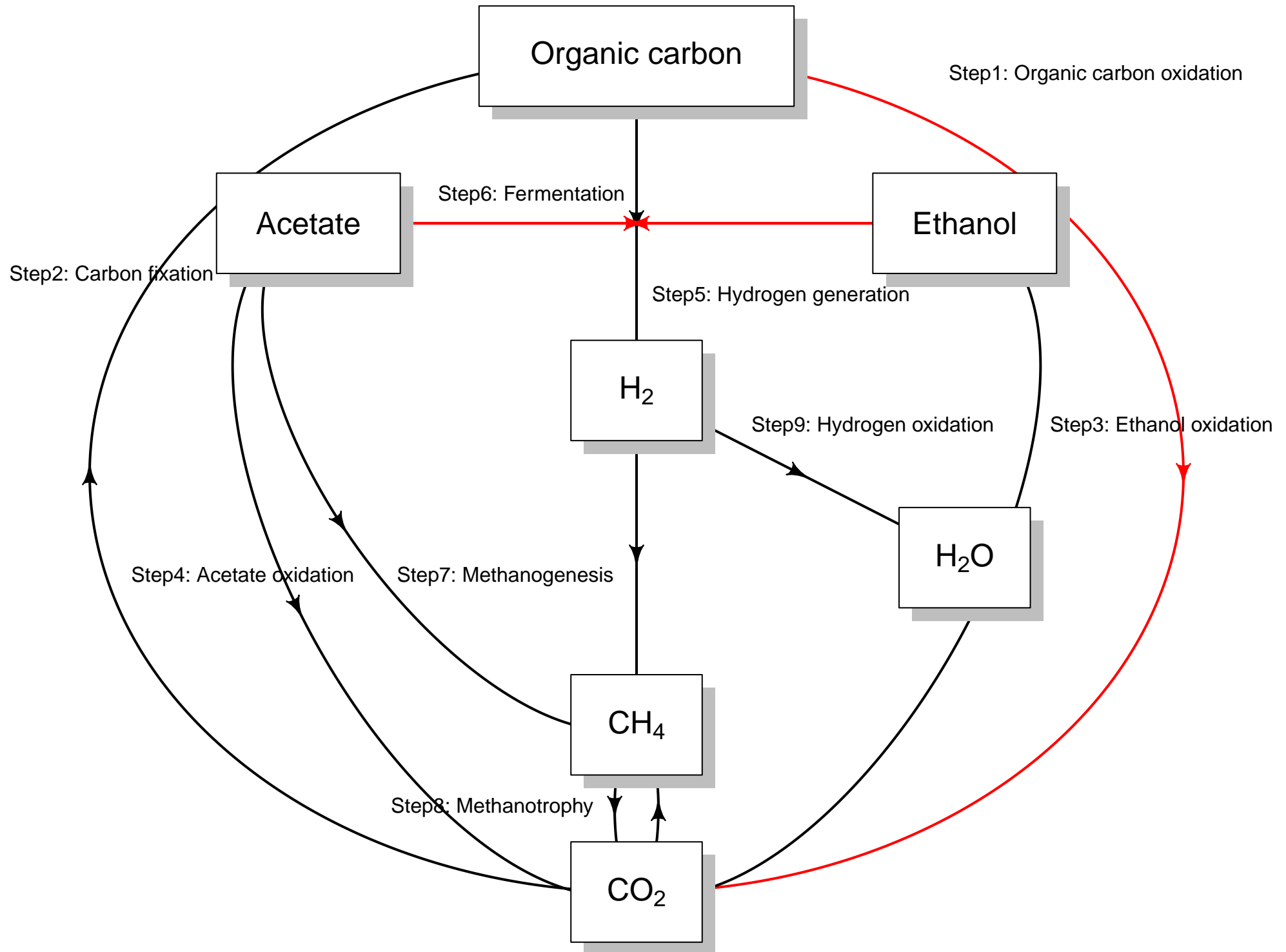

Supplement: Supplementary file 2 — Recovered nutrient cycles including Carbon, Nitrogen, and Sulphur cycles for all the metagenomic assembled genomes given as PDF images, and identifiable through bin numbers. [file 13104_2025_7441_MOESM2_ESM.zip › Supplementary Data2/bin.159.draw_carbon_cycle_single.pdf]

## Nitrogen Cycle: bin.160

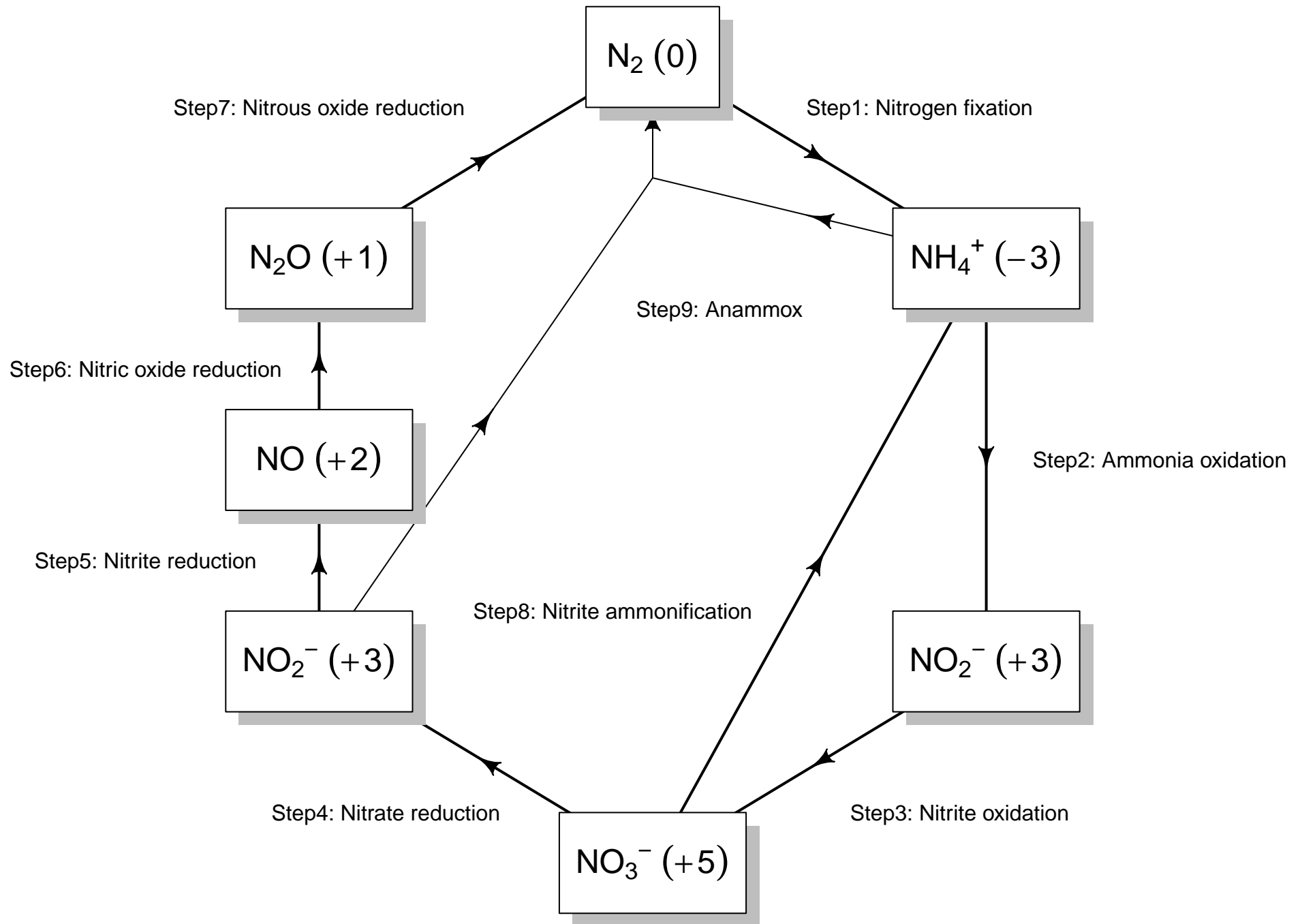

Supplement: Supplementary file 2 — Recovered nutrient cycles including Carbon, Nitrogen, and Sulphur cycles for all the metagenomic assembled genomes given as PDF images, and identifiable through bin numbers. [file 13104_2025_7441_MOESM2_ESM.zip › Supplementary Data2/bin.160.draw_nitrogen_cycle_single.pdf]

## Sulfur Cycle: bin.52

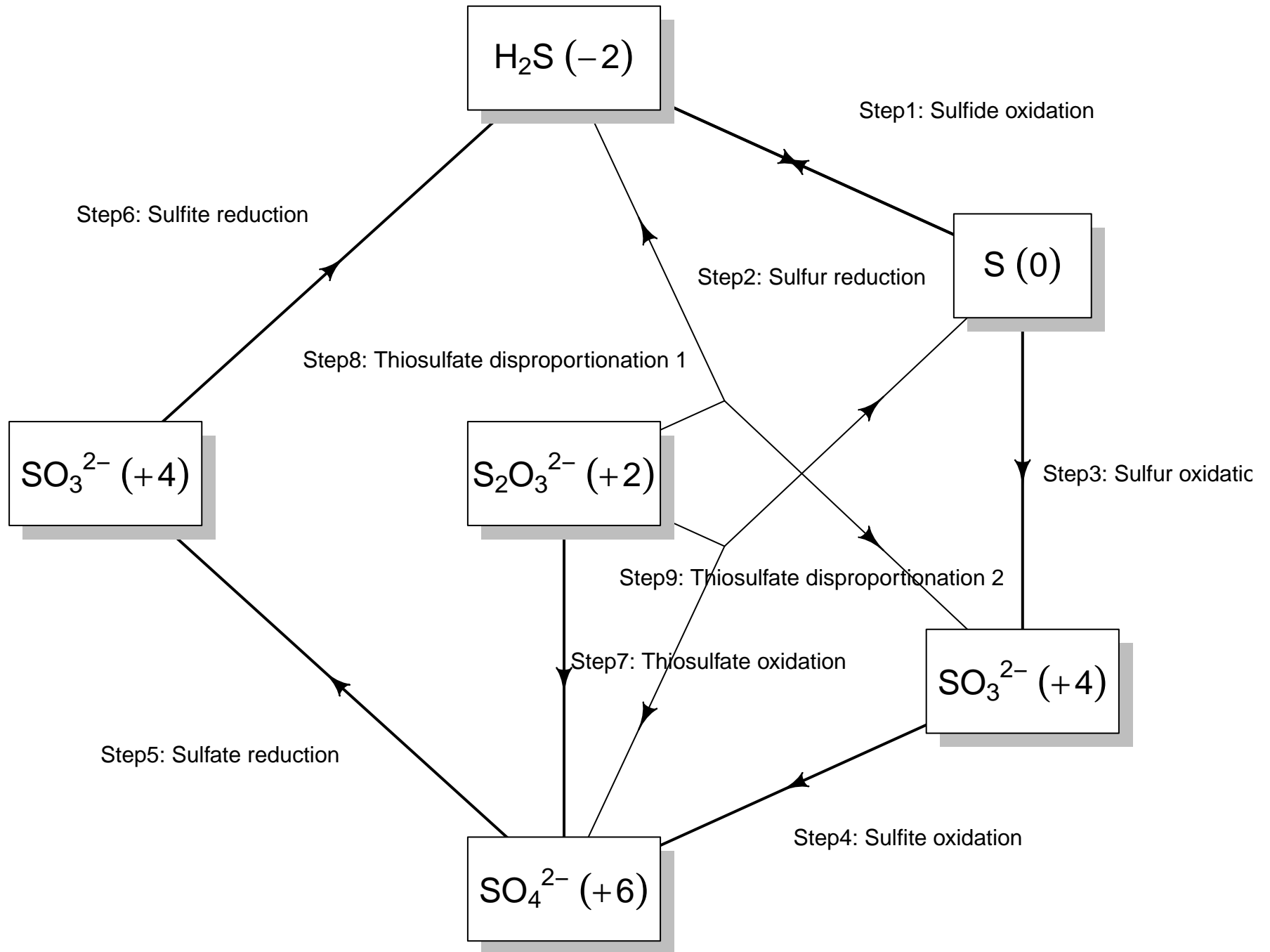

Supplement: Supplementary file 2 — Recovered nutrient cycles including Carbon, Nitrogen, and Sulphur cycles for all the metagenomic assembled genomes given as PDF images, and identifiable through bin numbers. [file 13104_2025_7441_MOESM2_ESM.zip › Supplementary Data2/bin.52.draw_sulfur_cycle_single.pdf]

# Carbon Cycle: bin.142

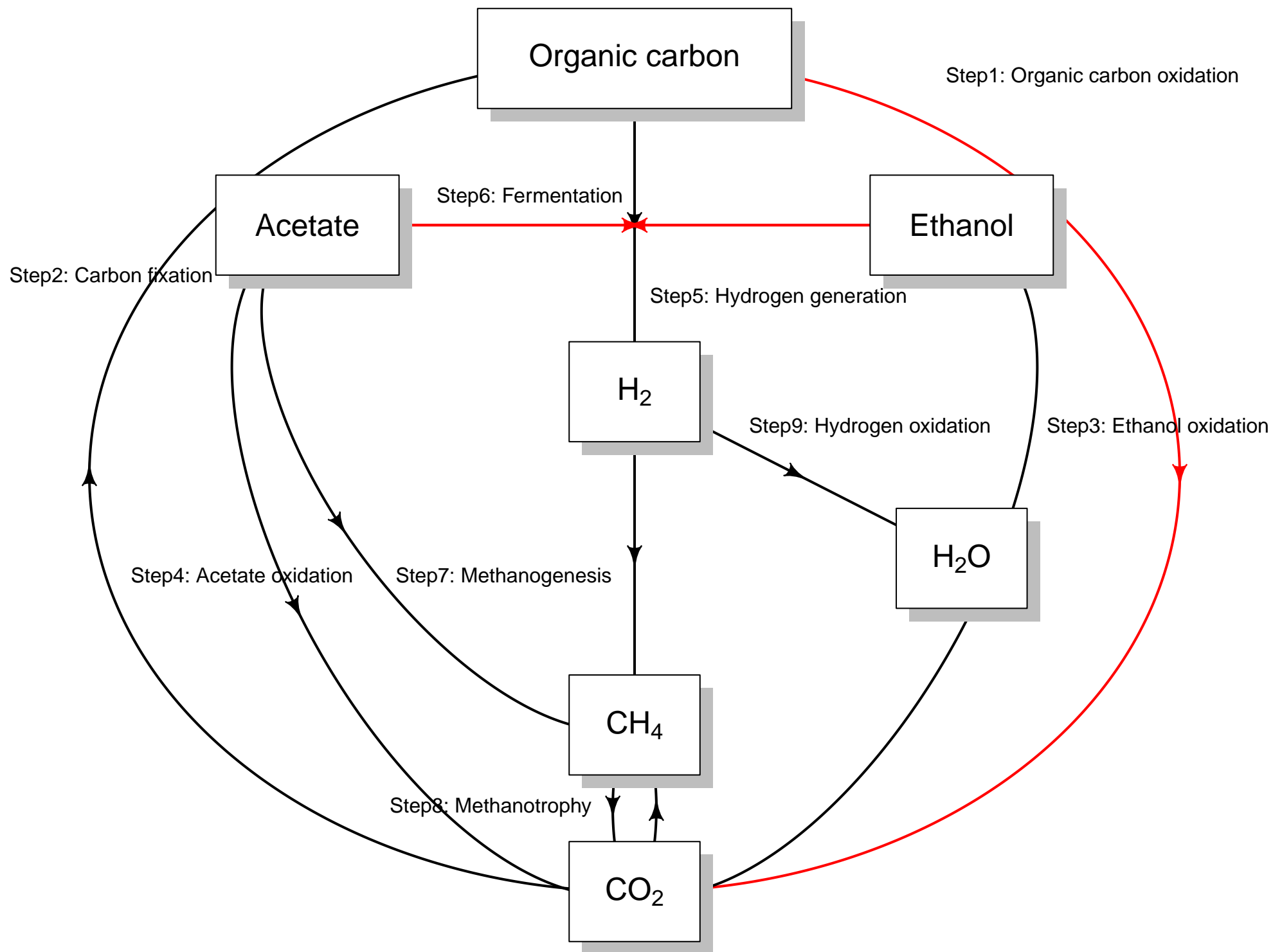

Supplement: Supplementary file 2 — Recovered nutrient cycles including Carbon, Nitrogen, and Sulphur cycles for all the metagenomic assembled genomes given as PDF images, and identifiable through bin numbers. [file 13104_2025_7441_MOESM2_ESM.zip › Supplementary Data2/bin.142.draw_carbon_cycle_single.pdf]

## Nitrogen Cycle: bin.93

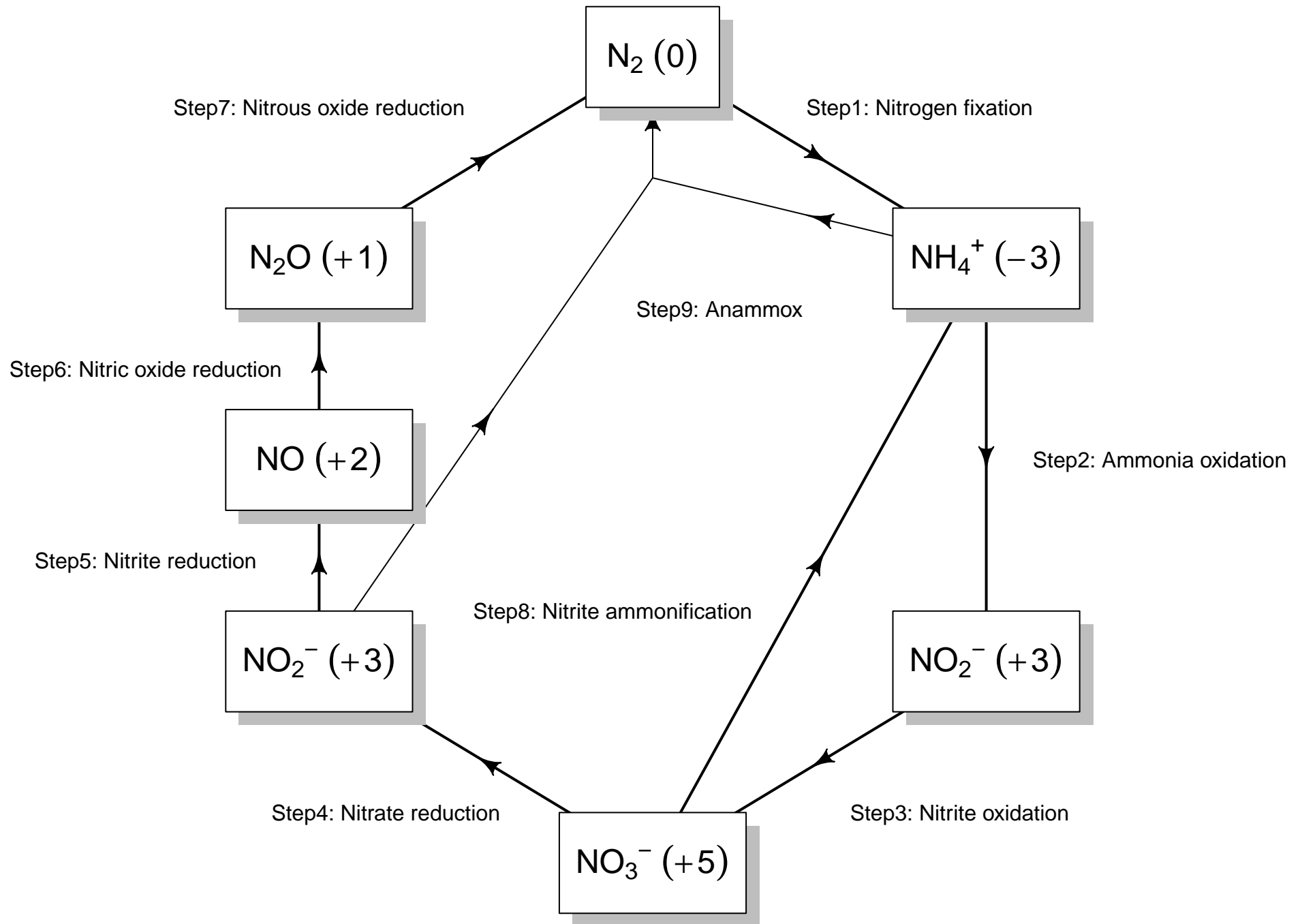

Supplement: Supplementary file 2 — Recovered nutrient cycles including Carbon, Nitrogen, and Sulphur cycles for all the metagenomic assembled genomes given as PDF images, and identifiable through bin numbers. [file 13104_2025_7441_MOESM2_ESM.zip › Supplementary Data2/bin.93.draw_nitrogen_cycle_single.pdf]

## Nitrogen Cycle: bin.80

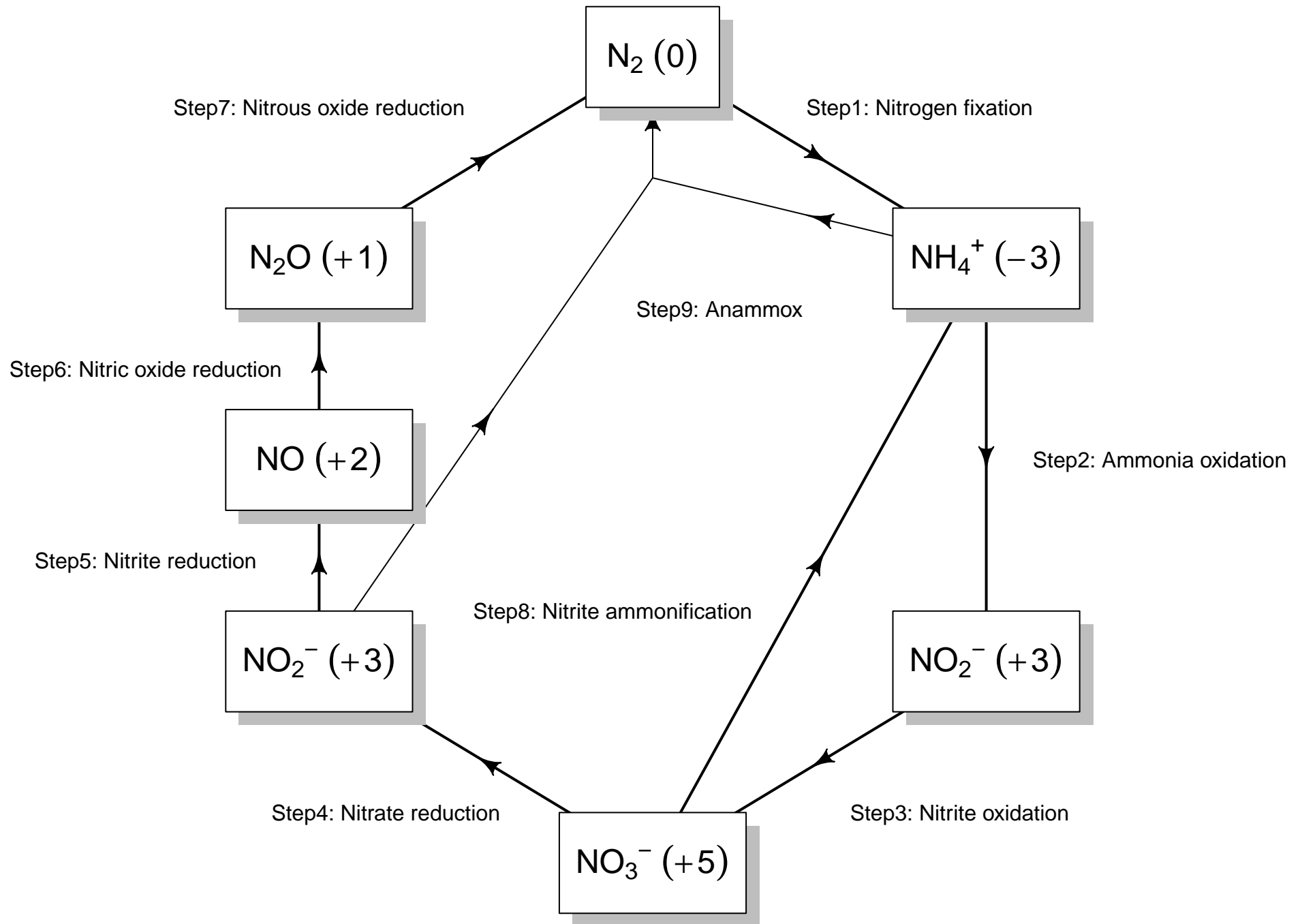

Supplement: Supplementary file 2 — Recovered nutrient cycles including Carbon, Nitrogen, and Sulphur cycles for all the metagenomic assembled genomes given as PDF images, and identifiable through bin numbers. [file 13104_2025_7441_MOESM2_ESM.zip › Supplementary Data2/bin.80.draw_nitrogen_cycle_single.pdf]

## Sulfur Cycle: bin.161

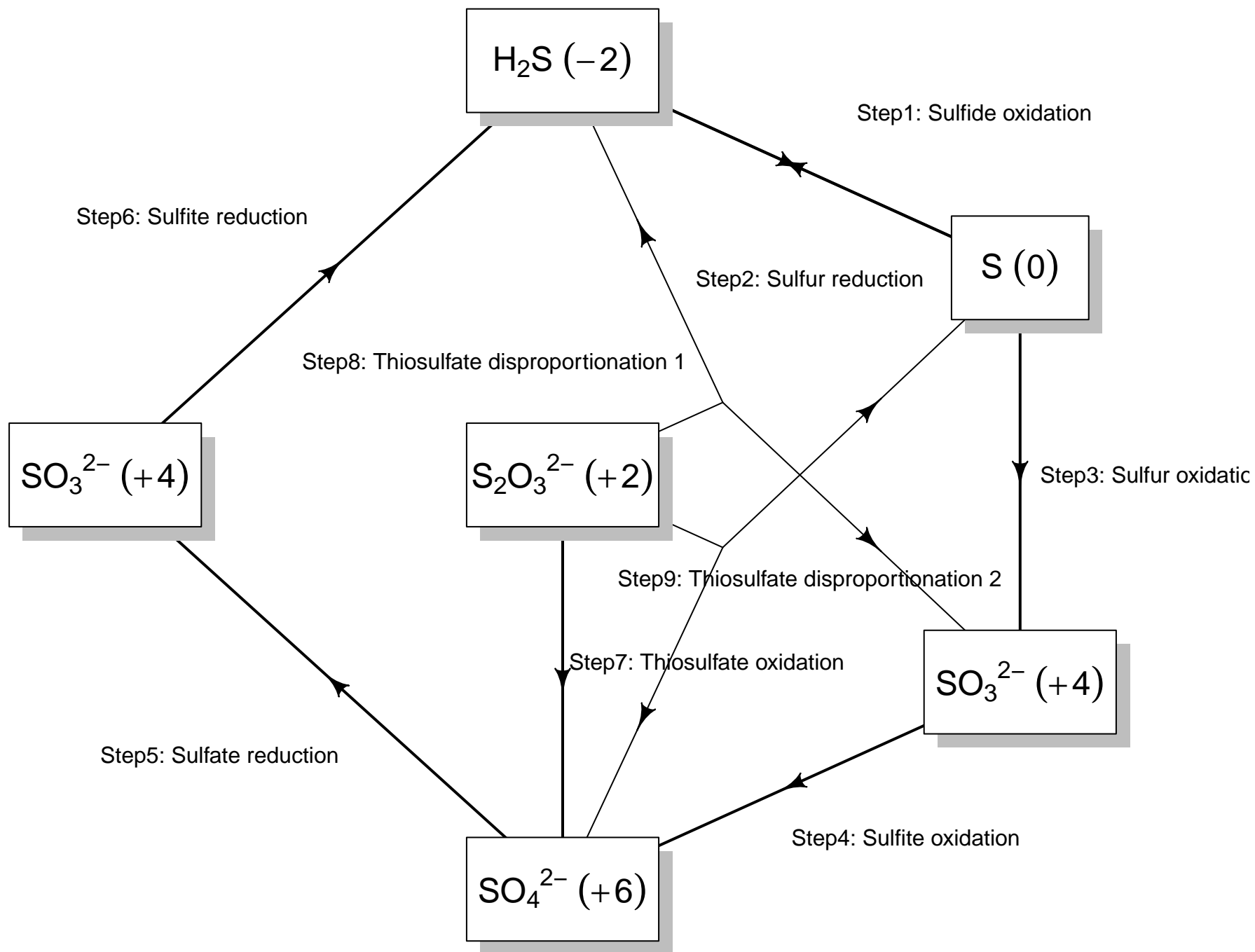

Supplement: Supplementary file 2 — Recovered nutrient cycles including Carbon, Nitrogen, and Sulphur cycles for all the metagenomic assembled genomes given as PDF images, and identifiable through bin numbers. [file 13104_2025_7441_MOESM2_ESM.zip › Supplementary Data2/bin.161.draw_sulfur_cycle_single.pdf]

# Carbon Cycle: bin.71

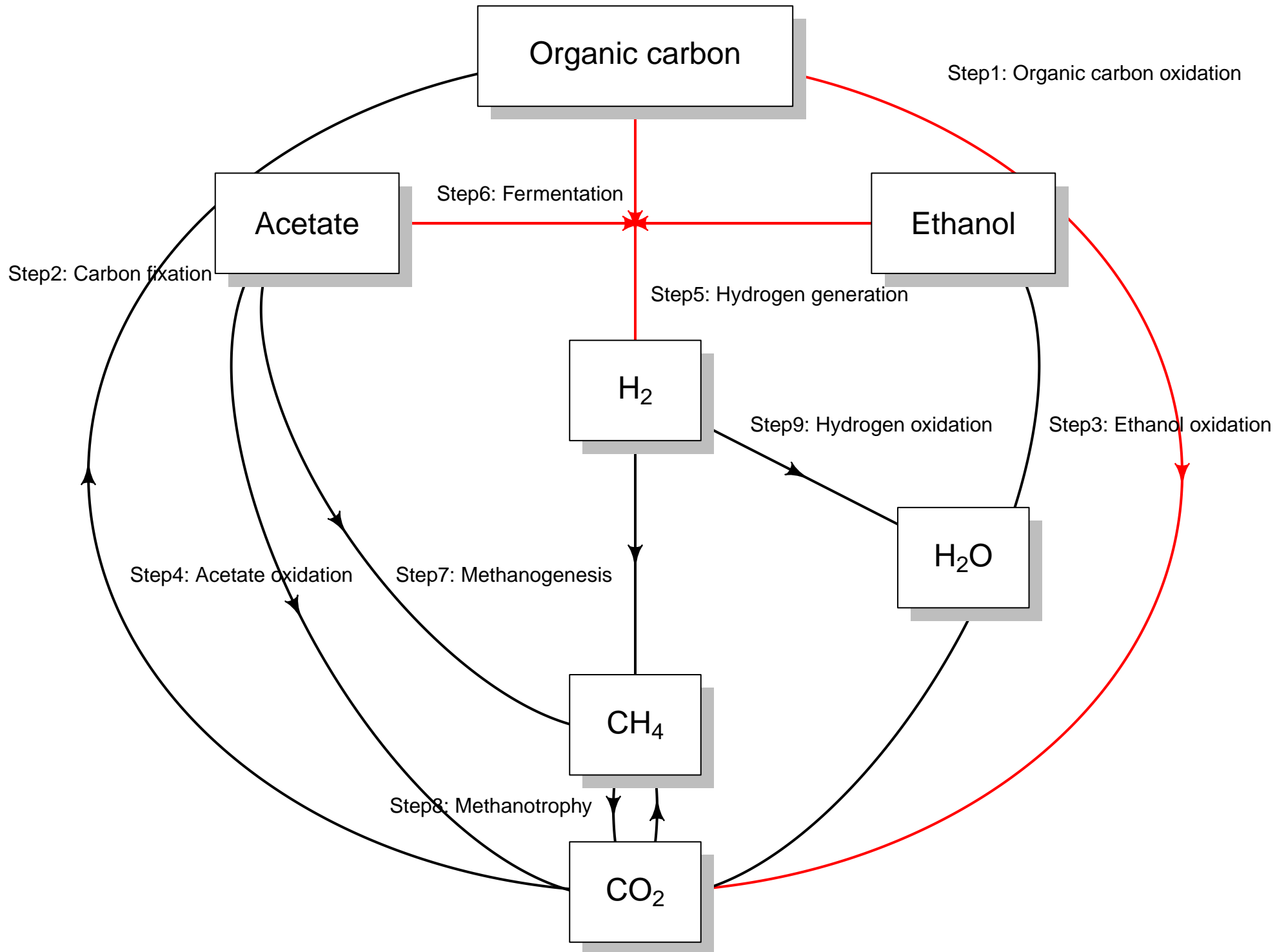

Supplement: Supplementary file 2 — Recovered nutrient cycles including Carbon, Nitrogen, and Sulphur cycles for all the metagenomic assembled genomes given as PDF images, and identifiable through bin numbers. [file 13104_2025_7441_MOESM2_ESM.zip › Supplementary Data2/bin.71.draw_carbon_cycle_single.pdf]

## Sulfur Cycle: bin.114

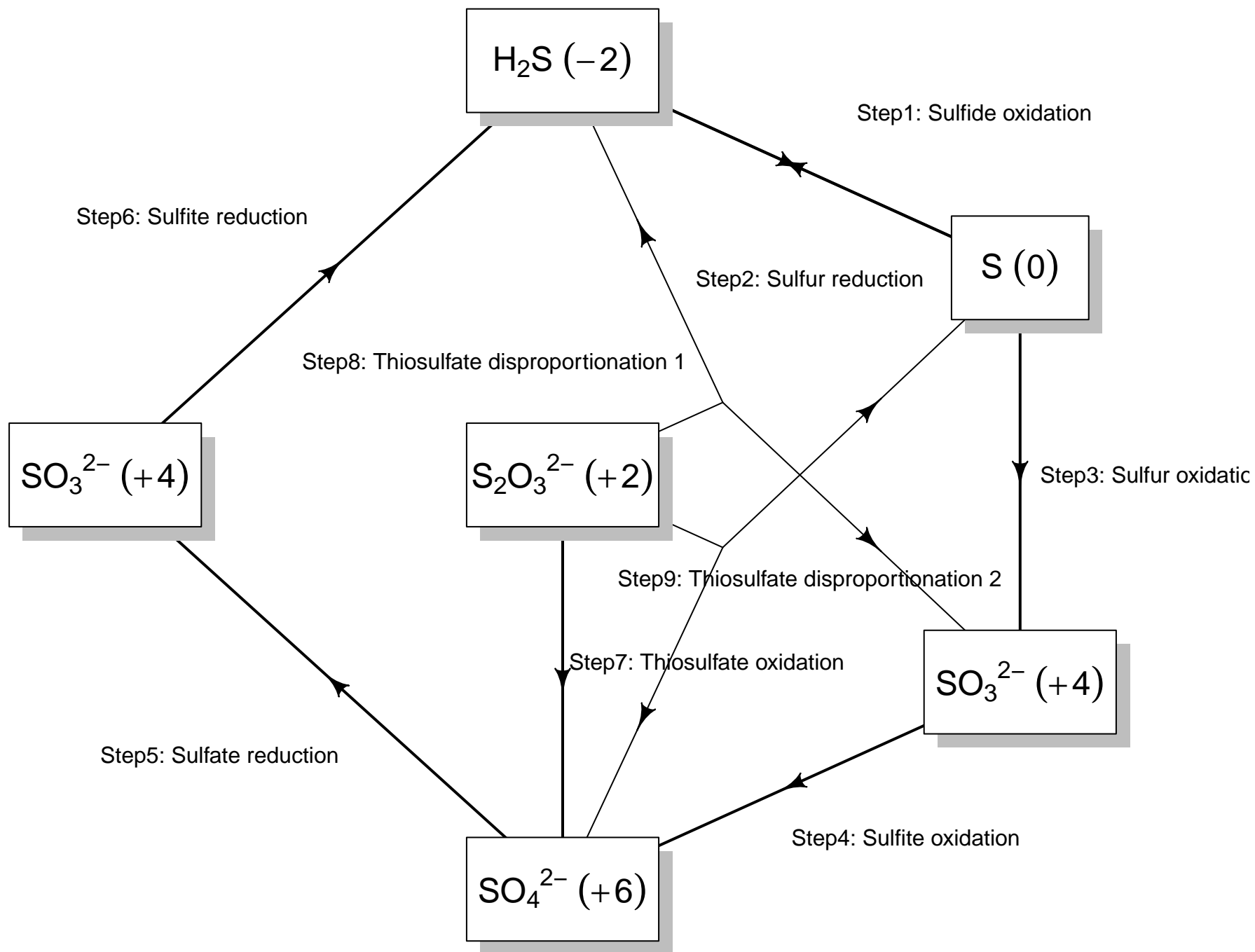

Supplement: Supplementary file 2 — Recovered nutrient cycles including Carbon, Nitrogen, and Sulphur cycles for all the metagenomic assembled genomes given as PDF images, and identifiable through bin numbers. [file 13104_2025_7441_MOESM2_ESM.zip › Supplementary Data2/bin.114.draw_sulfur_cycle_single.pdf]

# Carbon Cycle: bin.92

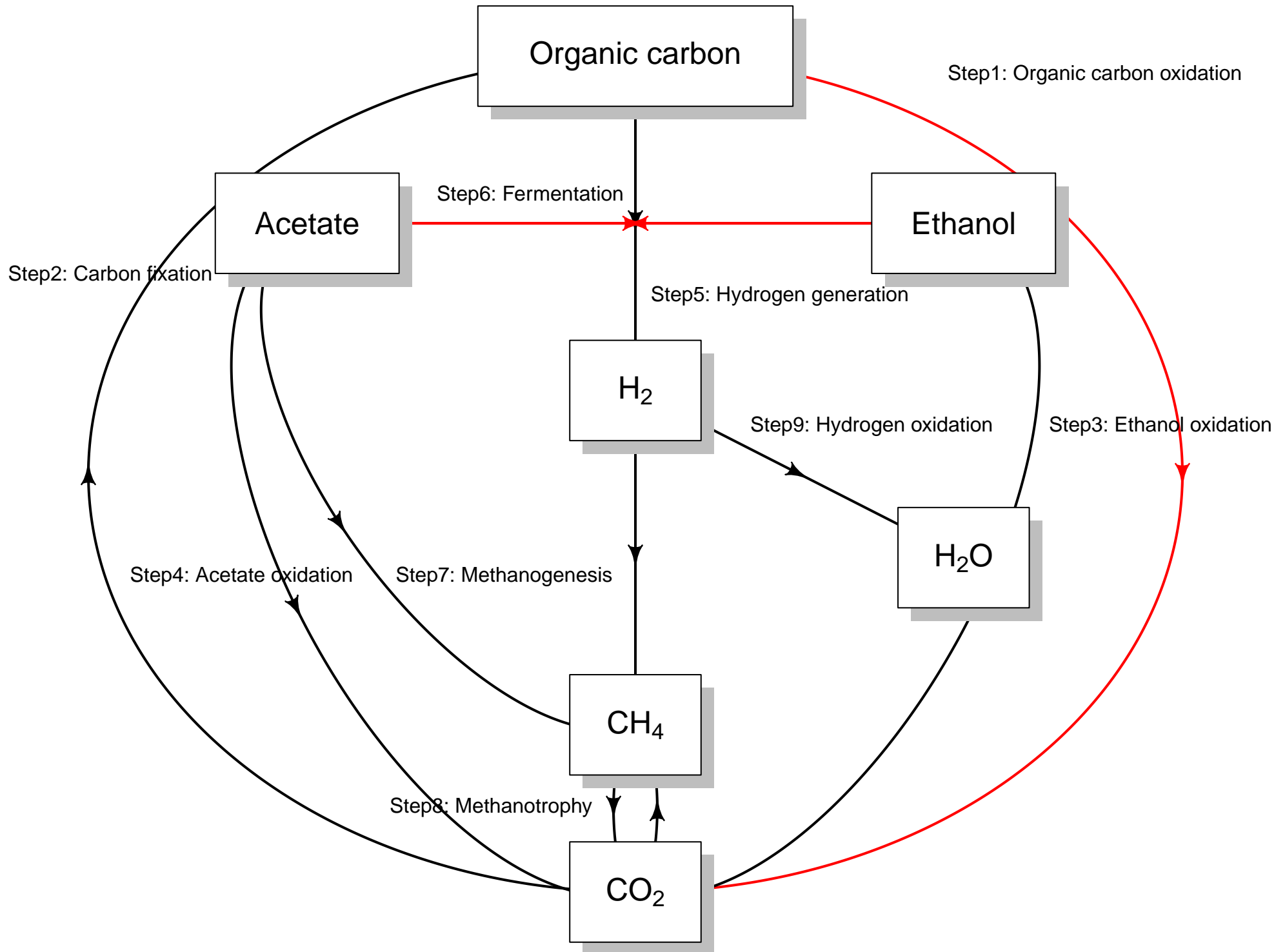

Supplement: Supplementary file 2 — Recovered nutrient cycles including Carbon, Nitrogen, and Sulphur cycles for all the metagenomic assembled genomes given as PDF images, and identifiable through bin numbers. [file 13104_2025_7441_MOESM2_ESM.zip › Supplementary Data2/bin.92.draw_carbon_cycle_single.pdf]

# Carbon Cycle: bin.137

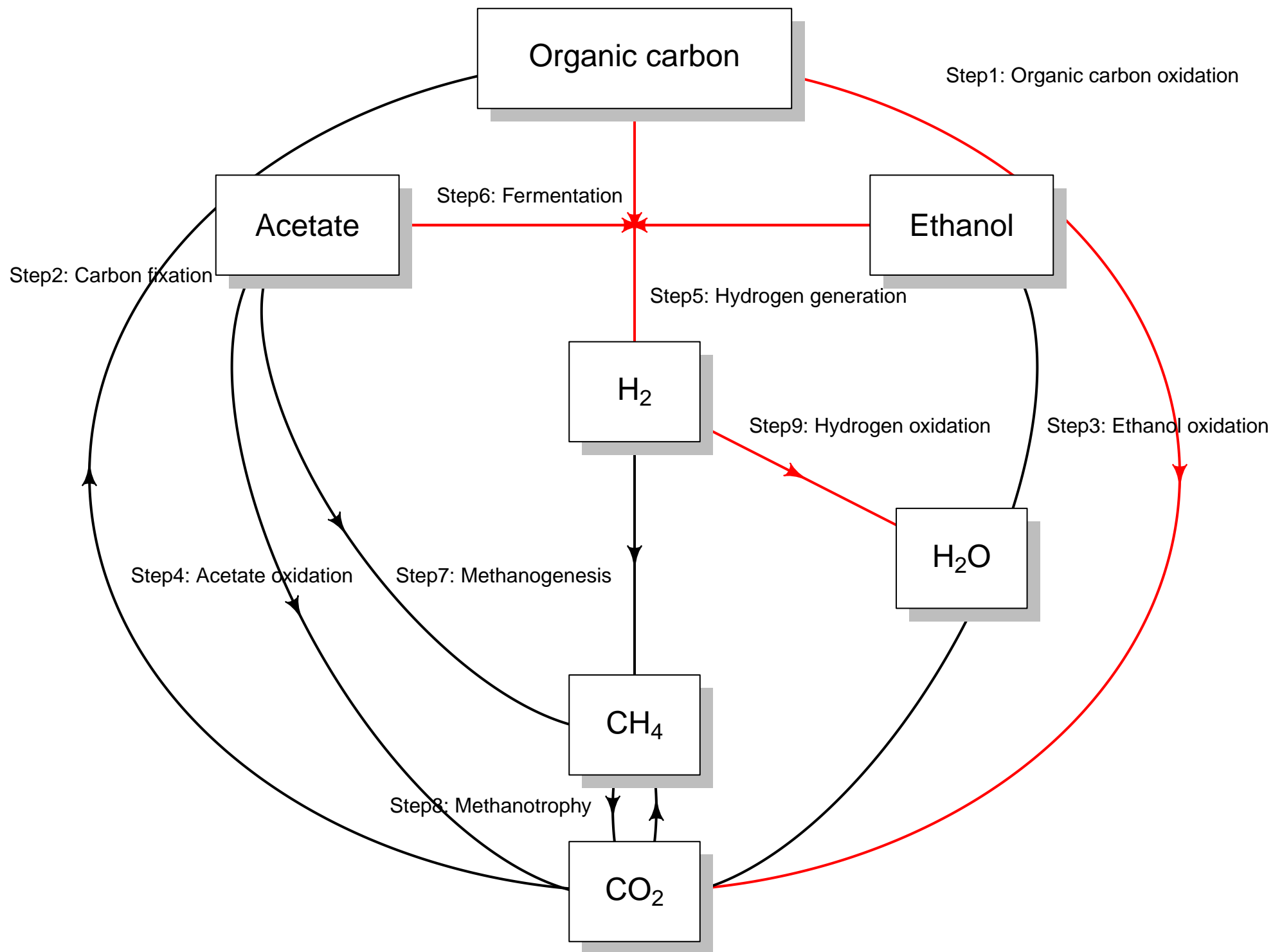

Supplement: Supplementary file 2 — Recovered nutrient cycles including Carbon, Nitrogen, and Sulphur cycles for all the metagenomic assembled genomes given as PDF images, and identifiable through bin numbers. [file 13104_2025_7441_MOESM2_ESM.zip › Supplementary Data2/bin.137.draw_carbon_cycle_single.pdf]

# Carbon Cycle: bin.4

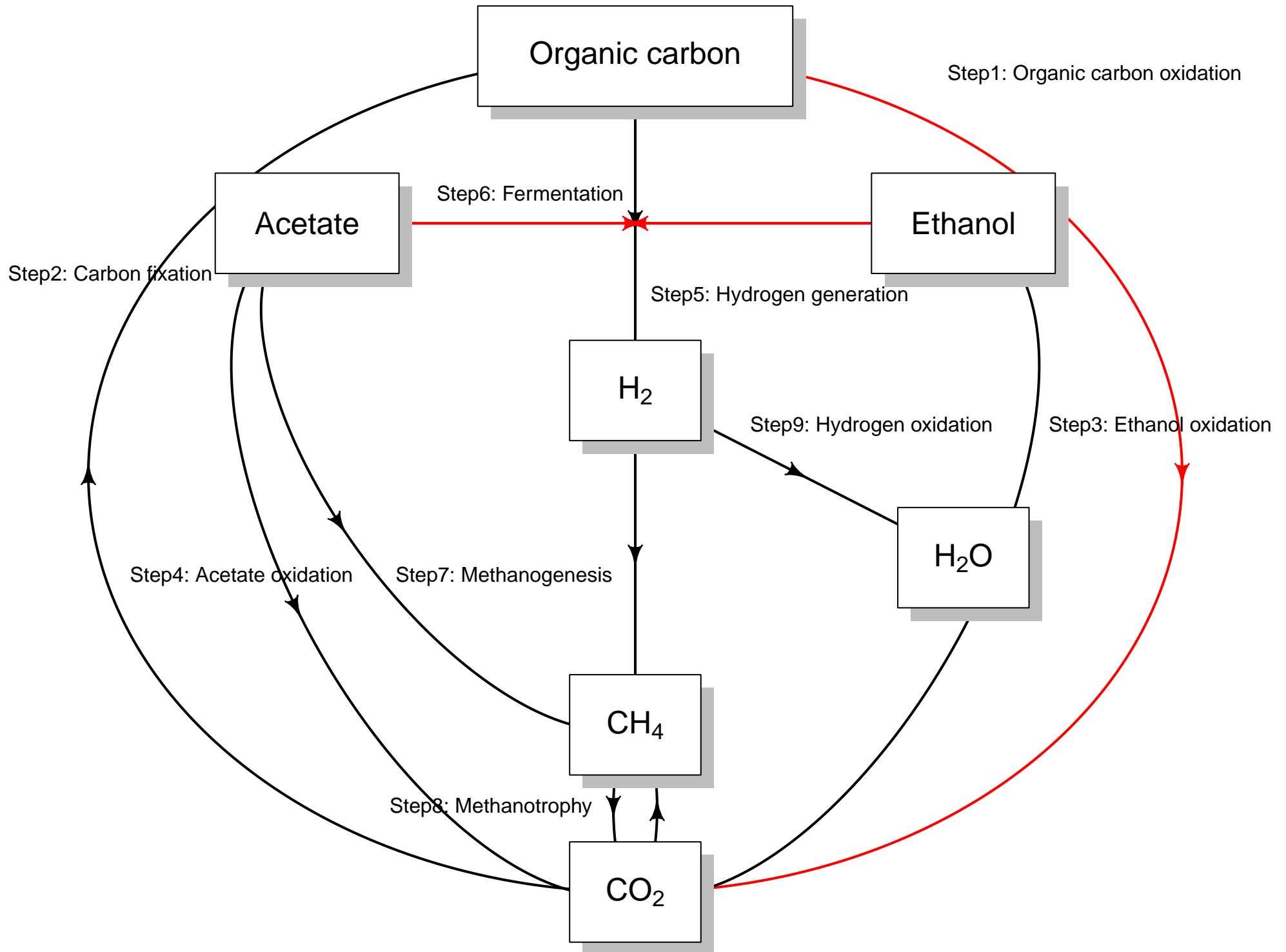

Supplement: Supplementary file 2 — Recovered nutrient cycles including Carbon, Nitrogen, and Sulphur cycles for all the metagenomic assembled genomes given as PDF images, and identifiable through bin numbers. [file 13104_2025_7441_MOESM2_ESM.zip › Supplementary Data2/bin.4.draw_carbon_cycle_single.pdf]

## Sulfur Cycle: bin.27

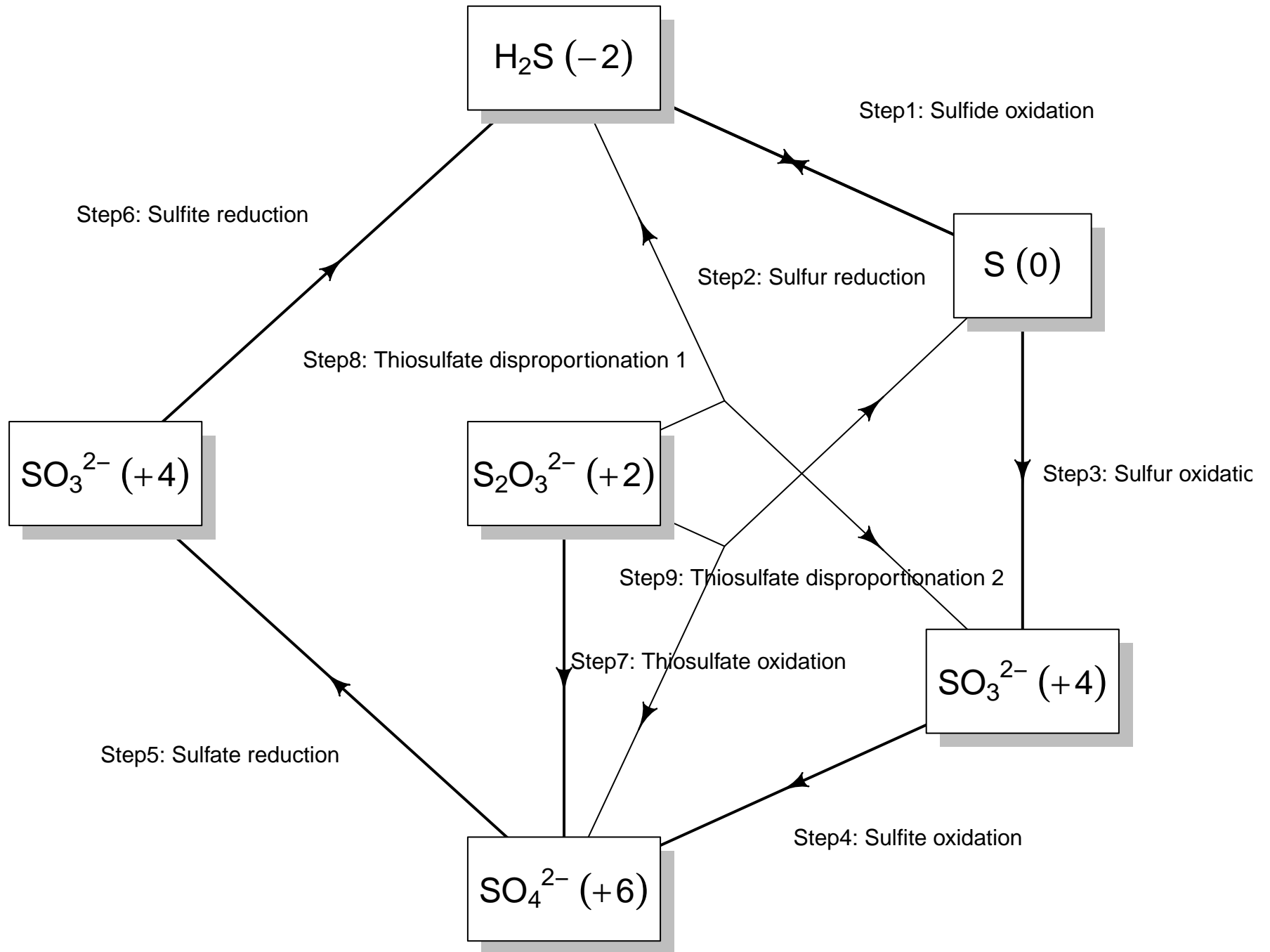

Supplement: Supplementary file 2 — Recovered nutrient cycles including Carbon, Nitrogen, and Sulphur cycles for all the metagenomic assembled genomes given as PDF images, and identifiable through bin numbers. [file 13104_2025_7441_MOESM2_ESM.zip › Supplementary Data2/bin.27.draw_sulfur_cycle_single.pdf]

# Carbon Cycle: bin.139

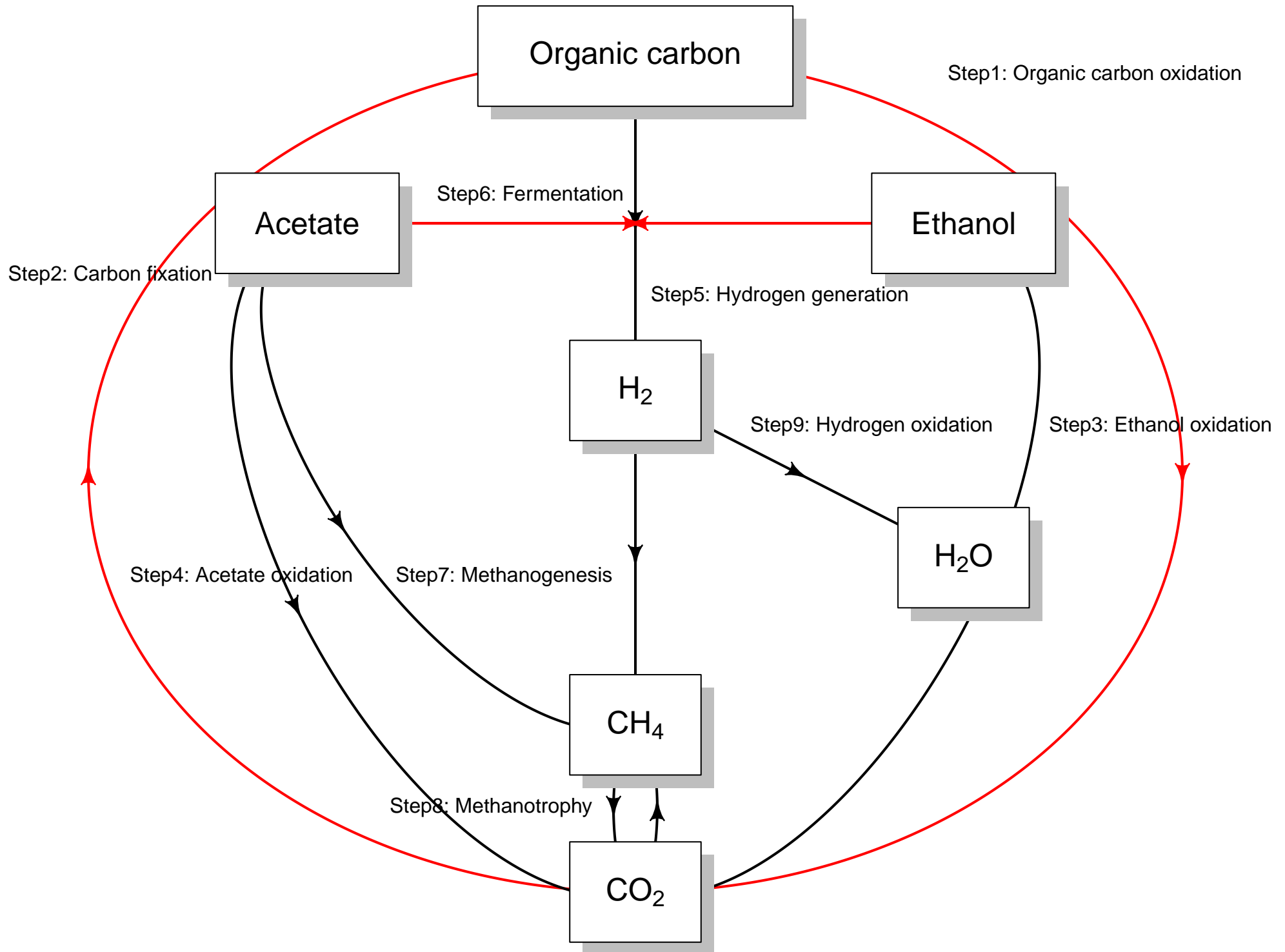

Supplement: Supplementary file 2 — Recovered nutrient cycles including Carbon, Nitrogen, and Sulphur cycles for all the metagenomic assembled genomes given as PDF images, and identifiable through bin numbers. [file 13104_2025_7441_MOESM2_ESM.zip › Supplementary Data2/bin.139.draw_carbon_cycle_single.pdf]

## Sulfur Cycle: bin.29

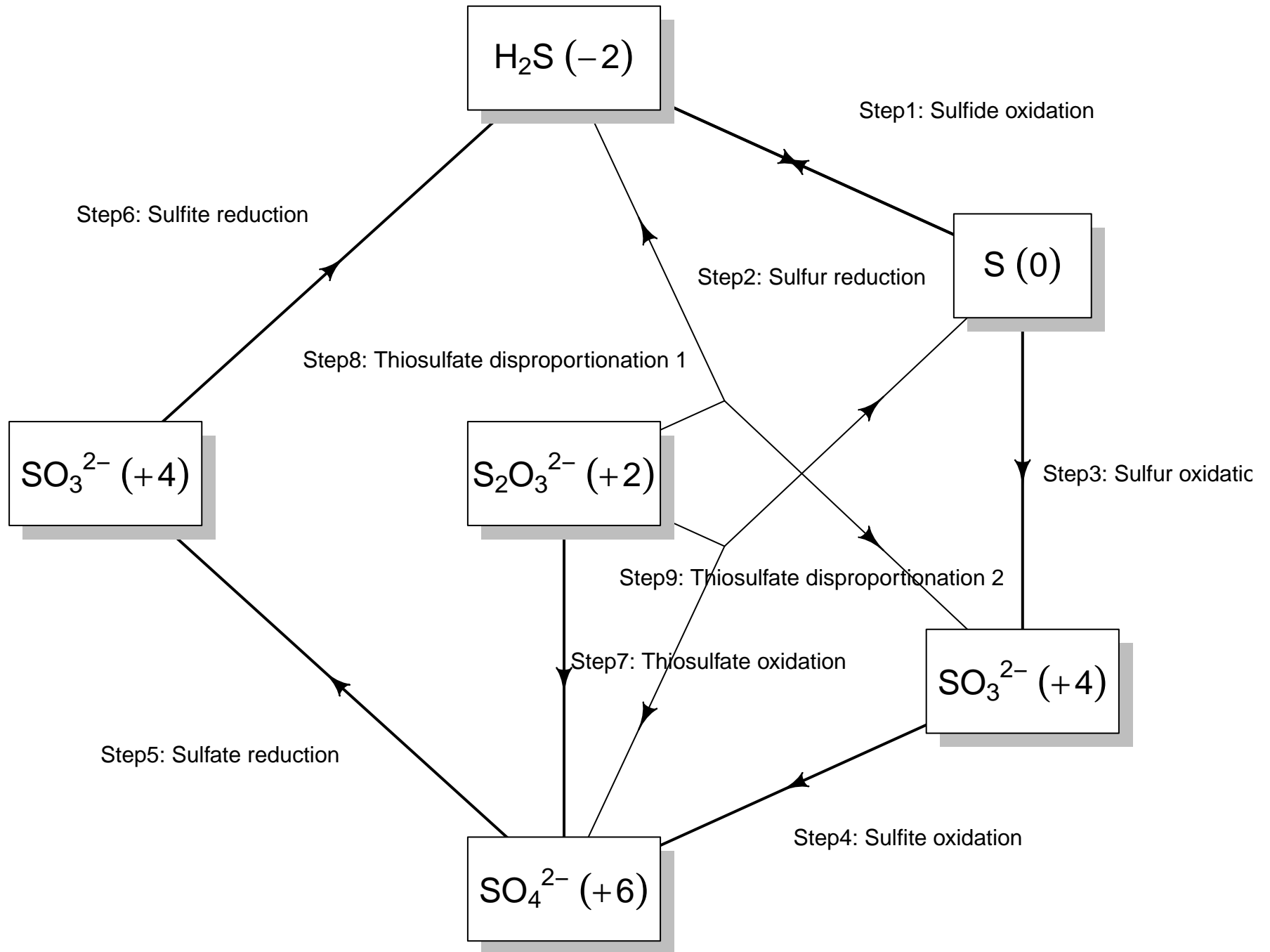

Supplement: Supplementary file 2 — Recovered nutrient cycles including Carbon, Nitrogen, and Sulphur cycles for all the metagenomic assembled genomes given as PDF images, and identifiable through bin numbers. [file 13104_2025_7441_MOESM2_ESM.zip › Supplementary Data2/bin.29.draw_sulfur_cycle_single.pdf]

## Nitrogen Cycle: bin.115

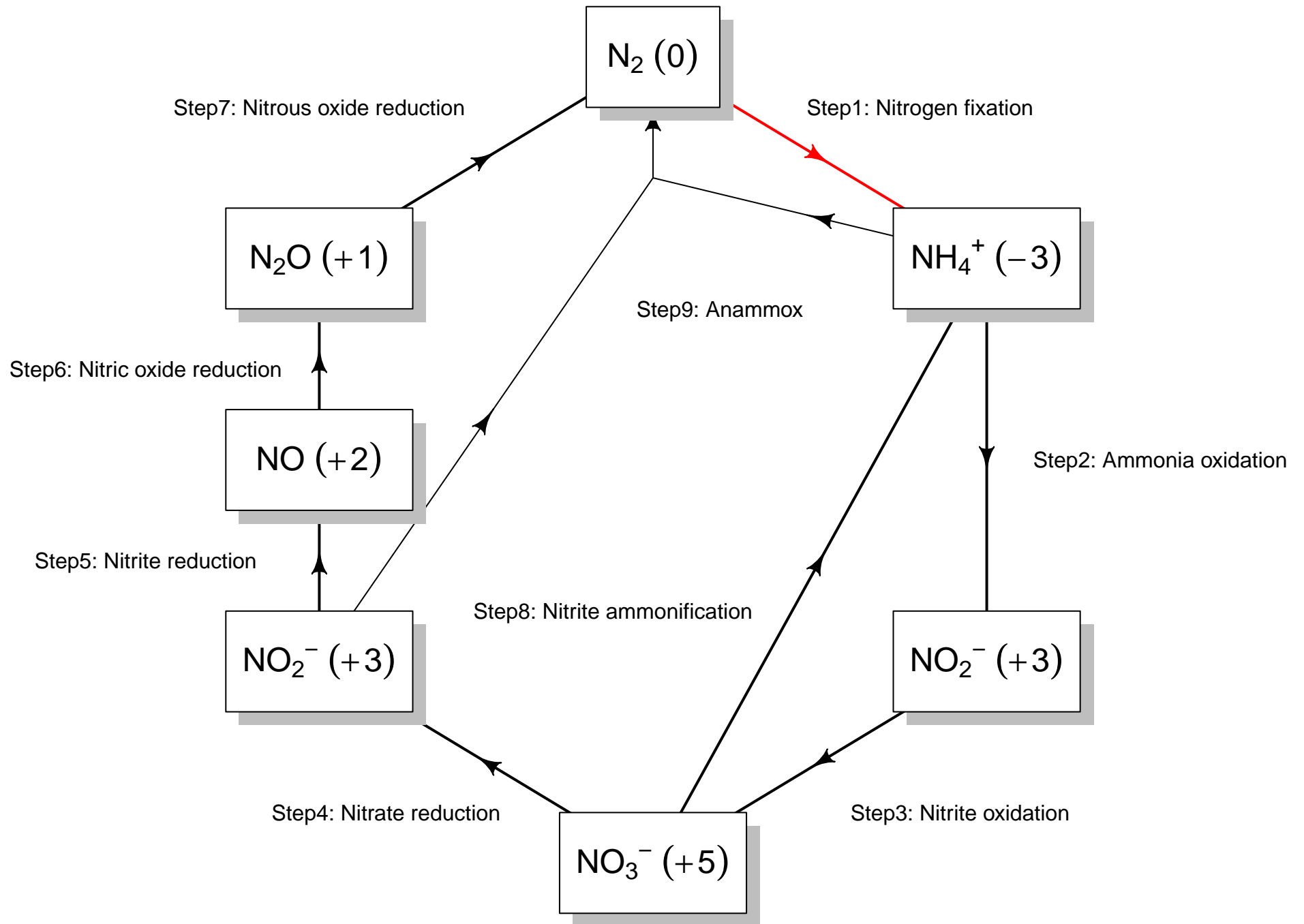

Supplement: Supplementary file 2 — Recovered nutrient cycles including Carbon, Nitrogen, and Sulphur cycles for all the metagenomic assembled genomes given as PDF images, and identifiable through bin numbers. [file 13104_2025_7441_MOESM2_ESM.zip › Supplementary Data2/bin.115.draw_nitrogen_cycle_single.pdf]

## Sulfur Cycle: bin.32

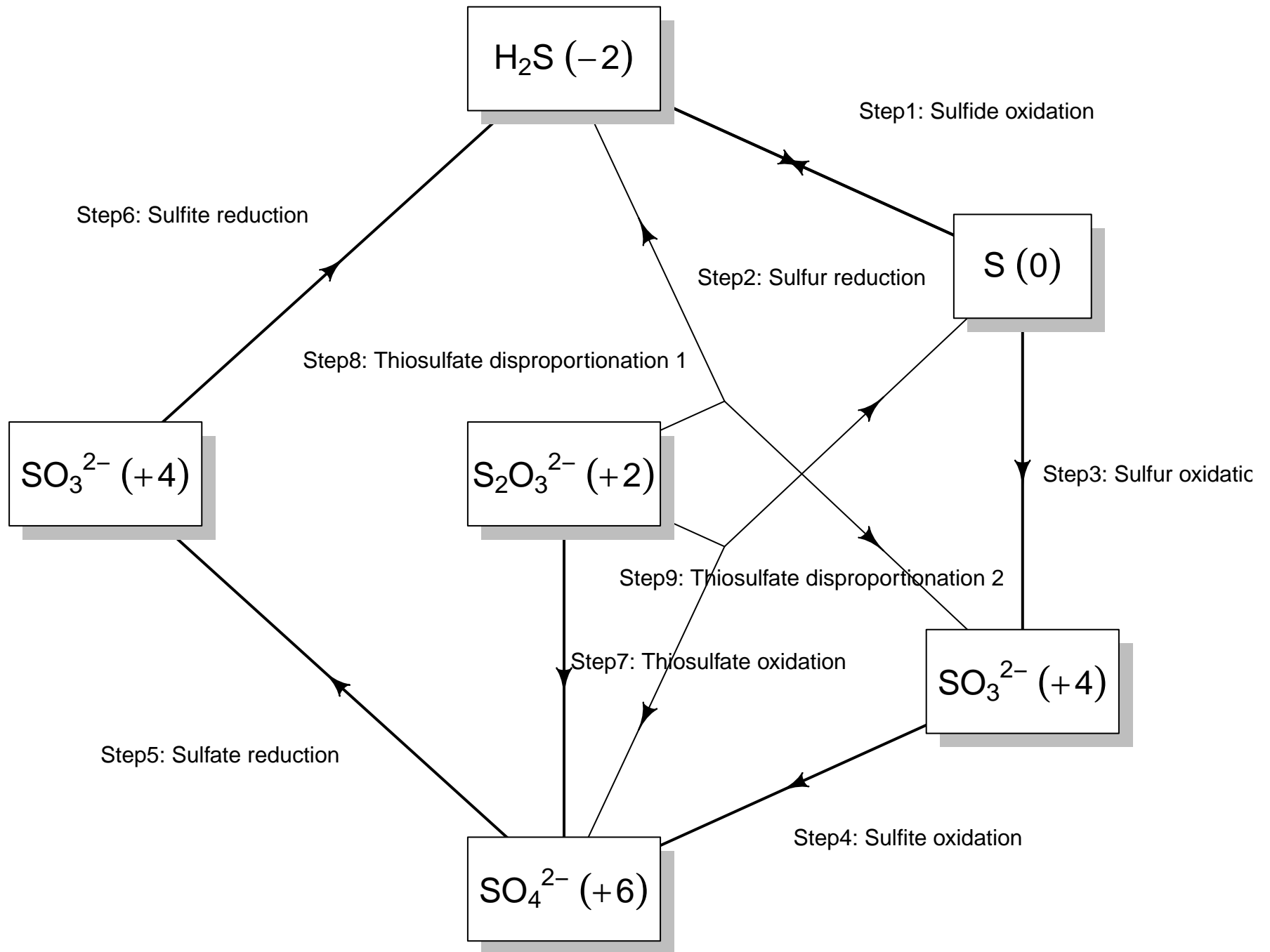

Supplement: Supplementary file 2 — Recovered nutrient cycles including Carbon, Nitrogen, and Sulphur cycles for all the metagenomic assembled genomes given as PDF images, and identifiable through bin numbers. [file 13104_2025_7441_MOESM2_ESM.zip › Supplementary Data2/bin.32.draw_sulfur_cycle_single.pdf]

## Nitrogen Cycle: bin.51

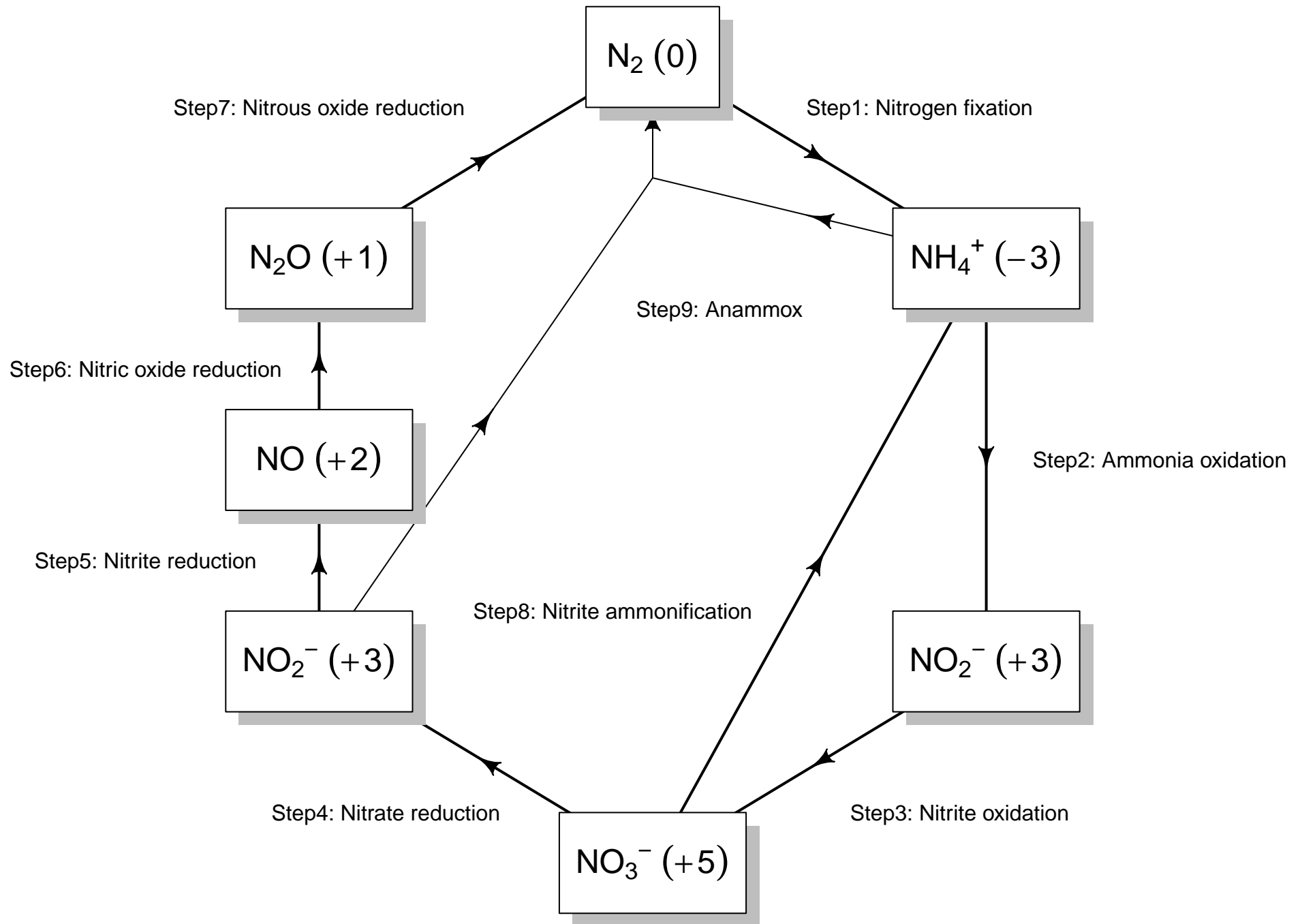

Supplement: Supplementary file 2 — Recovered nutrient cycles including Carbon, Nitrogen, and Sulphur cycles for all the metagenomic assembled genomes given as PDF images, and identifiable through bin numbers. [file 13104_2025_7441_MOESM2_ESM.zip › Supplementary Data2/bin.51.draw_nitrogen_cycle_single.pdf]

# Carbon Cycle: bin.122

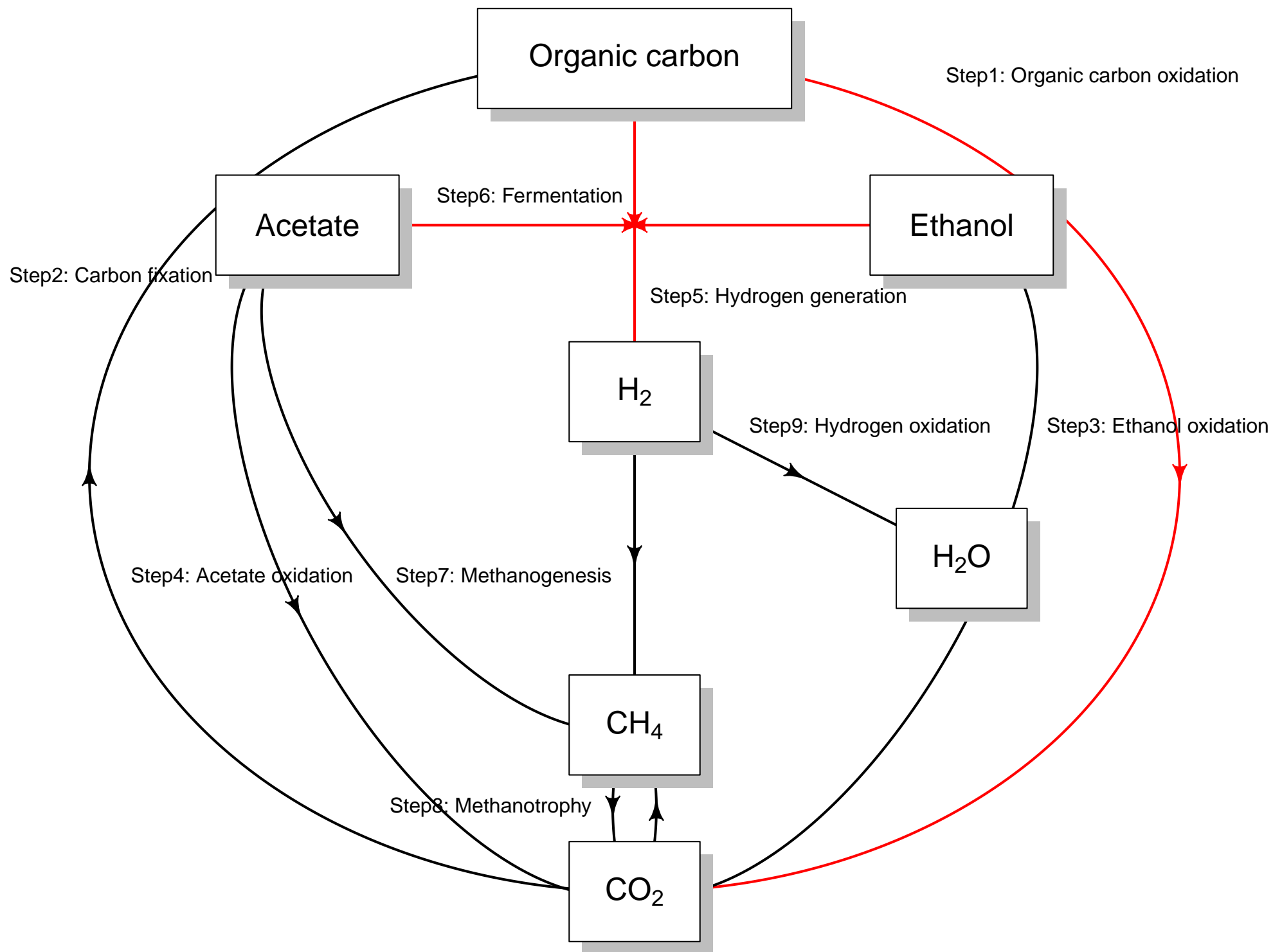

Supplement: Supplementary file 2 — Recovered nutrient cycles including Carbon, Nitrogen, and Sulphur cycles for all the metagenomic assembled genomes given as PDF images, and identifiable through bin numbers. [file 13104_2025_7441_MOESM2_ESM.zip › Supplementary Data2/bin.122.draw_carbon_cycle_single.pdf]

## Nitrogen Cycle: bin.149

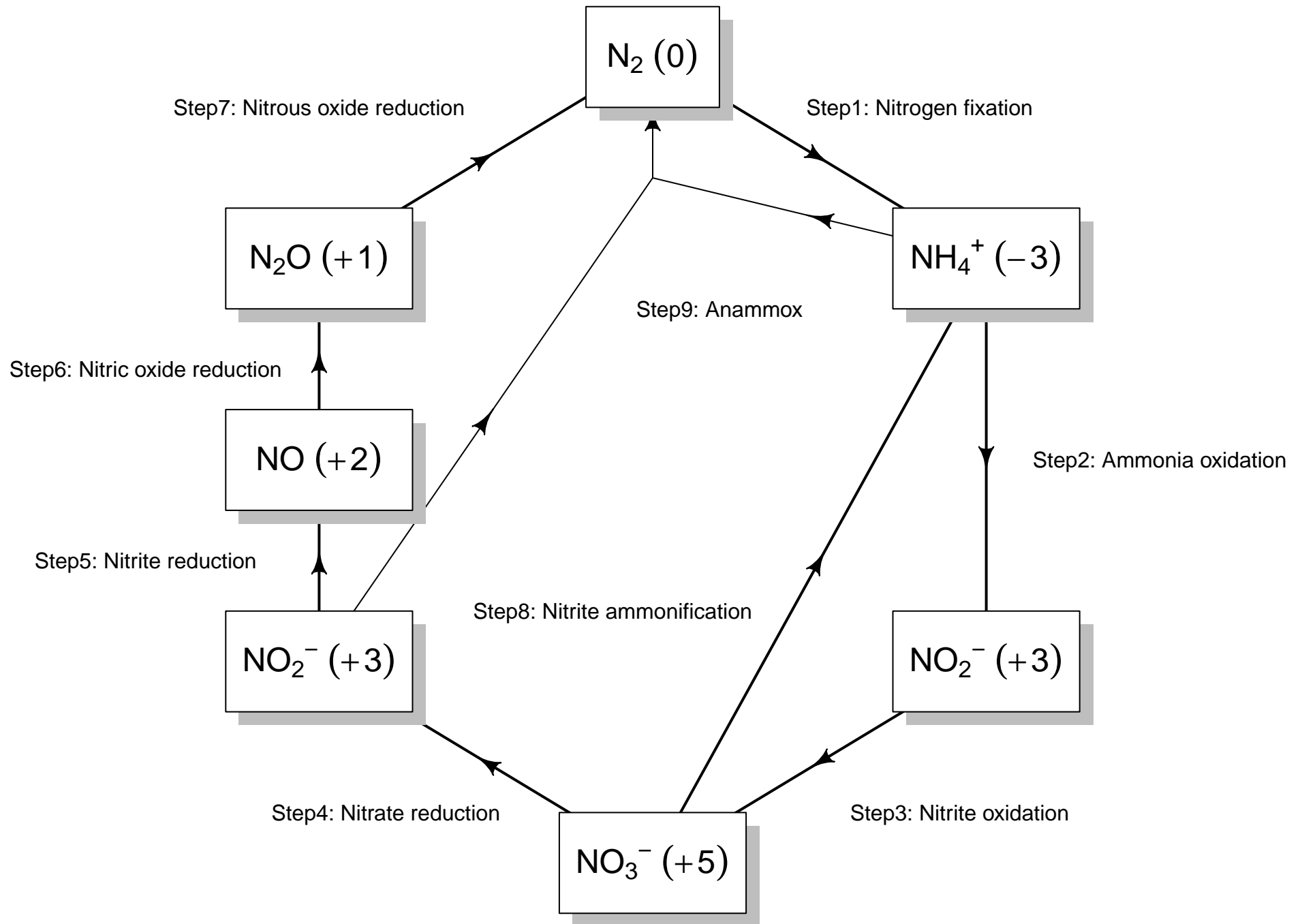

Supplement: Supplementary file 2 — Recovered nutrient cycles including Carbon, Nitrogen, and Sulphur cycles for all the metagenomic assembled genomes given as PDF images, and identifiable through bin numbers. [file 13104_2025_7441_MOESM2_ESM.zip › Supplementary Data2/bin.149.draw_nitrogen_cycle_single.pdf]

## Nitrogen Cycle: bin.42

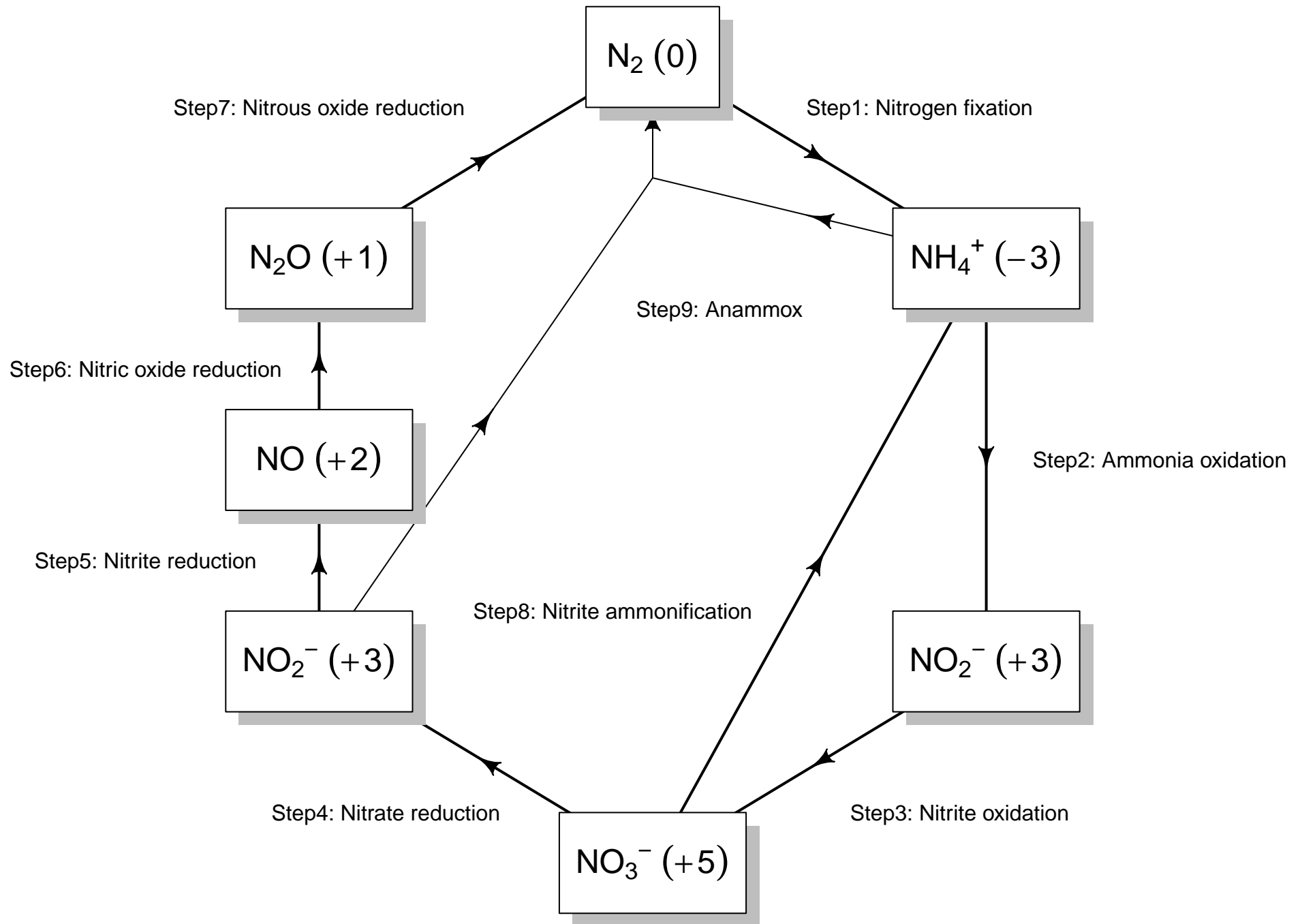

Supplement: Supplementary file 2 — Recovered nutrient cycles including Carbon, Nitrogen, and Sulphur cycles for all the metagenomic assembled genomes given as PDF images, and identifiable through bin numbers. [file 13104_2025_7441_MOESM2_ESM.zip › Supplementary Data2/bin.42.draw_nitrogen_cycle_single.pdf]

# Carbon Cycle: bin.89

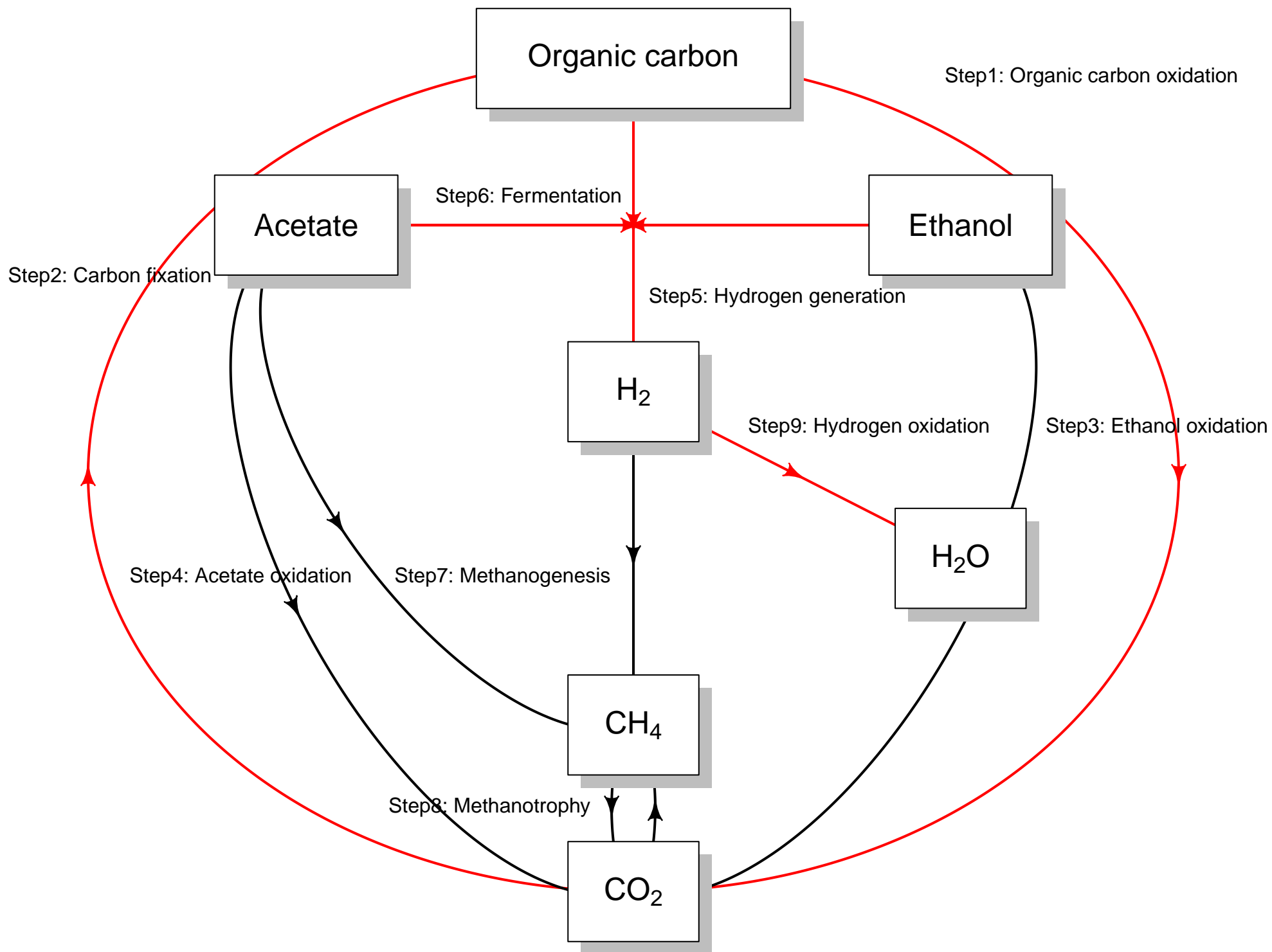

Supplement: Supplementary file 2 — Recovered nutrient cycles including Carbon, Nitrogen, and Sulphur cycles for all the metagenomic assembled genomes given as PDF images, and identifiable through bin numbers. [file 13104_2025_7441_MOESM2_ESM.zip › Supplementary Data2/bin.89.draw_carbon_cycle_single.pdf]

## Sulfur Cycle: bin.101

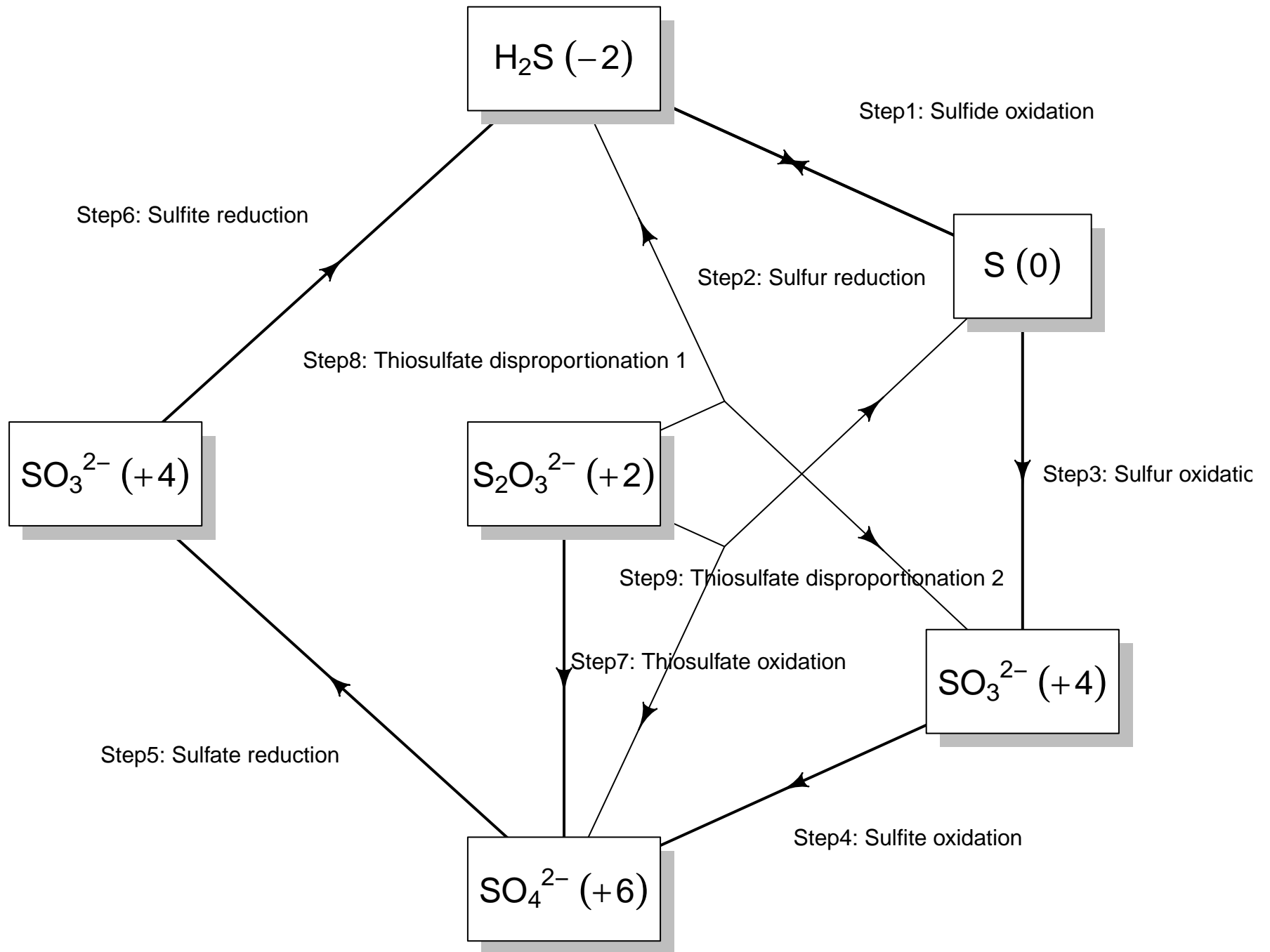

Supplement: Supplementary file 2 — Recovered nutrient cycles including Carbon, Nitrogen, and Sulphur cycles for all the metagenomic assembled genomes given as PDF images, and identifiable through bin numbers. [file 13104_2025_7441_MOESM2_ESM.zip › Supplementary Data2/bin.101.draw_sulfur_cycle_single.pdf]

## Nitrogen Cycle: bin.106

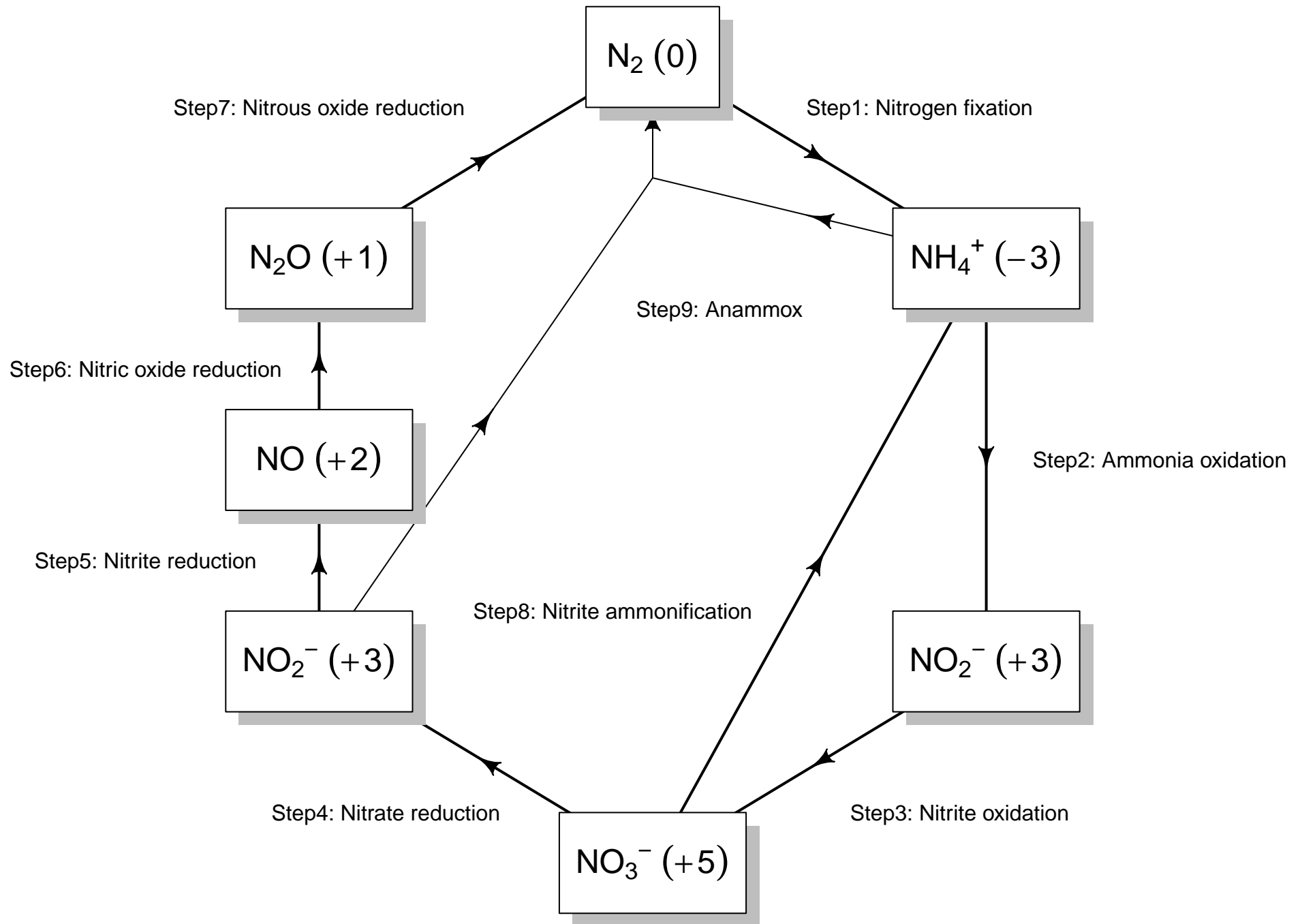

Supplement: Supplementary file 2 — Recovered nutrient cycles including Carbon, Nitrogen, and Sulphur cycles for all the metagenomic assembled genomes given as PDF images, and identifiable through bin numbers. [file 13104_2025_7441_MOESM2_ESM.zip › Supplementary Data2/bin.106.draw_nitrogen_cycle_single.pdf]

# Carbon Cycle: bin.11

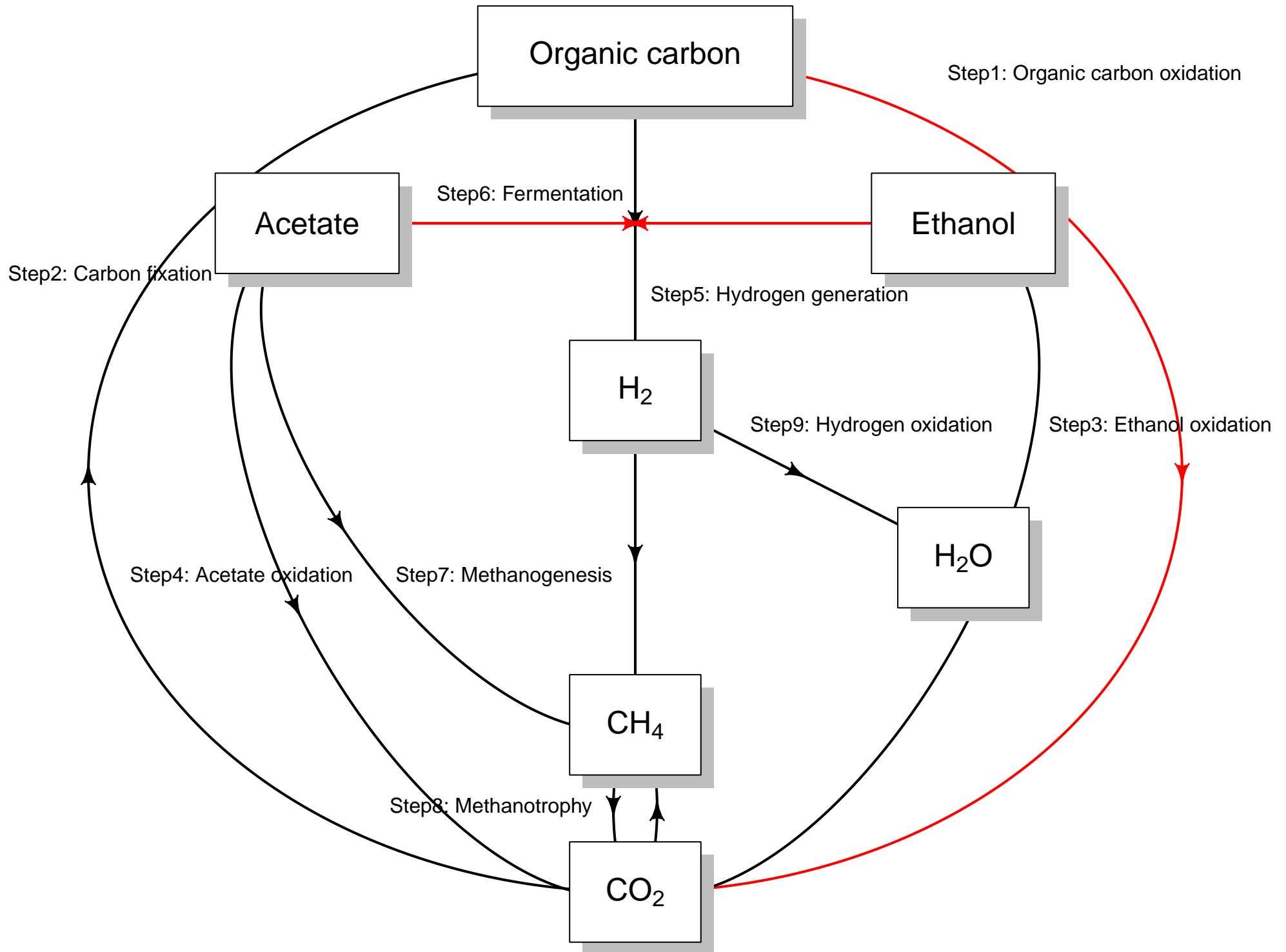

Supplement: Supplementary file 2 — Recovered nutrient cycles including Carbon, Nitrogen, and Sulphur cycles for all the metagenomic assembled genomes given as PDF images, and identifiable through bin numbers. [file 13104_2025_7441_MOESM2_ESM.zip › Supplementary Data2/bin.11.draw_carbon_cycle_single.pdf]

# Carbon Cycle: bin.35

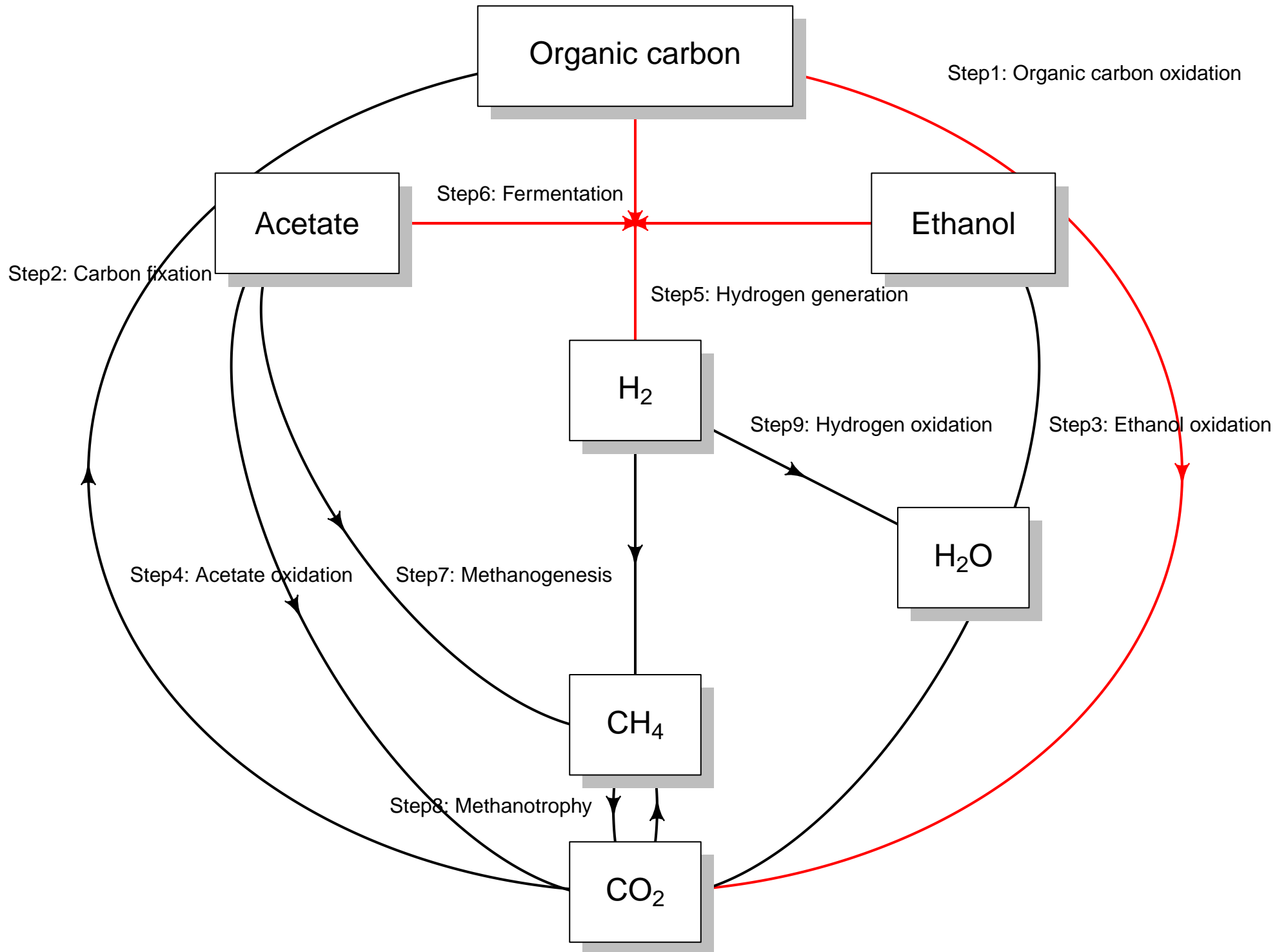

Supplement: Supplementary file 2 — Recovered nutrient cycles including Carbon, Nitrogen, and Sulphur cycles for all the metagenomic assembled genomes given as PDF images, and identifiable through bin numbers. [file 13104_2025_7441_MOESM2_ESM.zip › Supplementary Data2/bin.35.draw_carbon_cycle_single.pdf]

## Sulfur Cycle: bin.125

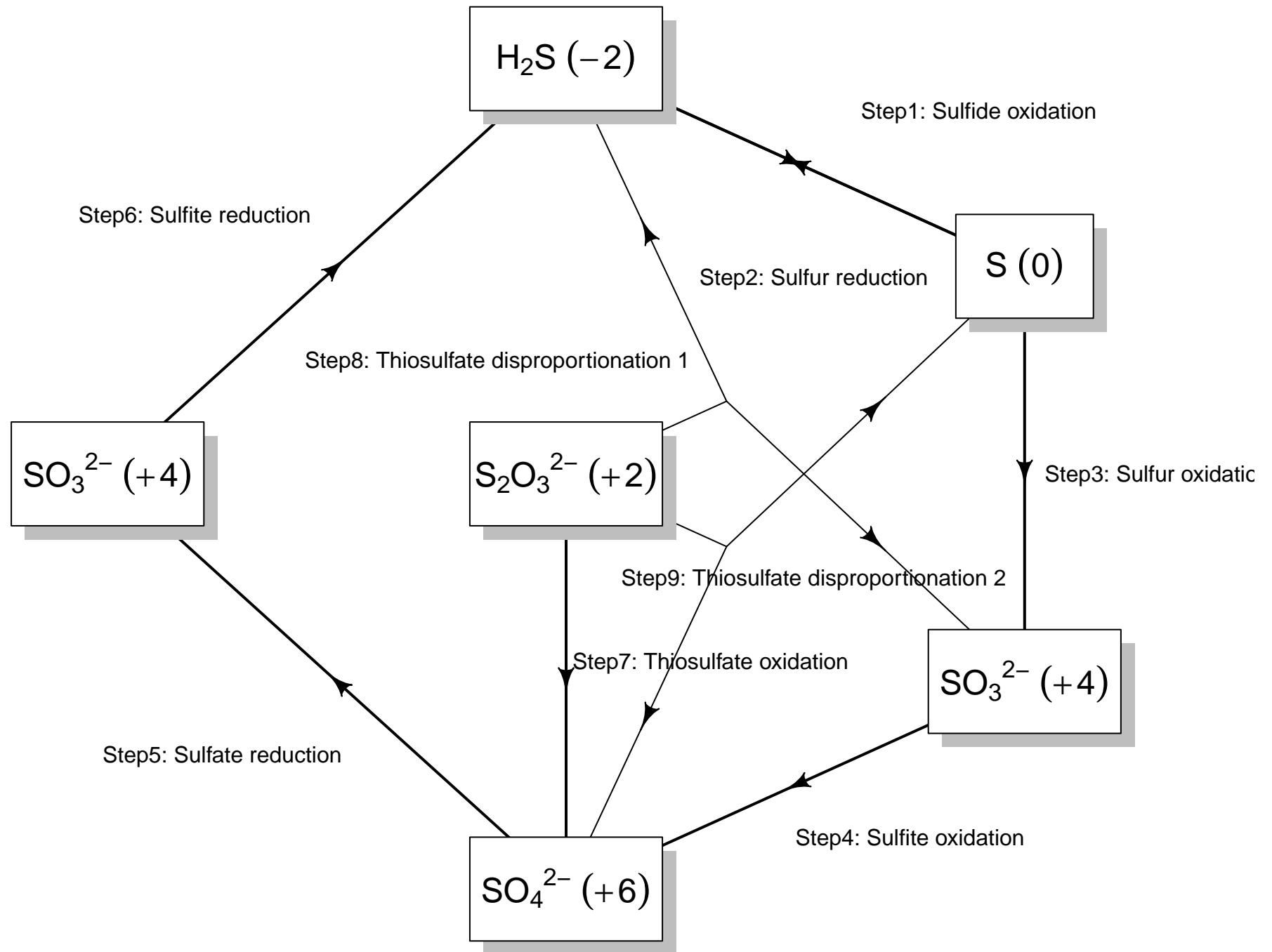

Supplement: Supplementary file 2 — Recovered nutrient cycles including Carbon, Nitrogen, and Sulphur cycles for all the metagenomic assembled genomes given as PDF images, and identifiable through bin numbers. [file 13104_2025_7441_MOESM2_ESM.zip › Supplementary Data2/bin.125.draw_sulfur_cycle_single.pdf]

# Carbon Cycle: bin.108

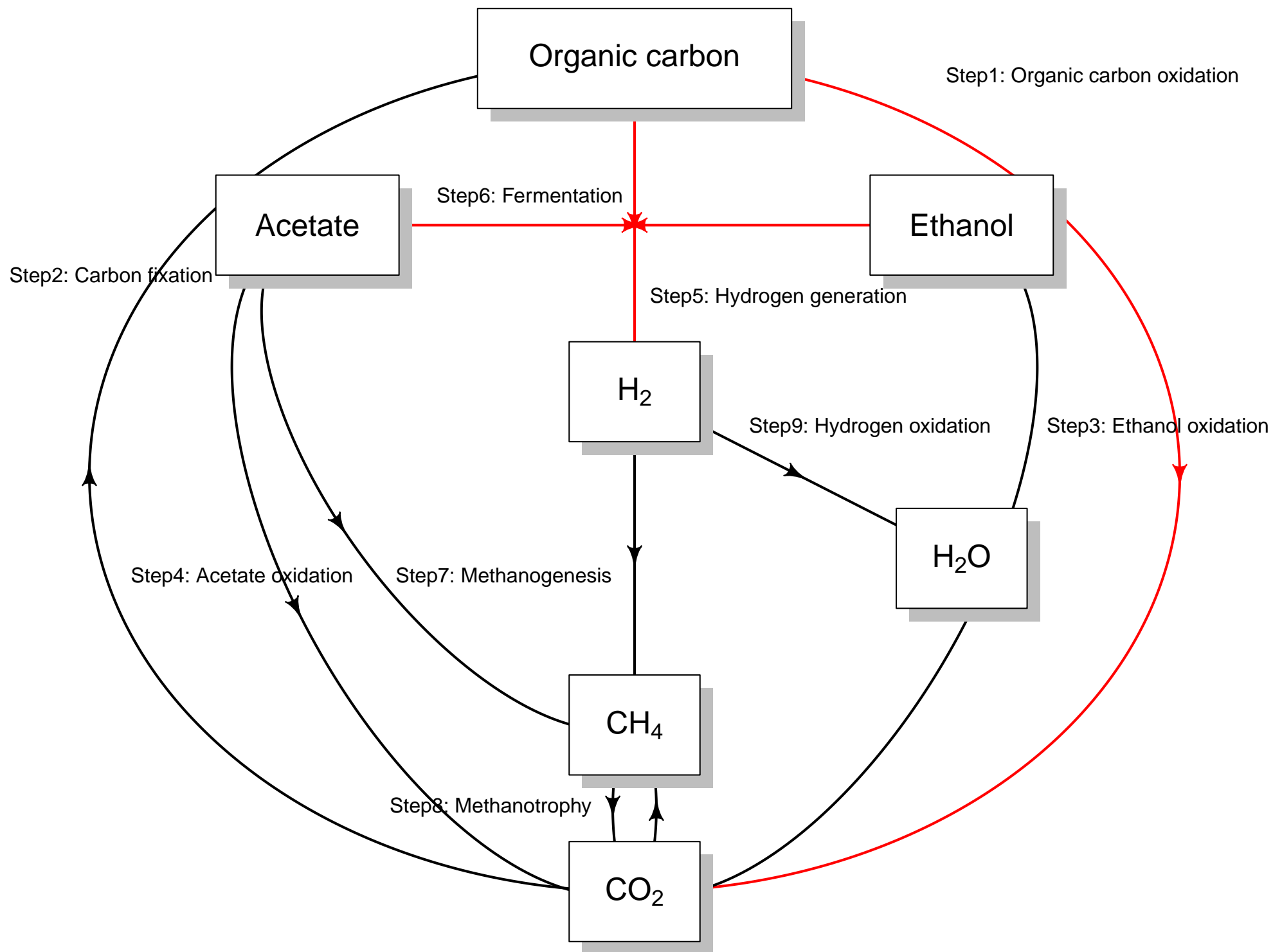

Supplement: Supplementary file 2 — Recovered nutrient cycles including Carbon, Nitrogen, and Sulphur cycles for all the metagenomic assembled genomes given as PDF images, and identifiable through bin numbers. [file 13104_2025_7441_MOESM2_ESM.zip › Supplementary Data2/bin.108.draw_carbon_cycle_single.pdf]

## Sulfur Cycle: bin.80

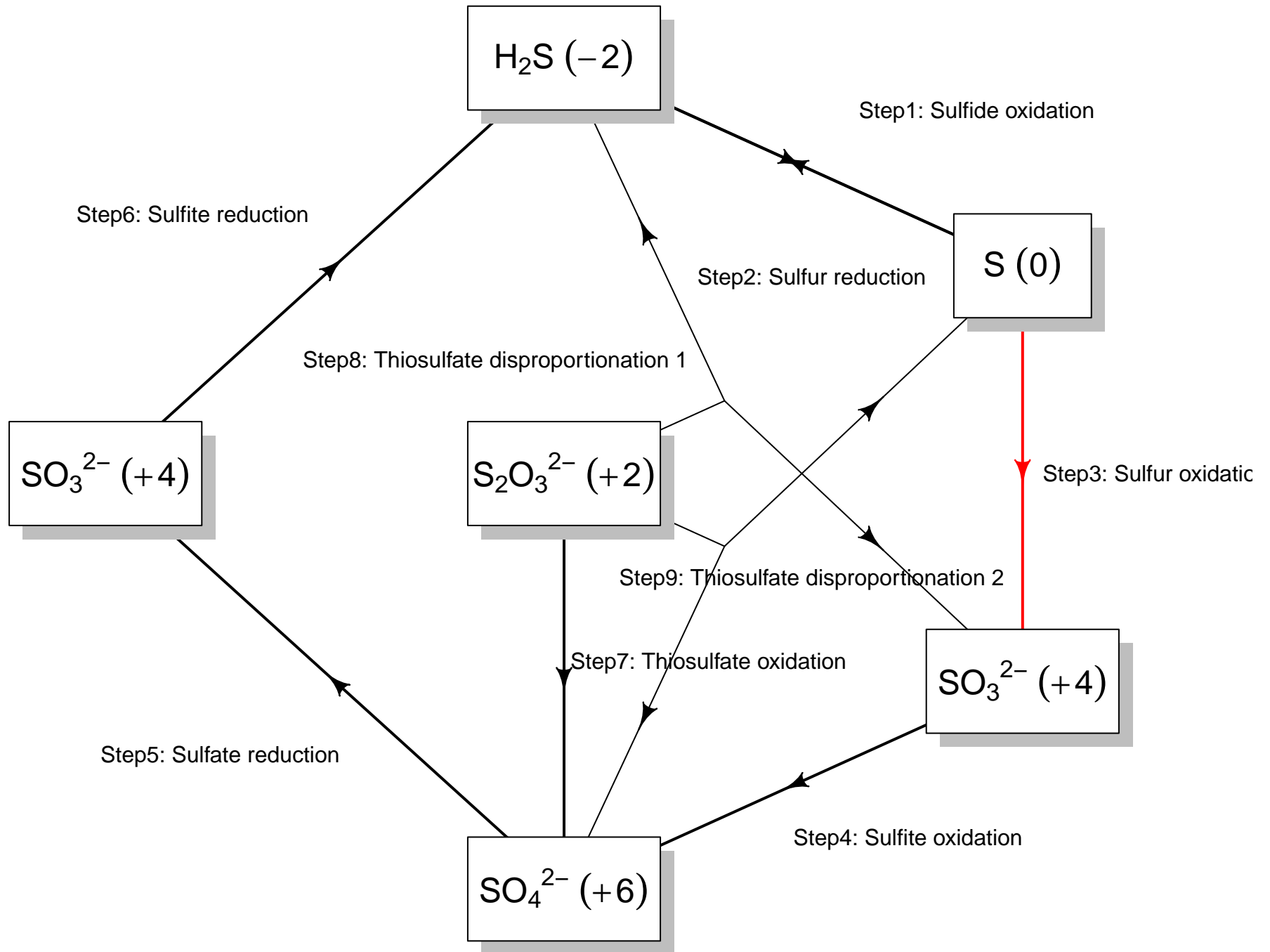

Supplement: Supplementary file 2 — Recovered nutrient cycles including Carbon, Nitrogen, and Sulphur cycles for all the metagenomic assembled genomes given as PDF images, and identifiable through bin numbers. [file 13104_2025_7441_MOESM2_ESM.zip › Supplementary Data2/bin.80.draw_sulfur_cycle_single.pdf]

## Sulfur Cycle: bin.18

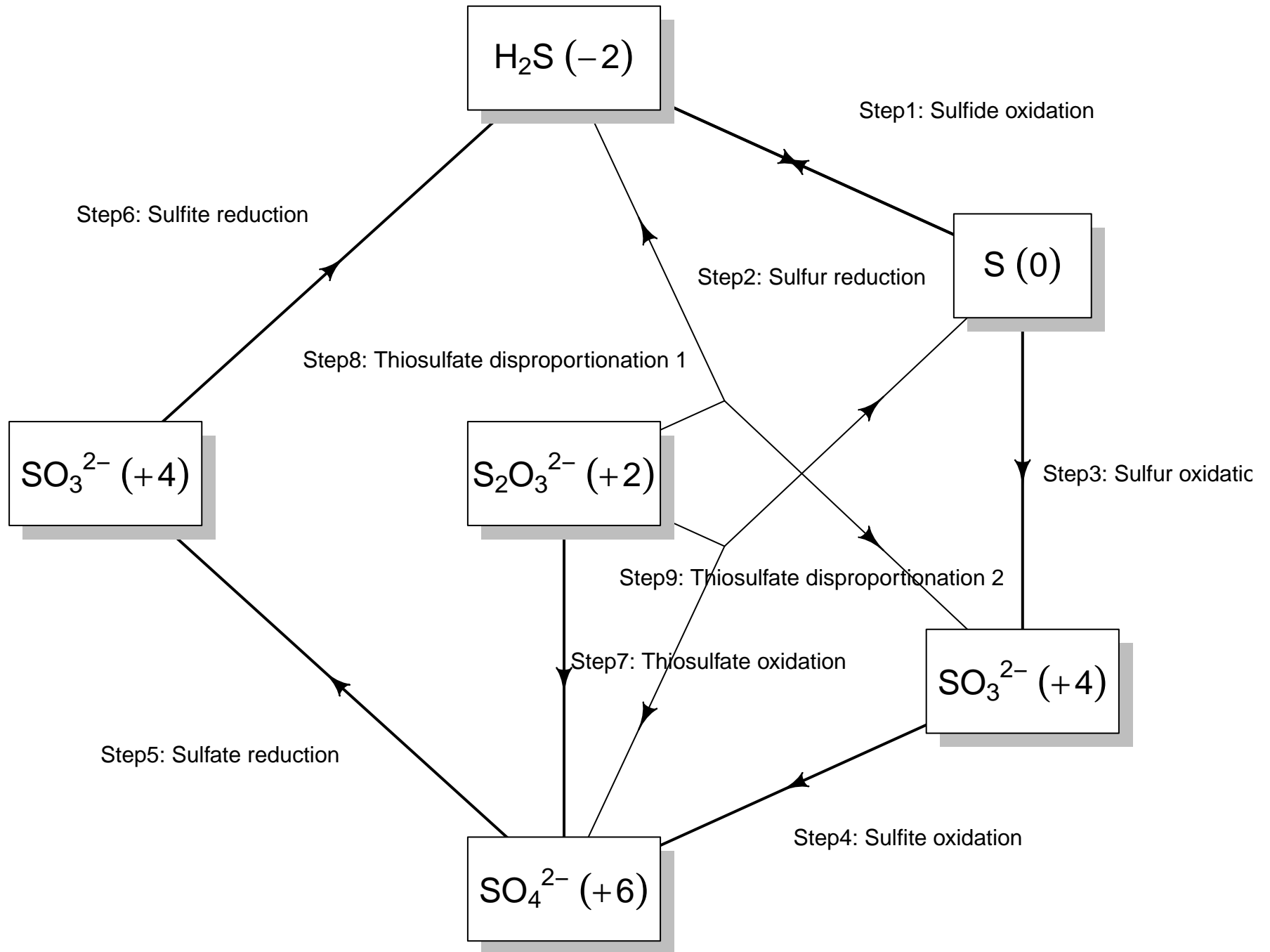

Supplement: Supplementary file 2 — Recovered nutrient cycles including Carbon, Nitrogen, and Sulphur cycles for all the metagenomic assembled genomes given as PDF images, and identifiable through bin numbers. [file 13104_2025_7441_MOESM2_ESM.zip › Supplementary Data2/bin.18.draw_sulfur_cycle_single.pdf]

# Carbon Cycle: bin.106

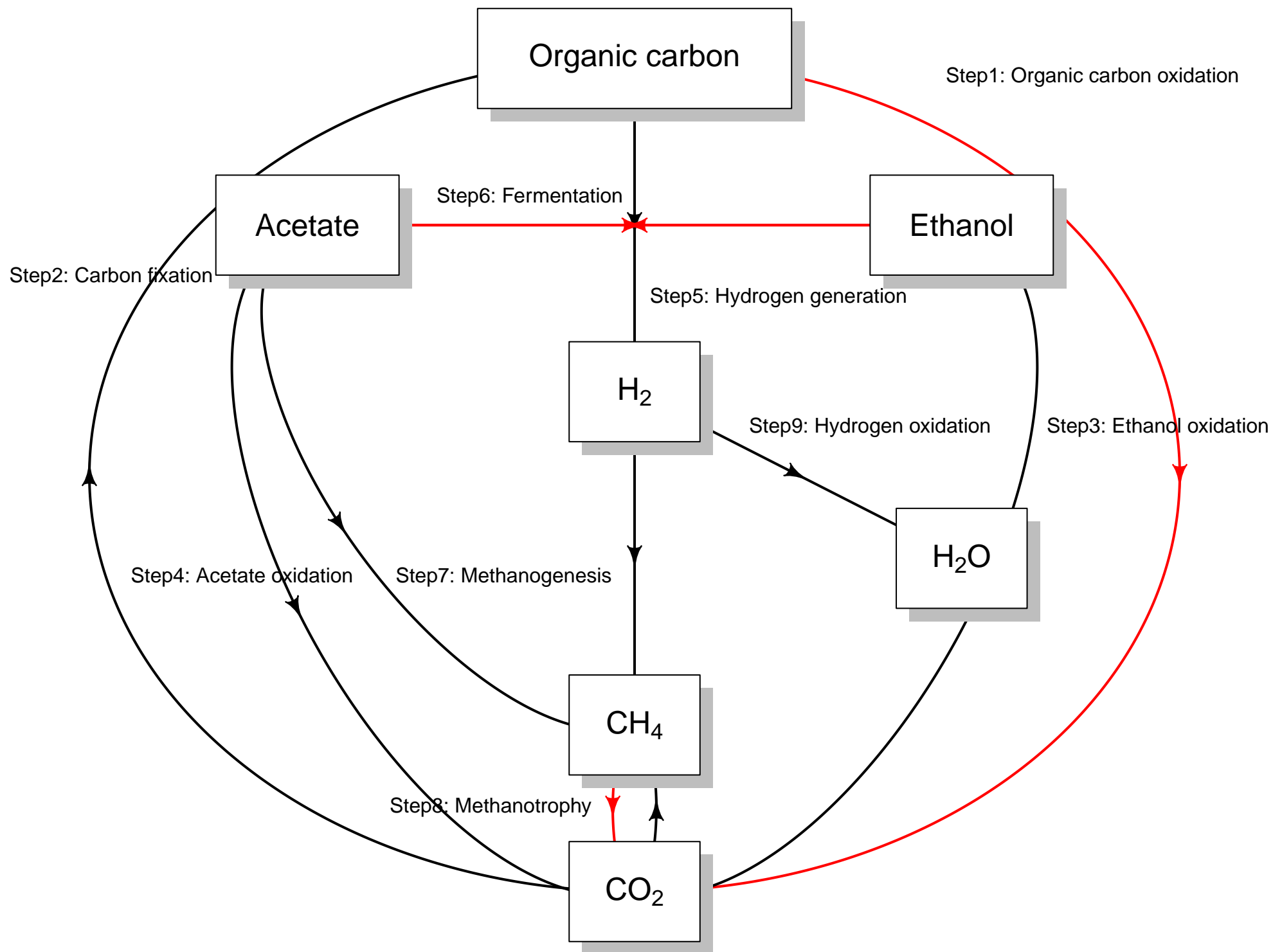

Supplement: Supplementary file 2 — Recovered nutrient cycles including Carbon, Nitrogen, and Sulphur cycles for all the metagenomic assembled genomes given as PDF images, and identifiable through bin numbers. [file 13104_2025_7441_MOESM2_ESM.zip › Supplementary Data2/bin.106.draw_carbon_cycle_single.pdf]

## Sulfur Cycle: bin.16

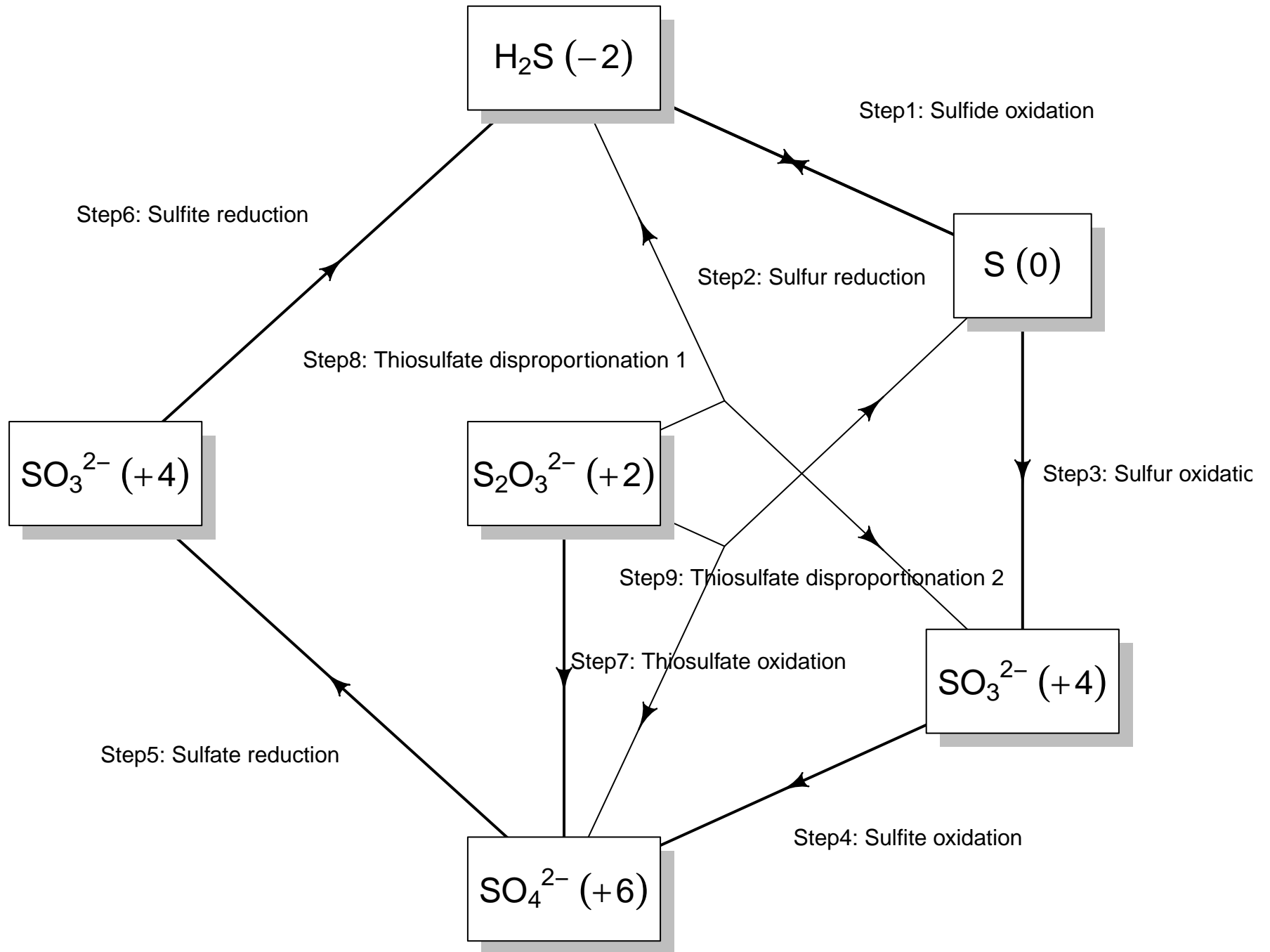

Supplement: Supplementary file 2 — Recovered nutrient cycles including Carbon, Nitrogen, and Sulphur cycles for all the metagenomic assembled genomes given as PDF images, and identifiable through bin numbers. [file 13104_2025_7441_MOESM2_ESM.zip › Supplementary Data2/bin.16.draw_sulfur_cycle_single.pdf]

## Nitrogen Cycle: bin.100

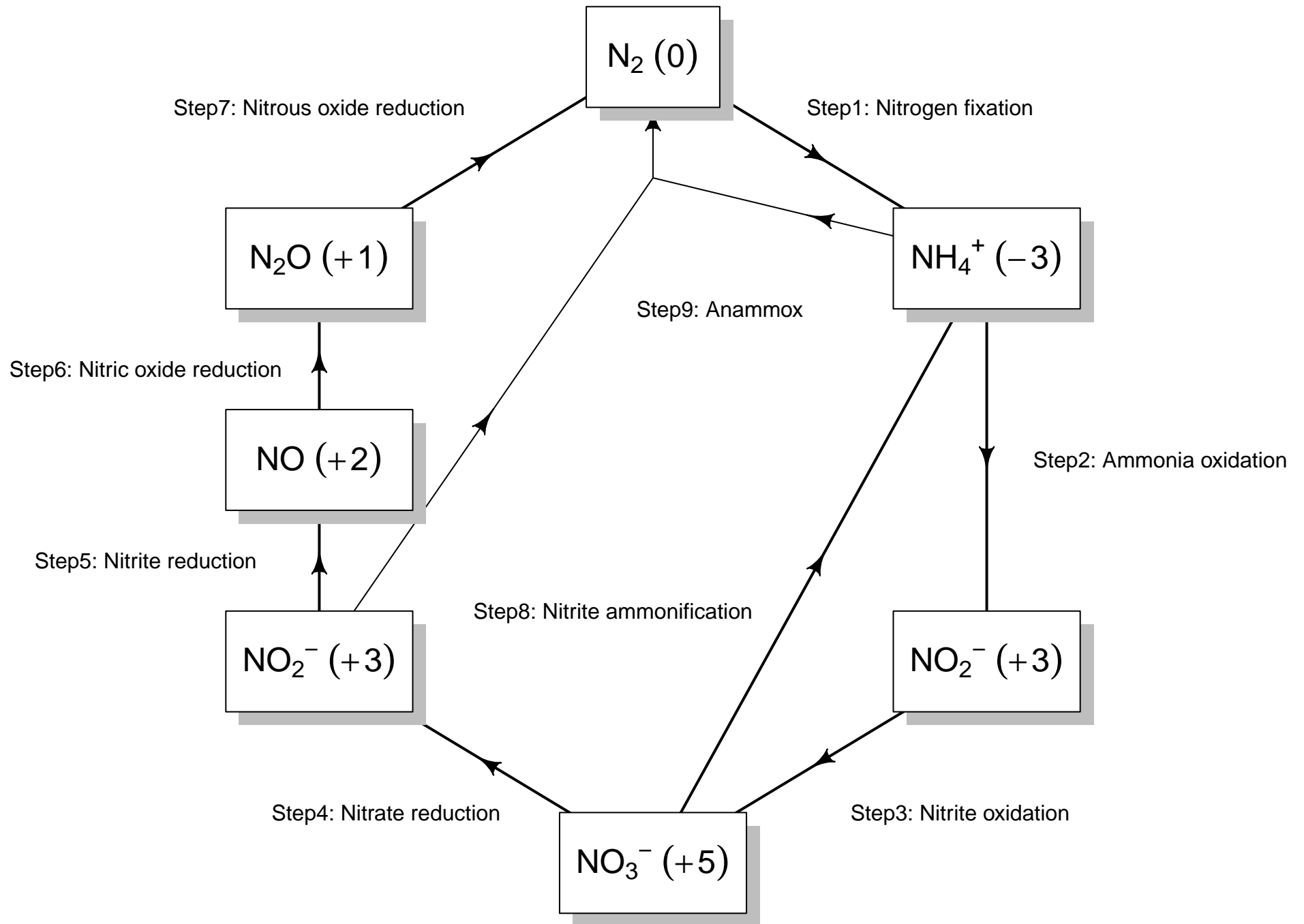

Supplement: Supplementary file 2 — Recovered nutrient cycles including Carbon, Nitrogen, and Sulphur cycles for all the metagenomic assembled genomes given as PDF images, and identifiable through bin numbers. [file 13104_2025_7441_MOESM2_ESM.zip › Supplementary Data2/bin.100.draw_nitrogen_cycle_single.pdf]

# Carbon Cycle: bin.113

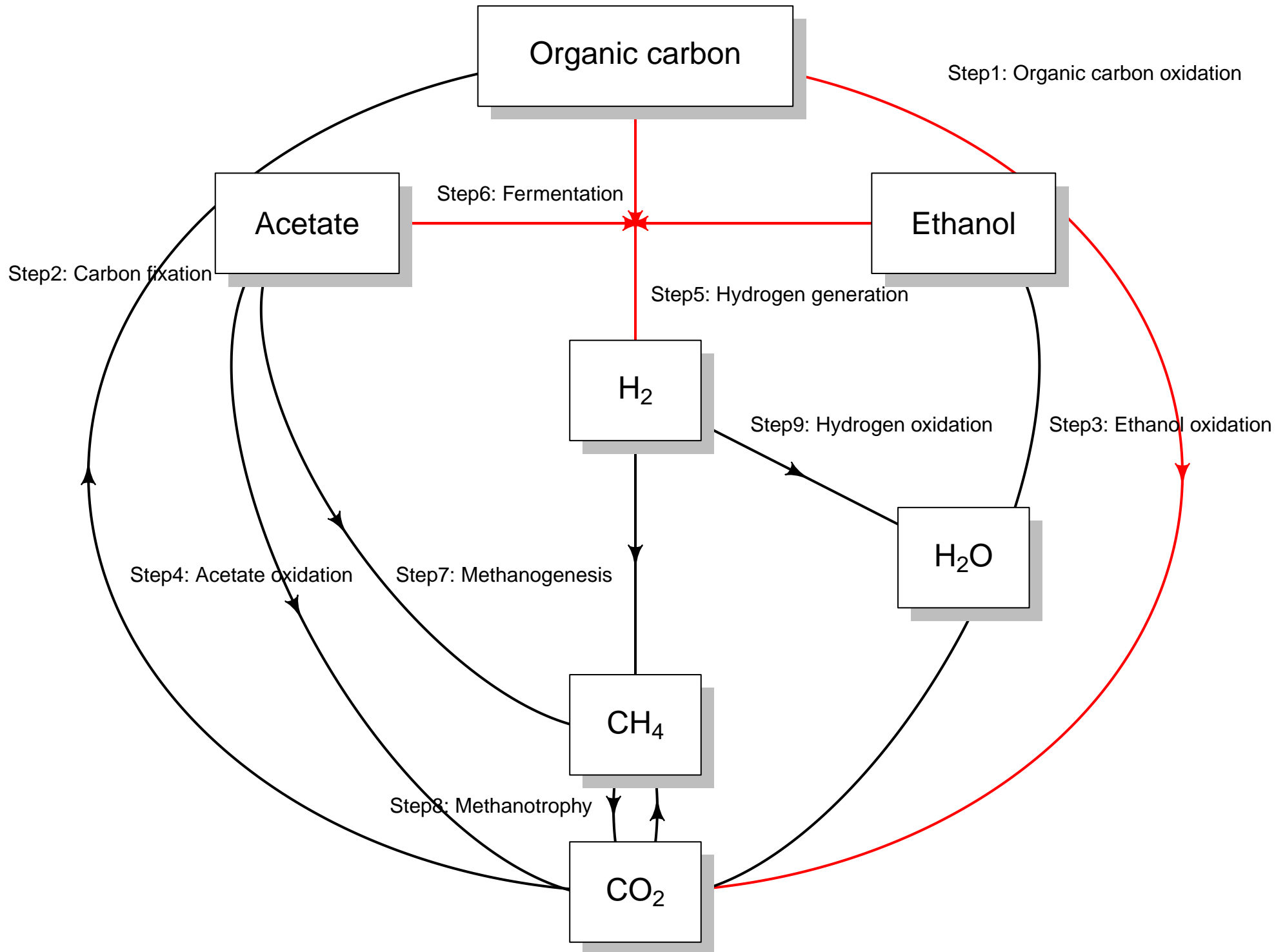

Supplement: Supplementary file 2 — Recovered nutrient cycles including Carbon, Nitrogen, and Sulphur cycles for all the metagenomic assembled genomes given as PDF images, and identifiable through bin numbers. [file 13104_2025_7441_MOESM2_ESM.zip › Supplementary Data2/bin.113.draw_carbon_cycle_single.pdf]

## Nitrogen Cycle: bin.44

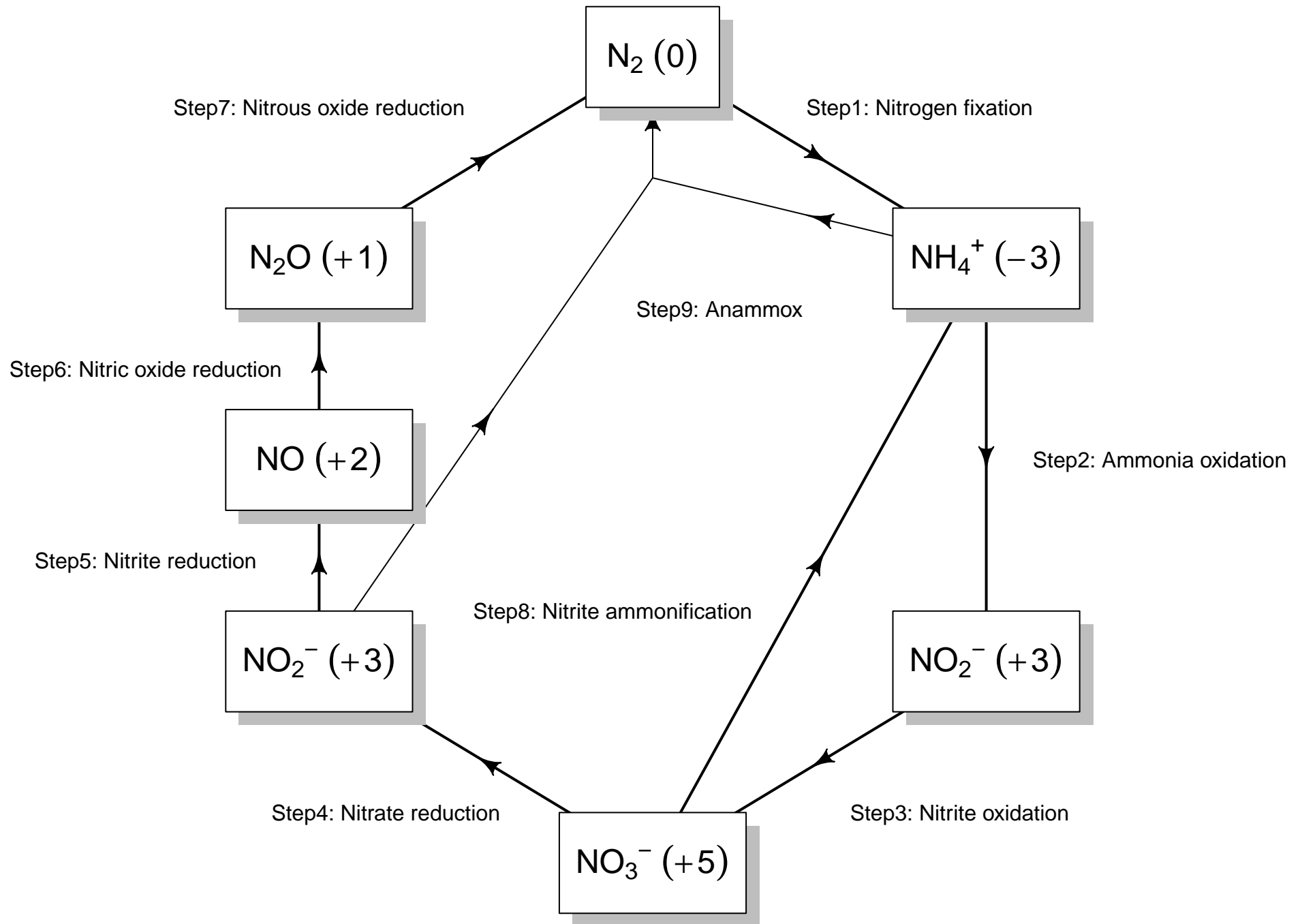

Supplement: Supplementary file 2 — Recovered nutrient cycles including Carbon, Nitrogen, and Sulphur cycles for all the metagenomic assembled genomes given as PDF images, and identifiable through bin numbers. [file 13104_2025_7441_MOESM2_ESM.zip › Supplementary Data2/bin.44.draw_nitrogen_cycle_single.pdf]

## Sulfur Cycle: bin.95

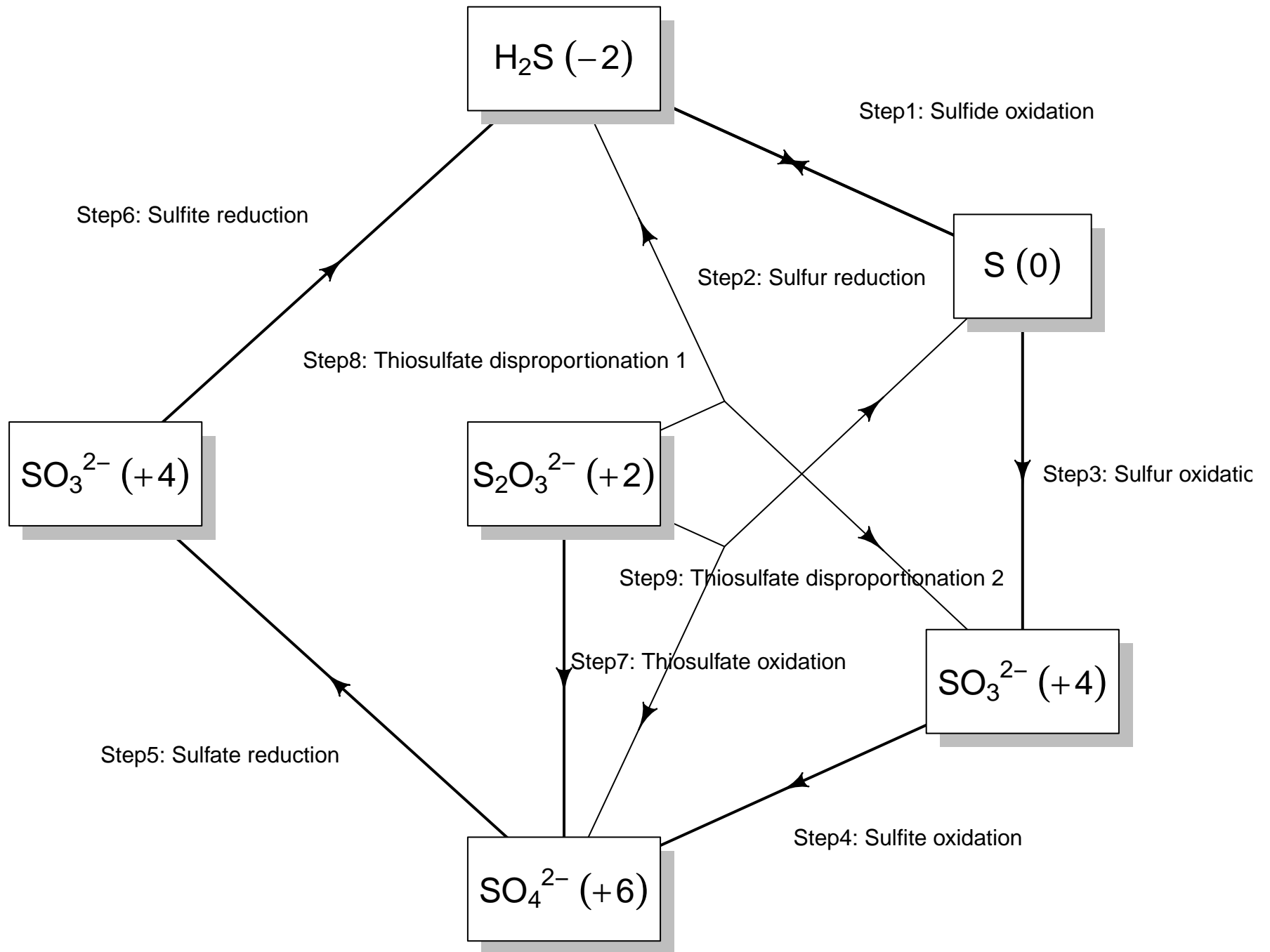

Supplement: Supplementary file 2 — Recovered nutrient cycles including Carbon, Nitrogen, and Sulphur cycles for all the metagenomic assembled genomes given as PDF images, and identifiable through bin numbers. [file 13104_2025_7441_MOESM2_ESM.zip › Supplementary Data2/bin.95.draw_sulfur_cycle_single.pdf]

## Nitrogen Cycle: bin.57

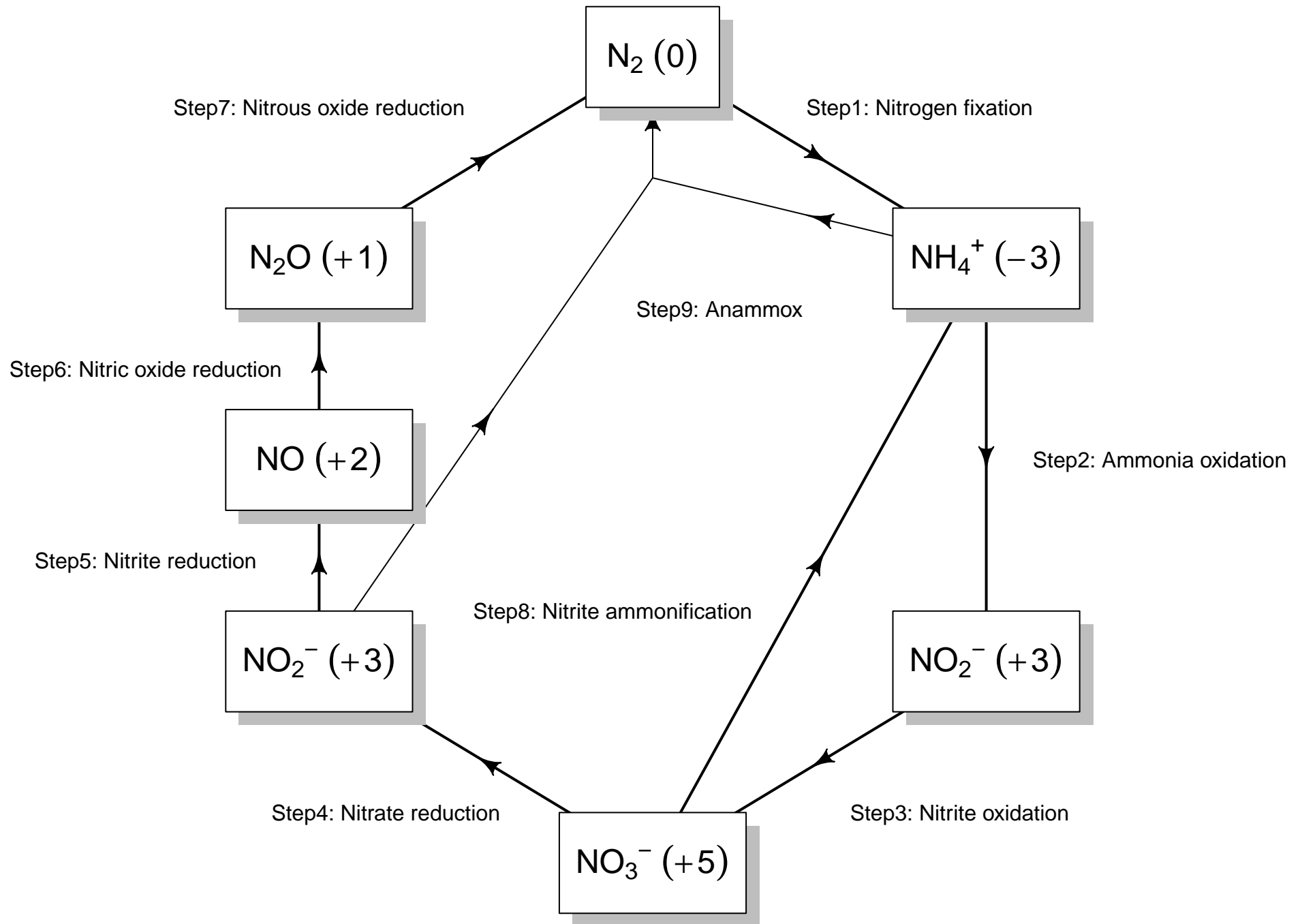

Supplement: Supplementary file 2 — Recovered nutrient cycles including Carbon, Nitrogen, and Sulphur cycles for all the metagenomic assembled genomes given as PDF images, and identifiable through bin numbers. [file 13104_2025_7441_MOESM2_ESM.zip › Supplementary Data2/bin.57.draw_nitrogen_cycle_single.pdf]

## Sulfur Cycle: bin.3

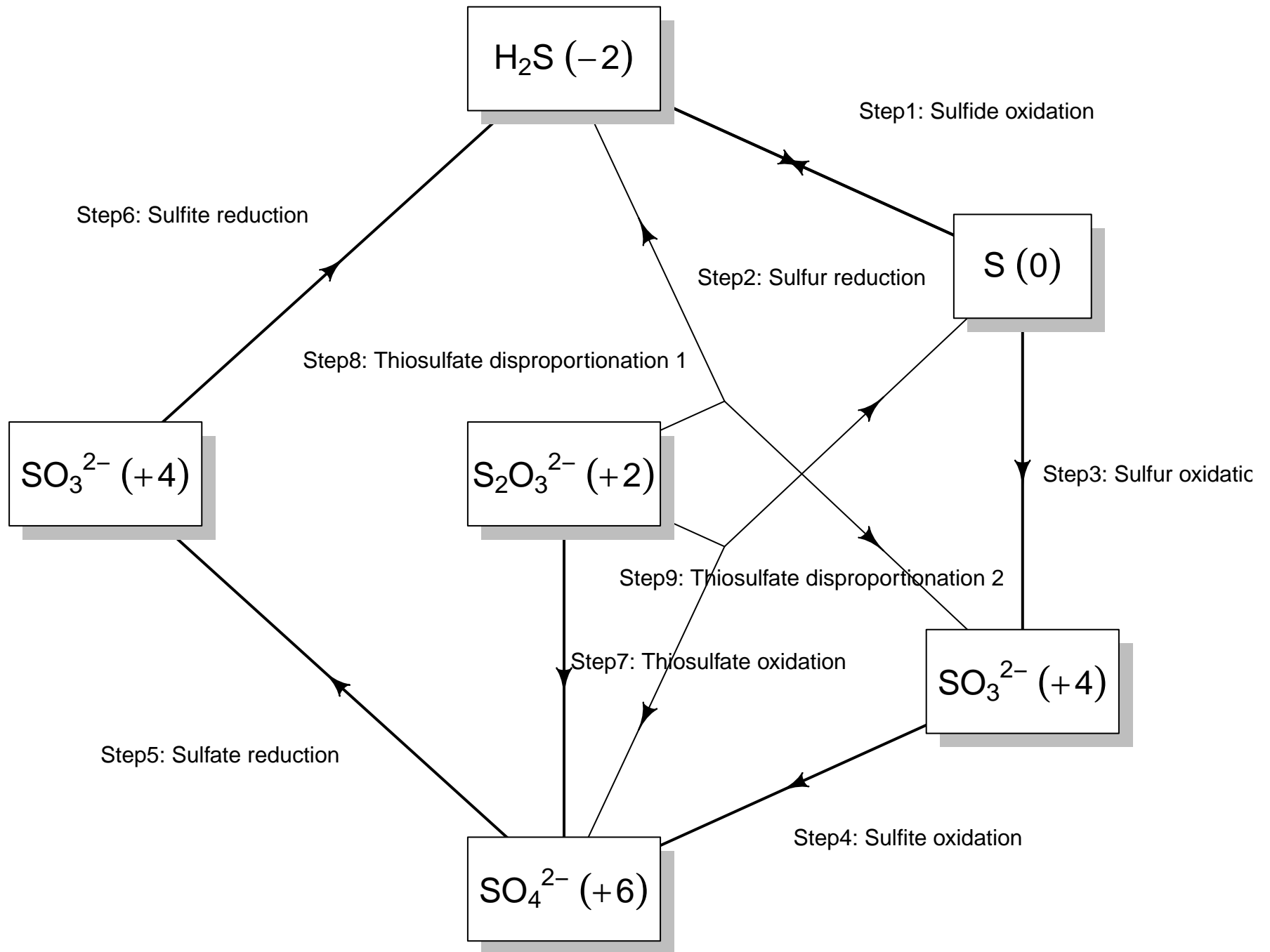

Supplement: Supplementary file 2 — Recovered nutrient cycles including Carbon, Nitrogen, and Sulphur cycles for all the metagenomic assembled genomes given as PDF images, and identifiable through bin numbers. [file 13104_2025_7441_MOESM2_ESM.zip › Supplementary Data2/bin.3.draw_sulfur_cycle_single.pdf]

## Sulfur Cycle: bin.130

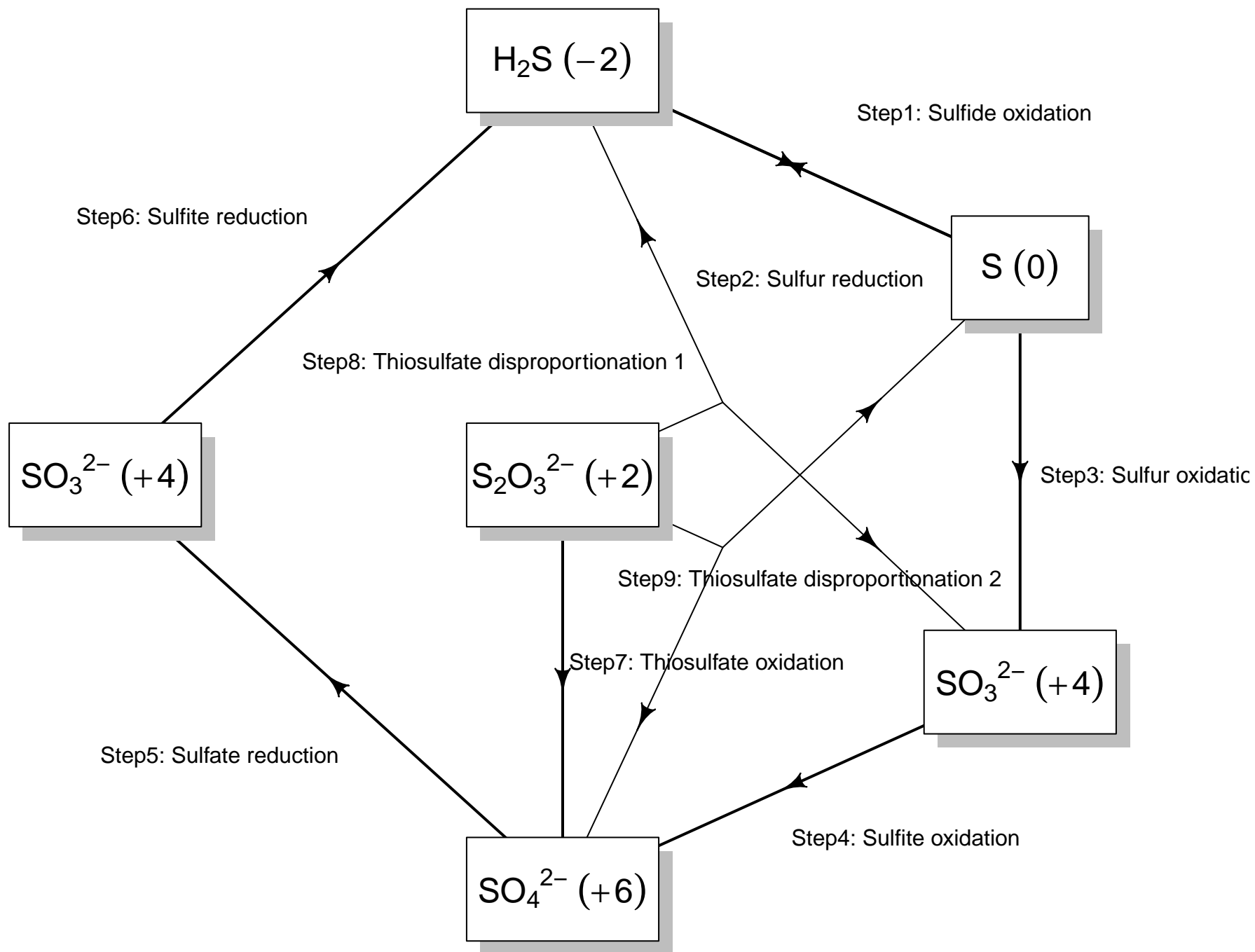

Supplement: Supplementary file 2 — Recovered nutrient cycles including Carbon, Nitrogen, and Sulphur cycles for all the metagenomic assembled genomes given as PDF images, and identifiable through bin numbers. [file 13104_2025_7441_MOESM2_ESM.zip › Supplementary Data2/bin.130.draw_sulfur_cycle_single.pdf]

# Carbon Cycle: bin.20

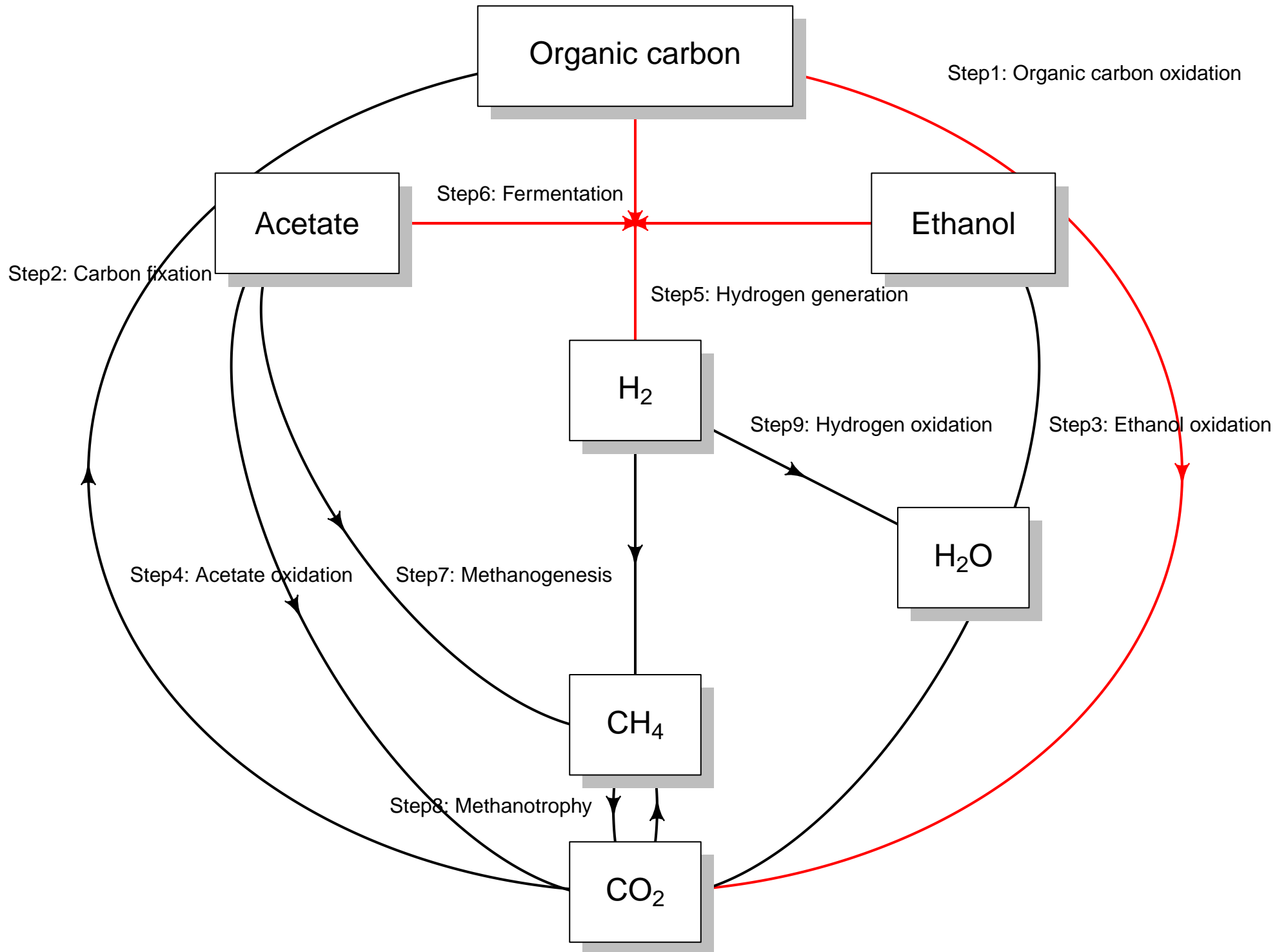

Supplement: Supplementary file 2 — Recovered nutrient cycles including Carbon, Nitrogen, and Sulphur cycles for all the metagenomic assembled genomes given as PDF images, and identifiable through bin numbers. [file 13104_2025_7441_MOESM2_ESM.zip › Supplementary Data2/bin.20.draw_carbon_cycle_single.pdf]

## Nitrogen Cycle: bin.18

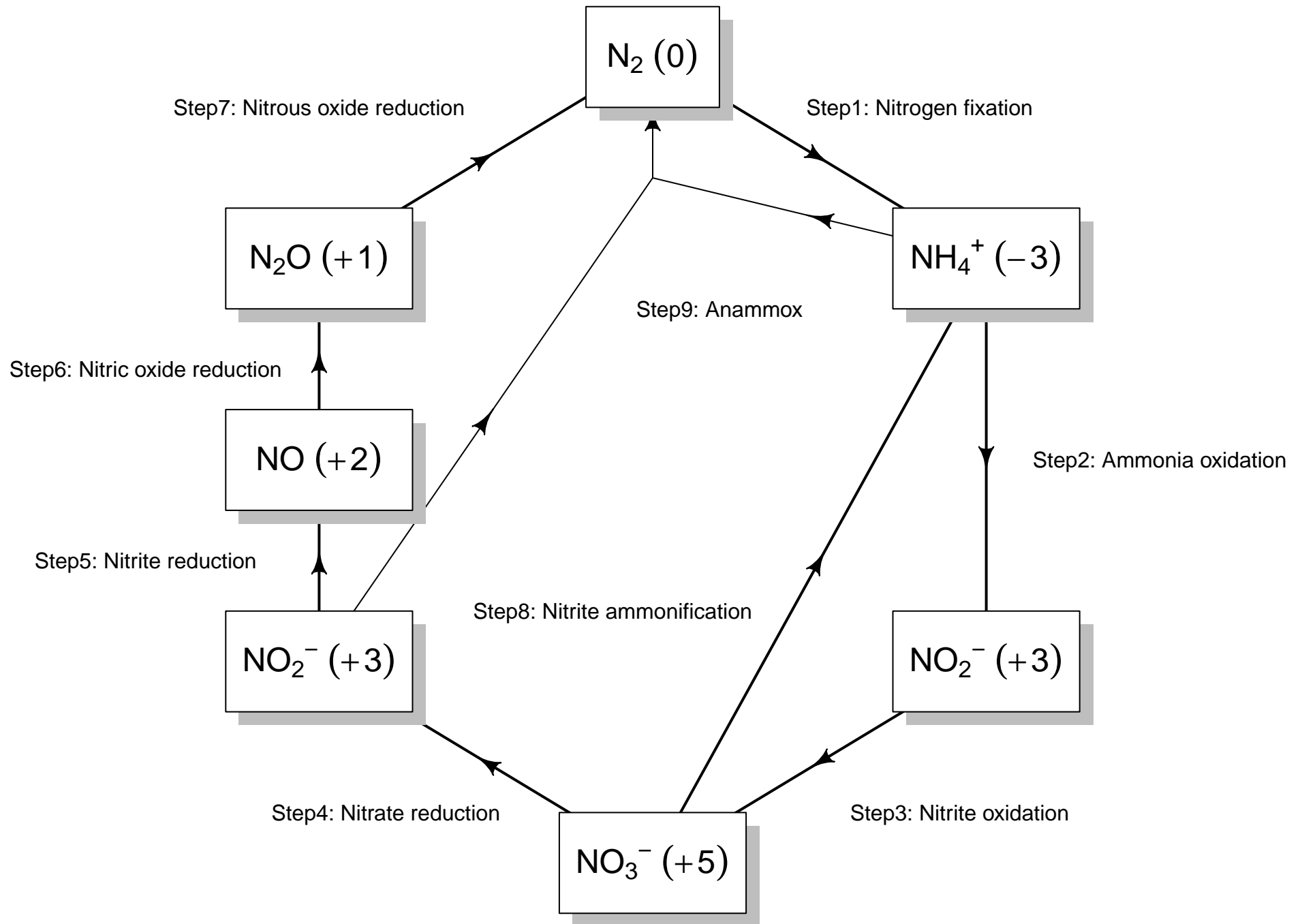

Supplement: Supplementary file 2 — Recovered nutrient cycles including Carbon, Nitrogen, and Sulphur cycles for all the metagenomic assembled genomes given as PDF images, and identifiable through bin numbers. [file 13104_2025_7441_MOESM2_ESM.zip › Supplementary Data2/bin.18.draw_nitrogen_cycle_single.pdf]

## Nitrogen Cycle: bin.113

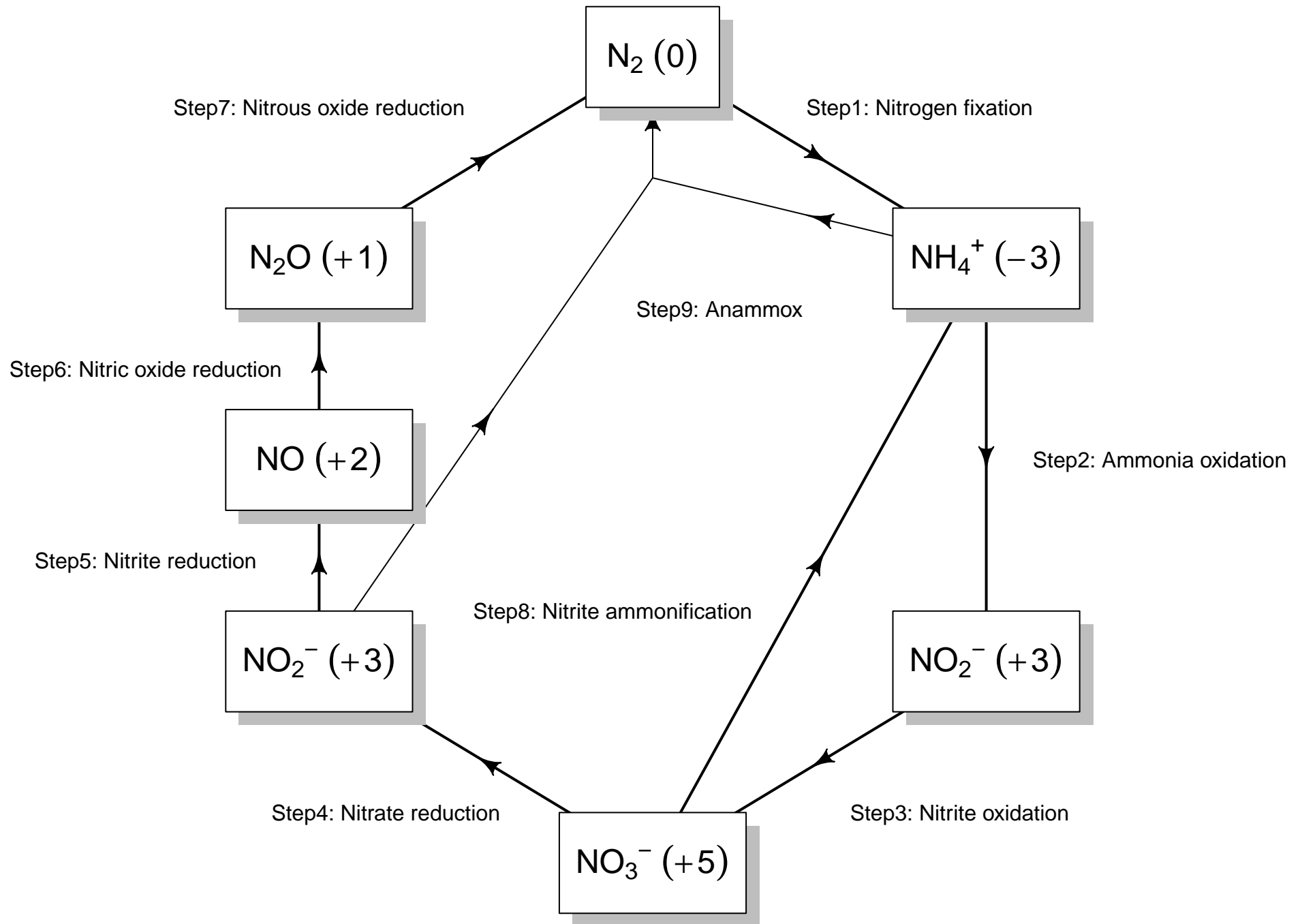

Supplement: Supplementary file 2 — Recovered nutrient cycles including Carbon, Nitrogen, and Sulphur cycles for all the metagenomic assembled genomes given as PDF images, and identifiable through bin numbers. [file 13104_2025_7441_MOESM2_ESM.zip › Supplementary Data2/bin.113.draw_nitrogen_cycle_single.pdf]

## Sulfur Cycle: bin.54

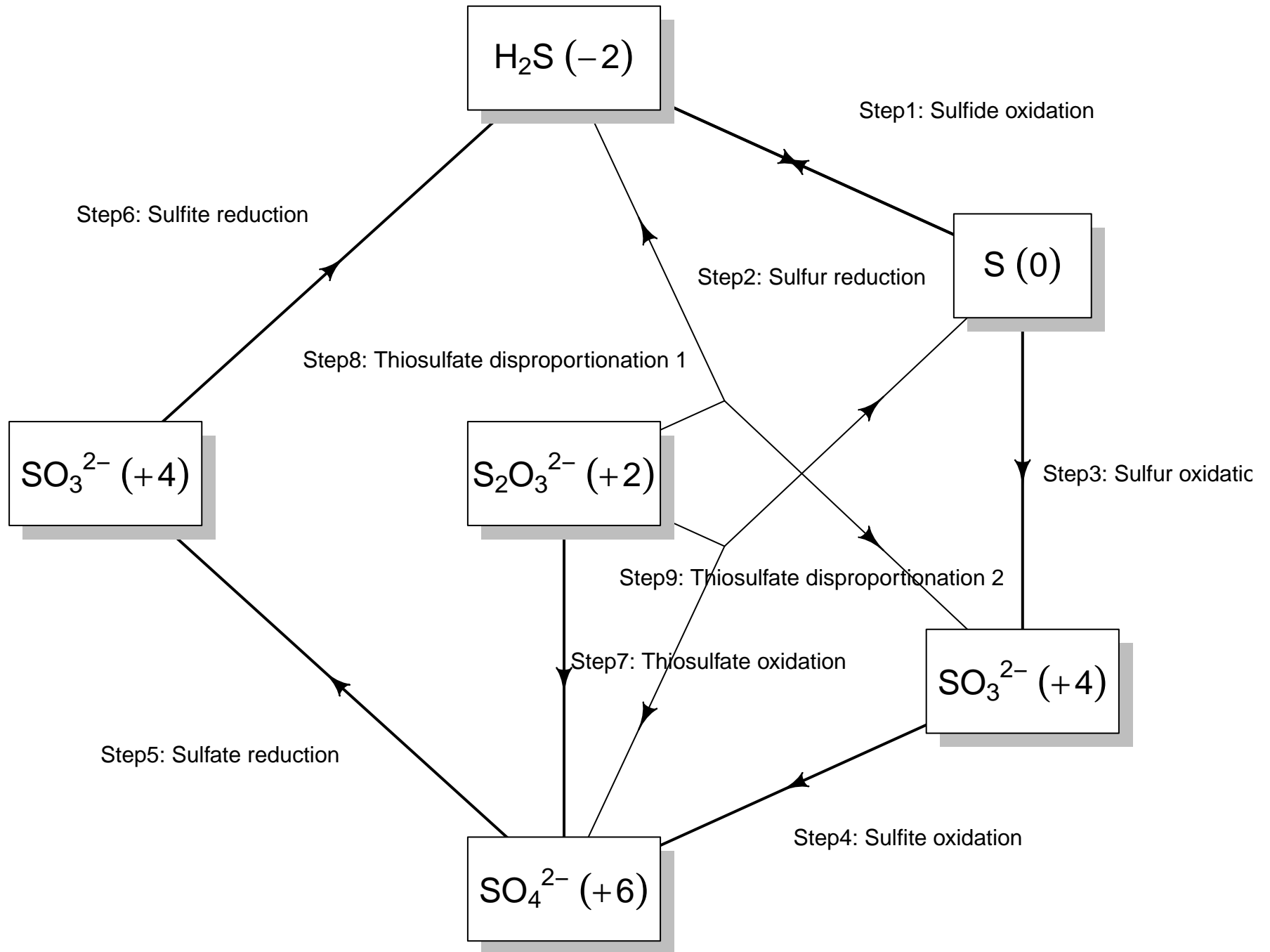

Supplement: Supplementary file 2 — Recovered nutrient cycles including Carbon, Nitrogen, and Sulphur cycles for all the metagenomic assembled genomes given as PDF images, and identifiable through bin numbers. [file 13104_2025_7441_MOESM2_ESM.zip › Supplementary Data2/bin.54.draw_sulfur_cycle_single.pdf]

# Nitrogen Cycle: bin.145

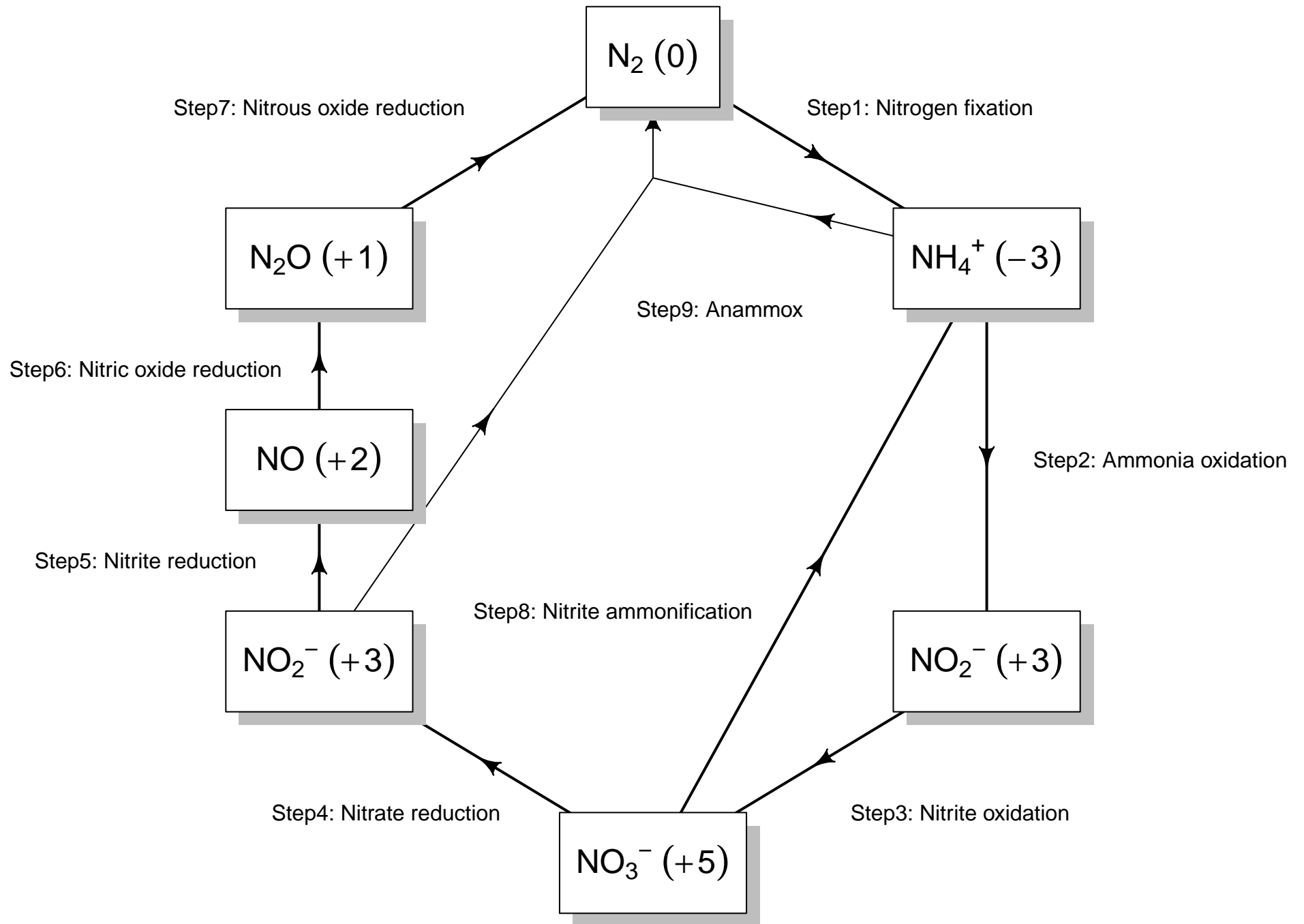

Supplement: Supplementary file 2 — Recovered nutrient cycles including Carbon, Nitrogen, and Sulphur cycles for all the metagenomic assembled genomes given as PDF images, and identifiable through bin numbers. [file 13104_2025_7441_MOESM2_ESM.zip › Supplementary Data2/bin.145.draw_nitrogen_cycle_single.pdf]

# Nitrogen Cycle: bin.119

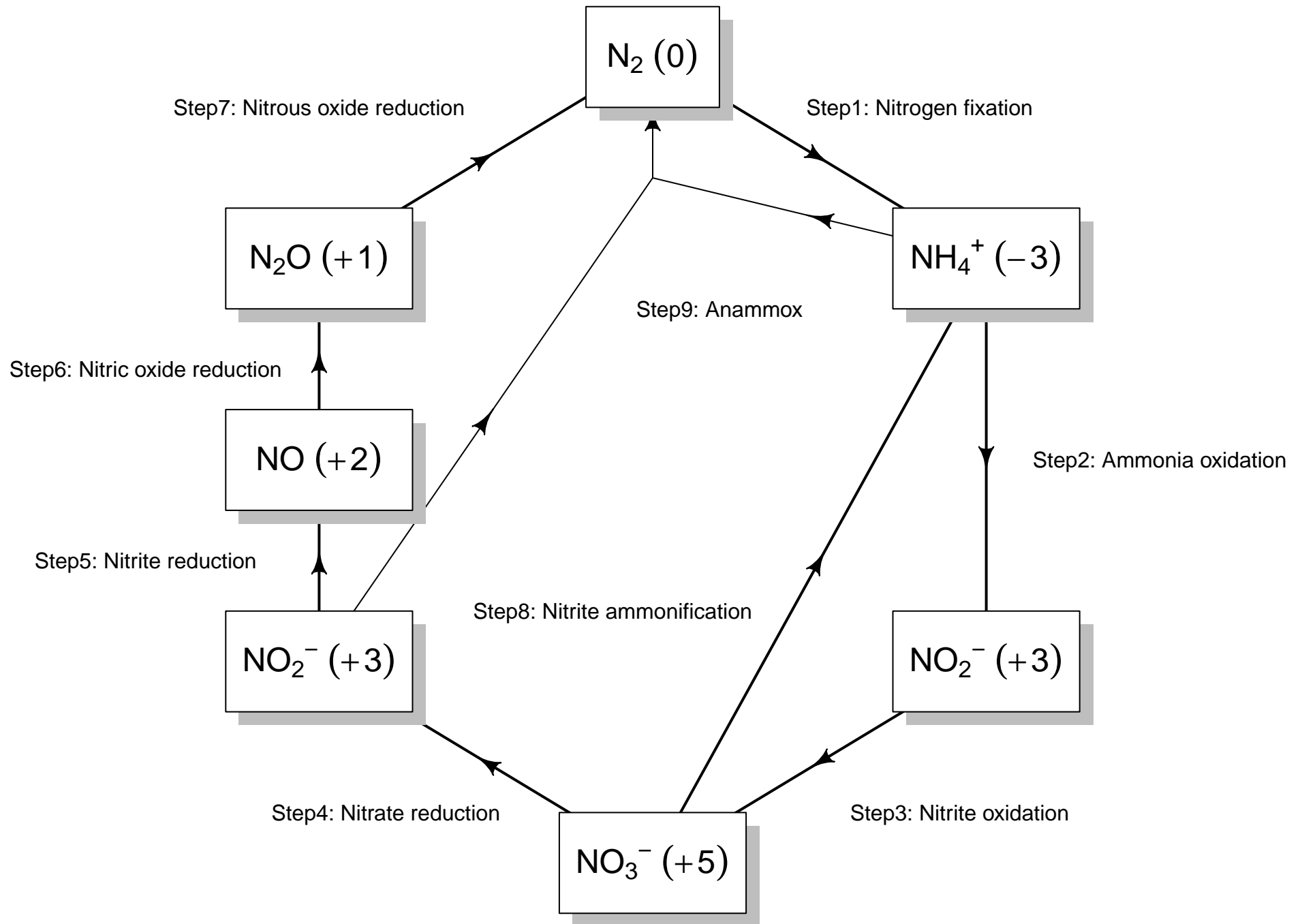

Supplement: Supplementary file 2 — Recovered nutrient cycles including Carbon, Nitrogen, and Sulphur cycles for all the metagenomic assembled genomes given as PDF images, and identifiable through bin numbers. [file 13104_2025_7441_MOESM2_ESM.zip › Supplementary Data2/bin.119.draw_nitrogen_cycle_single.pdf]

# Carbon Cycle: bin.77

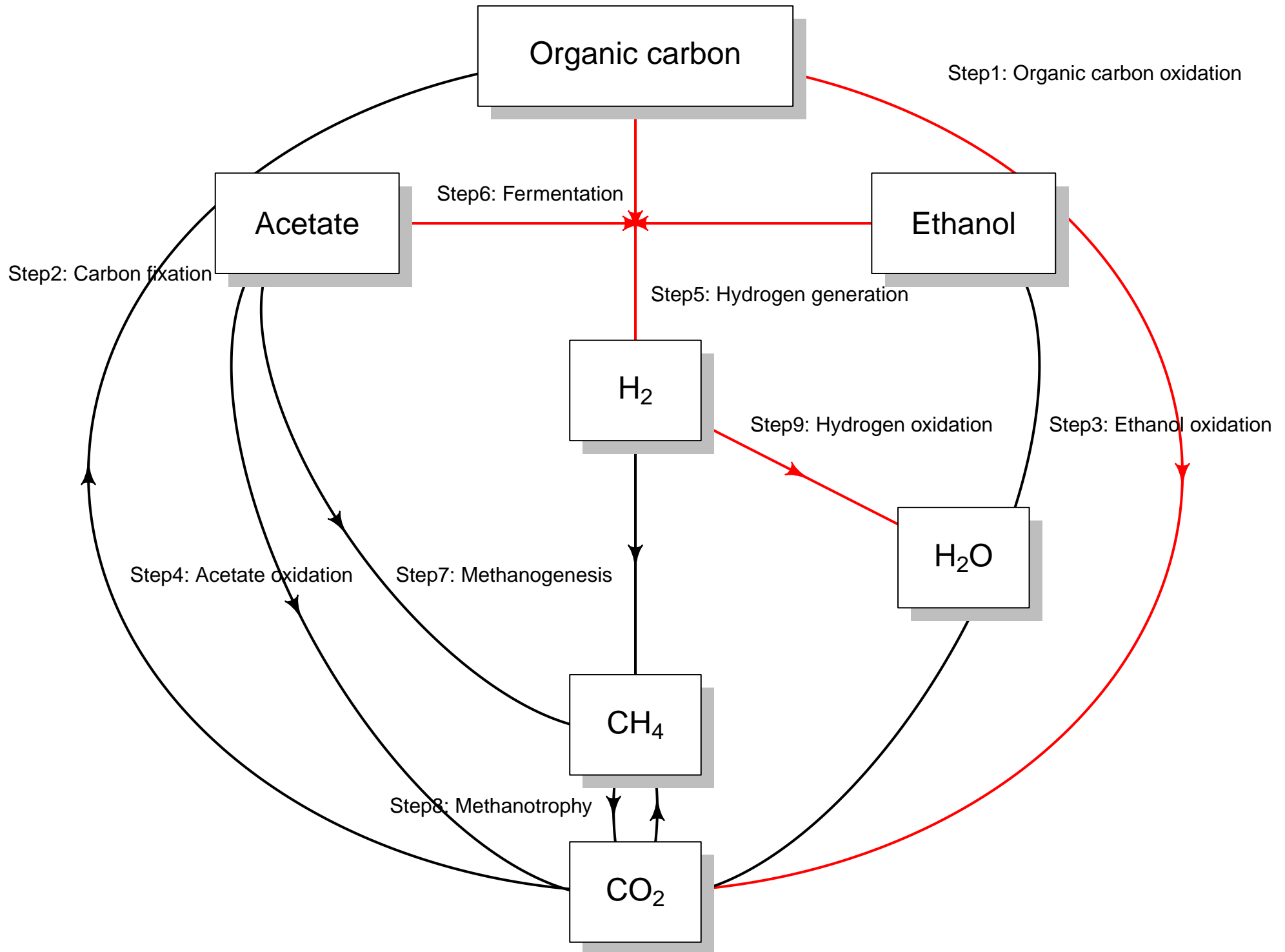

Supplement: Supplementary file 2 — Recovered nutrient cycles including Carbon, Nitrogen, and Sulphur cycles for all the metagenomic assembled genomes given as PDF images, and identifiable through bin numbers. [file 13104_2025_7441_MOESM2_ESM.zip › Supplementary Data2/bin.77.draw_carbon_cycle_single.pdf]
