# Supplementary figures and images for: Investigating microbial population structure and function in the chicken caeca and large intestine over time using metagenomics (part 1 of 2)
Source: BMC Res Notes. 2025 Aug 15;18:355. doi: 10.1186/s13104-025-07441-7 (PMC12357378; doi:10.1186/s13104-025-07441-7)

# Carbon Cycle: bin.43

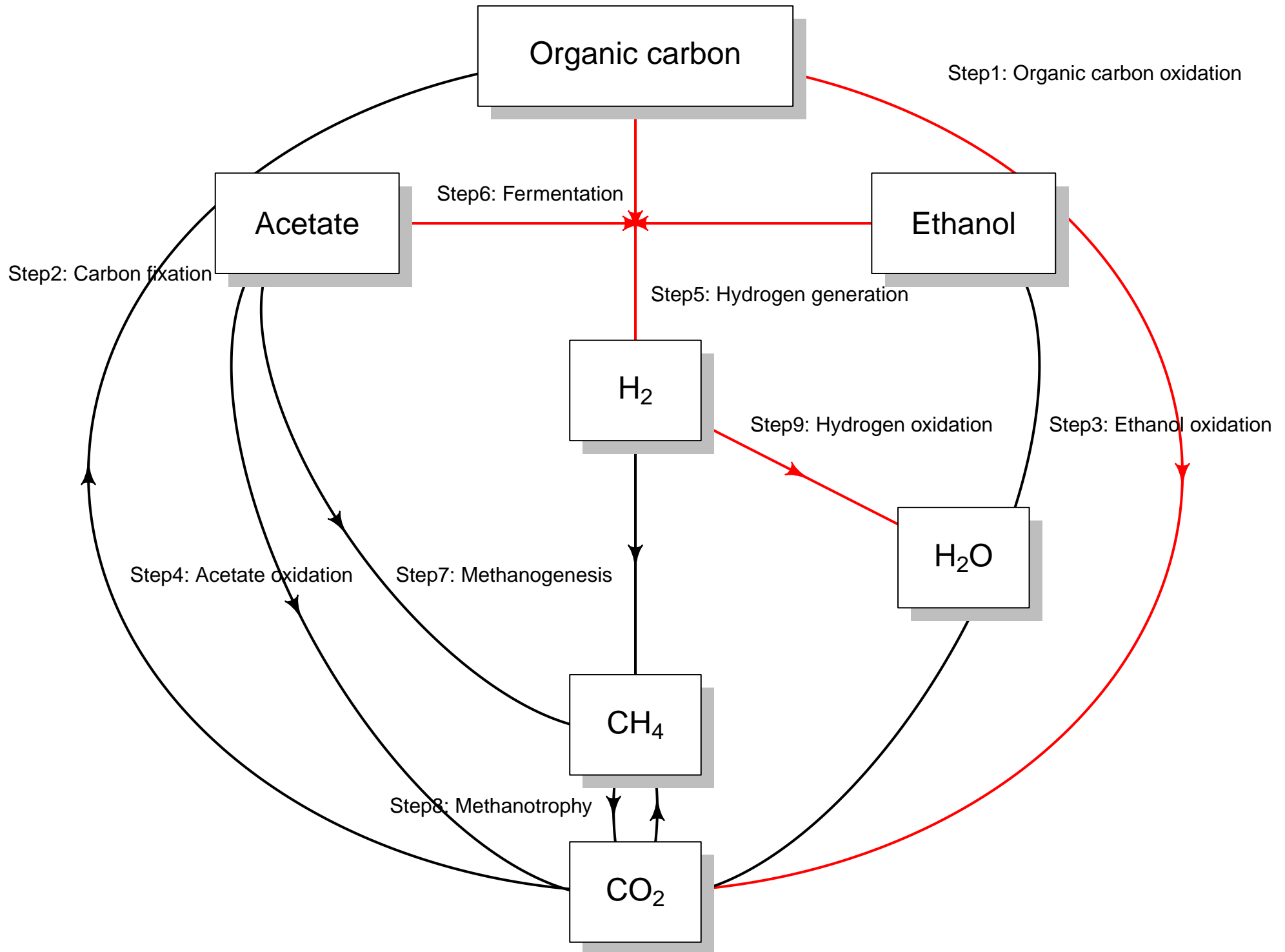

Supplement: Supplementary file 2 — Recovered nutrient cycles including Carbon, Nitrogen, and Sulphur cycles for all the metagenomic assembled genomes given as PDF images, and identifiable through bin numbers. [file 13104_2025_7441_MOESM2_ESM.zip › Supplementary Data2/bin.43.draw_carbon_cycle_single.pdf]

## Sulfur Cycle: bin.60

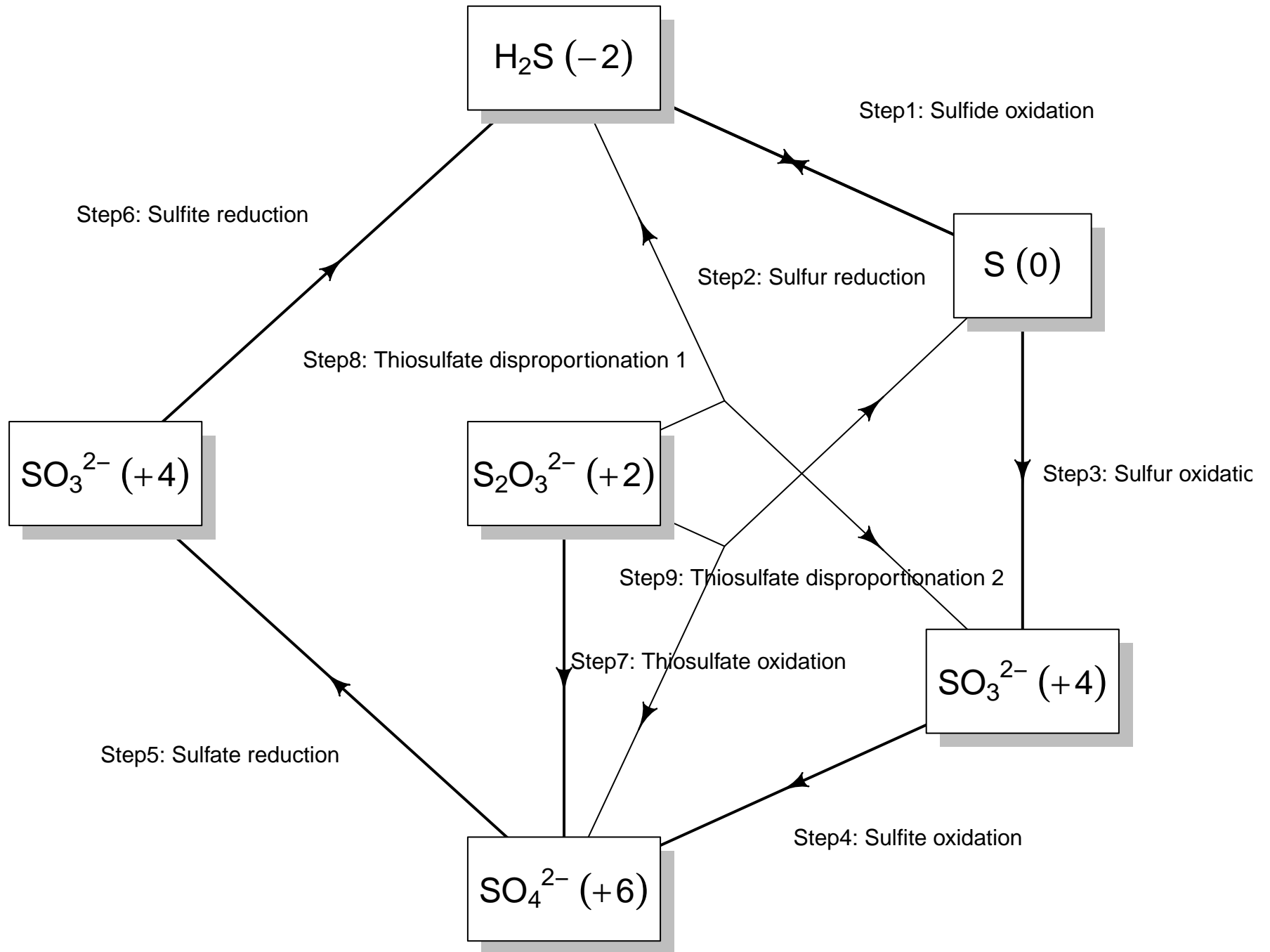

Supplement: Supplementary file 2 — Recovered nutrient cycles including Carbon, Nitrogen, and Sulphur cycles for all the metagenomic assembled genomes given as PDF images, and identifiable through bin numbers. [file 13104_2025_7441_MOESM2_ESM.zip › Supplementary Data2/bin.60.draw_sulfur_cycle_single.pdf]

## Nitrogen Cycle: bin.19

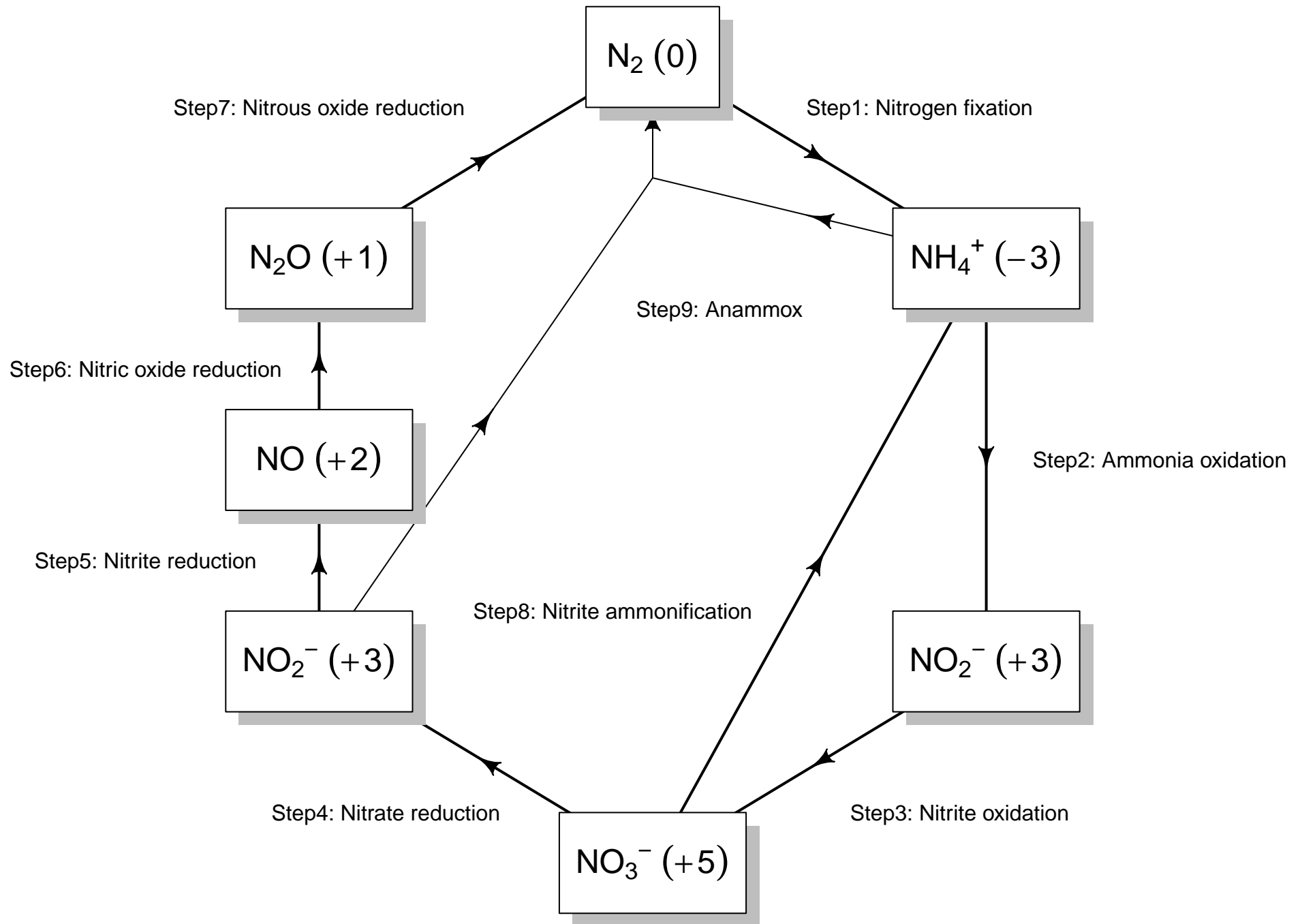

Supplement: Supplementary file 2 — Recovered nutrient cycles including Carbon, Nitrogen, and Sulphur cycles for all the metagenomic assembled genomes given as PDF images, and identifiable through bin numbers. [file 13104_2025_7441_MOESM2_ESM.zip › Supplementary Data2/bin.19.draw_nitrogen_cycle_single.pdf]

# Nitrogen Cycle: bin.112

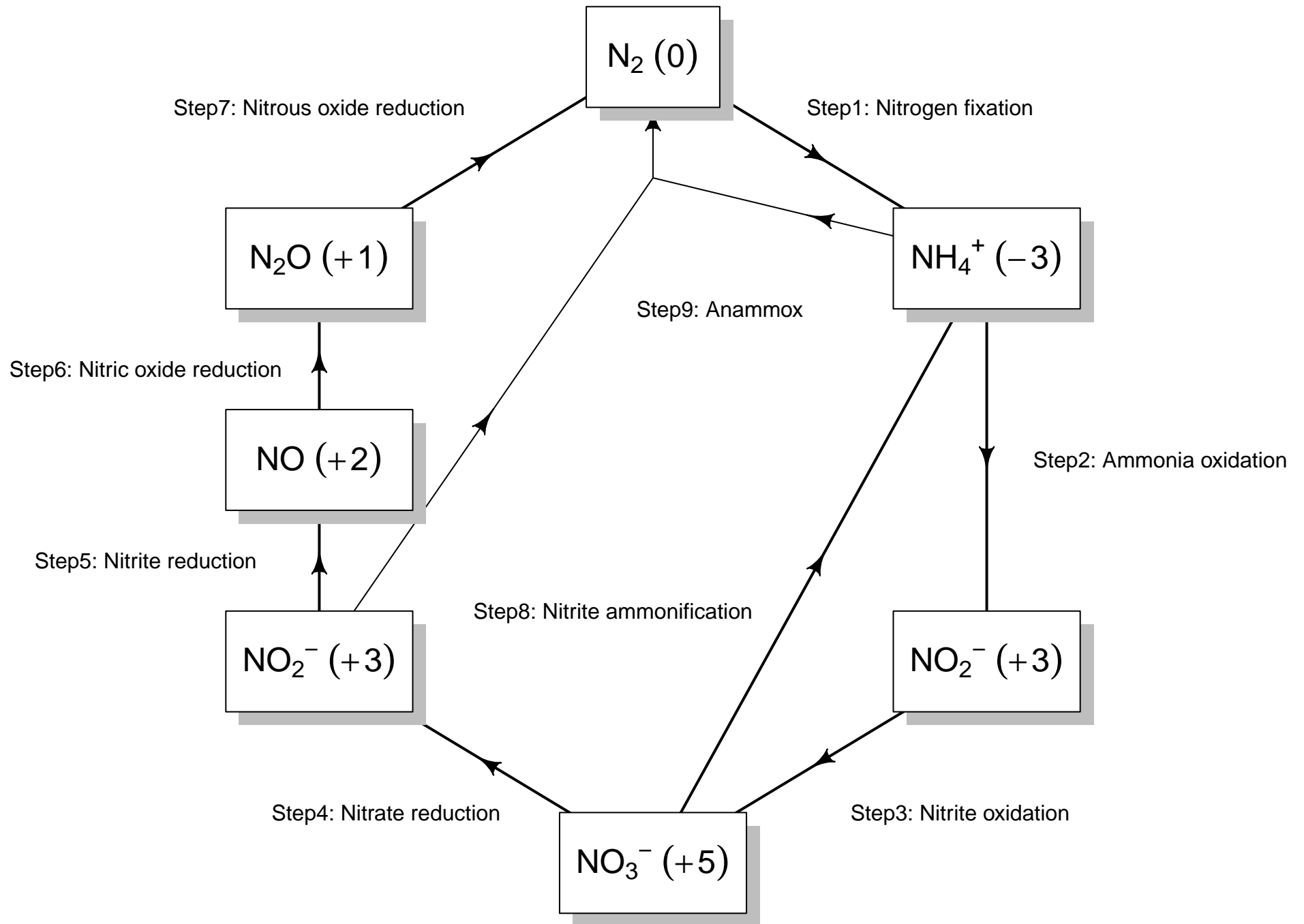

Supplement: Supplementary file 2 — Recovered nutrient cycles including Carbon, Nitrogen, and Sulphur cycles for all the metagenomic assembled genomes given as PDF images, and identifiable through bin numbers. [file 13104_2025_7441_MOESM2_ESM.zip › Supplementary Data2/bin.112.draw_nitrogen_cycle_single.pdf]

# Nitrogen Cycle: bin.56

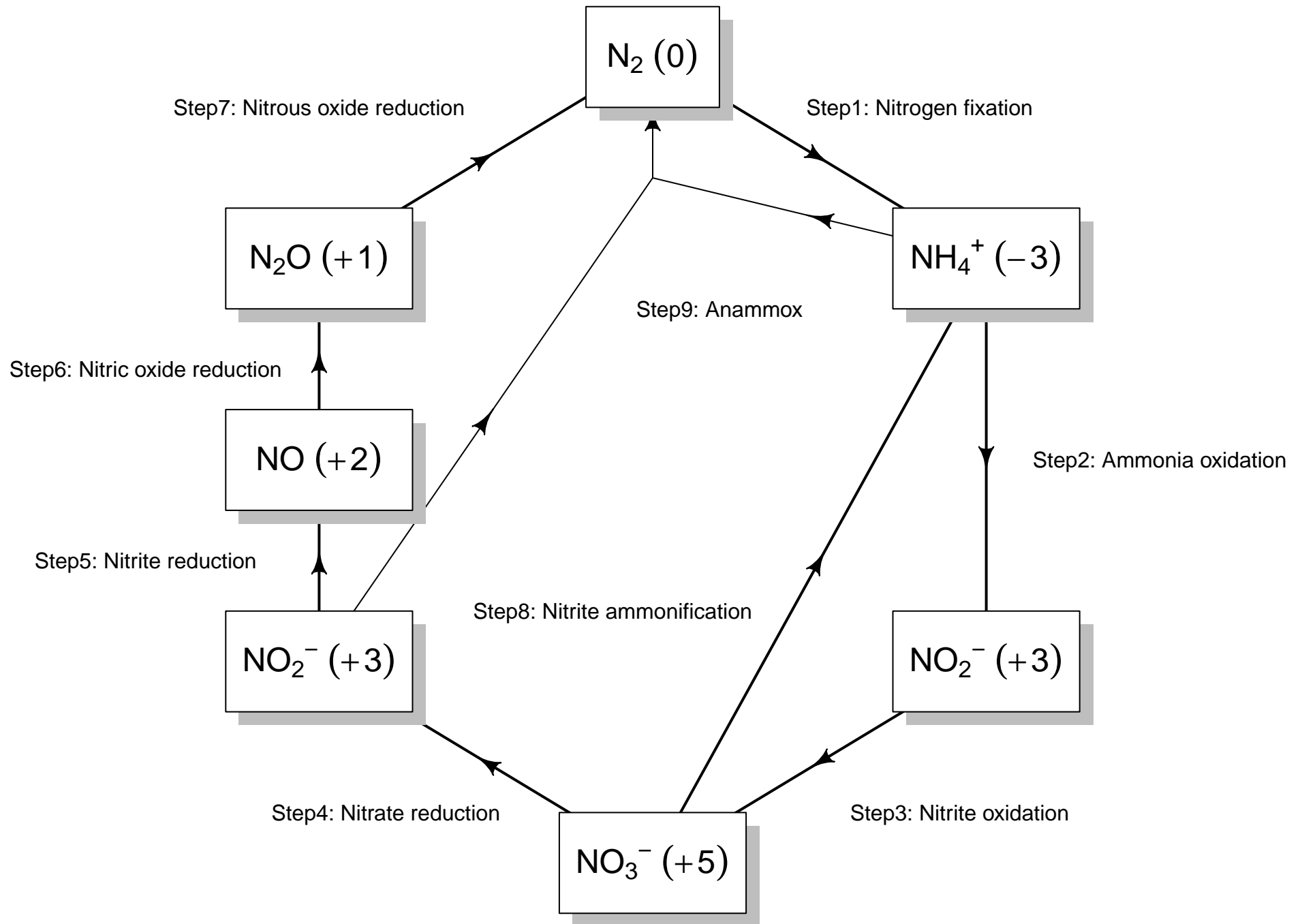

Supplement: Supplementary file 2 — Recovered nutrient cycles including Carbon, Nitrogen, and Sulphur cycles for all the metagenomic assembled genomes given as PDF images, and identifiable through bin numbers. [file 13104_2025_7441_MOESM2_ESM.zip › Supplementary Data2/bin.56.draw_nitrogen_cycle_single.pdf]

## Sulfur Cycle: bin.75

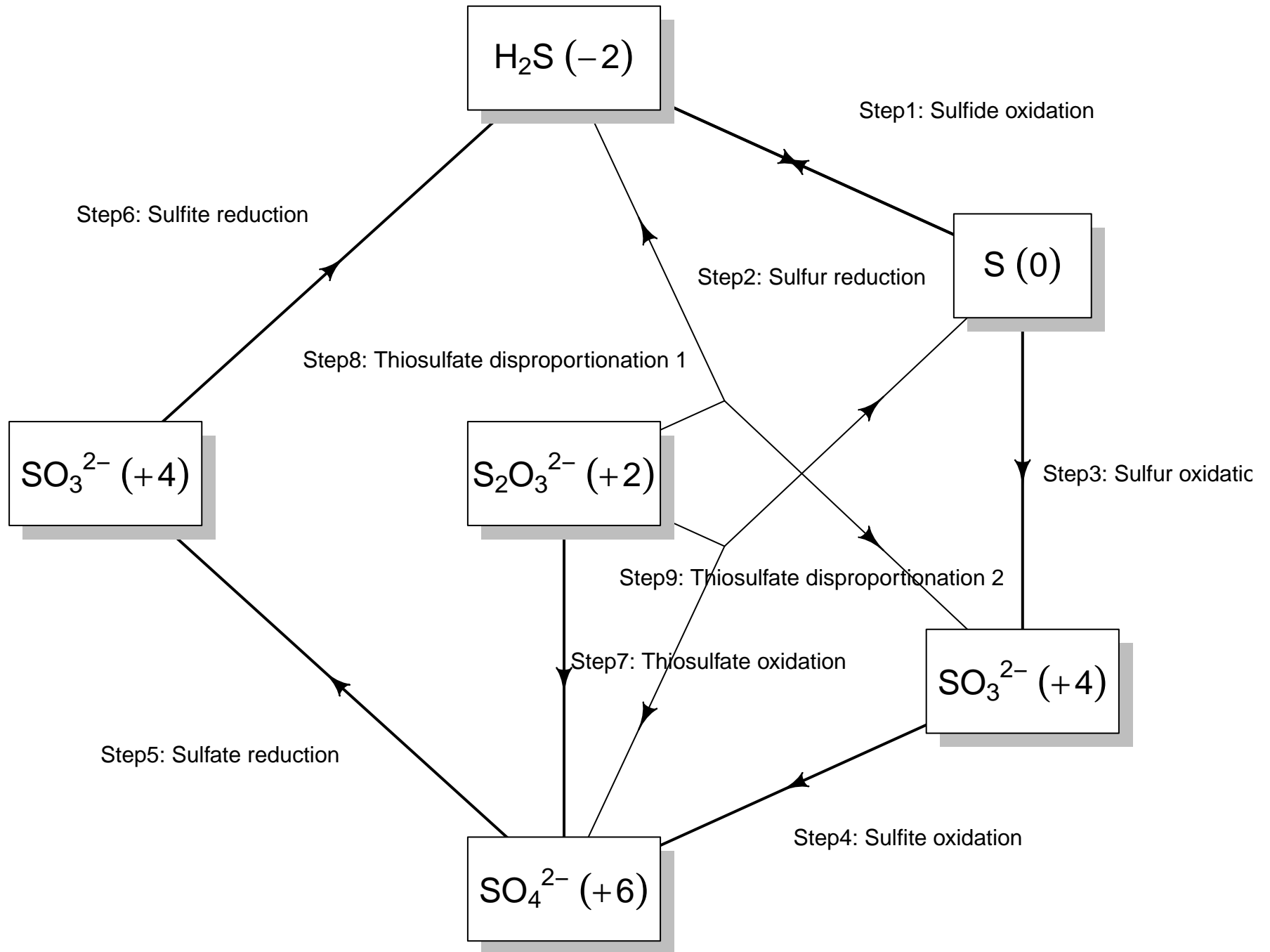

Supplement: Supplementary file 2 — Recovered nutrient cycles including Carbon, Nitrogen, and Sulphur cycles for all the metagenomic assembled genomes given as PDF images, and identifiable through bin numbers. [file 13104_2025_7441_MOESM2_ESM.zip › Supplementary Data2/bin.75.draw_sulfur_cycle_single.pdf]

## Sulfur Cycle: bin.148

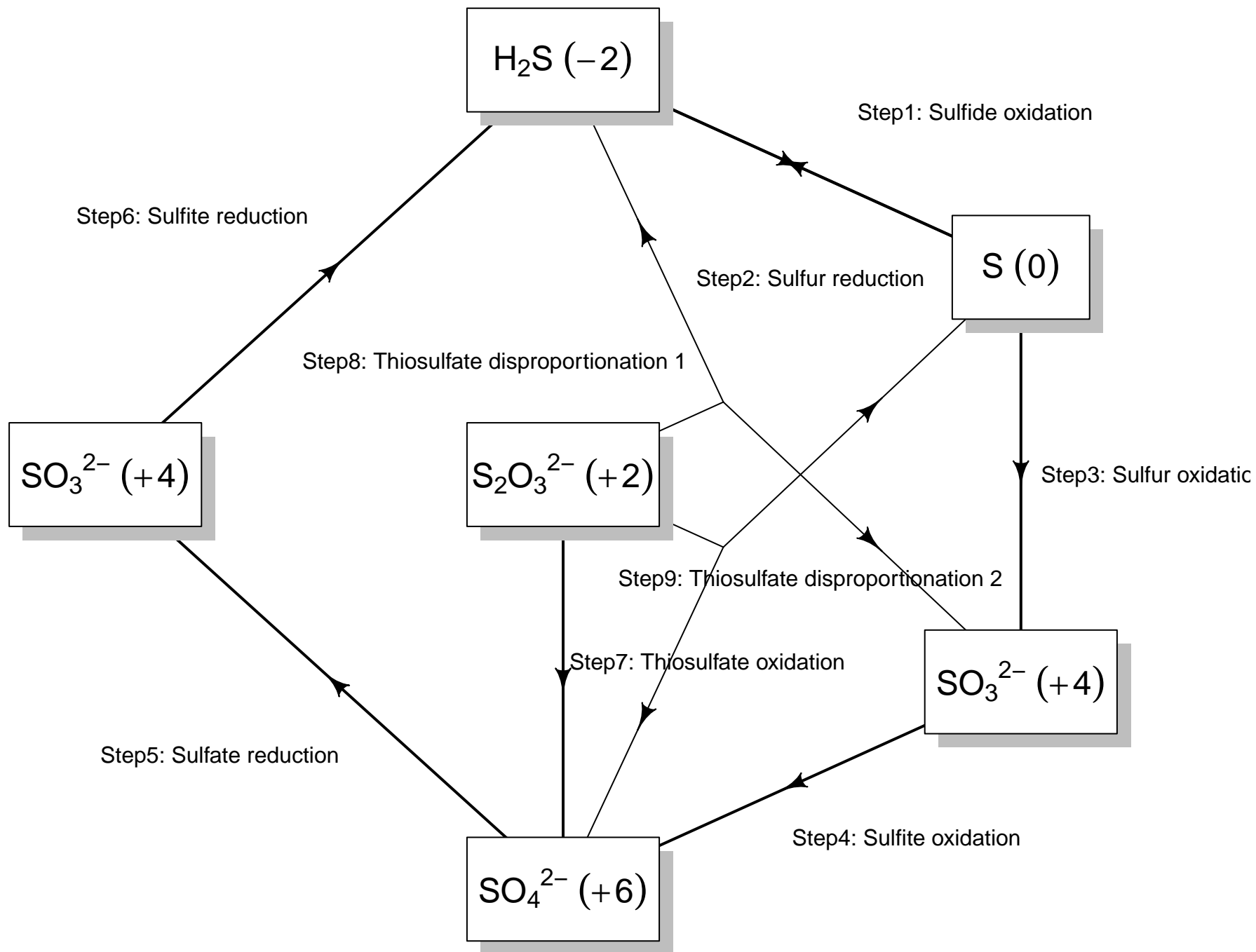

Supplement: Supplementary file 2 — Recovered nutrient cycles including Carbon, Nitrogen, and Sulphur cycles for all the metagenomic assembled genomes given as PDF images, and identifiable through bin numbers. [file 13104_2025_7441_MOESM2_ESM.zip › Supplementary Data2/bin.148.draw_sulfur_cycle_single.pdf]

# Carbon Cycle: bin.58

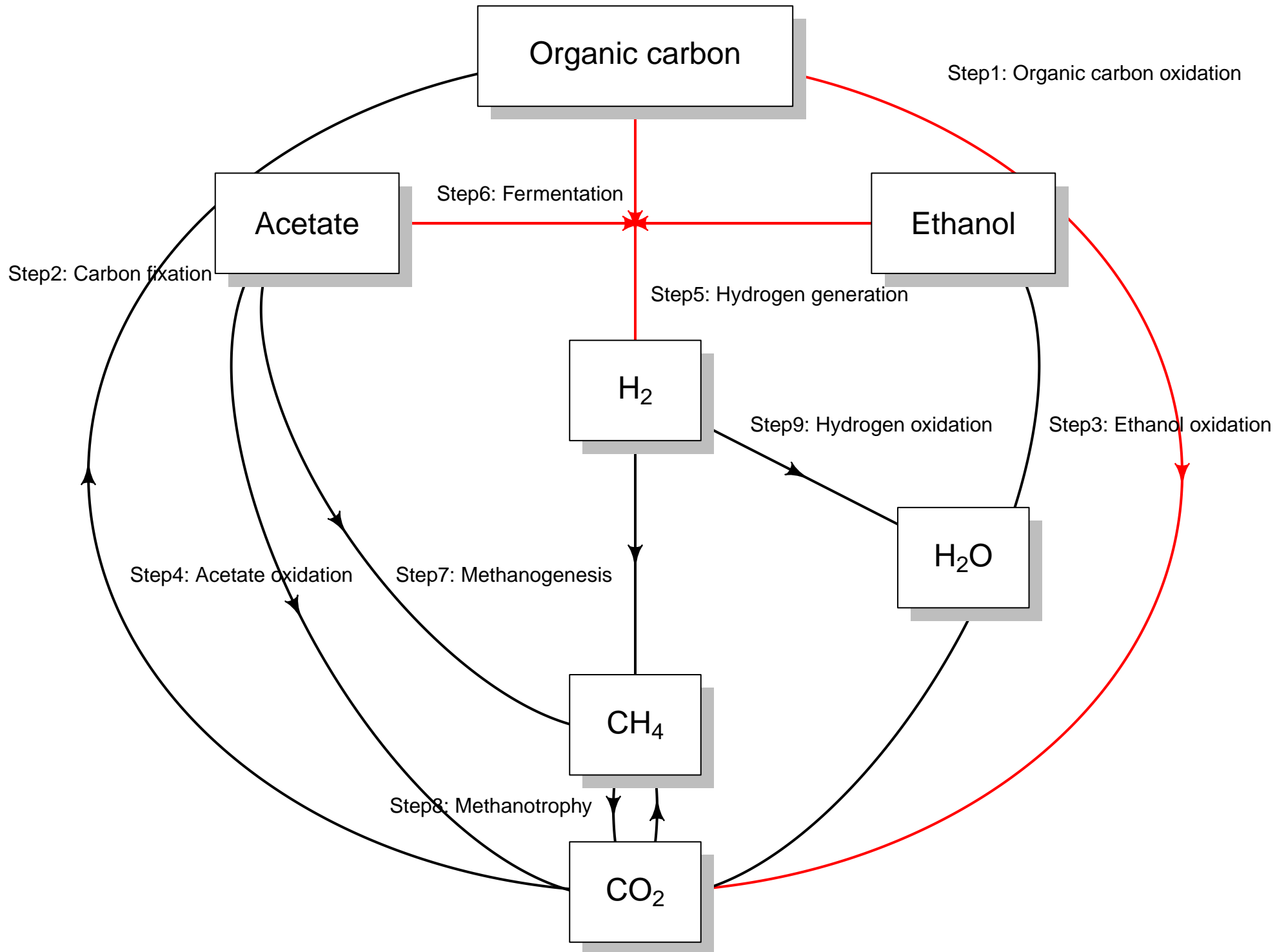

Supplement: Supplementary file 2 — Recovered nutrient cycles including Carbon, Nitrogen, and Sulphur cycles for all the metagenomic assembled genomes given as PDF images, and identifiable through bin numbers. [file 13104_2025_7441_MOESM2_ESM.zip › Supplementary Data2/bin.58.draw_carbon_cycle_single.pdf]

## Nitrogen Cycle: bin.45

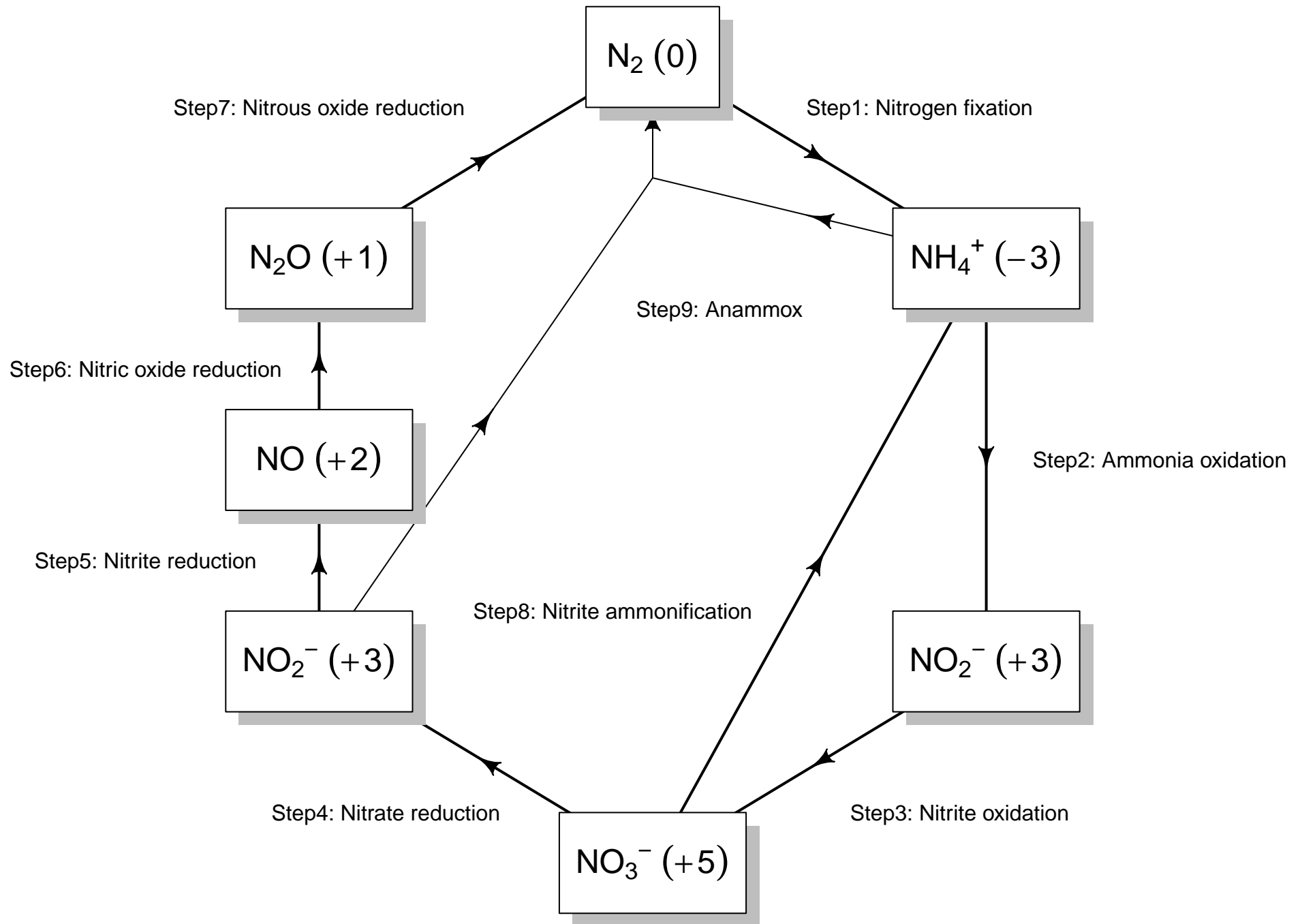

Supplement: Supplementary file 2 — Recovered nutrient cycles including Carbon, Nitrogen, and Sulphur cycles for all the metagenomic assembled genomes given as PDF images, and identifiable through bin numbers. [file 13104_2025_7441_MOESM2_ESM.zip › Supplementary Data2/bin.45.draw_nitrogen_cycle_single.pdf]

# Nitrogen Cycle: bin.101

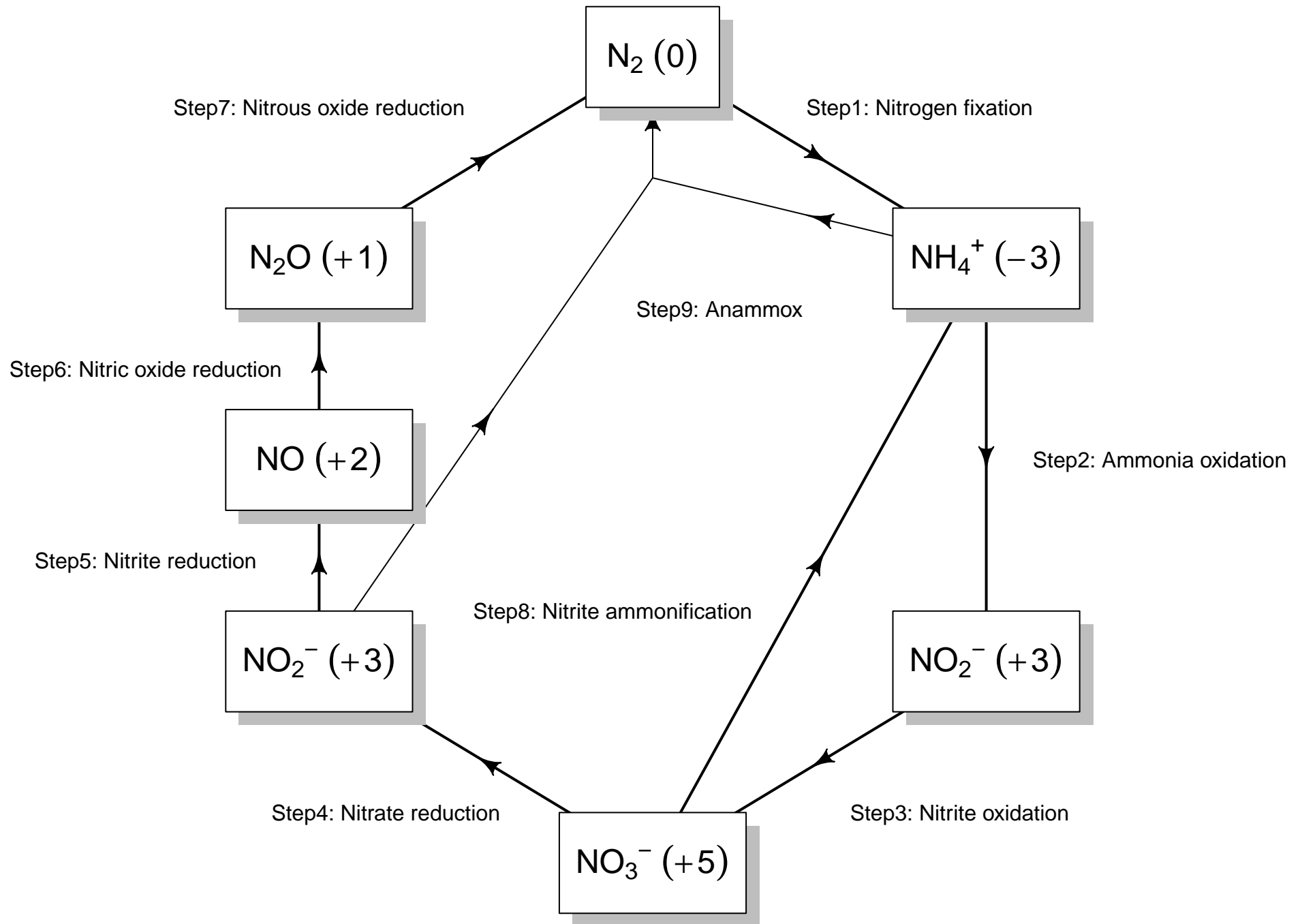

Supplement: Supplementary file 2 — Recovered nutrient cycles including Carbon, Nitrogen, and Sulphur cycles for all the metagenomic assembled genomes given as PDF images, and identifiable through bin numbers. [file 13104_2025_7441_MOESM2_ESM.zip › Supplementary Data2/bin.101.draw_nitrogen_cycle_single.pdf]

## Sulfur Cycle: bin.146

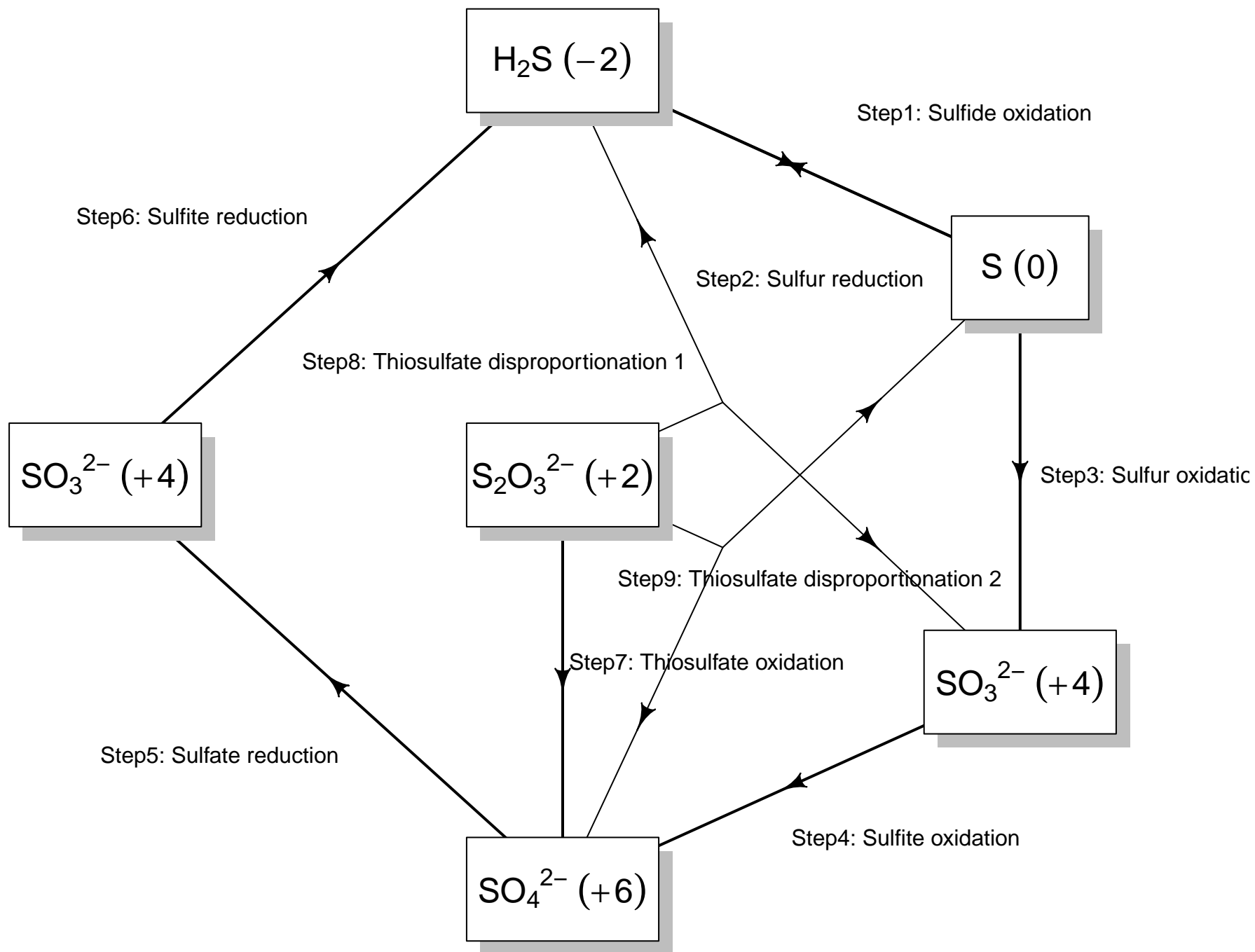

Supplement: Supplementary file 2 — Recovered nutrient cycles including Carbon, Nitrogen, and Sulphur cycles for all the metagenomic assembled genomes given as PDF images, and identifiable through bin numbers. [file 13104_2025_7441_MOESM2_ESM.zip › Supplementary Data2/bin.146.draw_sulfur_cycle_single.pdf]

# Carbon Cycle: bin.56

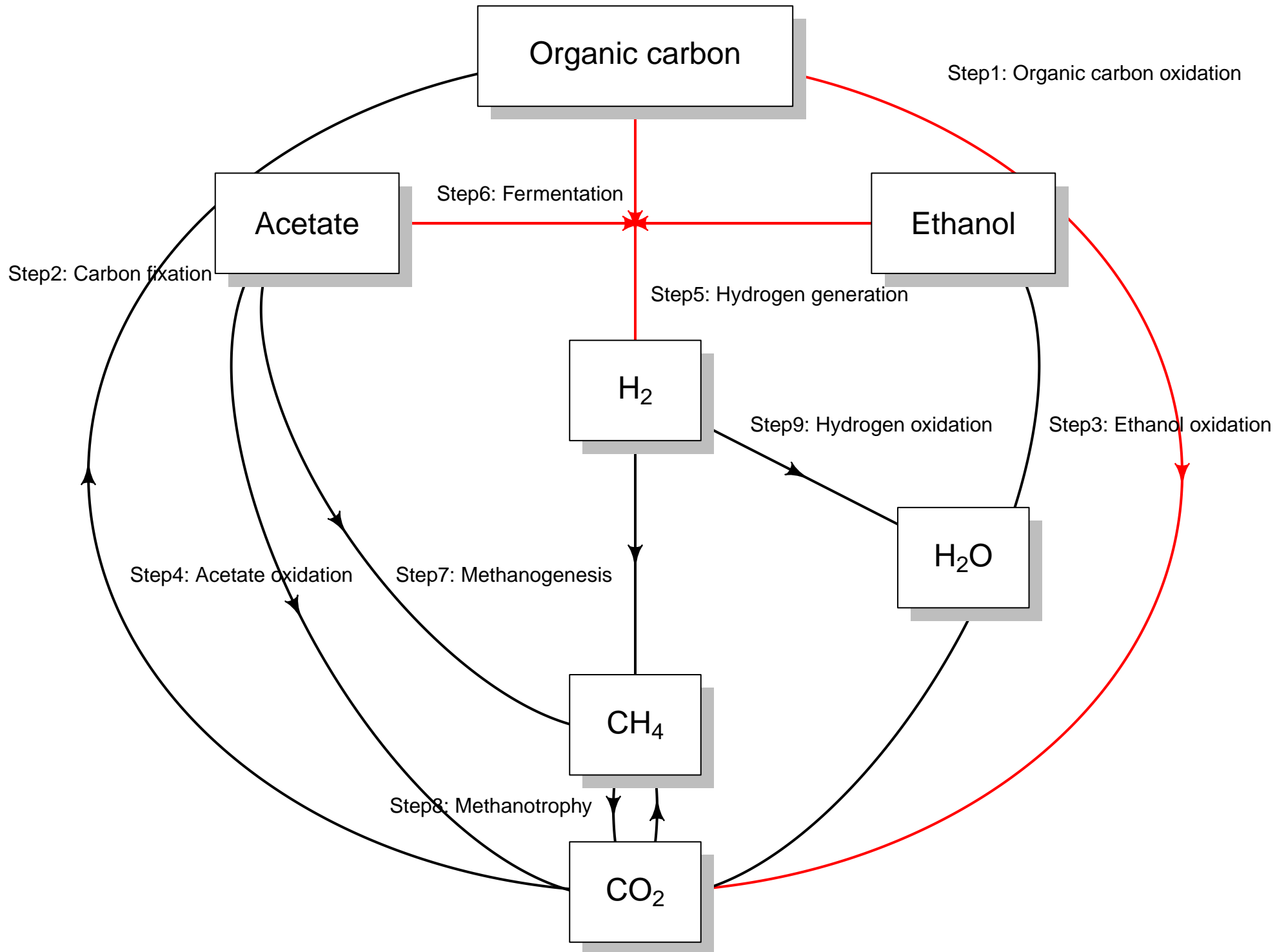

Supplement: Supplementary file 2 — Recovered nutrient cycles including Carbon, Nitrogen, and Sulphur cycles for all the metagenomic assembled genomes given as PDF images, and identifiable through bin numbers. [file 13104_2025_7441_MOESM2_ESM.zip › Supplementary Data2/bin.56.draw_carbon_cycle_single.pdf]

# Carbon Cycle: bin.141

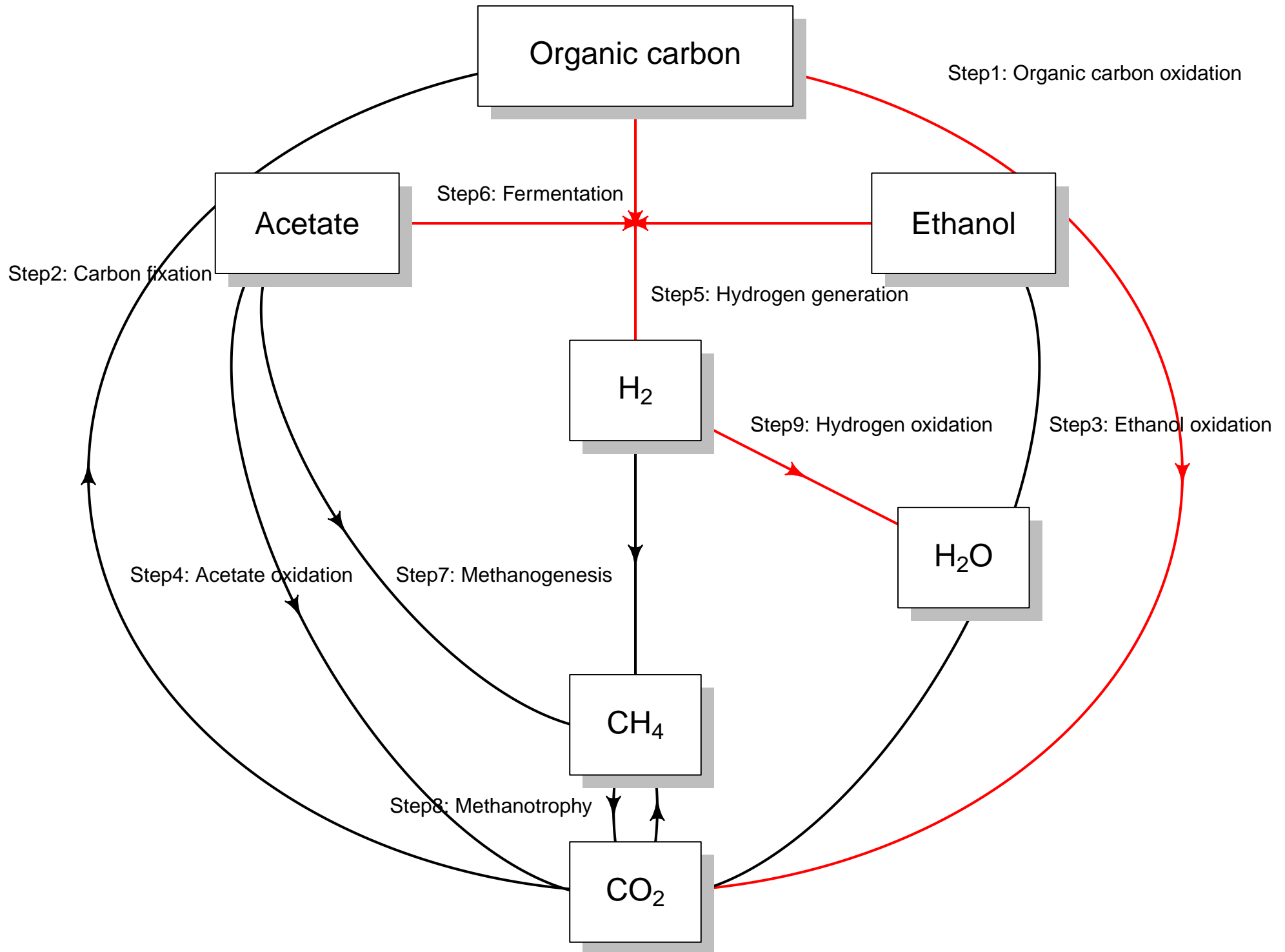

Supplement: Supplementary file 2 — Recovered nutrient cycles including Carbon, Nitrogen, and Sulphur cycles for all the metagenomic assembled genomes given as PDF images, and identifiable through bin numbers. [file 13104_2025_7441_MOESM2_ESM.zip › Supplementary Data2/bin.141.draw_carbon_cycle_single.pdf]

## Sulfur Cycle: bin.51

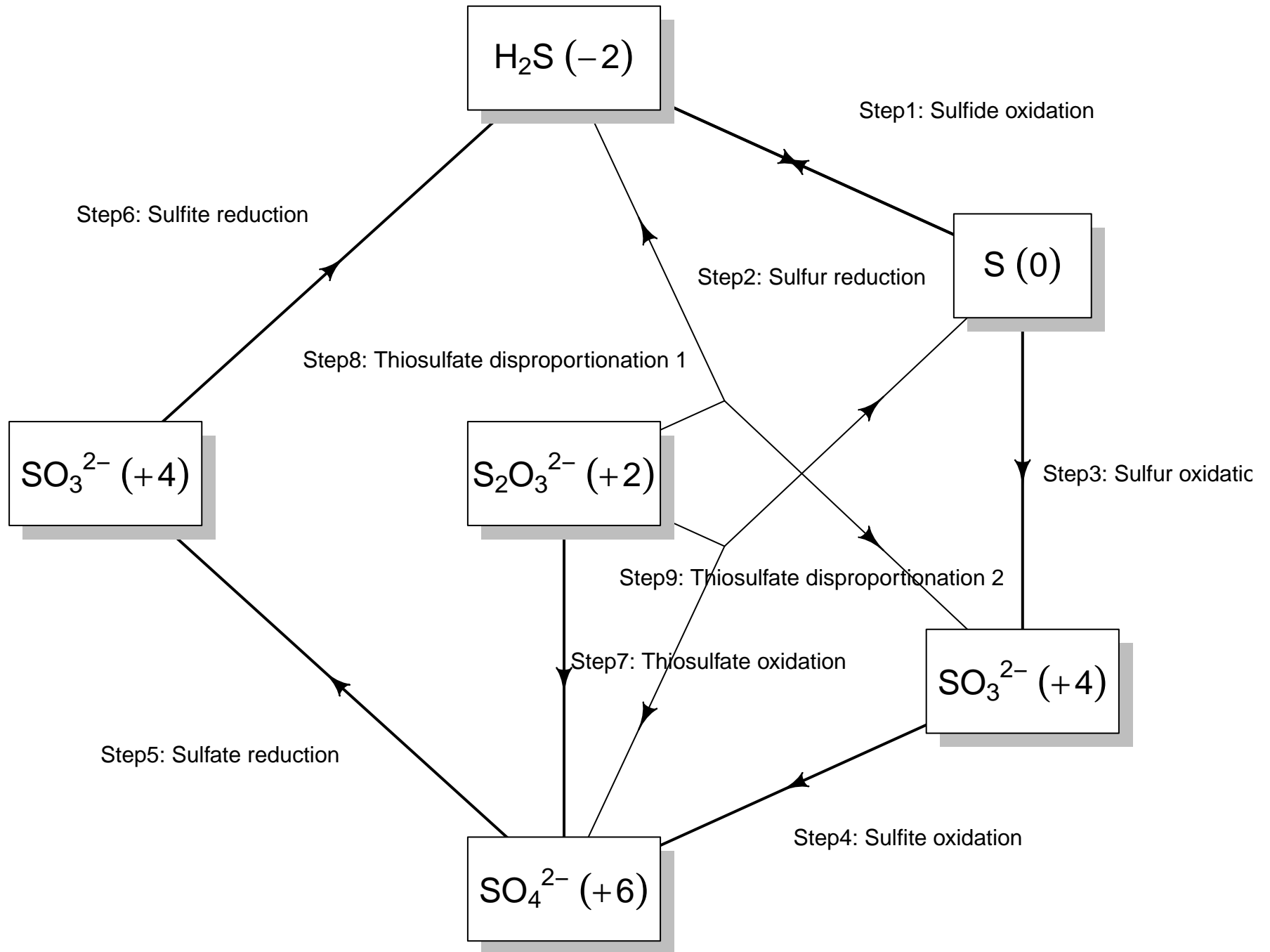

Supplement: Supplementary file 2 — Recovered nutrient cycles including Carbon, Nitrogen, and Sulphur cycles for all the metagenomic assembled genomes given as PDF images, and identifiable through bin numbers. [file 13104_2025_7441_MOESM2_ESM.zip › Supplementary Data2/bin.51.draw_sulfur_cycle_single.pdf]

## Sulfur Cycle: bin.44

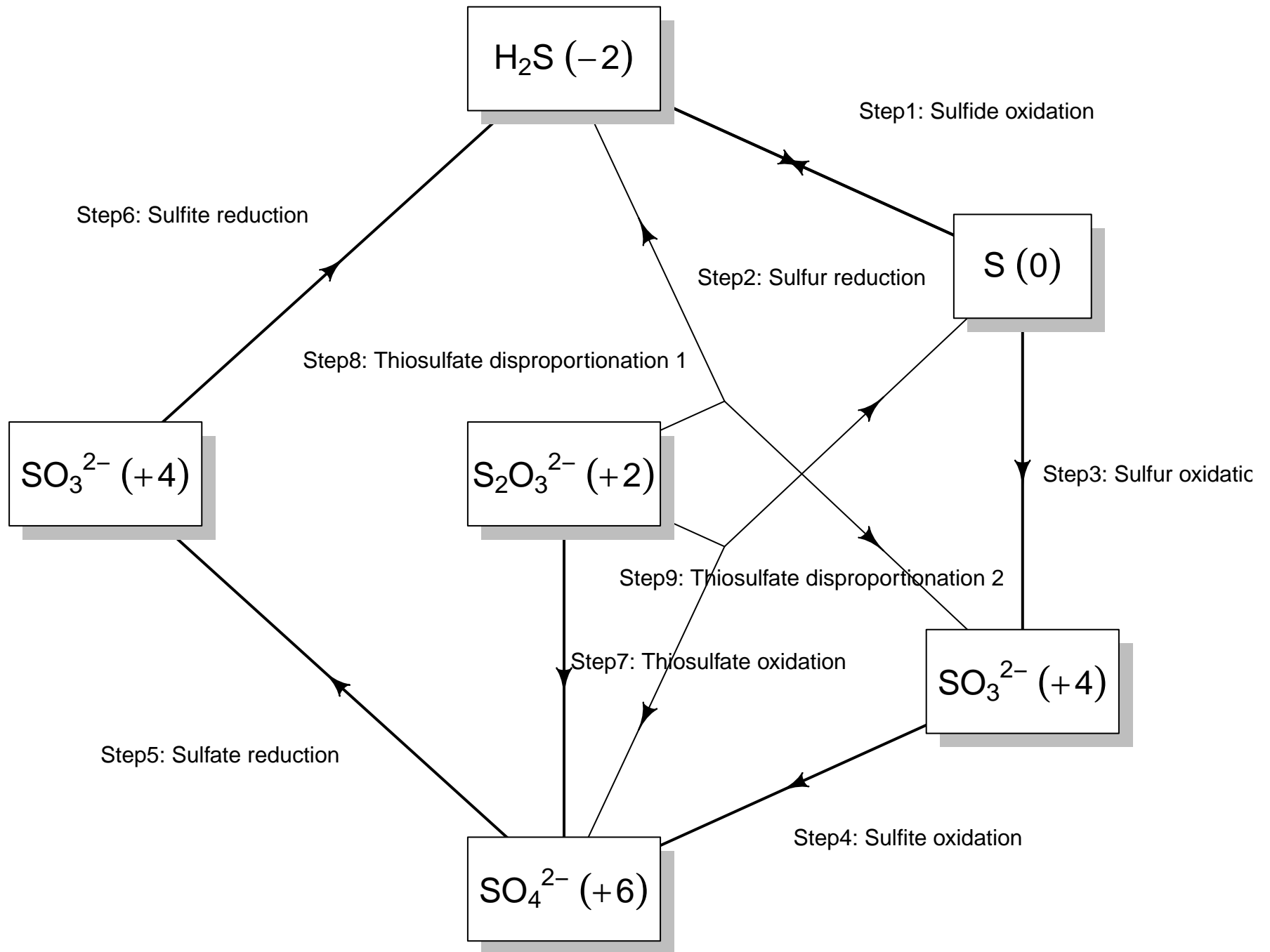

Supplement: Supplementary file 2 — Recovered nutrient cycles including Carbon, Nitrogen, and Sulphur cycles for all the metagenomic assembled genomes given as PDF images, and identifiable through bin numbers. [file 13104_2025_7441_MOESM2_ESM.zip › Supplementary Data2/bin.44.draw_sulfur_cycle_single.pdf]

# Nitrogen Cycle: bin.107

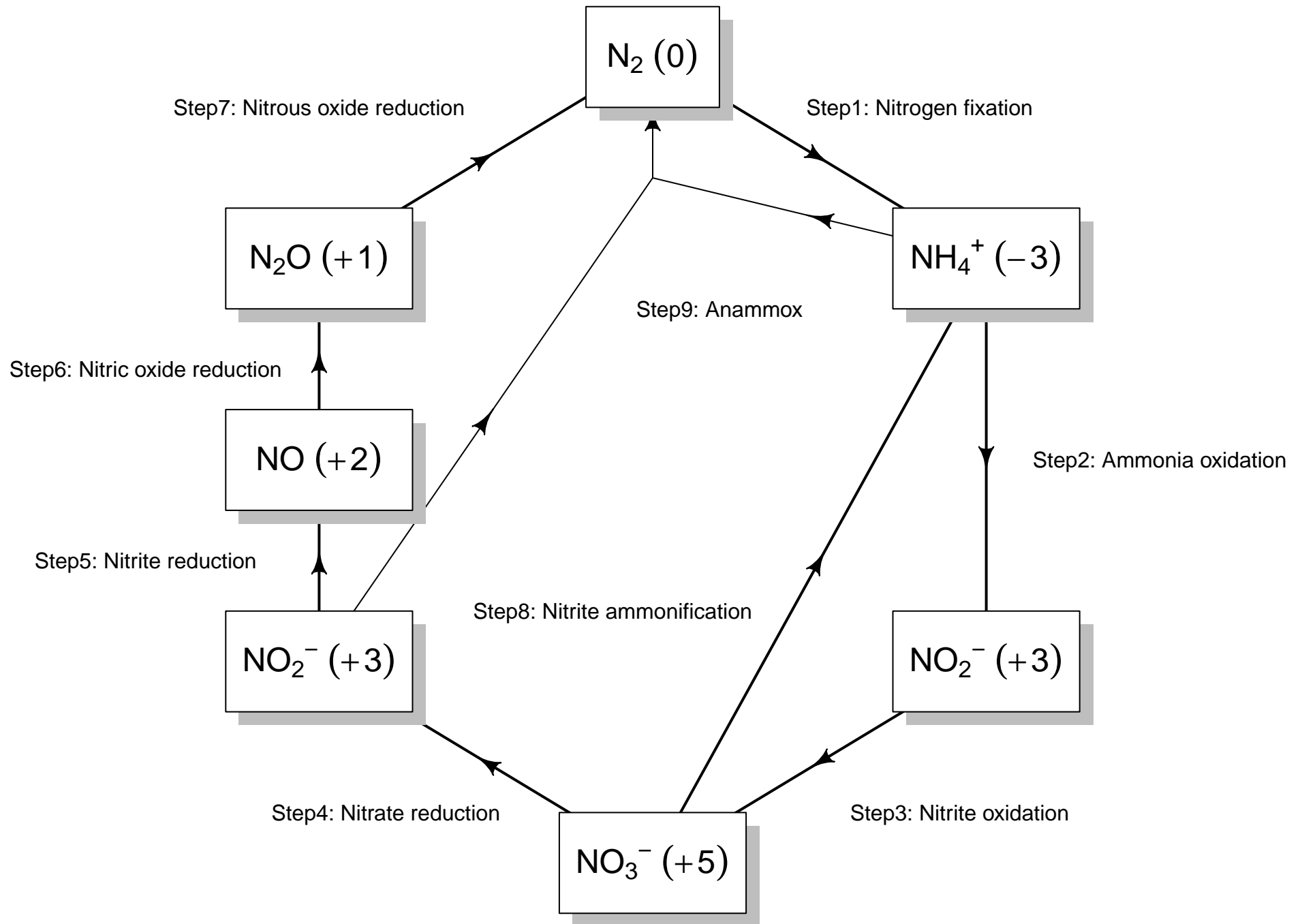

Supplement: Supplementary file 2 — Recovered nutrient cycles including Carbon, Nitrogen, and Sulphur cycles for all the metagenomic assembled genomes given as PDF images, and identifiable through bin numbers. [file 13104_2025_7441_MOESM2_ESM.zip › Supplementary Data2/bin.107.draw_nitrogen_cycle_single.pdf]

# Carbon Cycle: bin.154

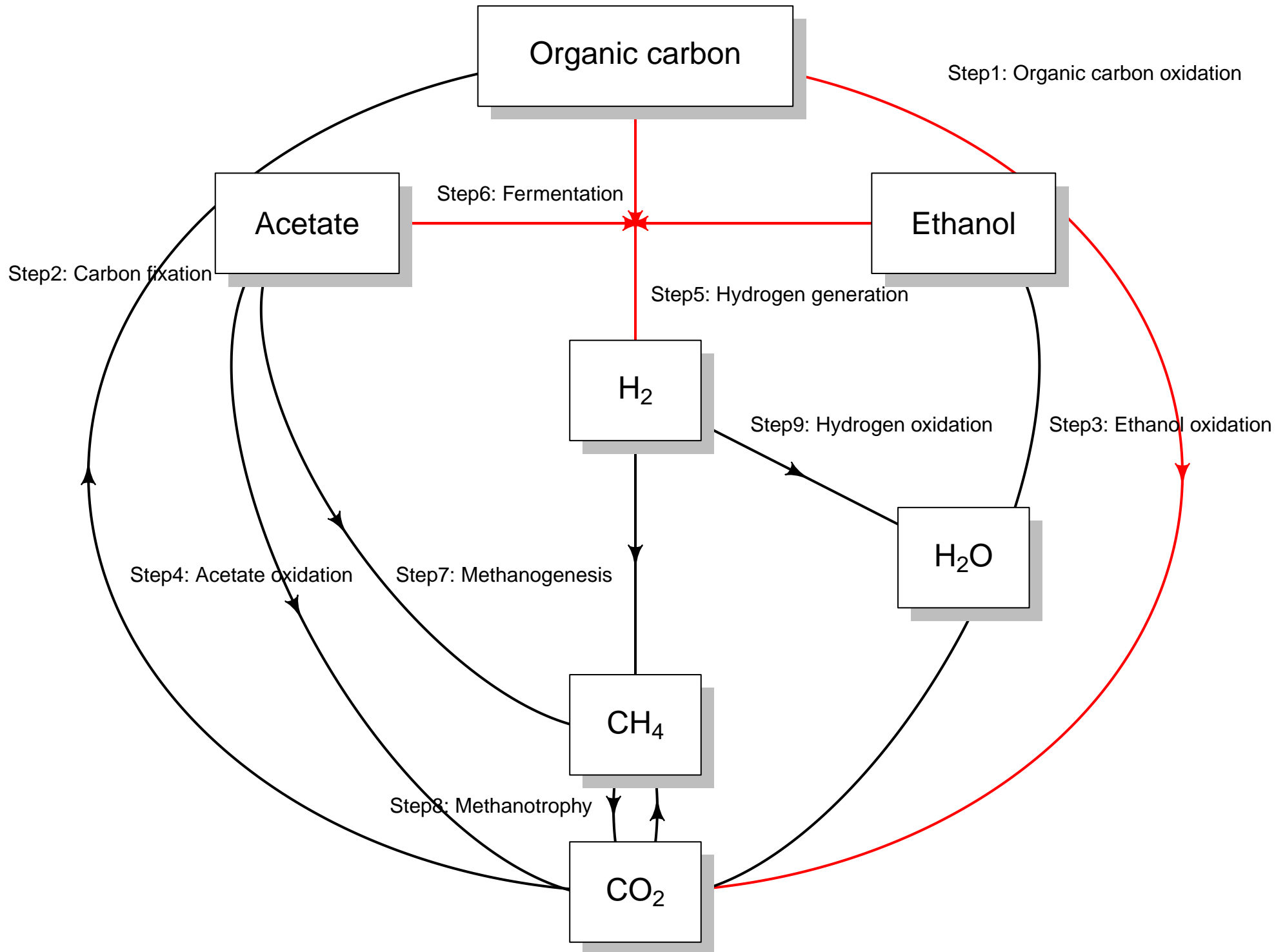

Supplement: Supplementary file 2 — Recovered nutrient cycles including Carbon, Nitrogen, and Sulphur cycles for all the metagenomic assembled genomes given as PDF images, and identifiable through bin numbers. [file 13104_2025_7441_MOESM2_ESM.zip › Supplementary Data2/bin.154.draw_carbon_cycle_single.pdf]

# Nitrogen Cycle: bin.148

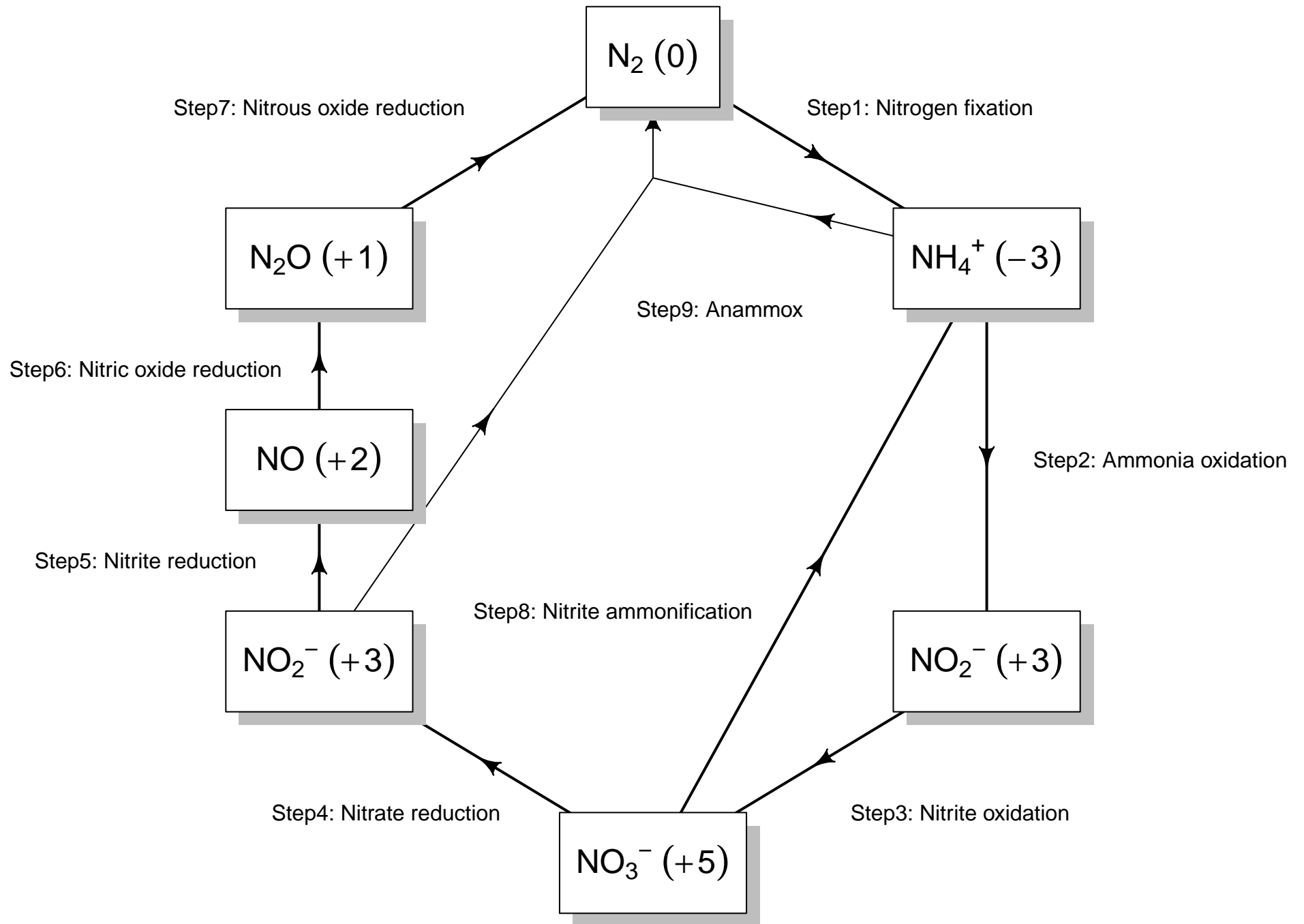

Supplement: Supplementary file 2 — Recovered nutrient cycles including Carbon, Nitrogen, and Sulphur cycles for all the metagenomic assembled genomes given as PDF images, and identifiable through bin numbers. [file 13104_2025_7441_MOESM2_ESM.zip › Supplementary Data2/bin.148.draw_nitrogen_cycle_single.pdf]

## Nitrogen Cycle: bin.43

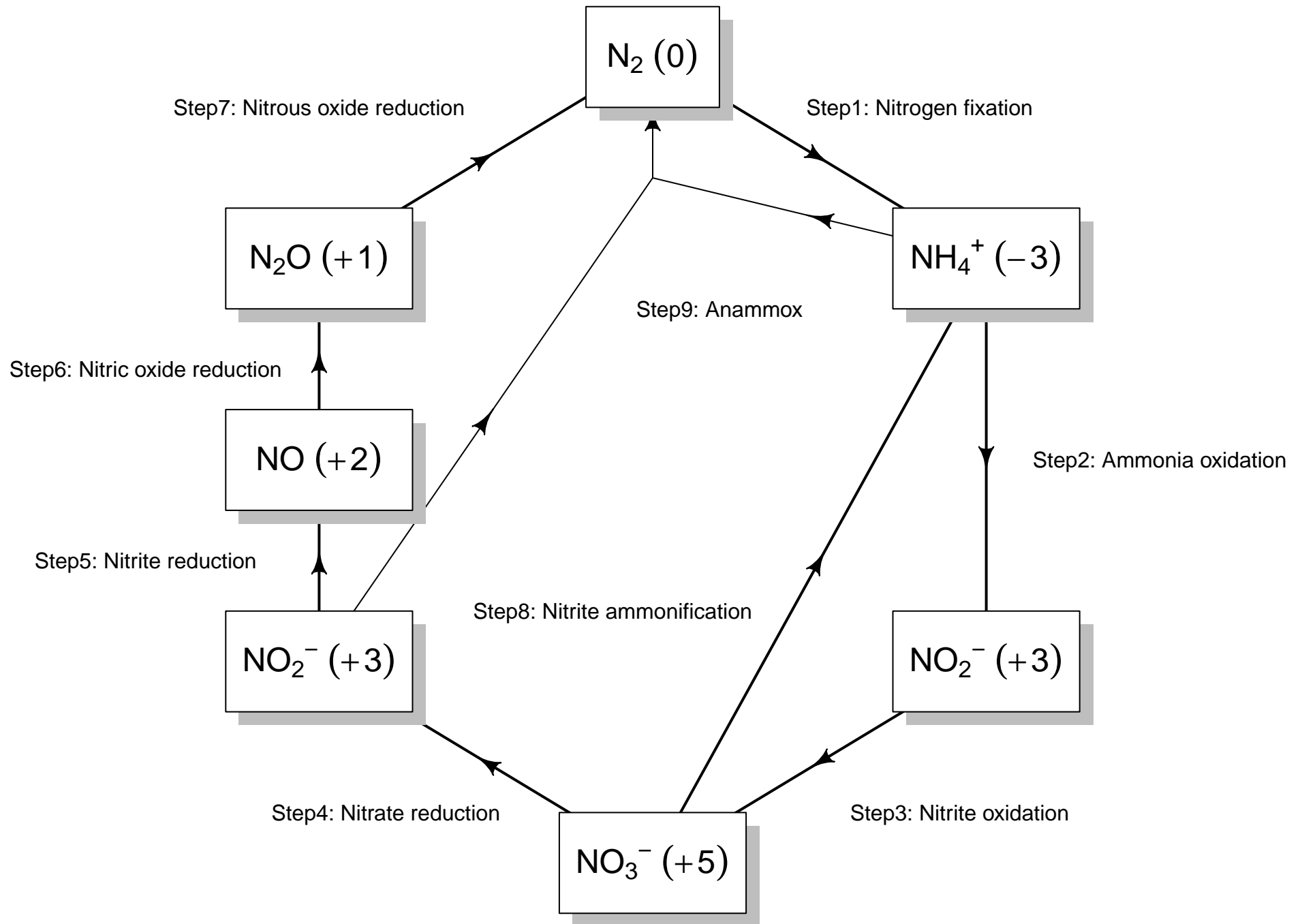

Supplement: Supplementary file 2 — Recovered nutrient cycles including Carbon, Nitrogen, and Sulphur cycles for all the metagenomic assembled genomes given as PDF images, and identifiable through bin numbers. [file 13104_2025_7441_MOESM2_ESM.zip › Supplementary Data2/bin.43.draw_nitrogen_cycle_single.pdf]

## Nitrogen Cycle: bin.50

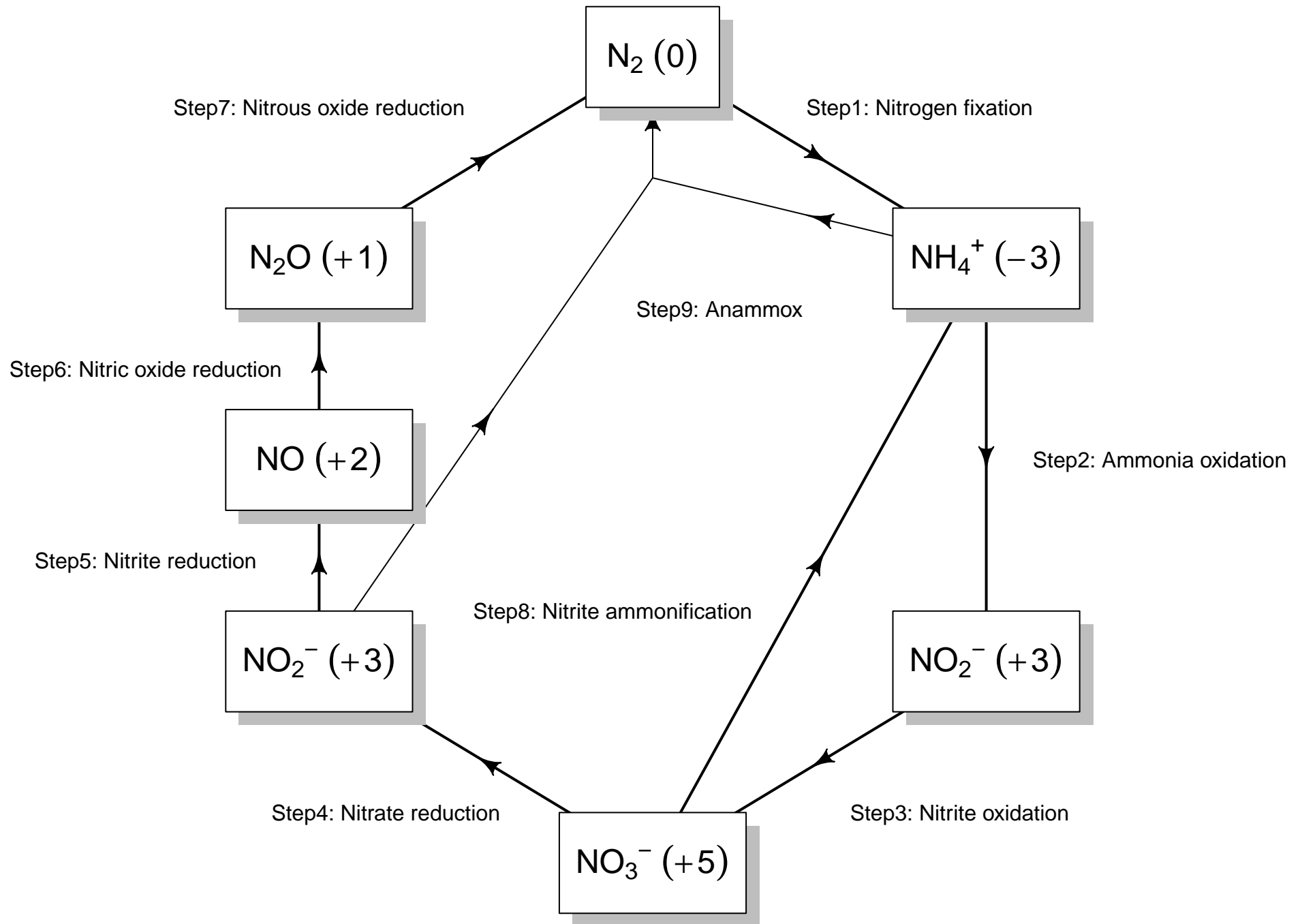

Supplement: Supplementary file 2 — Recovered nutrient cycles including Carbon, Nitrogen, and Sulphur cycles for all the metagenomic assembled genomes given as PDF images, and identifiable through bin numbers. [file 13104_2025_7441_MOESM2_ESM.zip › Supplementary Data2/bin.50.draw_nitrogen_cycle_single.pdf]

# Carbon Cycle: bin.67

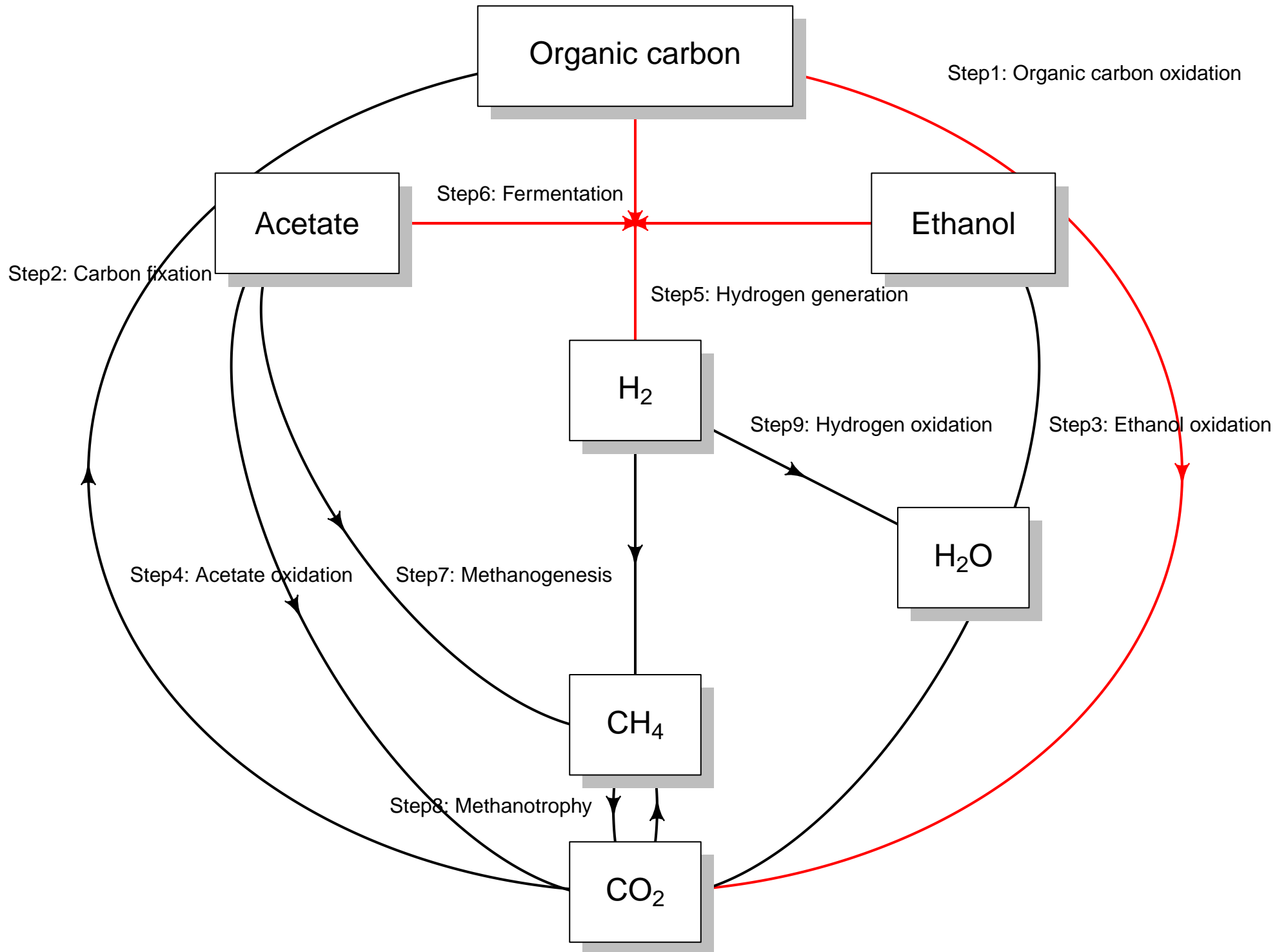

Supplement: Supplementary file 2 — Recovered nutrient cycles including Carbon, Nitrogen, and Sulphur cycles for all the metagenomic assembled genomes given as PDF images, and identifiable through bin numbers. [file 13104_2025_7441_MOESM2_ESM.zip › Supplementary Data2/bin.67.draw_carbon_cycle_single.pdf]

# Carbon Cycle: bin.69

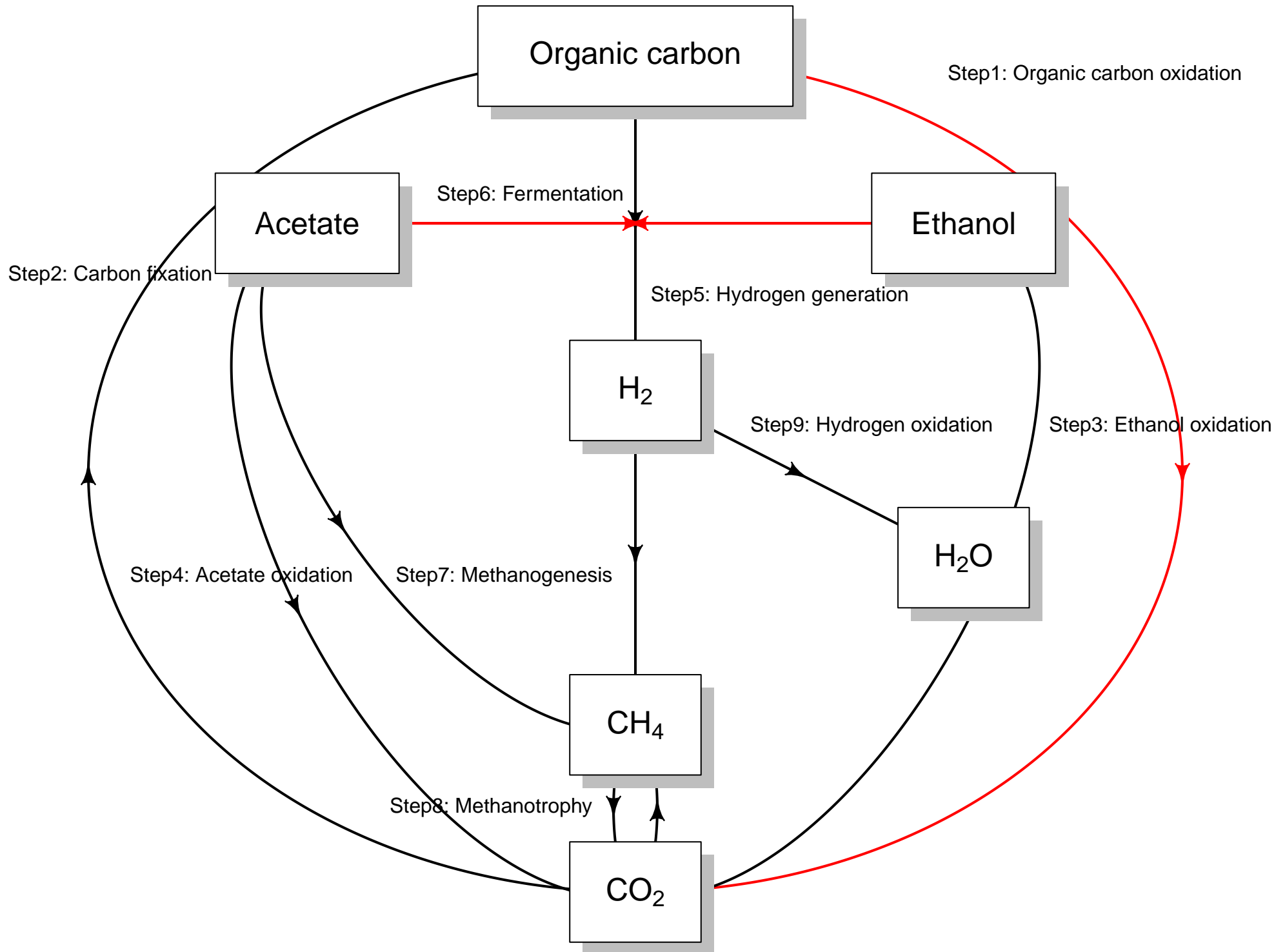

Supplement: Supplementary file 2 — Recovered nutrient cycles including Carbon, Nitrogen, and Sulphur cycles for all the metagenomic assembled genomes given as PDF images, and identifiable through bin numbers. [file 13104_2025_7441_MOESM2_ESM.zip › Supplementary Data2/bin.69.draw_carbon_cycle_single.pdf]

# Nitrogen Cycle: bin.114

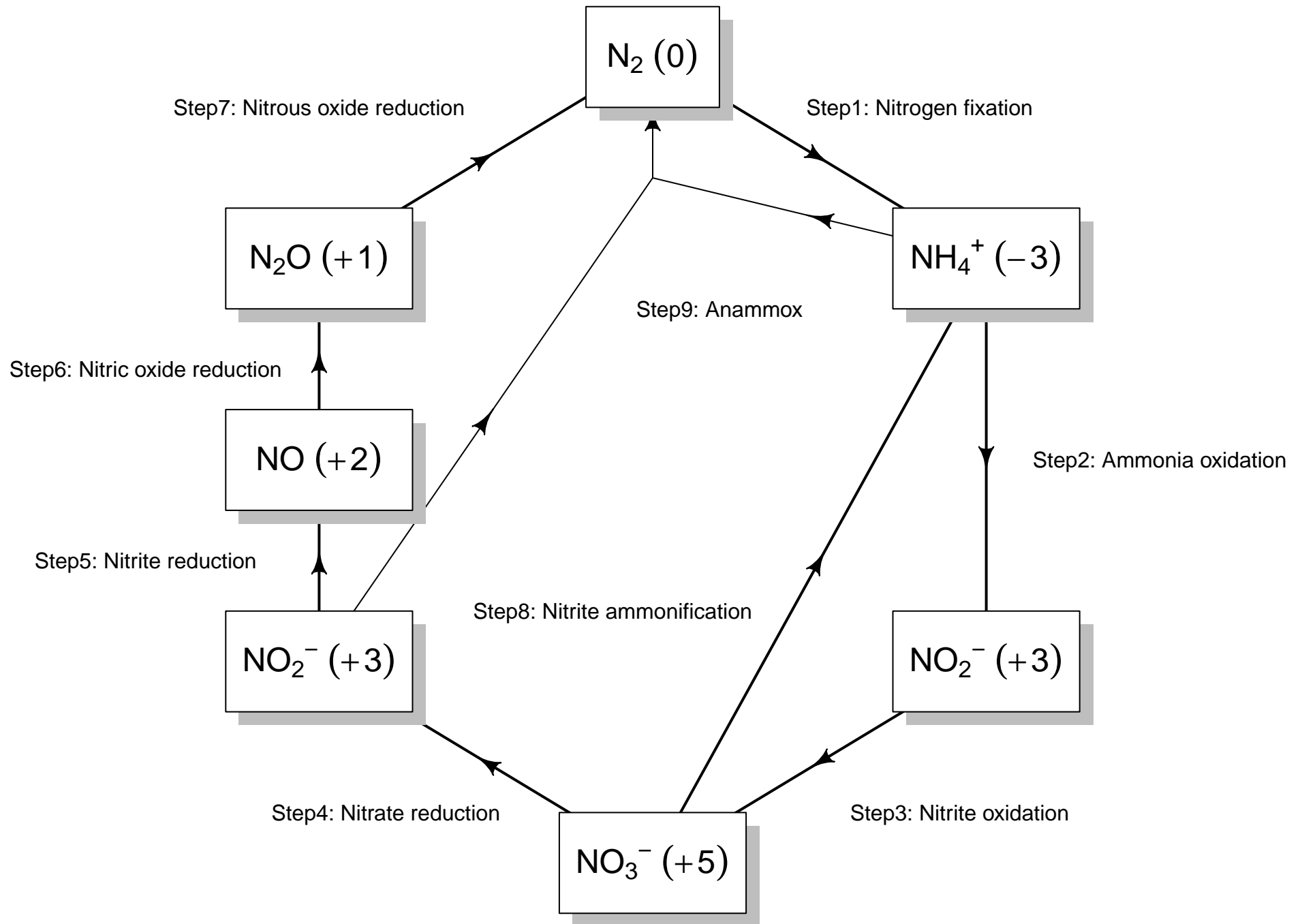

Supplement: Supplementary file 2 — Recovered nutrient cycles including Carbon, Nitrogen, and Sulphur cycles for all the metagenomic assembled genomes given as PDF images, and identifiable through bin numbers. [file 13104_2025_7441_MOESM2_ESM.zip › Supplementary Data2/bin.114.draw_nitrogen_cycle_single.pdf]

## Sulfur Cycle: bin.13

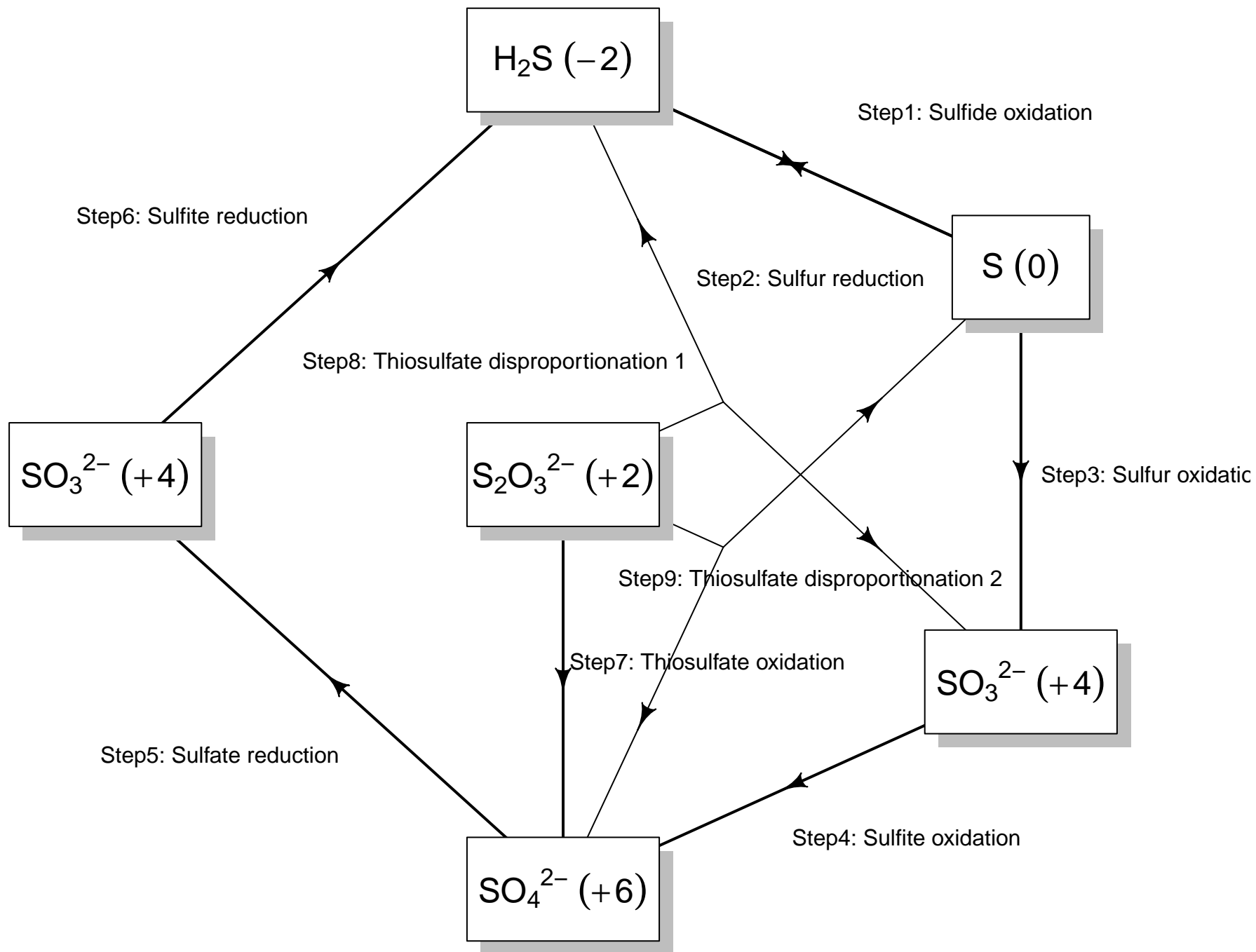

Supplement: Supplementary file 2 — Recovered nutrient cycles including Carbon, Nitrogen, and Sulphur cycles for all the metagenomic assembled genomes given as PDF images, and identifiable through bin numbers. [file 13104_2025_7441_MOESM2_ESM.zip › Supplementary Data2/bin.13.draw_sulfur_cycle_single.pdf]

## Nitrogen Cycle: bin.142

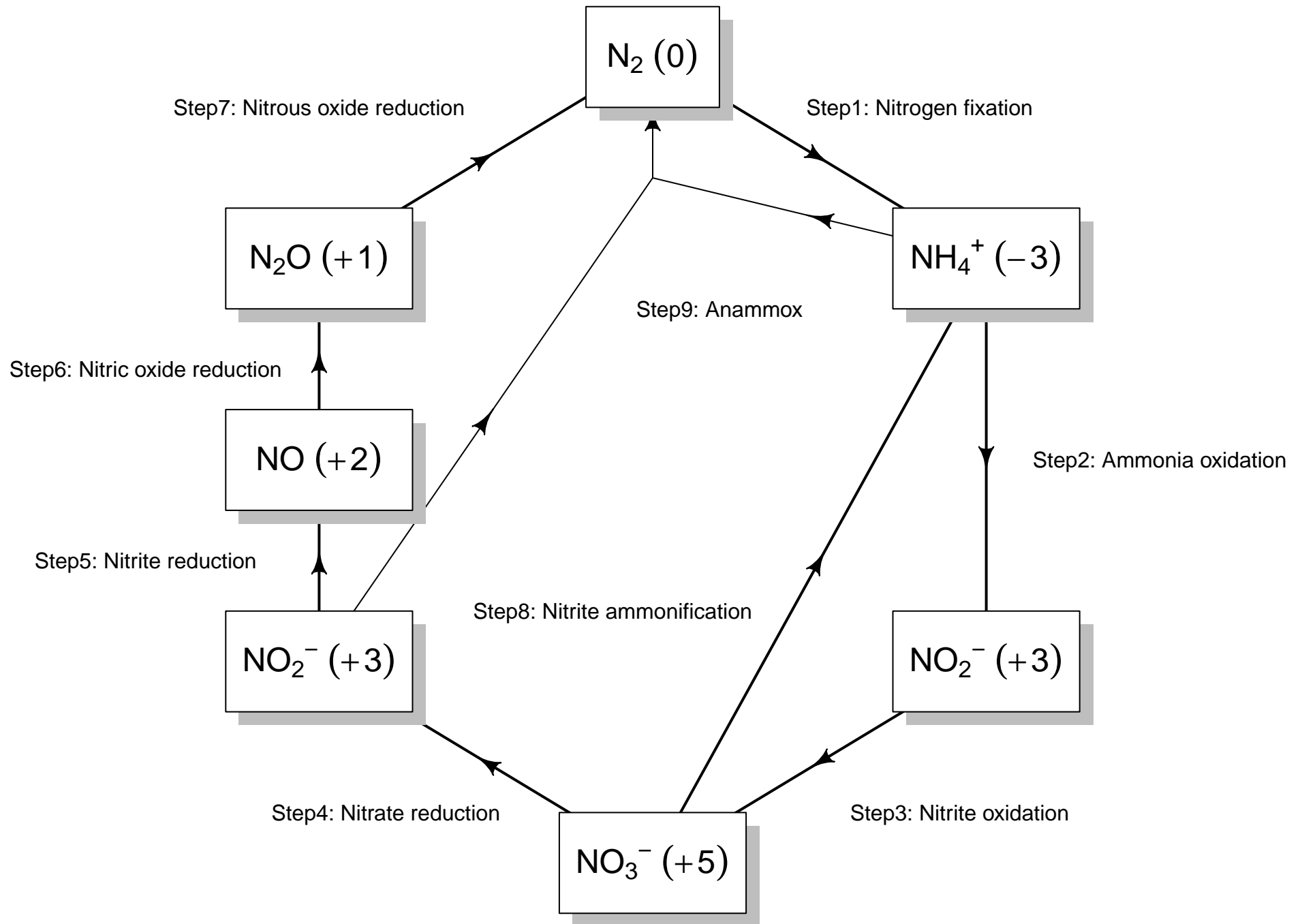

Supplement: Supplementary file 2 — Recovered nutrient cycles including Carbon, Nitrogen, and Sulphur cycles for all the metagenomic assembled genomes given as PDF images, and identifiable through bin numbers. [file 13104_2025_7441_MOESM2_ESM.zip › Supplementary Data2/bin.142.draw_nitrogen_cycle_single.pdf]

# Carbon Cycle: bin.103

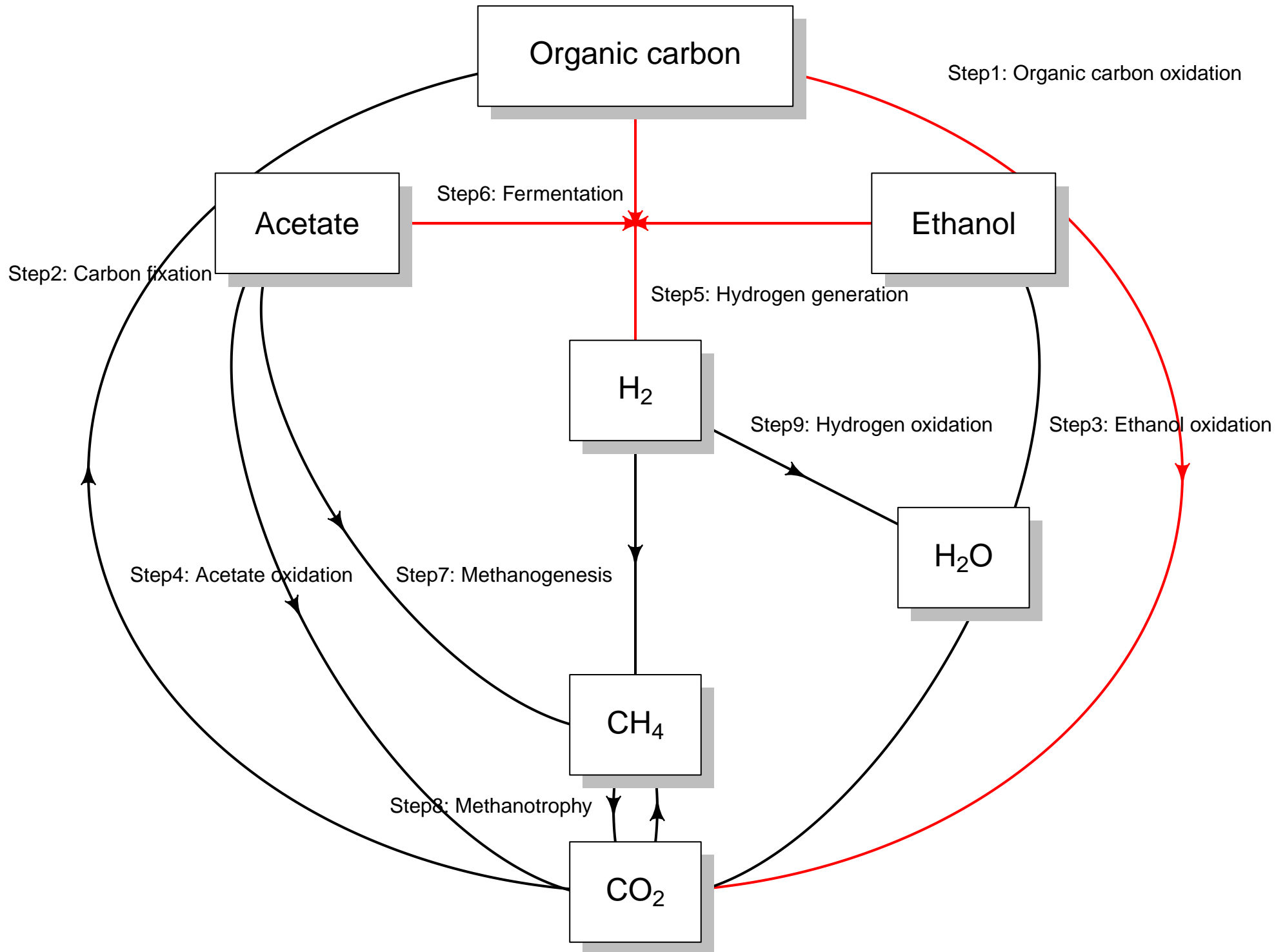

Supplement: Supplementary file 2 — Recovered nutrient cycles including Carbon, Nitrogen, and Sulphur cycles for all the metagenomic assembled genomes given as PDF images, and identifiable through bin numbers. [file 13104_2025_7441_MOESM2_ESM.zip › Supplementary Data2/bin.103.draw_carbon_cycle_single.pdf]

## Sulfur Cycle: bin.85

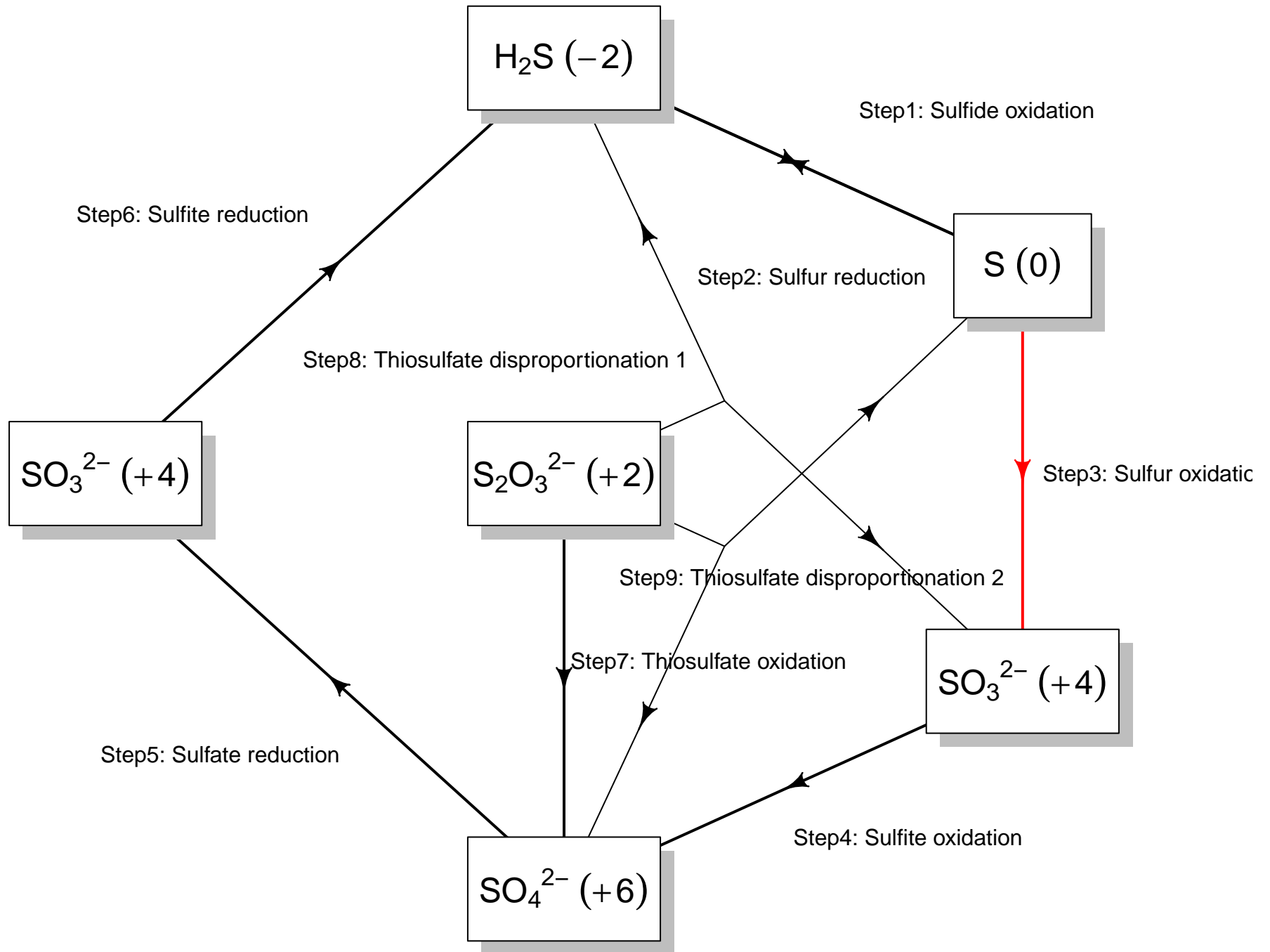

Supplement: Supplementary file 2 — Recovered nutrient cycles including Carbon, Nitrogen, and Sulphur cycles for all the metagenomic assembled genomes given as PDF images, and identifiable through bin numbers. [file 13104_2025_7441_MOESM2_ESM.zip › Supplementary Data2/bin.85.draw_sulfur_cycle_single.pdf]

## Sulfur Cycle: bin.120

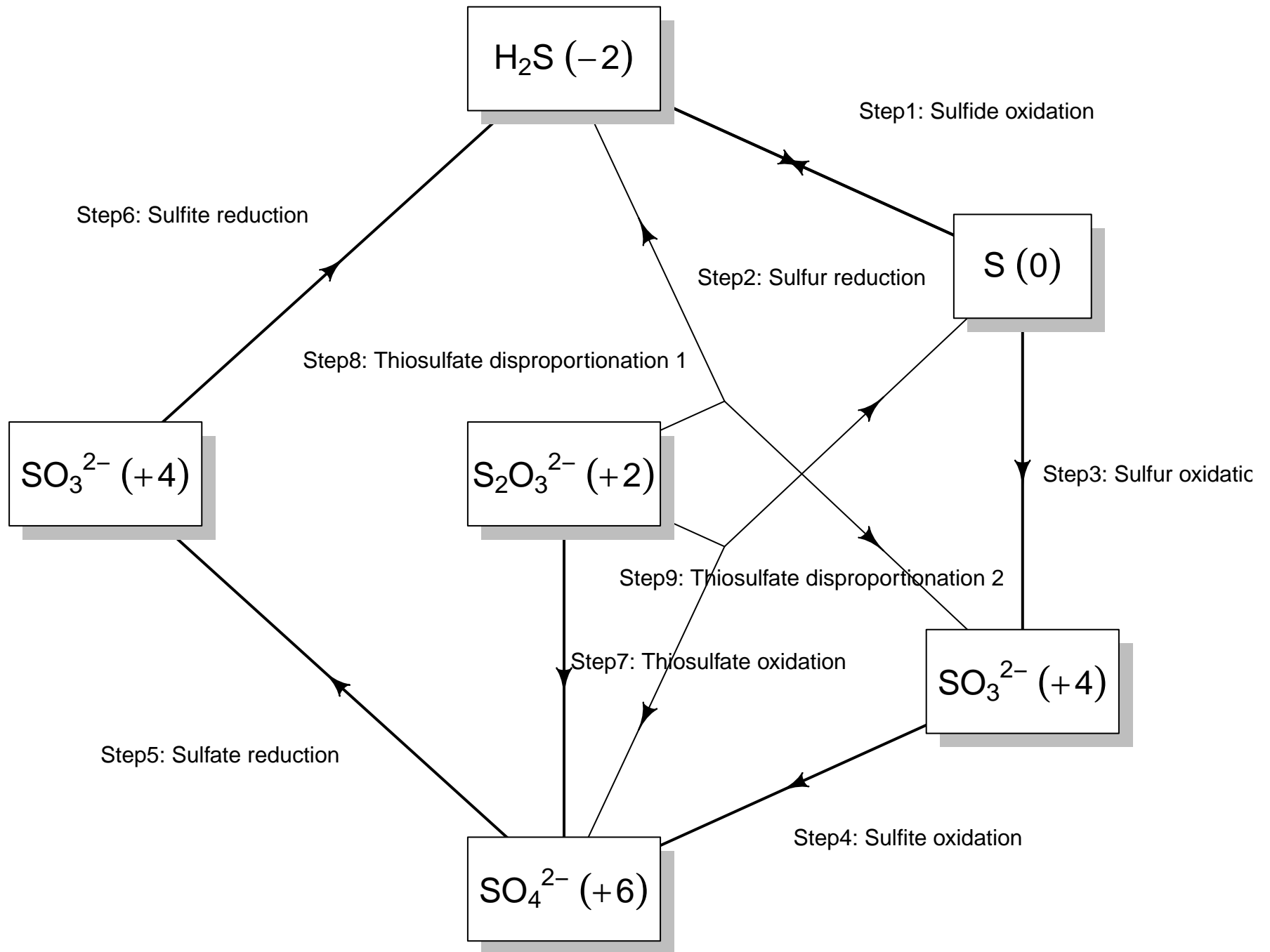

Supplement: Supplementary file 2 — Recovered nutrient cycles including Carbon, Nitrogen, and Sulphur cycles for all the metagenomic assembled genomes given as PDF images, and identifiable through bin numbers. [file 13104_2025_7441_MOESM2_ESM.zip › Supplementary Data2/bin.120.draw_sulfur_cycle_single.pdf]

## Nitrogen Cycle: bin.15

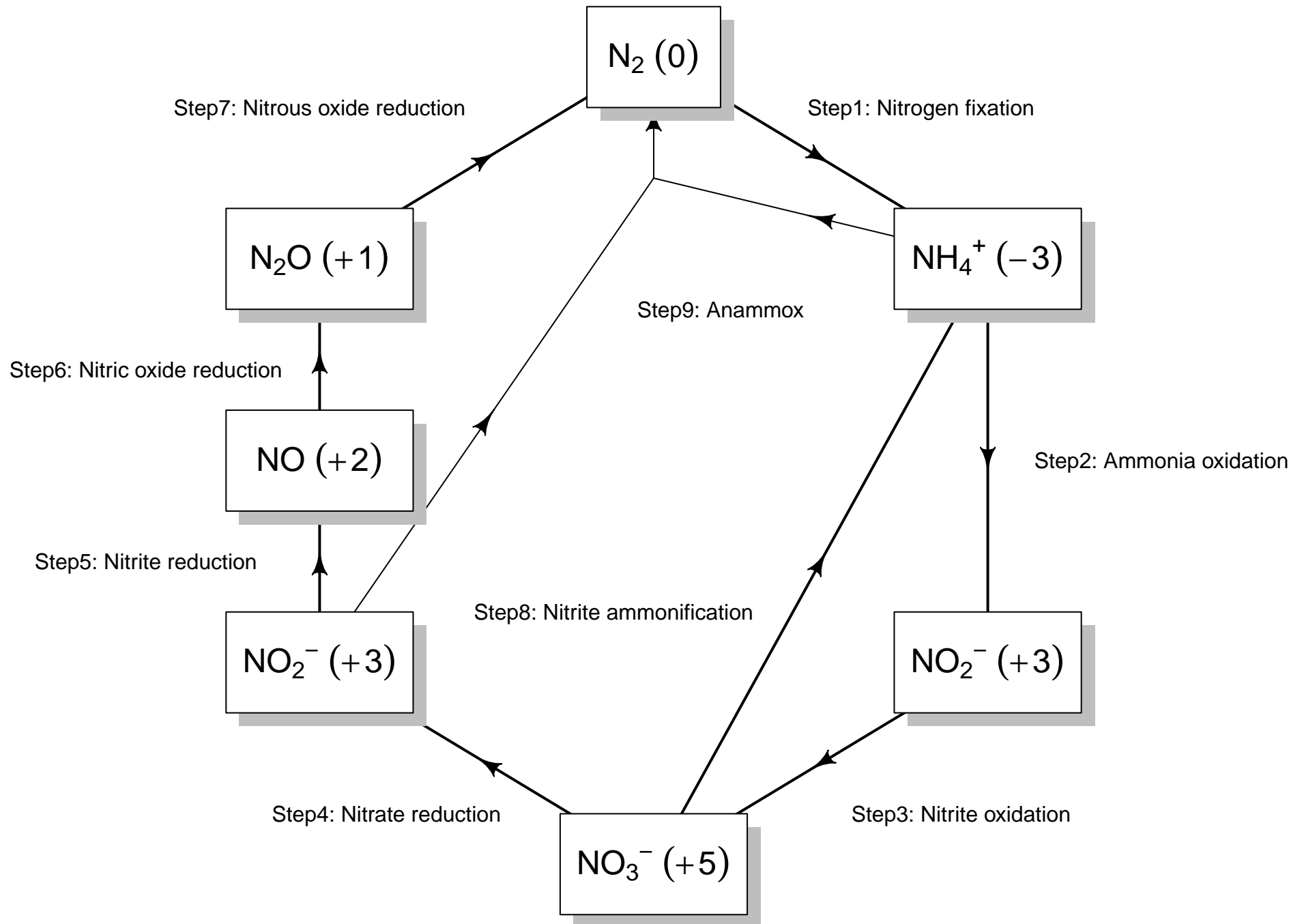

Supplement: Supplementary file 2 — Recovered nutrient cycles including Carbon, Nitrogen, and Sulphur cycles for all the metagenomic assembled genomes given as PDF images, and identifiable through bin numbers. [file 13104_2025_7441_MOESM2_ESM.zip › Supplementary Data2/bin.15.draw_nitrogen_cycle_single.pdf]

# Carbon Cycle: bin.30

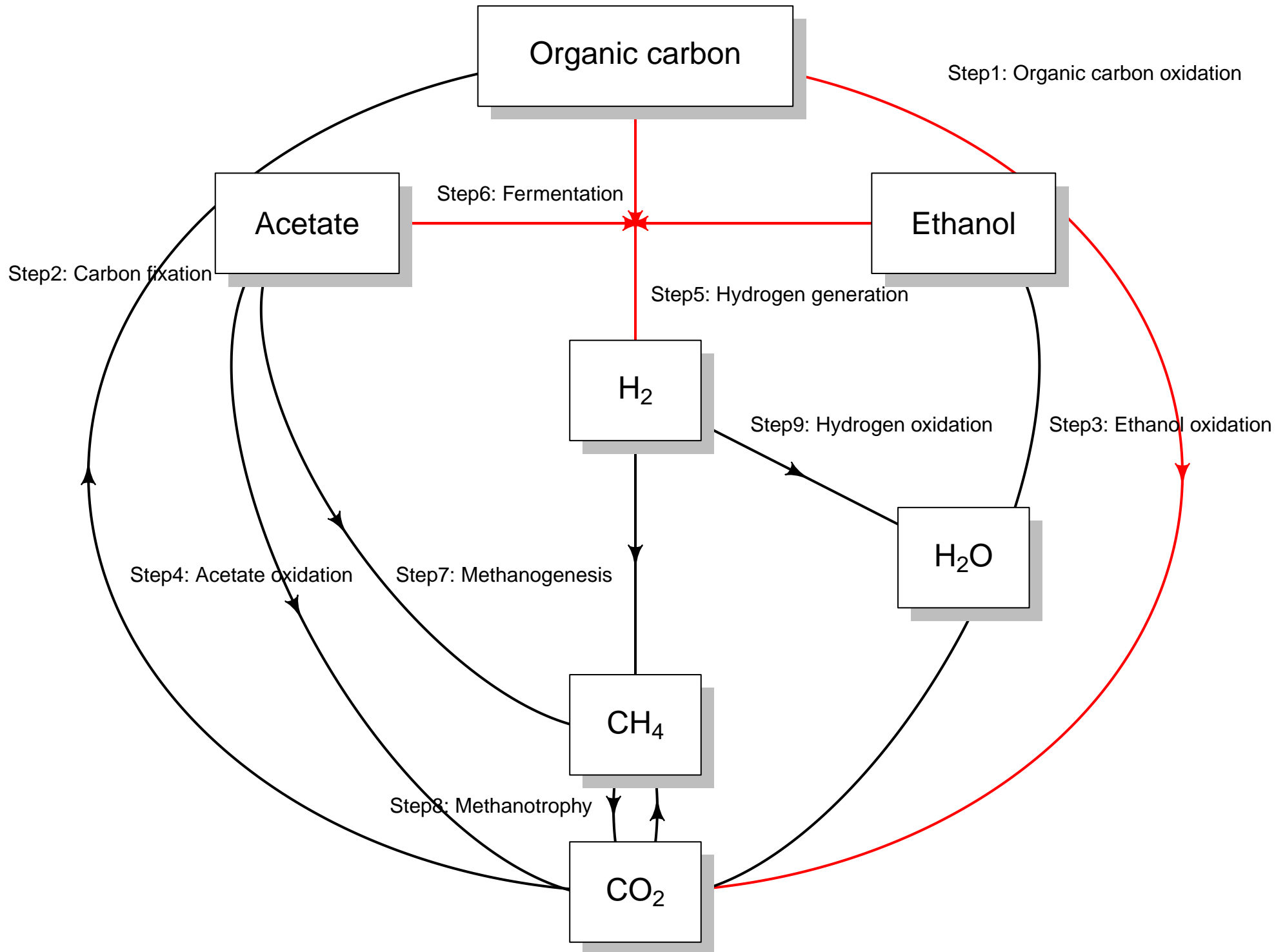

Supplement: Supplementary file 2 — Recovered nutrient cycles including Carbon, Nitrogen, and Sulphur cycles for all the metagenomic assembled genomes given as PDF images, and identifiable through bin numbers. [file 13104_2025_7441_MOESM2_ESM.zip › Supplementary Data2/bin.30.draw_carbon_cycle_single.pdf]

# Nitrogen Cycle: bin.151

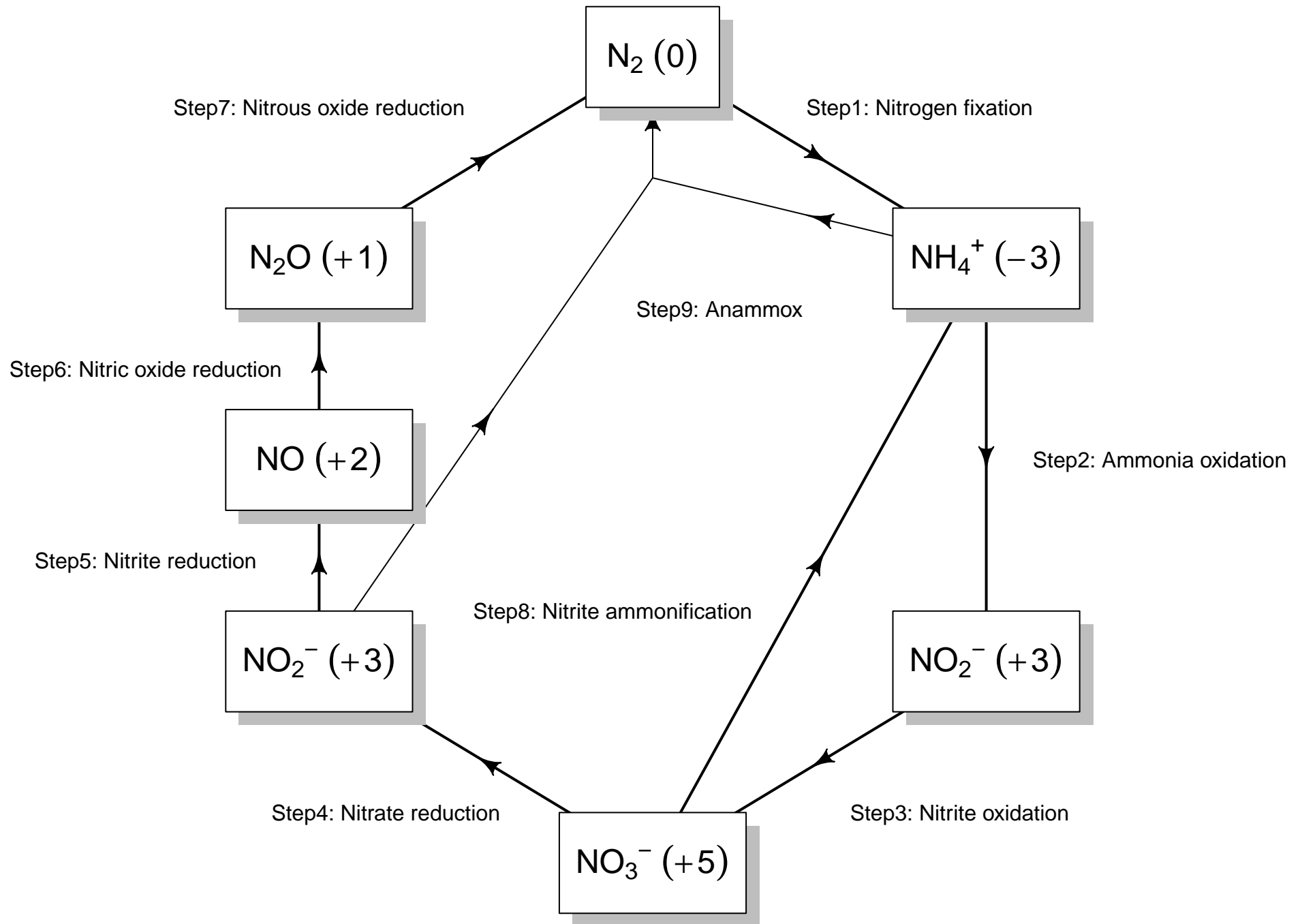

Supplement: Supplementary file 2 — Recovered nutrient cycles including Carbon, Nitrogen, and Sulphur cycles for all the metagenomic assembled genomes given as PDF images, and identifiable through bin numbers. [file 13104_2025_7441_MOESM2_ESM.zip › Supplementary Data2/bin.151.draw_nitrogen_cycle_single.pdf]

## Sulfur Cycle: bin.8

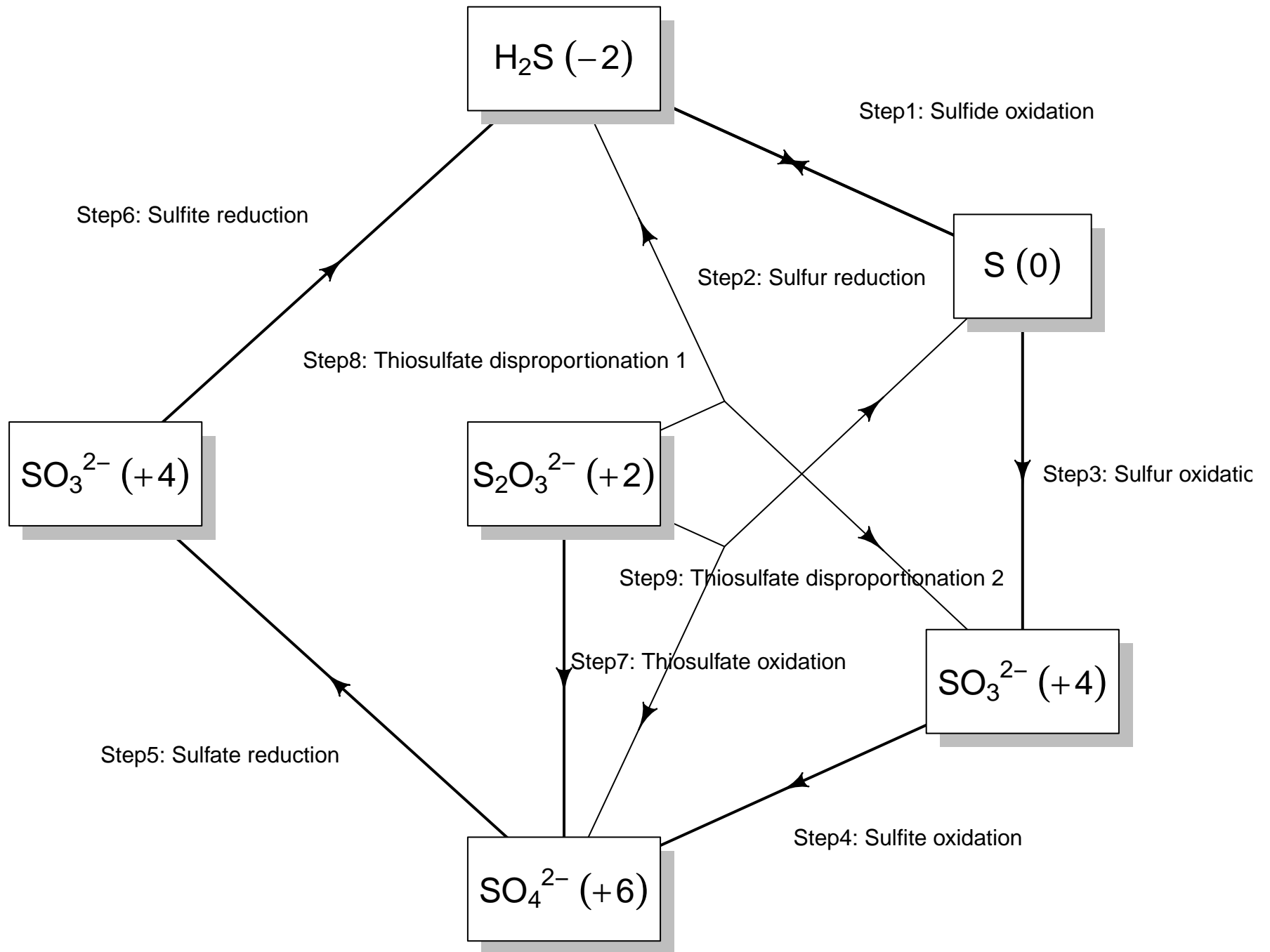

Supplement: Supplementary file 2 — Recovered nutrient cycles including Carbon, Nitrogen, and Sulphur cycles for all the metagenomic assembled genomes given as PDF images, and identifiable through bin numbers. [file 13104_2025_7441_MOESM2_ESM.zip › Supplementary Data2/bin.8.draw_sulfur_cycle_single.pdf]

# Carbon Cycle: bin.25

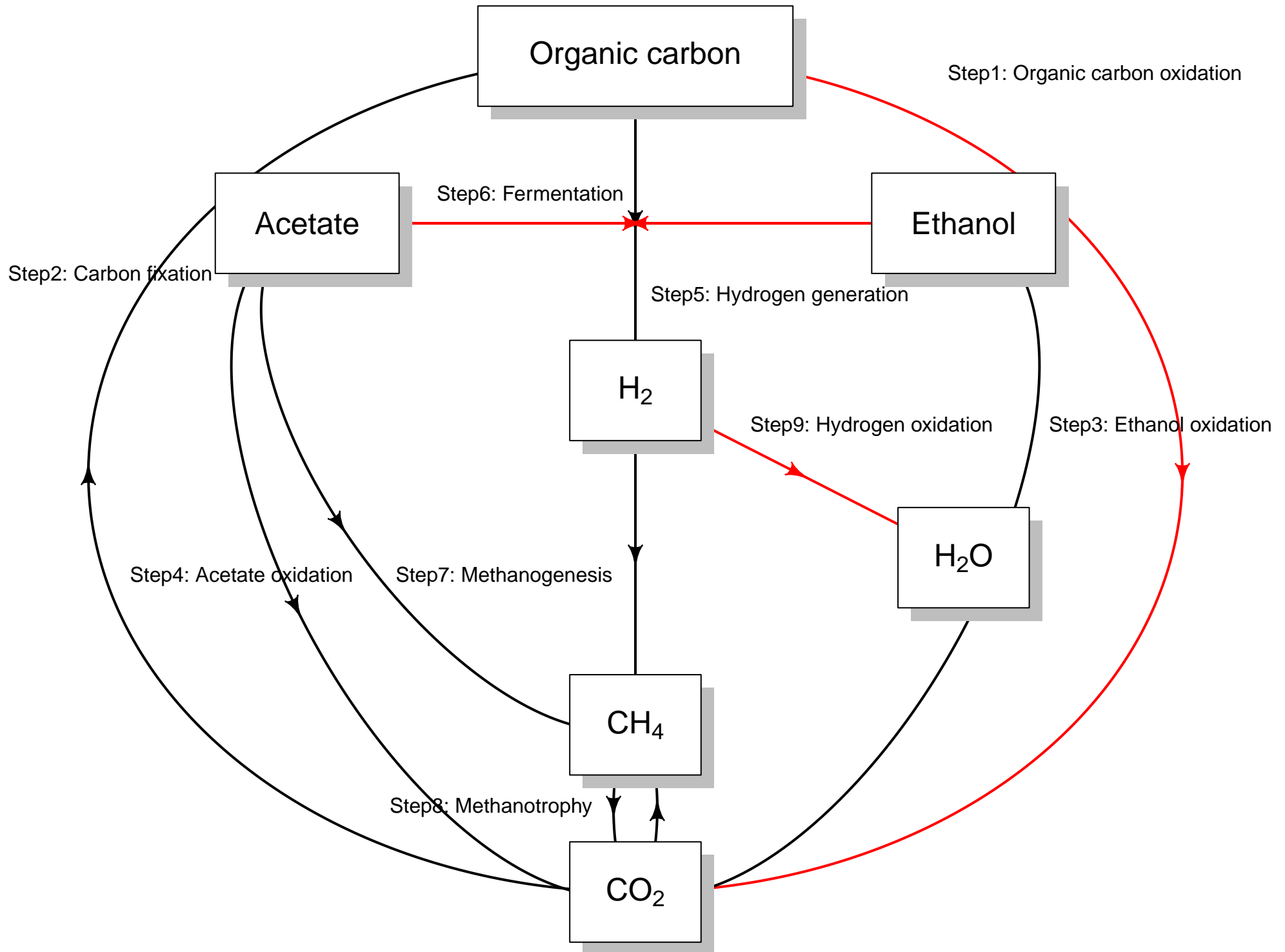

Supplement: Supplementary file 2 — Recovered nutrient cycles including Carbon, Nitrogen, and Sulphur cycles for all the metagenomic assembled genomes given as PDF images, and identifiable through bin numbers. [file 13104_2025_7441_MOESM2_ESM.zip › Supplementary Data2/bin.25.draw_carbon_cycle_single.pdf]

## Sulfur Cycle: bin.6

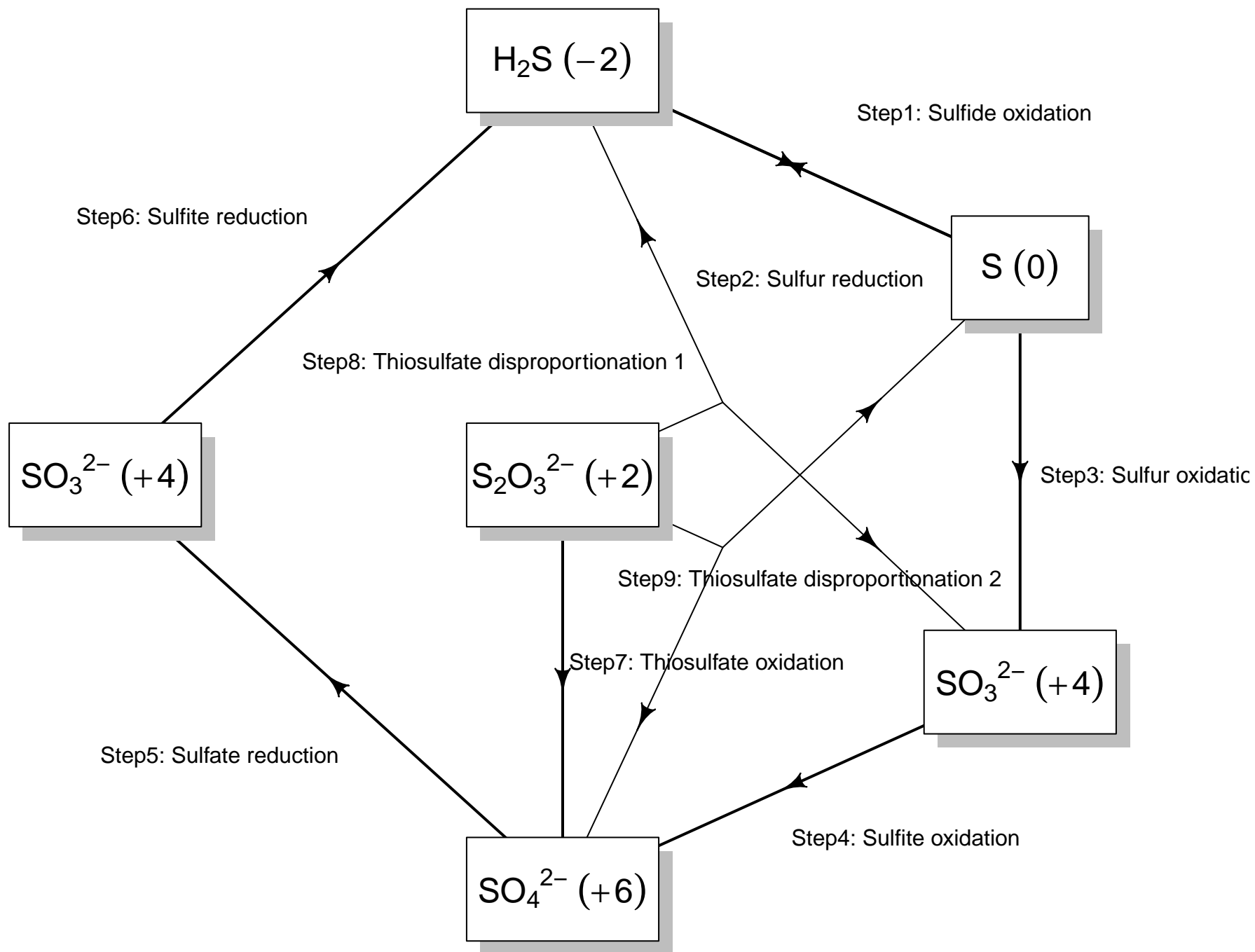

Supplement: Supplementary file 2 — Recovered nutrient cycles including Carbon, Nitrogen, and Sulphur cycles for all the metagenomic assembled genomes given as PDF images, and identifiable through bin numbers. [file 13104_2025_7441_MOESM2_ESM.zip › Supplementary Data2/bin.6.draw_sulfur_cycle_single.pdf]

# Carbon Cycle: bin.118

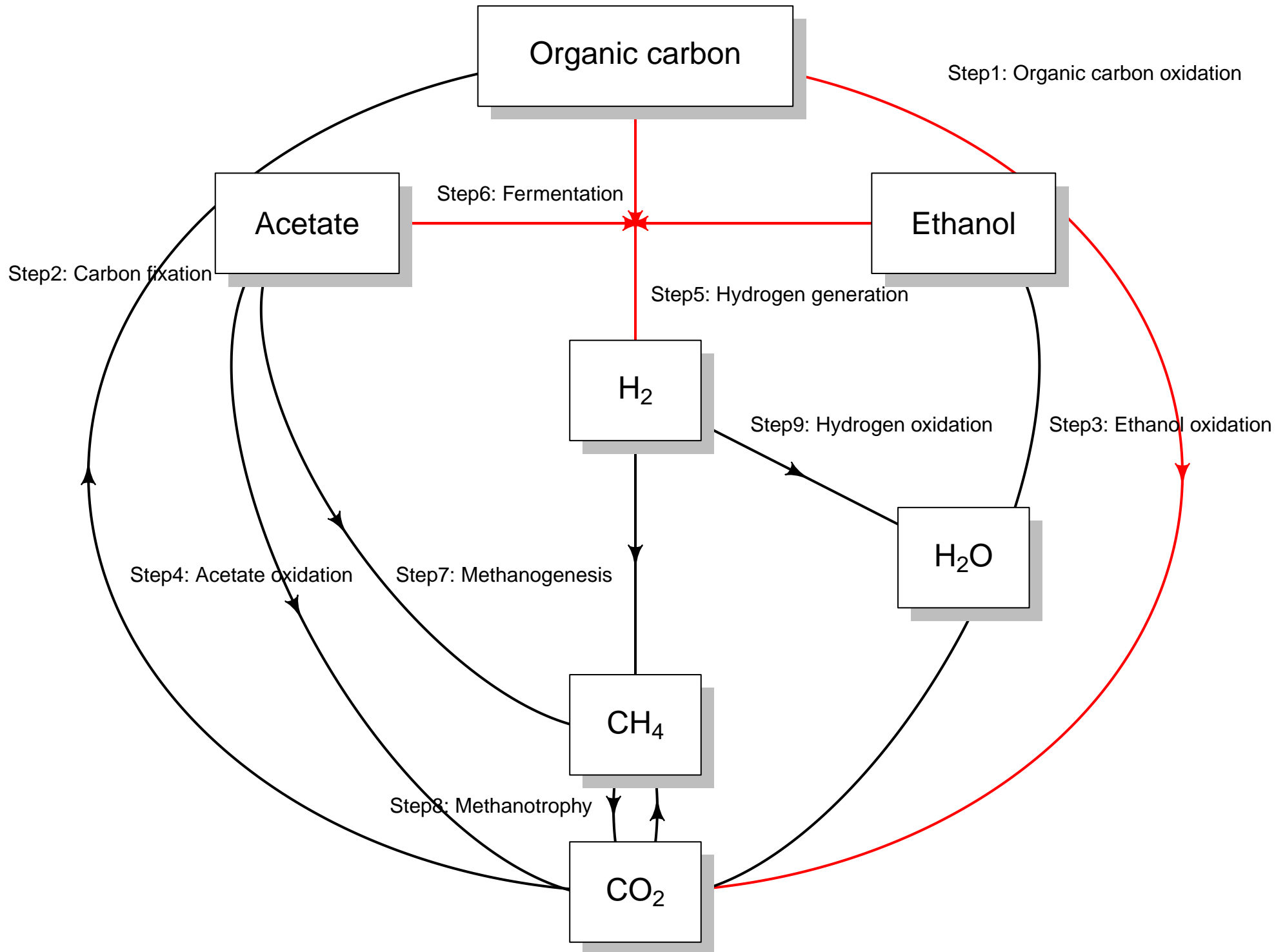

Supplement: Supplementary file 2 — Recovered nutrient cycles including Carbon, Nitrogen, and Sulphur cycles for all the metagenomic assembled genomes given as PDF images, and identifiable through bin numbers. [file 13104_2025_7441_MOESM2_ESM.zip › Supplementary Data2/bin.118.draw_carbon_cycle_single.pdf]

## Sulfur Cycle: bin.90

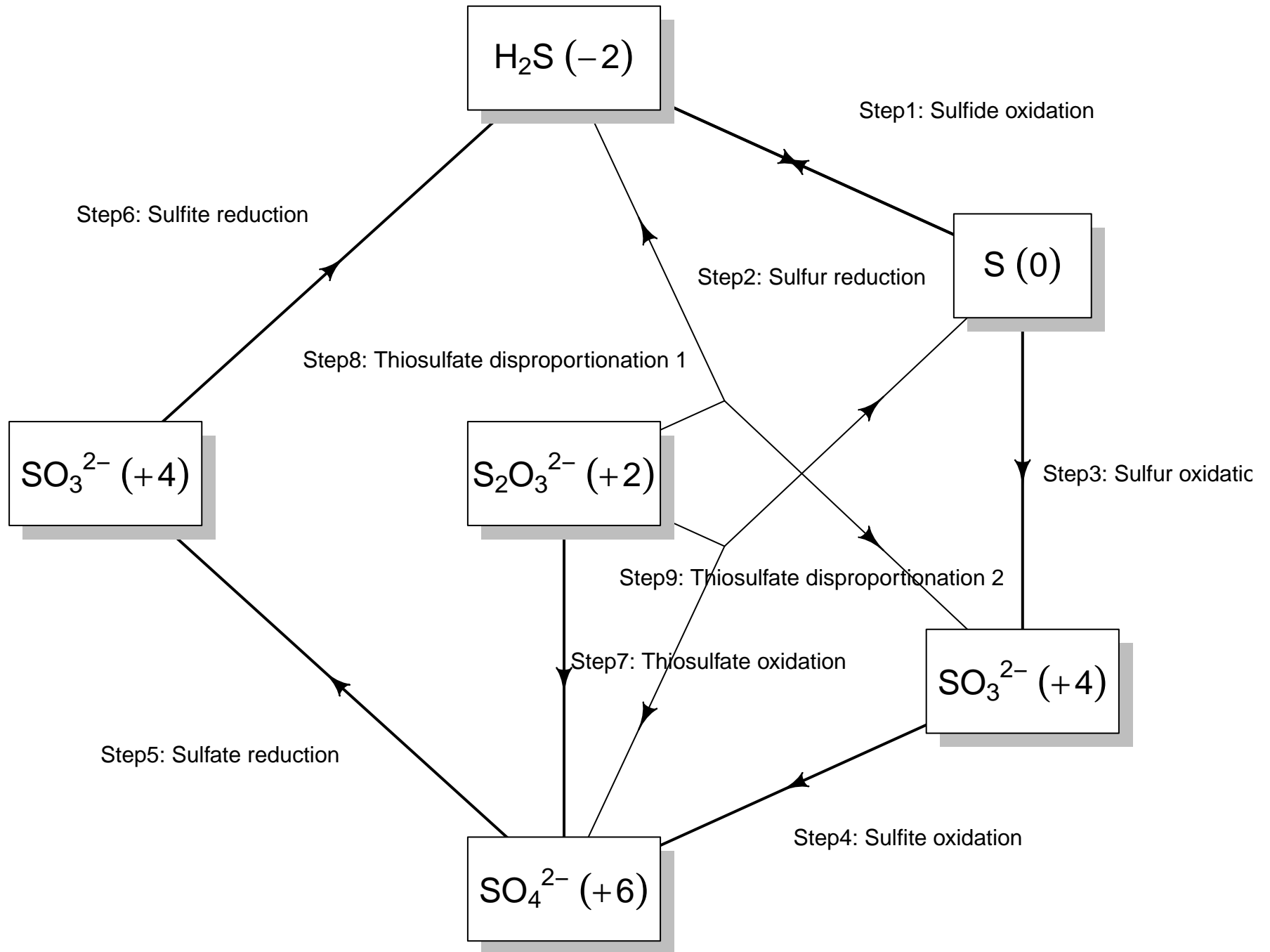

Supplement: Supplementary file 2 — Recovered nutrient cycles including Carbon, Nitrogen, and Sulphur cycles for all the metagenomic assembled genomes given as PDF images, and identifiable through bin numbers. [file 13104_2025_7441_MOESM2_ESM.zip › Supplementary Data2/bin.90.draw_sulfur_cycle_single.pdf]

## Sulfur Cycle : Summary Figure

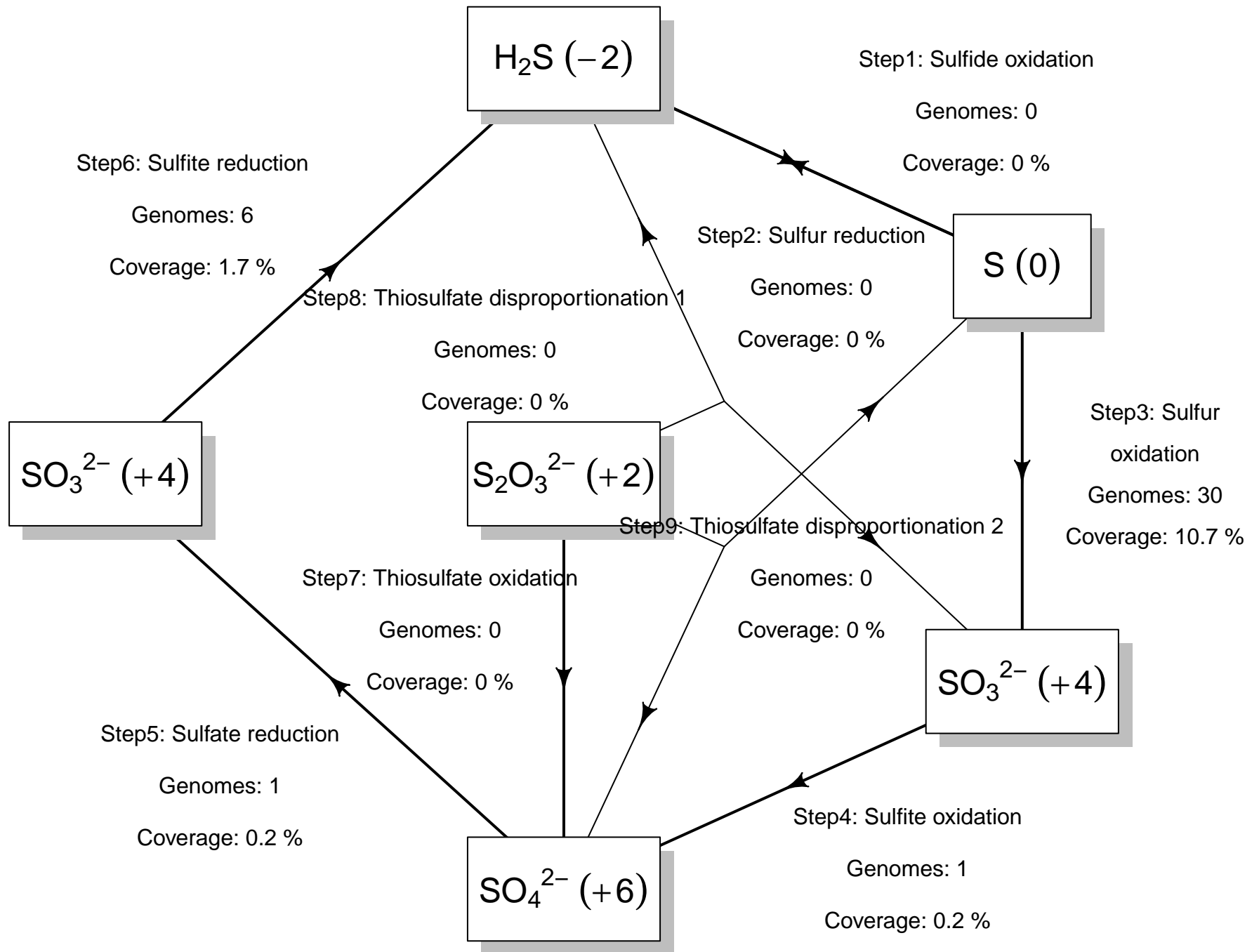

Supplement: Supplementary file 2 — Recovered nutrient cycles including Carbon, Nitrogen, and Sulphur cycles for all the metagenomic assembled genomes given as PDF images, and identifiable through bin numbers. [file 13104_2025_7441_MOESM2_ESM.zip › Supplementary Data2/draw_sulfur_cycle_total.pdf]

# Carbon Cycle: bin.116

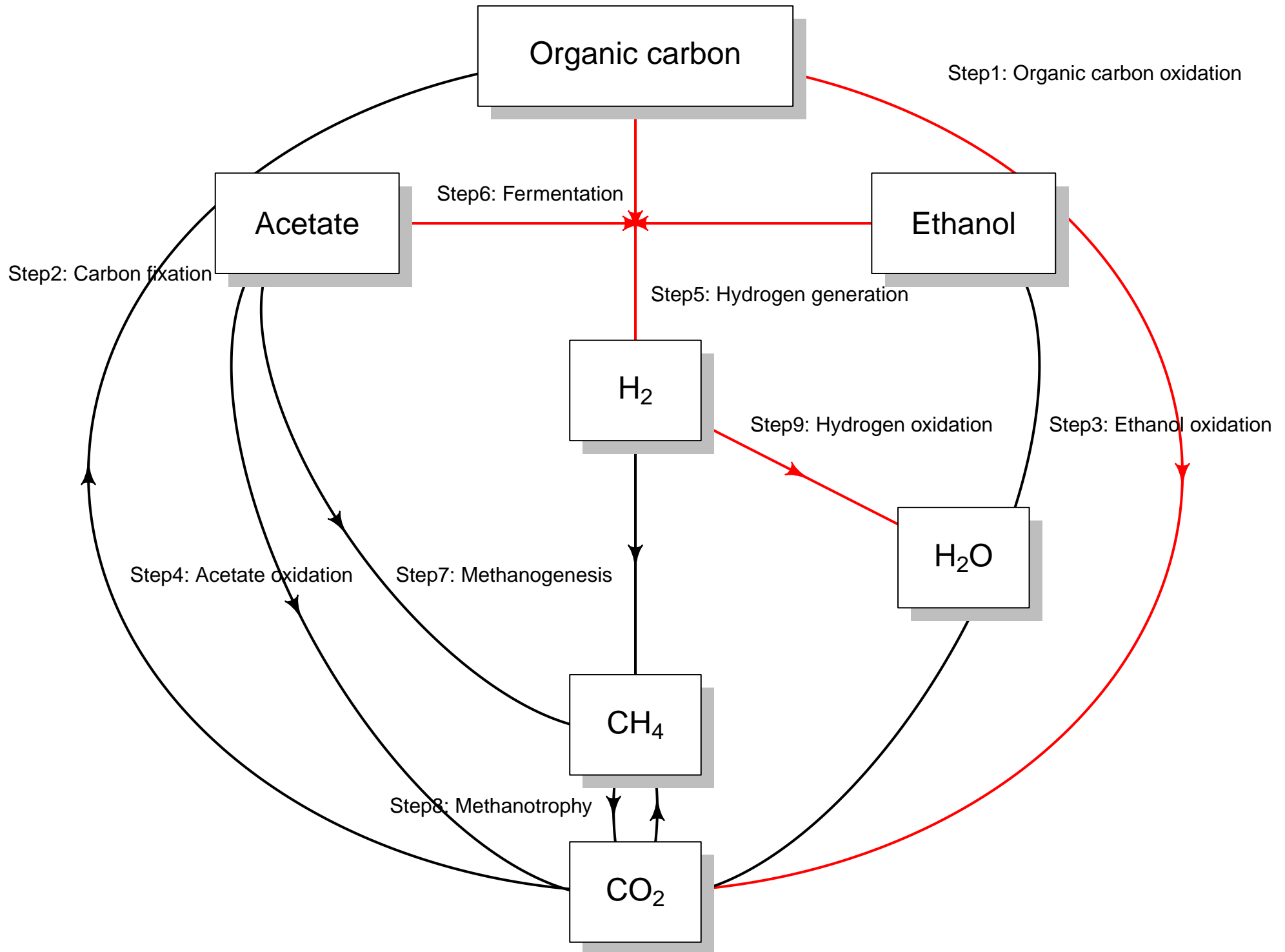

Supplement: Supplementary file 2 — Recovered nutrient cycles including Carbon, Nitrogen, and Sulphur cycles for all the metagenomic assembled genomes given as PDF images, and identifiable through bin numbers. [file 13104_2025_7441_MOESM2_ESM.zip › Supplementary Data2/bin.116.draw_carbon_cycle_single.pdf]

# Nitrogen Cycle: bin.157

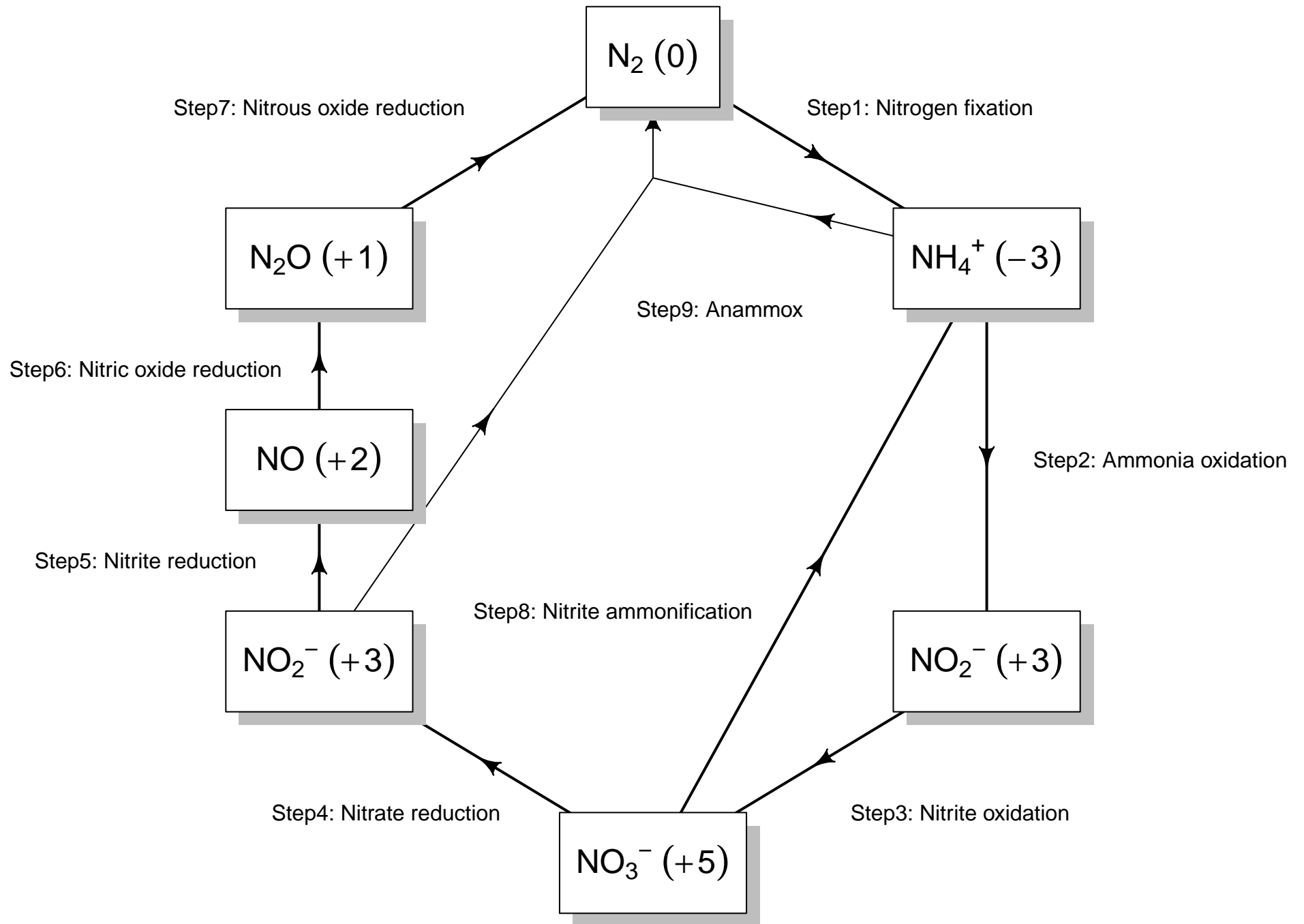

Supplement: Supplementary file 2 — Recovered nutrient cycles including Carbon, Nitrogen, and Sulphur cycles for all the metagenomic assembled genomes given as PDF images, and identifiable through bin numbers. [file 13104_2025_7441_MOESM2_ESM.zip › Supplementary Data2/bin.157.draw_nitrogen_cycle_single.pdf]

## Sulfur Cycle: bin.22

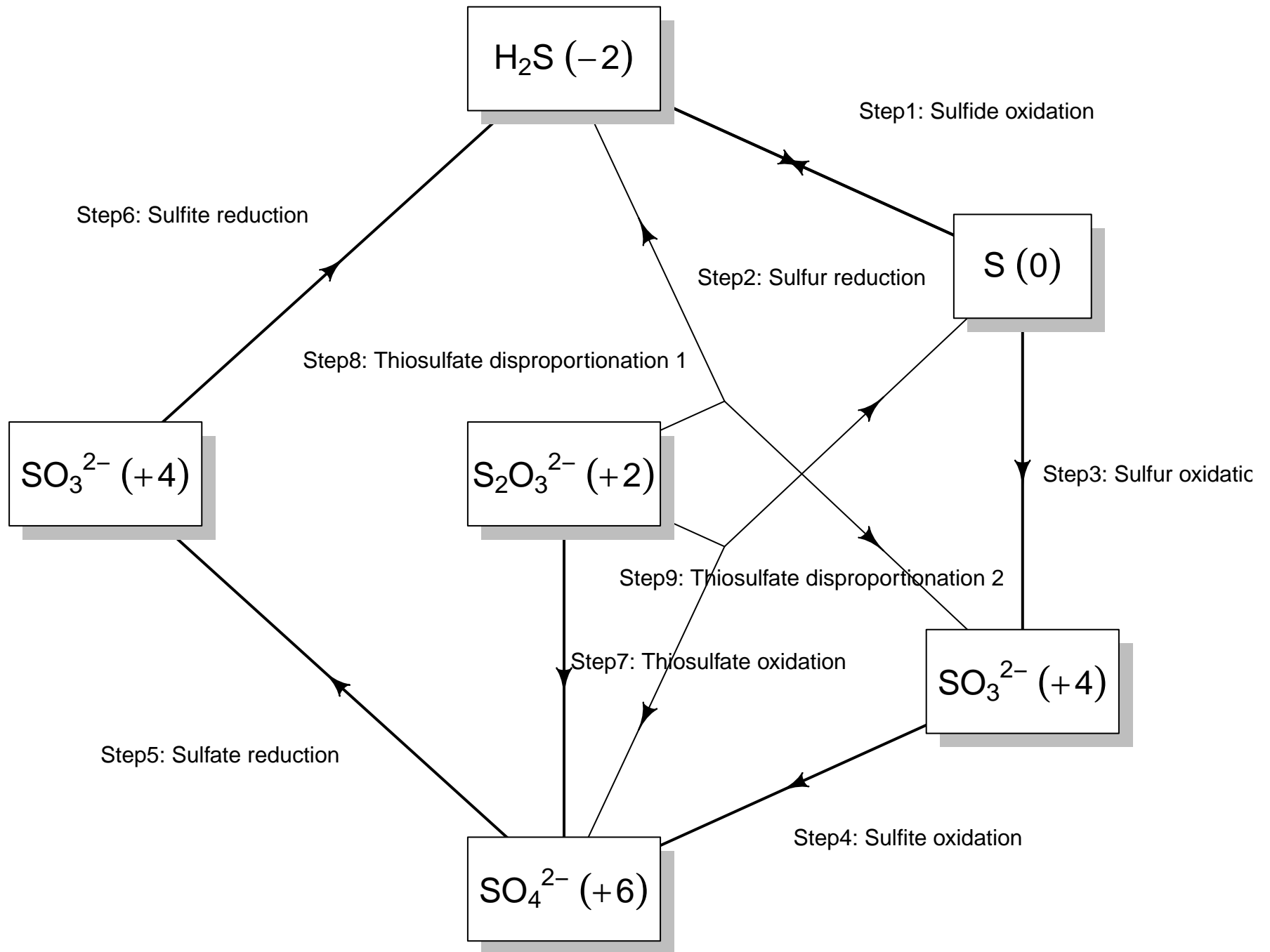

Supplement: Supplementary file 2 — Recovered nutrient cycles including Carbon, Nitrogen, and Sulphur cycles for all the metagenomic assembled genomes given as PDF images, and identifiable through bin numbers. [file 13104_2025_7441_MOESM2_ESM.zip › Supplementary Data2/bin.22.draw_sulfur_cycle_single.pdf]

## Nitrogen Cycle: bin.118

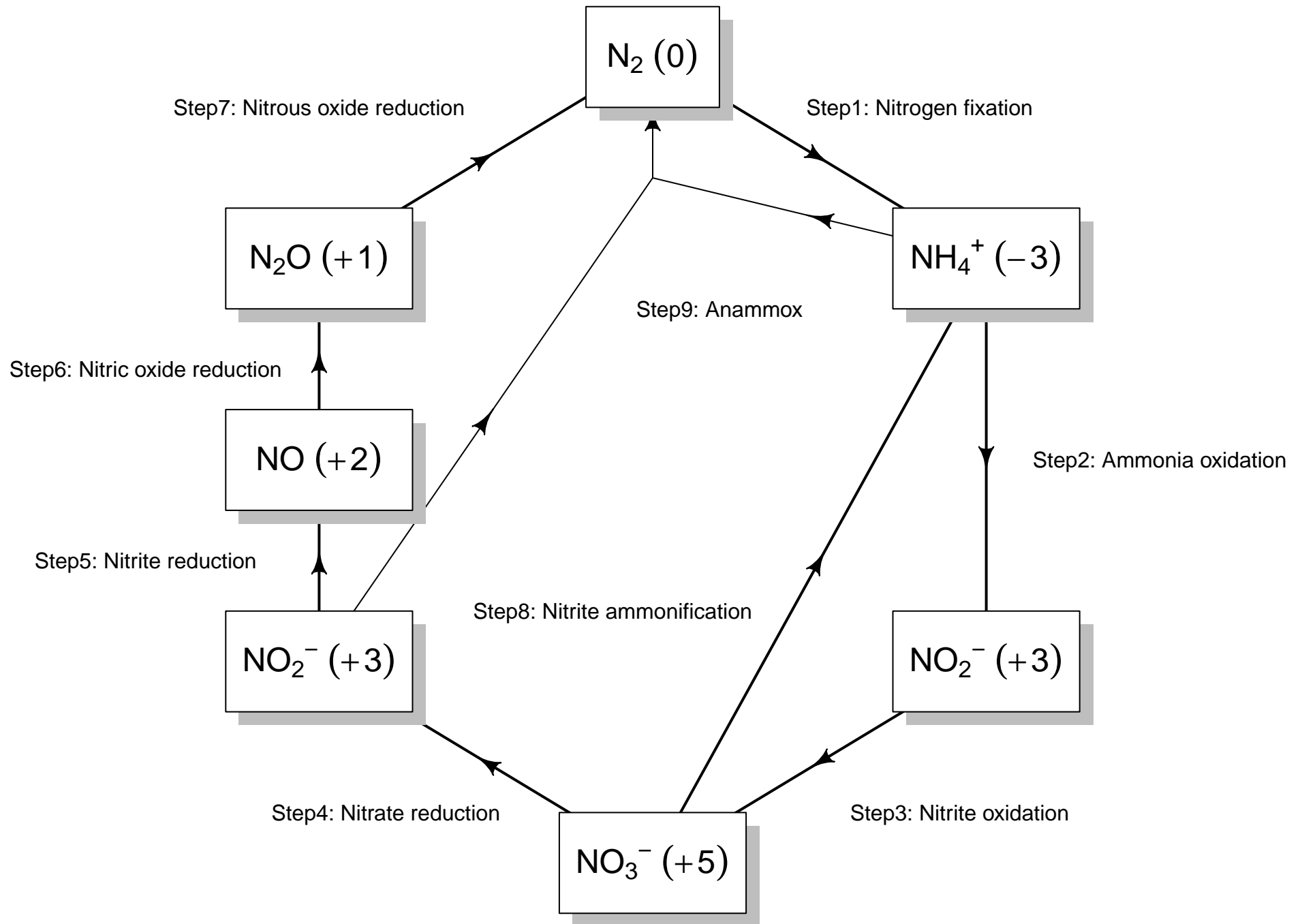

Supplement: Supplementary file 2 — Recovered nutrient cycles including Carbon, Nitrogen, and Sulphur cycles for all the metagenomic assembled genomes given as PDF images, and identifiable through bin numbers. [file 13104_2025_7441_MOESM2_ESM.zip › Supplementary Data2/bin.118.draw_nitrogen_cycle_single.pdf]

# Carbon Cycle: bin.132

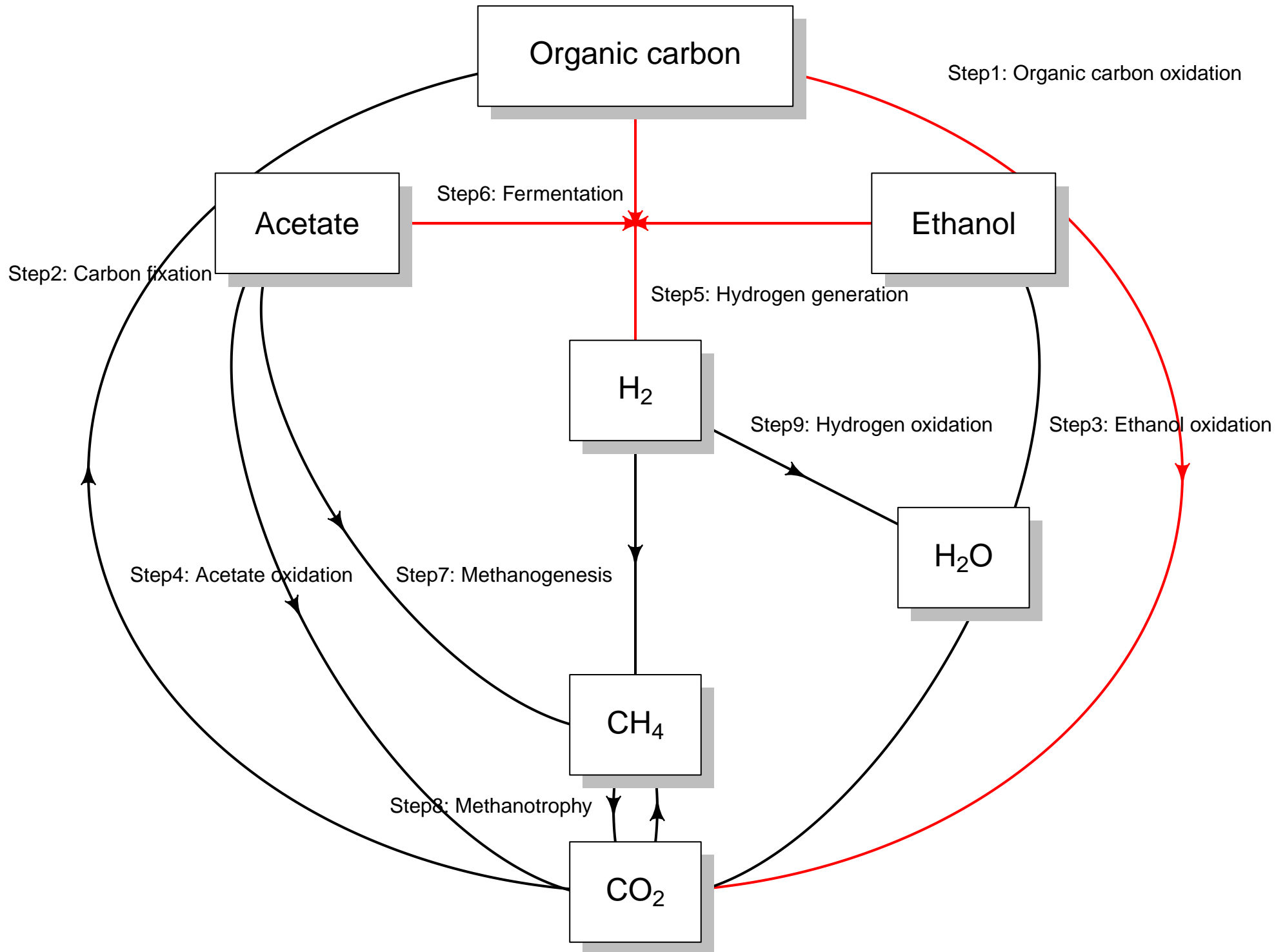

Supplement: Supplementary file 2 — Recovered nutrient cycles including Carbon, Nitrogen, and Sulphur cycles for all the metagenomic assembled genomes given as PDF images, and identifiable through bin numbers. [file 13104_2025_7441_MOESM2_ESM.zip › Supplementary Data2/bin.132.draw_carbon_cycle_single.pdf]

# Carbon Cycle: bin.1

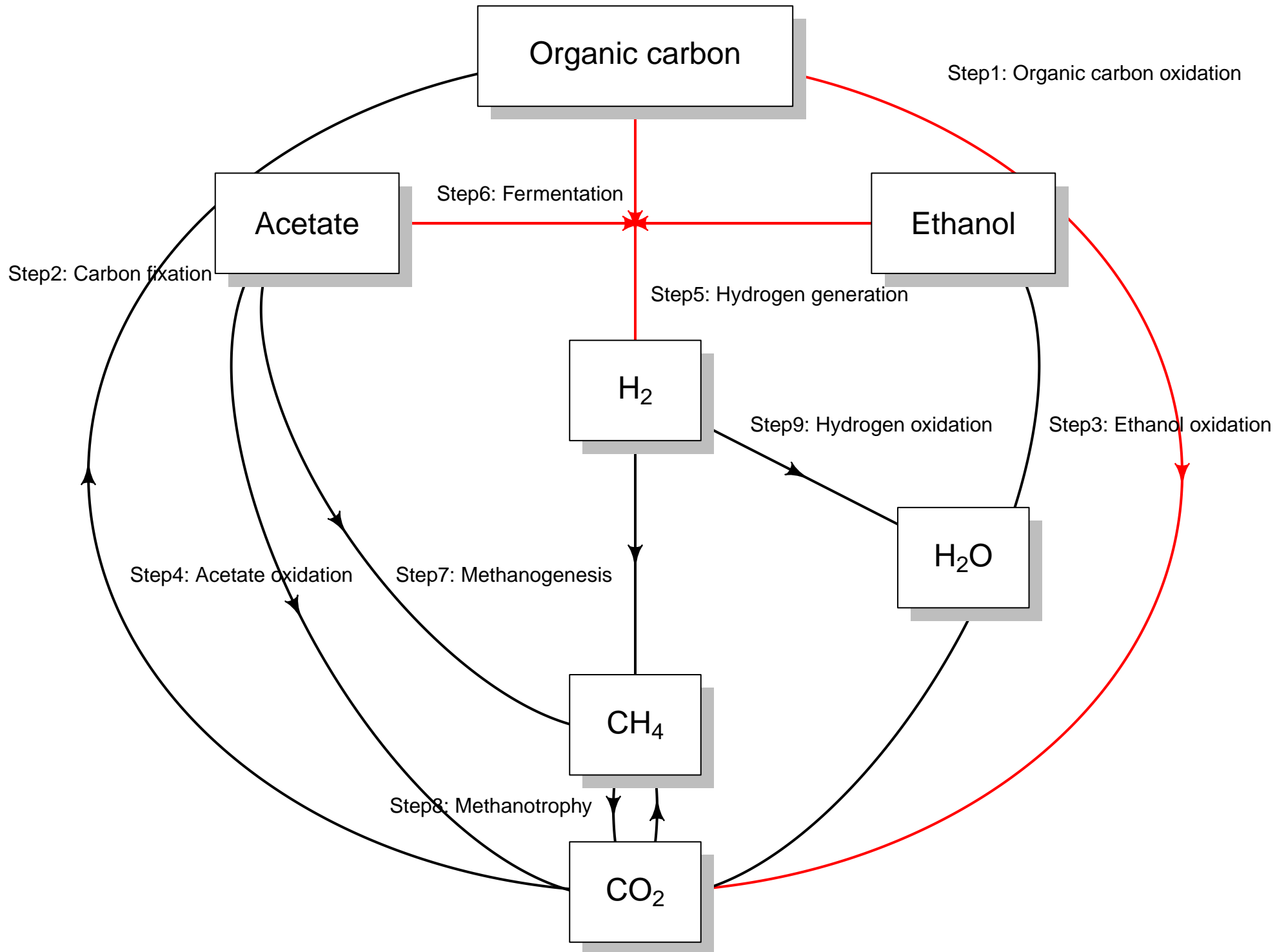

Supplement: Supplementary file 2 — Recovered nutrient cycles including Carbon, Nitrogen, and Sulphur cycles for all the metagenomic assembled genomes given as PDF images, and identifiable through bin numbers. [file 13104_2025_7441_MOESM2_ESM.zip › Supplementary Data2/bin.1.draw_carbon_cycle_single.pdf]

## Nitrogen Cycle: bin.13

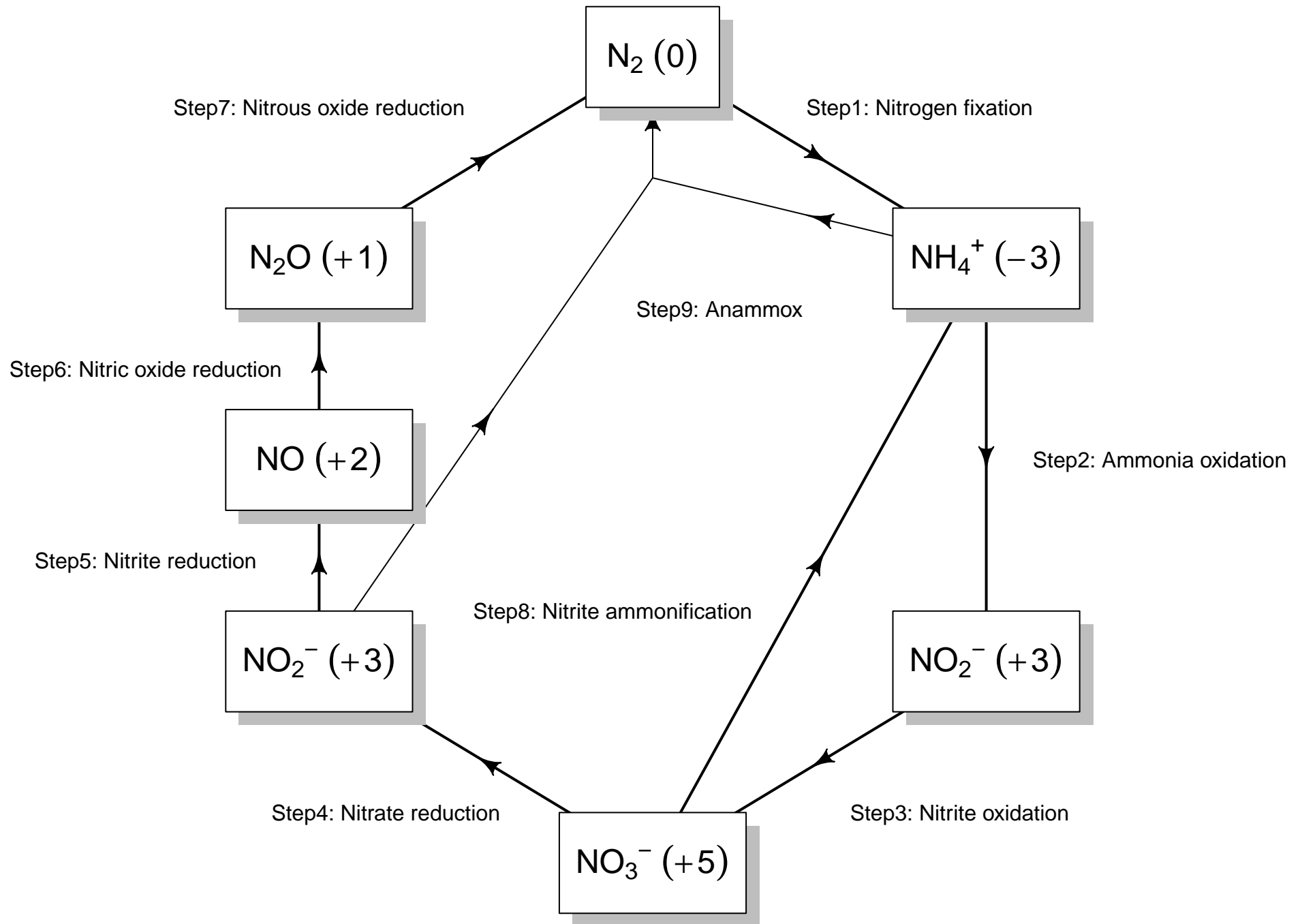

Supplement: Supplementary file 2 — Recovered nutrient cycles including Carbon, Nitrogen, and Sulphur cycles for all the metagenomic assembled genomes given as PDF images, and identifiable through bin numbers. [file 13104_2025_7441_MOESM2_ESM.zip › Supplementary Data2/bin.13.draw_nitrogen_cycle_single.pdf]

# Carbon Cycle: bin.97

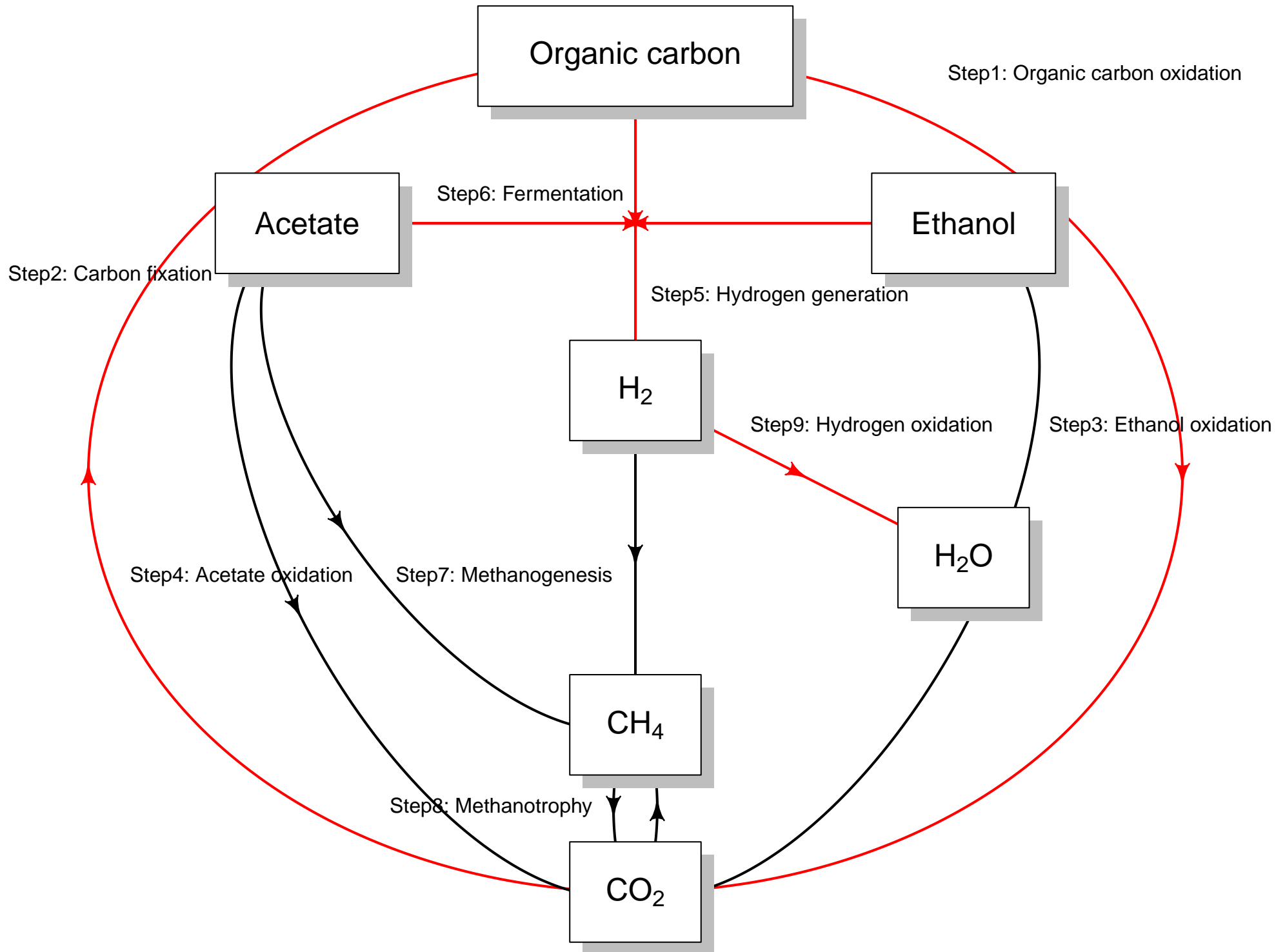

Supplement: Supplementary file 2 — Recovered nutrient cycles including Carbon, Nitrogen, and Sulphur cycles for all the metagenomic assembled genomes given as PDF images, and identifiable through bin numbers. [file 13104_2025_7441_MOESM2_ESM.zip › Supplementary Data2/bin.97.draw_carbon_cycle_single.pdf]

# Carbon Cycle: bin.99

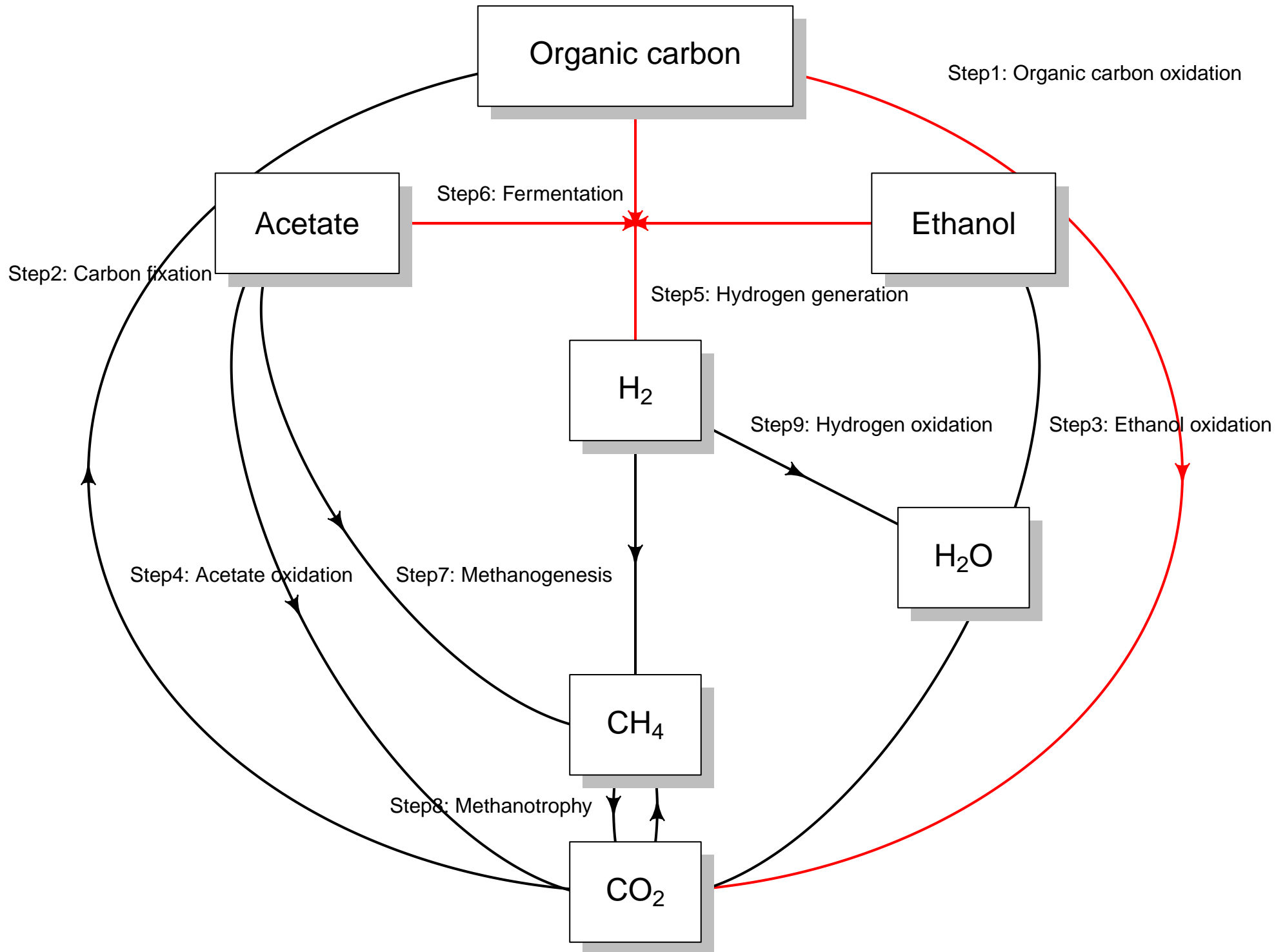

Supplement: Supplementary file 2 — Recovered nutrient cycles including Carbon, Nitrogen, and Sulphur cycles for all the metagenomic assembled genomes given as PDF images, and identifiable through bin numbers. [file 13104_2025_7441_MOESM2_ESM.zip › Supplementary Data2/bin.99.draw_carbon_cycle_single.pdf]

## Sulfur Cycle: bin.111

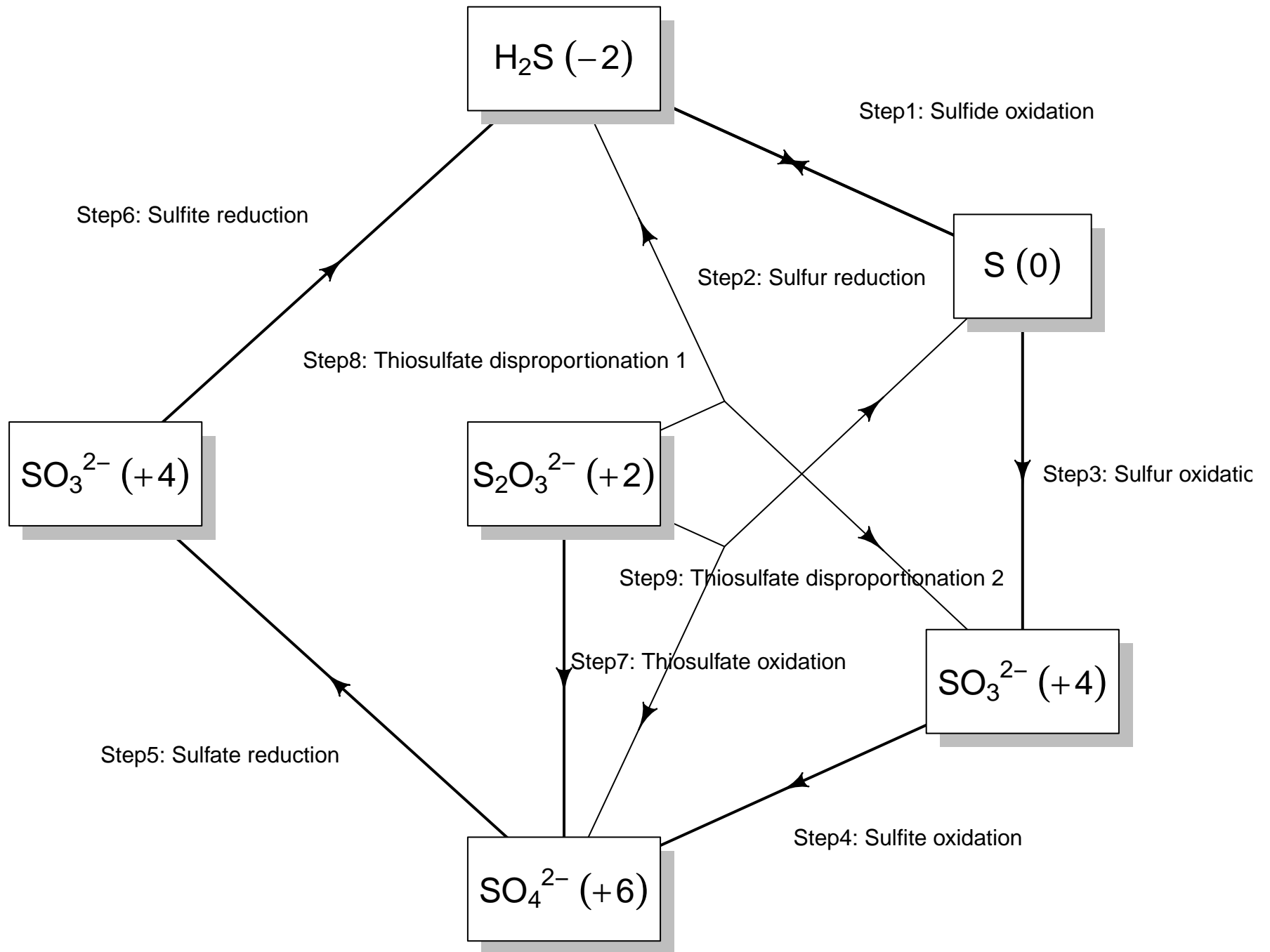

Supplement: Supplementary file 2 — Recovered nutrient cycles including Carbon, Nitrogen, and Sulphur cycles for all the metagenomic assembled genomes given as PDF images, and identifiable through bin numbers. [file 13104_2025_7441_MOESM2_ESM.zip › Supplementary Data2/bin.111.draw_sulfur_cycle_single.pdf]

# Carbon Cycle: bin.14

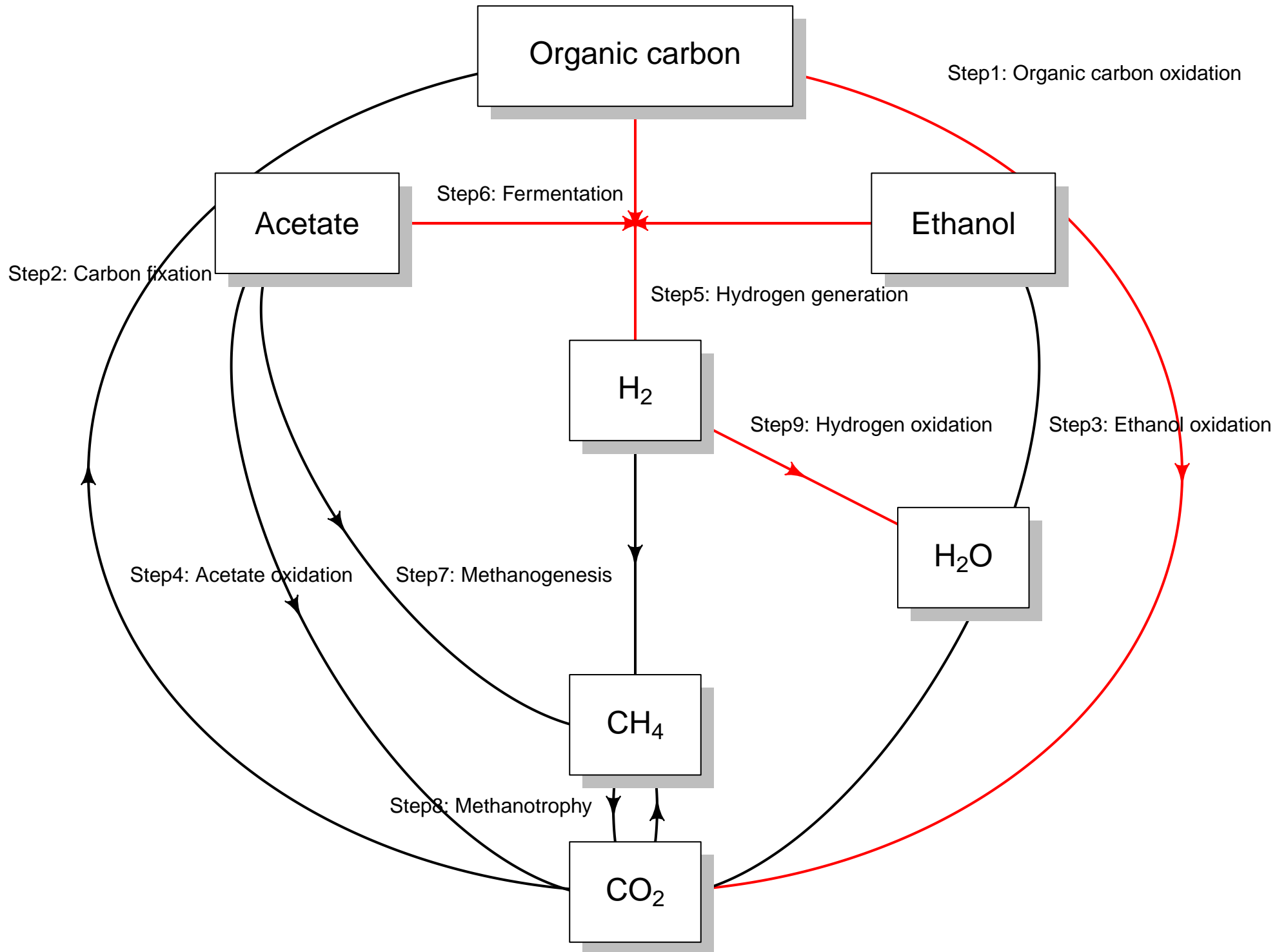

Supplement: Supplementary file 2 — Recovered nutrient cycles including Carbon, Nitrogen, and Sulphur cycles for all the metagenomic assembled genomes given as PDF images, and identifiable through bin numbers. [file 13104_2025_7441_MOESM2_ESM.zip › Supplementary Data2/bin.14.draw_carbon_cycle_single.pdf]

## Sulfur Cycle: bin.104

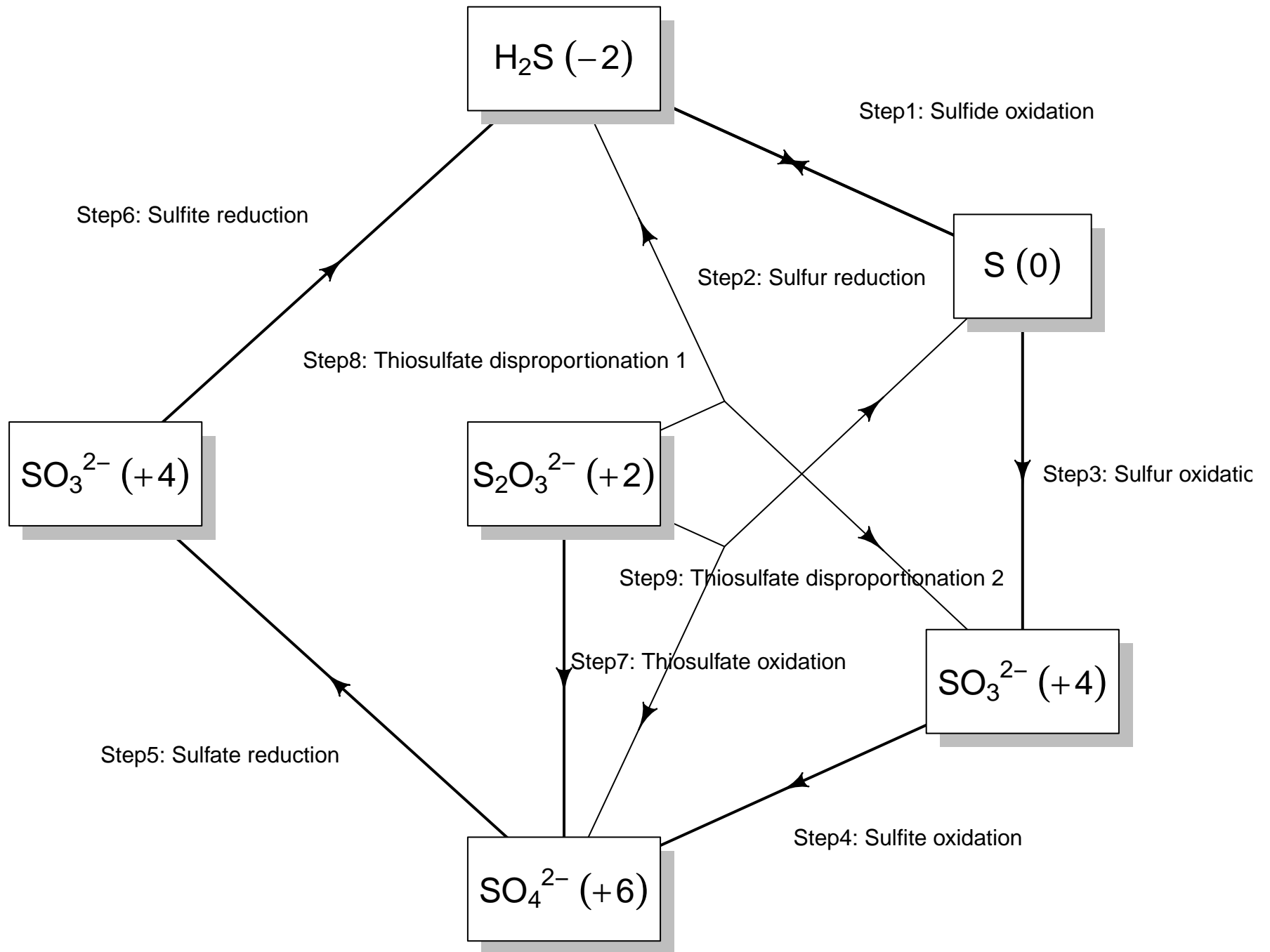

Supplement: Supplementary file 2 — Recovered nutrient cycles including Carbon, Nitrogen, and Sulphur cycles for all the metagenomic assembled genomes given as PDF images, and identifiable through bin numbers. [file 13104_2025_7441_MOESM2_ESM.zip › Supplementary Data2/bin.104.draw_sulfur_cycle_single.pdf]

# Carbon Cycle: bin.82

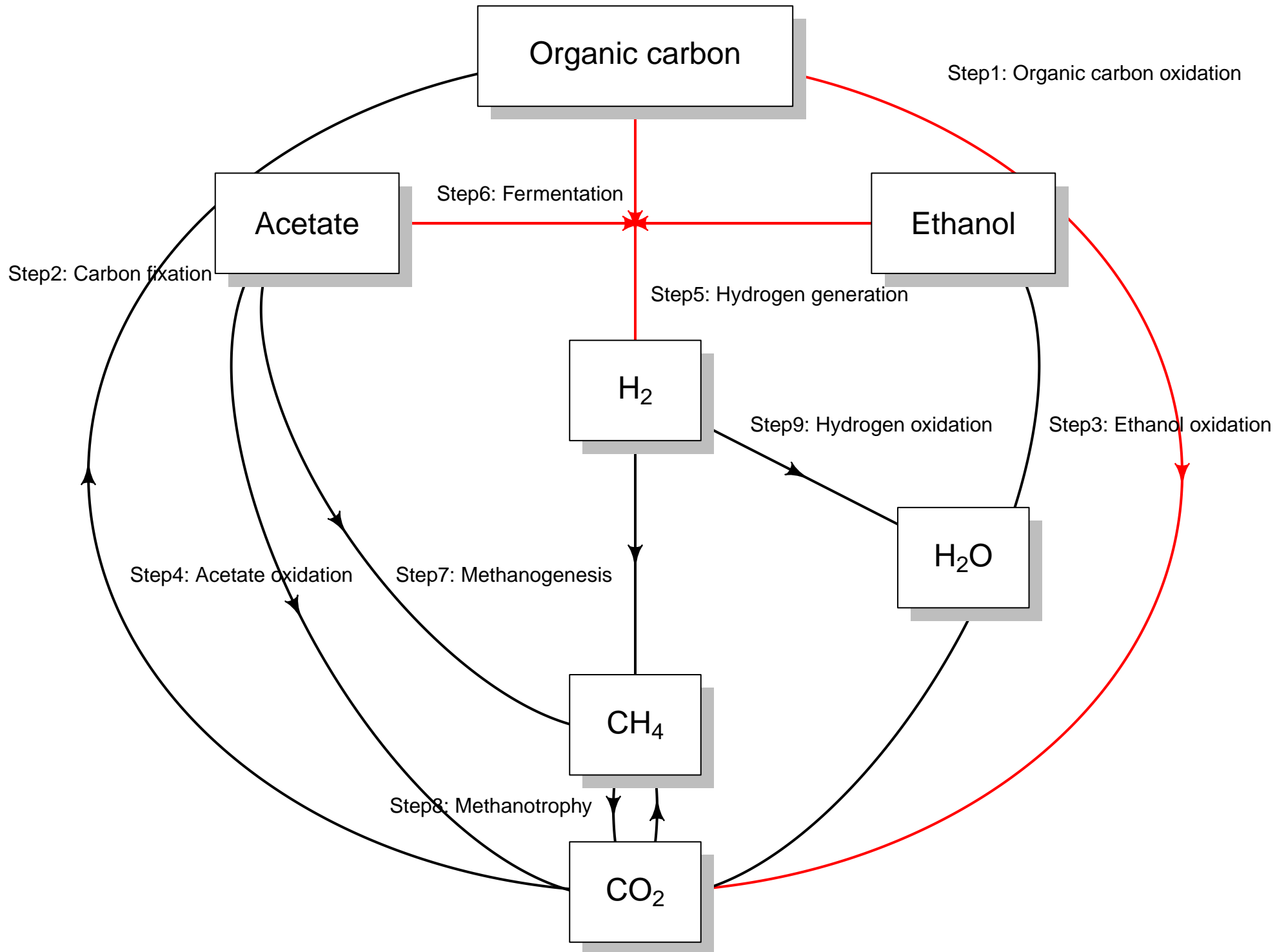

Supplement: Supplementary file 2 — Recovered nutrient cycles including Carbon, Nitrogen, and Sulphur cycles for all the metagenomic assembled genomes given as PDF images, and identifiable through bin numbers. [file 13104_2025_7441_MOESM2_ESM.zip › Supplementary Data2/bin.82.draw_carbon_cycle_single.pdf]

# Carbon Cycle: bin.127

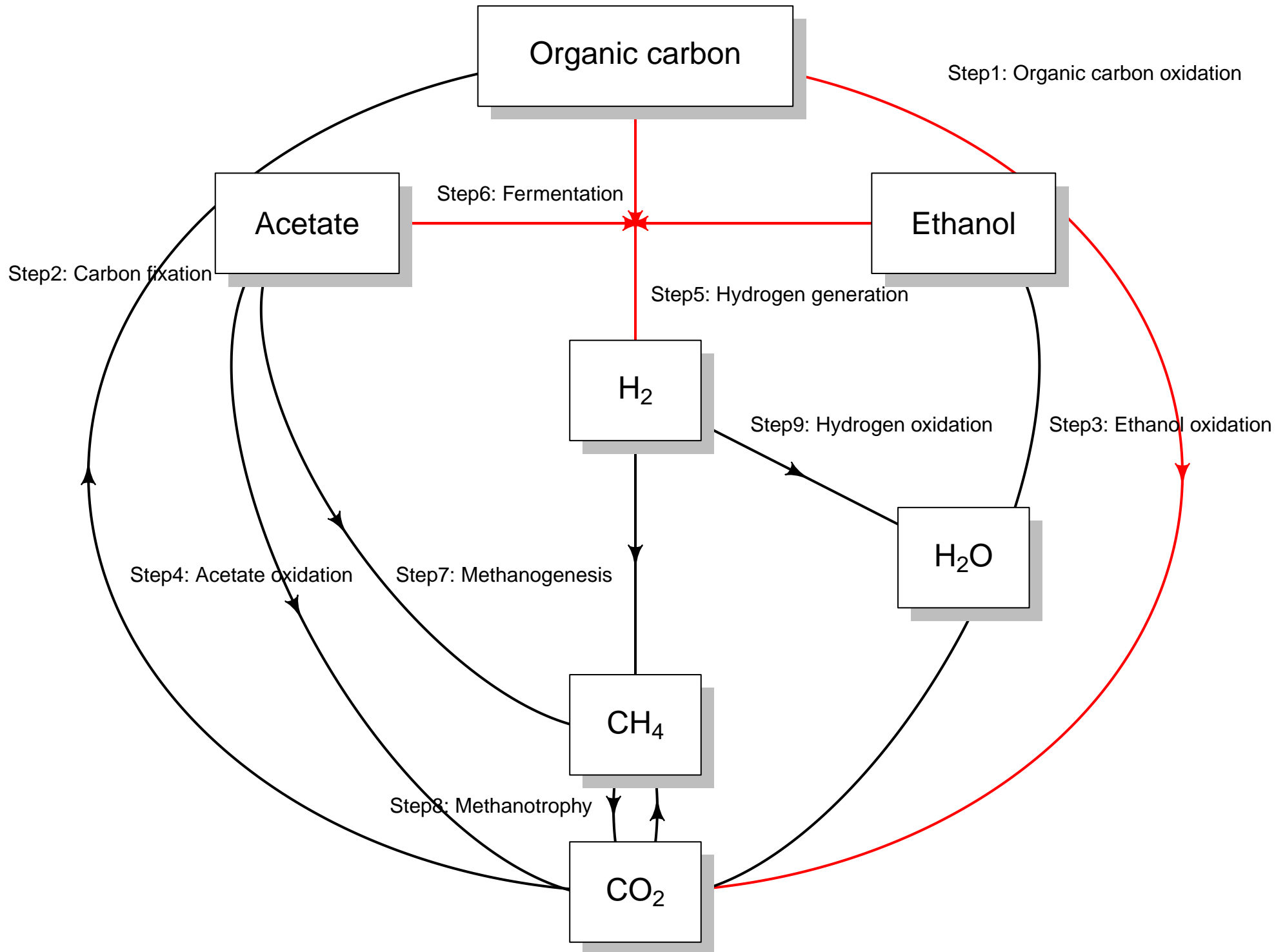

Supplement: Supplementary file 2 — Recovered nutrient cycles including Carbon, Nitrogen, and Sulphur cycles for all the metagenomic assembled genomes given as PDF images, and identifiable through bin numbers. [file 13104_2025_7441_MOESM2_ESM.zip › Supplementary Data2/bin.127.draw_carbon_cycle_single.pdf]

## Sulfur Cycle: bin.37

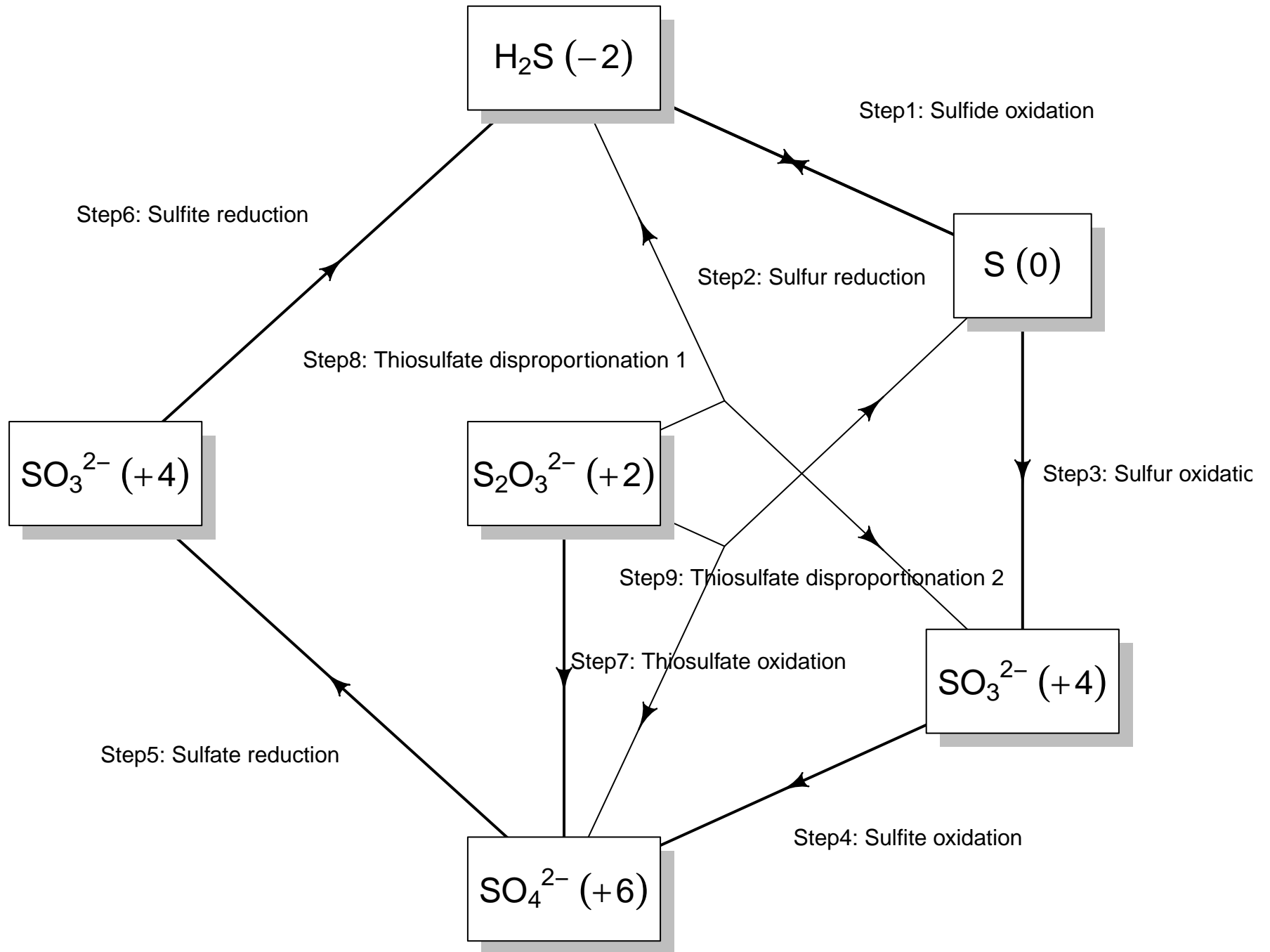

Supplement: Supplementary file 2 — Recovered nutrient cycles including Carbon, Nitrogen, and Sulphur cycles for all the metagenomic assembled genomes given as PDF images, and identifiable through bin numbers. [file 13104_2025_7441_MOESM2_ESM.zip › Supplementary Data2/bin.37.draw_sulfur_cycle_single.pdf]

# Carbon Cycle: bin.129

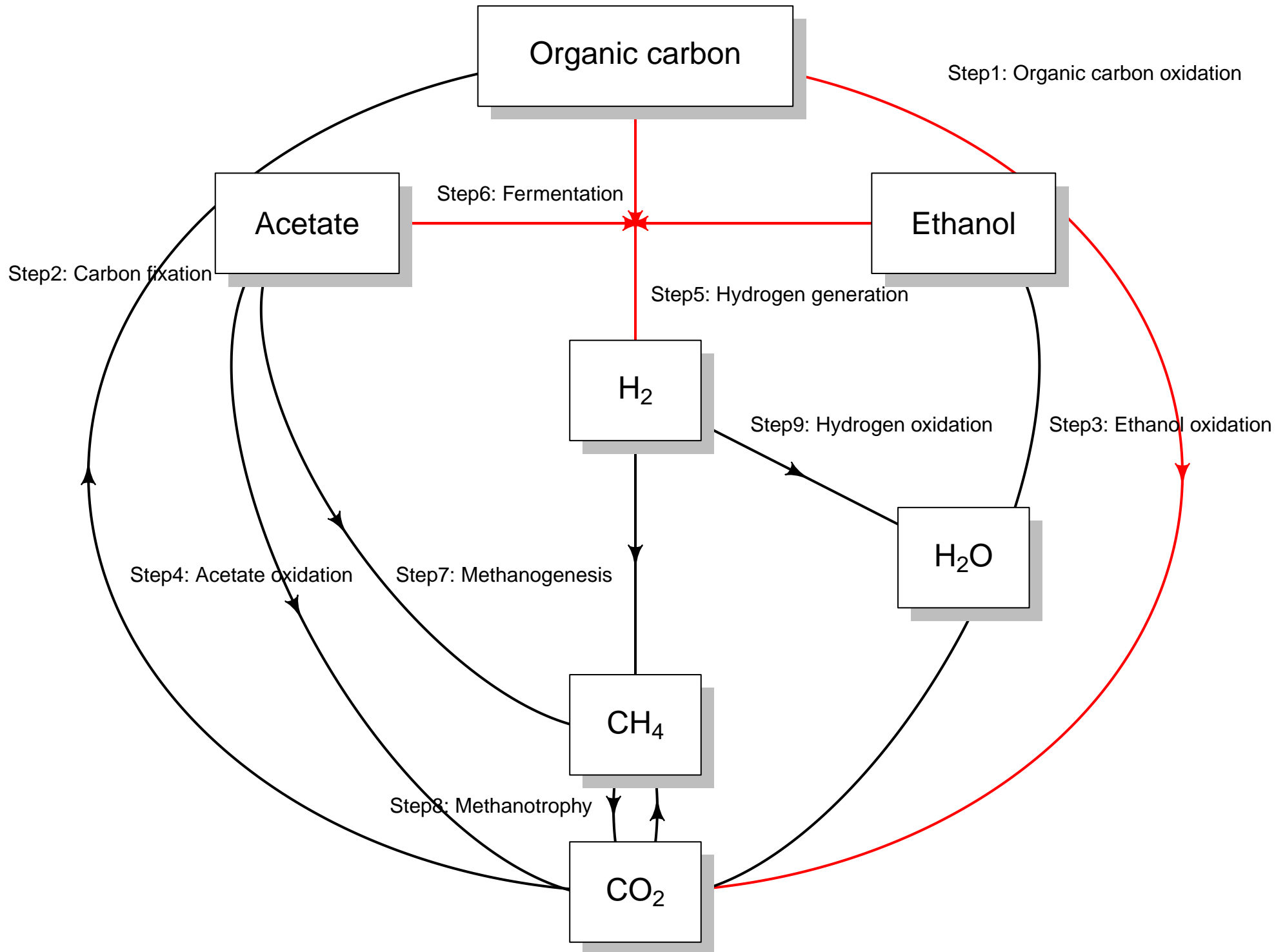

Supplement: Supplementary file 2 — Recovered nutrient cycles including Carbon, Nitrogen, and Sulphur cycles for all the metagenomic assembled genomes given as PDF images, and identifiable through bin numbers. [file 13104_2025_7441_MOESM2_ESM.zip › Supplementary Data2/bin.129.draw_carbon_cycle_single.pdf]

## Sulfur Cycle: bin.39

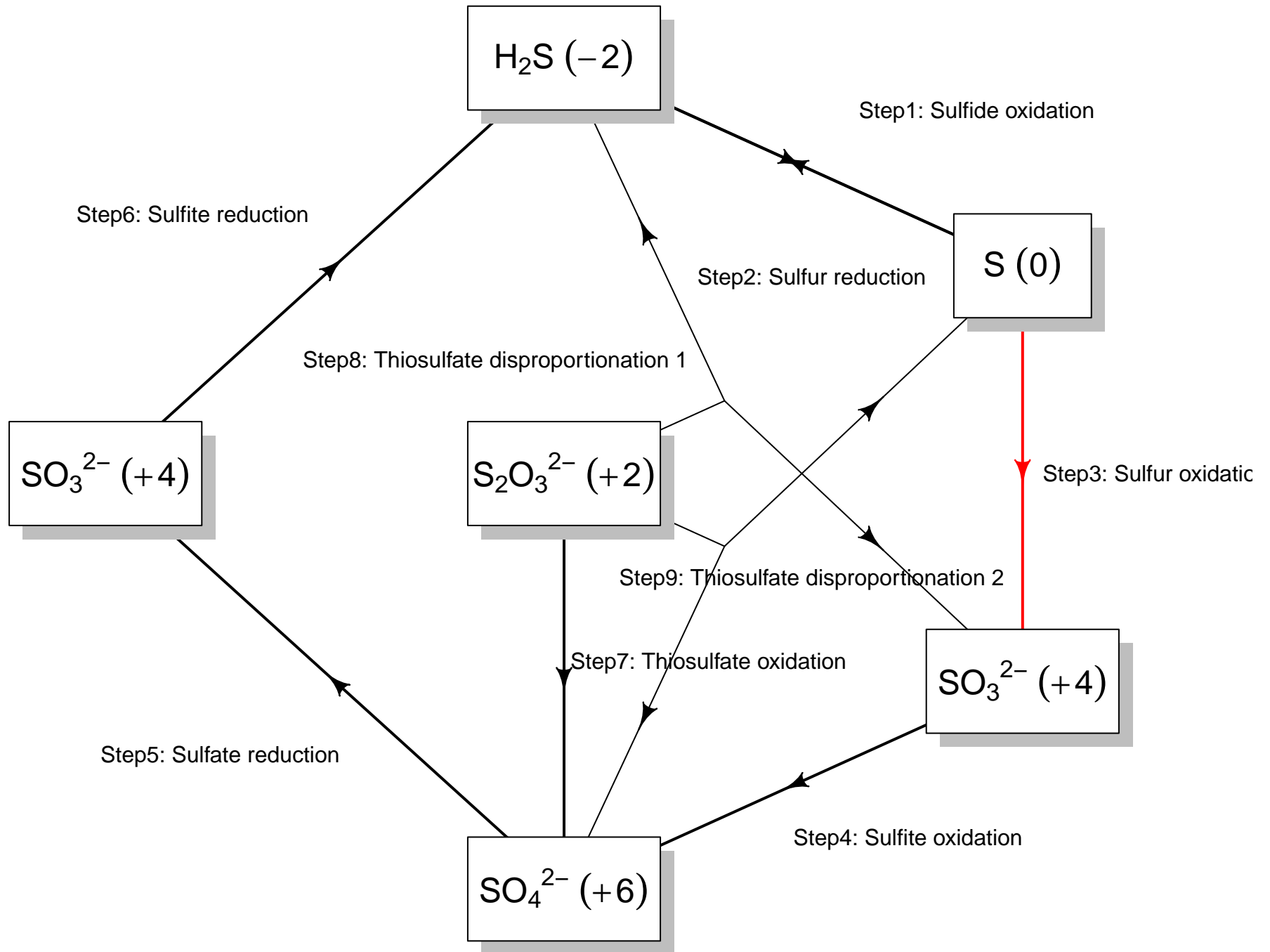

Supplement: Supplementary file 2 — Recovered nutrient cycles including Carbon, Nitrogen, and Sulphur cycles for all the metagenomic assembled genomes given as PDF images, and identifiable through bin numbers. [file 13104_2025_7441_MOESM2_ESM.zip › Supplementary Data2/bin.39.draw_sulfur_cycle_single.pdf]

## Nitrogen Cycle: bin.99

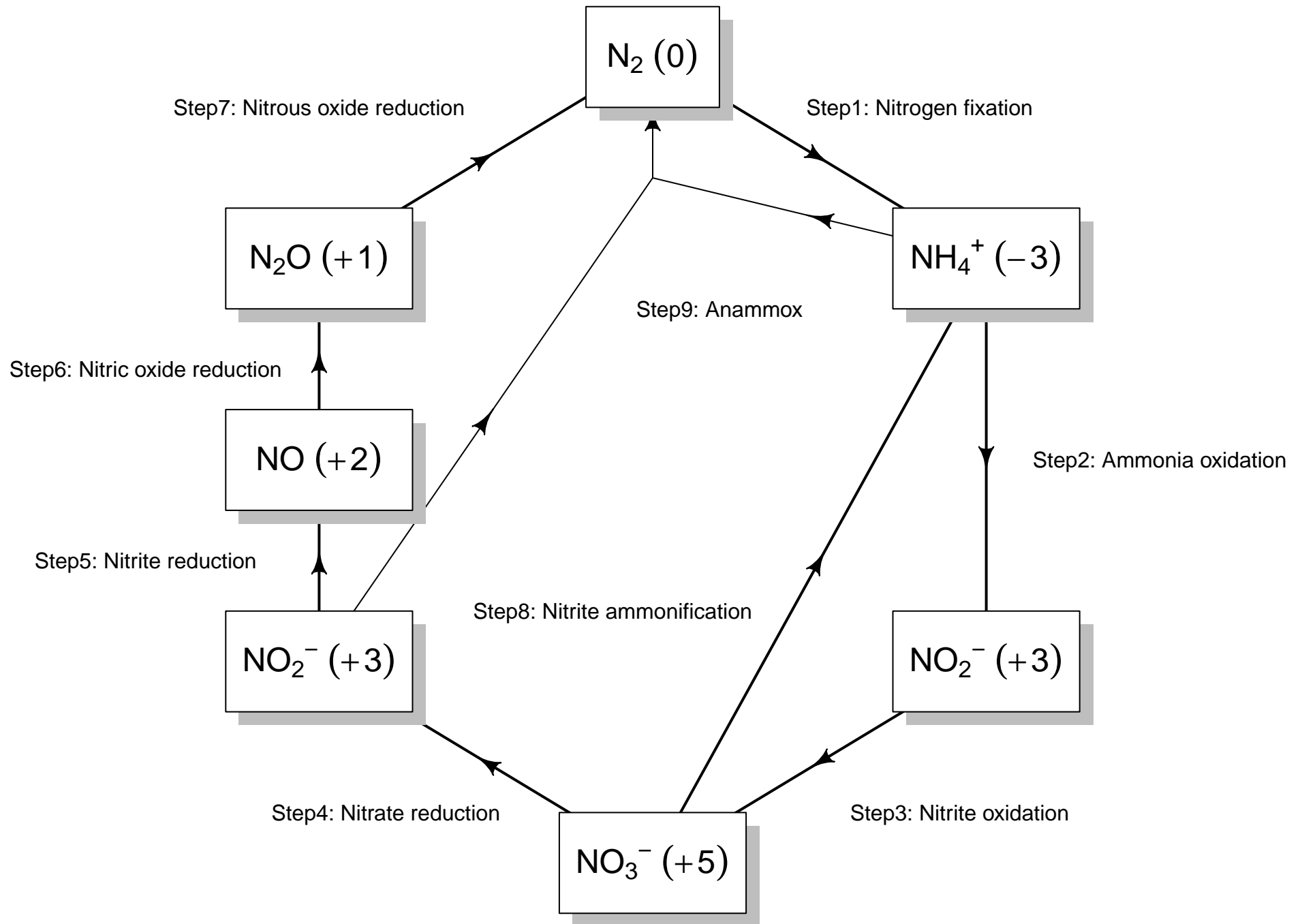

Supplement: Supplementary file 2 — Recovered nutrient cycles including Carbon, Nitrogen, and Sulphur cycles for all the metagenomic assembled genomes given as PDF images, and identifiable through bin numbers. [file 13104_2025_7441_MOESM2_ESM.zip › Supplementary Data2/bin.99.draw_nitrogen_cycle_single.pdf]

## Sulfur Cycle: bin.93

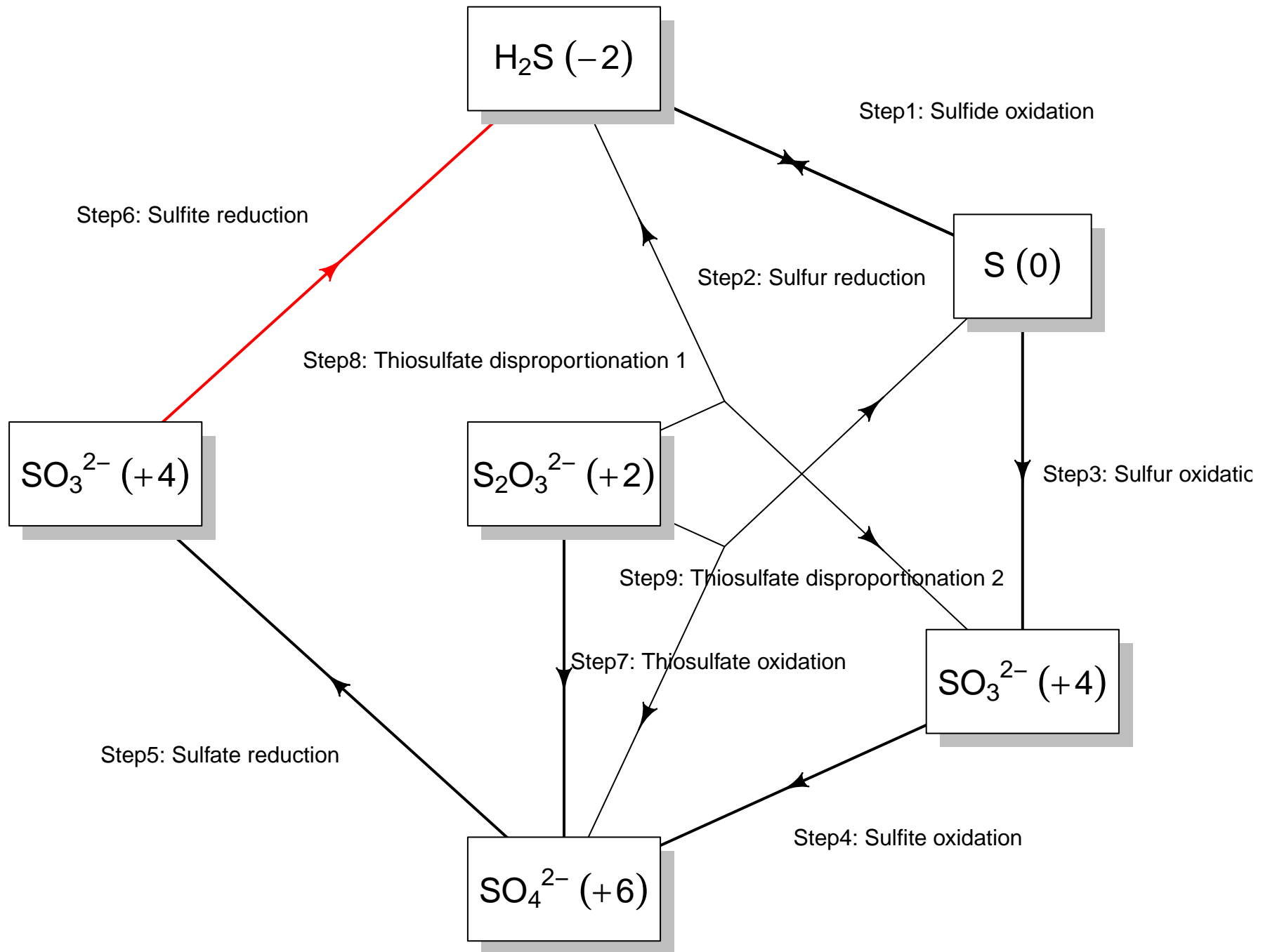

Supplement: Supplementary file 2 — Recovered nutrient cycles including Carbon, Nitrogen, and Sulphur cycles for all the metagenomic assembled genomes given as PDF images, and identifiable through bin numbers. [file 13104_2025_7441_MOESM2_ESM.zip › Supplementary Data2/bin.93.draw_sulfur_cycle_single.pdf]

# Carbon Cycle: bin.115

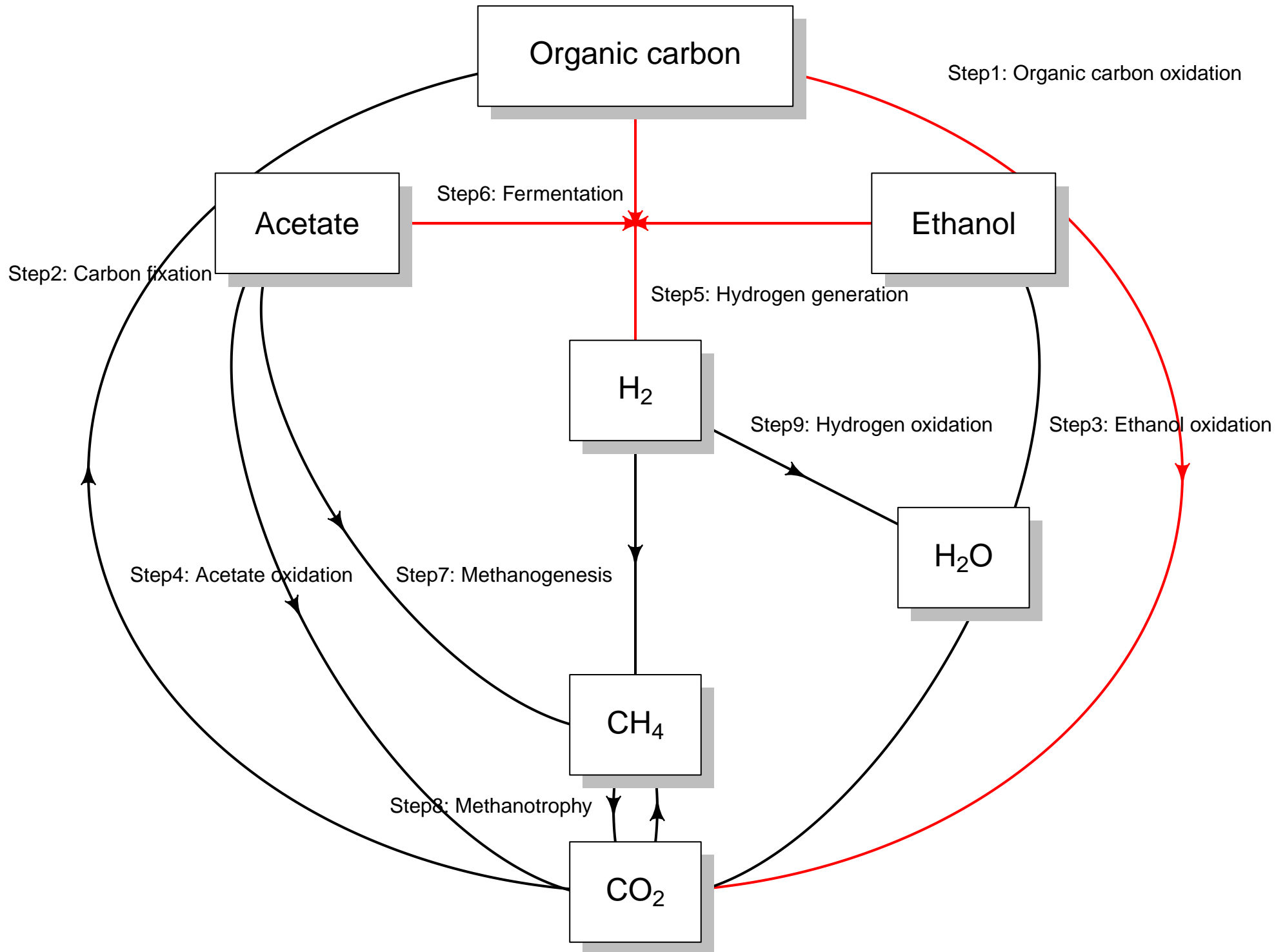

Supplement: Supplementary file 2 — Recovered nutrient cycles including Carbon, Nitrogen, and Sulphur cycles for all the metagenomic assembled genomes given as PDF images, and identifiable through bin numbers. [file 13104_2025_7441_MOESM2_ESM.zip › Supplementary Data2/bin.115.draw_carbon_cycle_single.pdf]

## Sulfur Cycle: bin.138

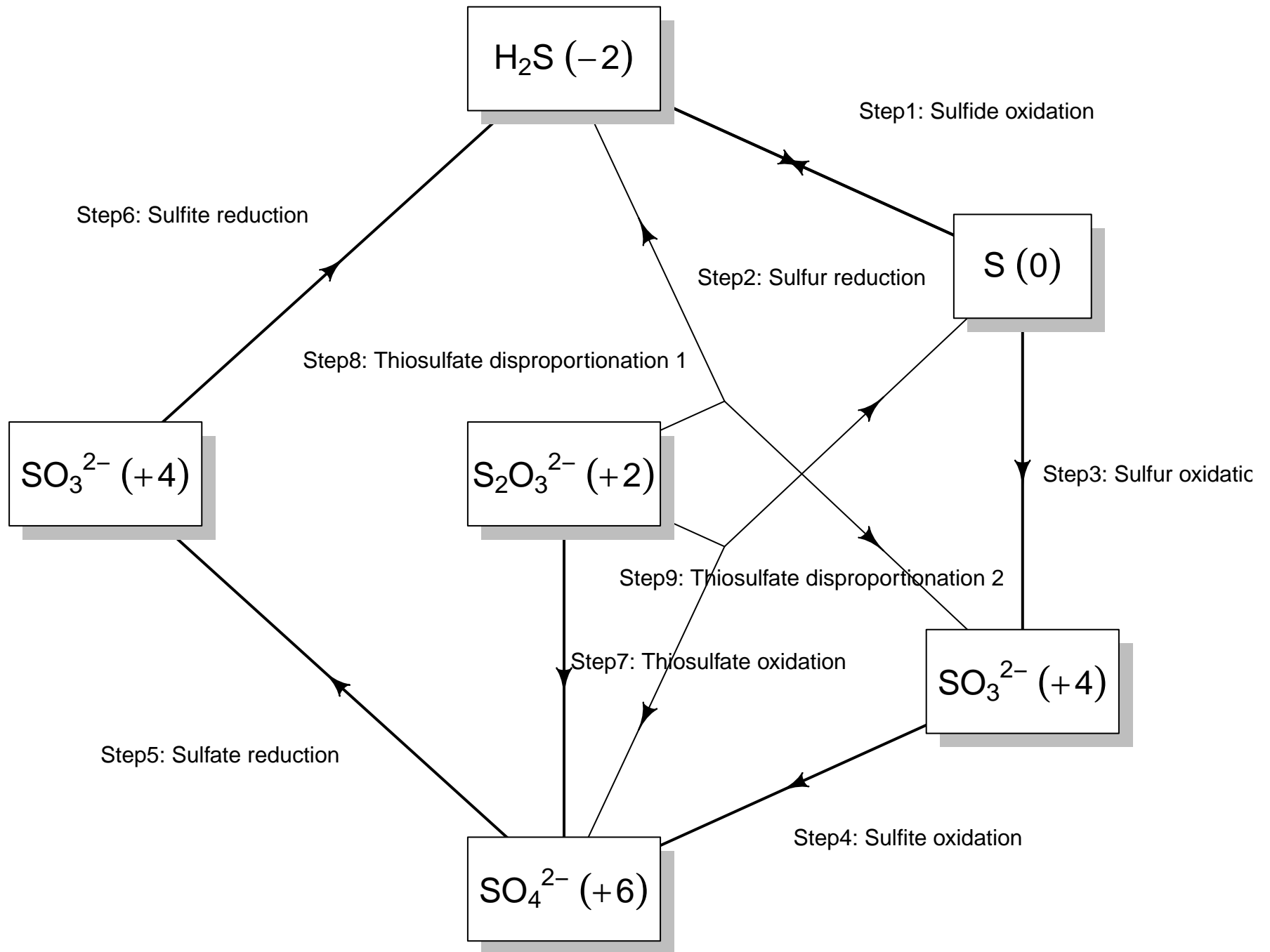

Supplement: Supplementary file 2 — Recovered nutrient cycles including Carbon, Nitrogen, and Sulphur cycles for all the metagenomic assembled genomes given as PDF images, and identifiable through bin numbers. [file 13104_2025_7441_MOESM2_ESM.zip › Supplementary Data2/bin.138.draw_sulfur_cycle_single.pdf]

# Carbon Cycle: bin.28

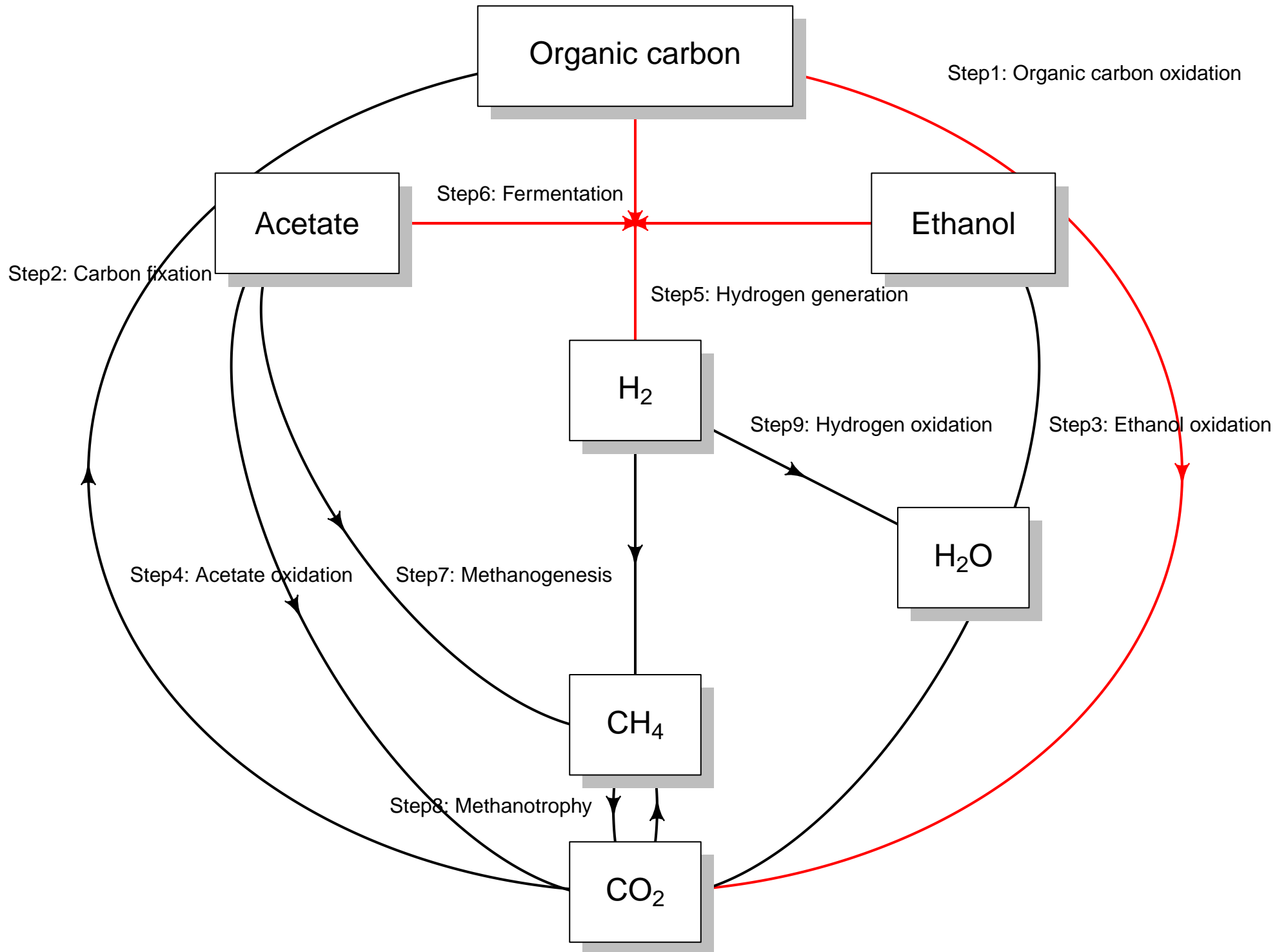

Supplement: Supplementary file 2 — Recovered nutrient cycles including Carbon, Nitrogen, and Sulphur cycles for all the metagenomic assembled genomes given as PDF images, and identifiable through bin numbers. [file 13104_2025_7441_MOESM2_ESM.zip › Supplementary Data2/bin.28.draw_carbon_cycle_single.pdf]

## Sulfur Cycle: bin.5

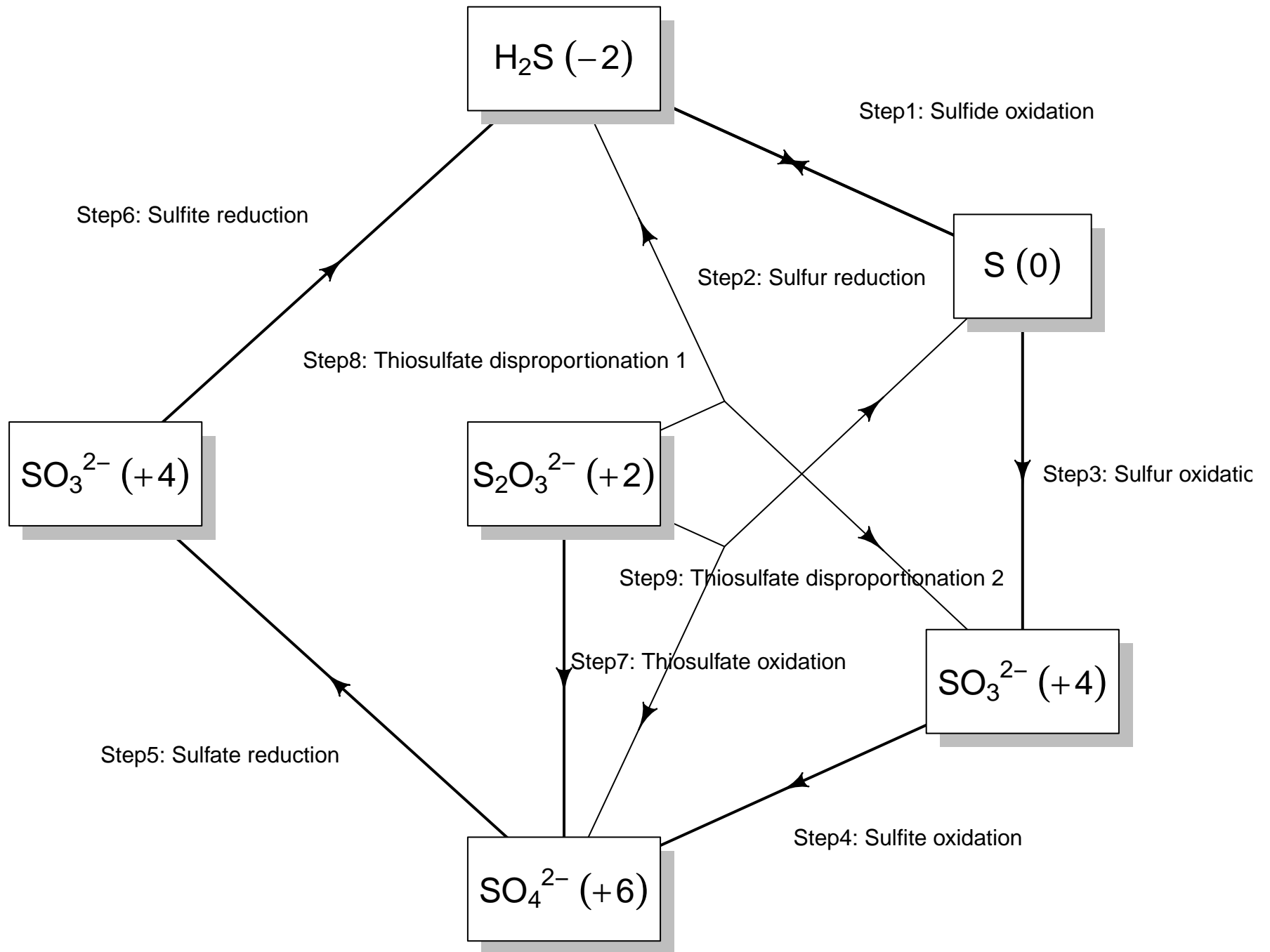

Supplement: Supplementary file 2 — Recovered nutrient cycles including Carbon, Nitrogen, and Sulphur cycles for all the metagenomic assembled genomes given as PDF images, and identifiable through bin numbers. [file 13104_2025_7441_MOESM2_ESM.zip › Supplementary Data2/bin.5.draw_sulfur_cycle_single.pdf]

## Sulfur Cycle: bin.136

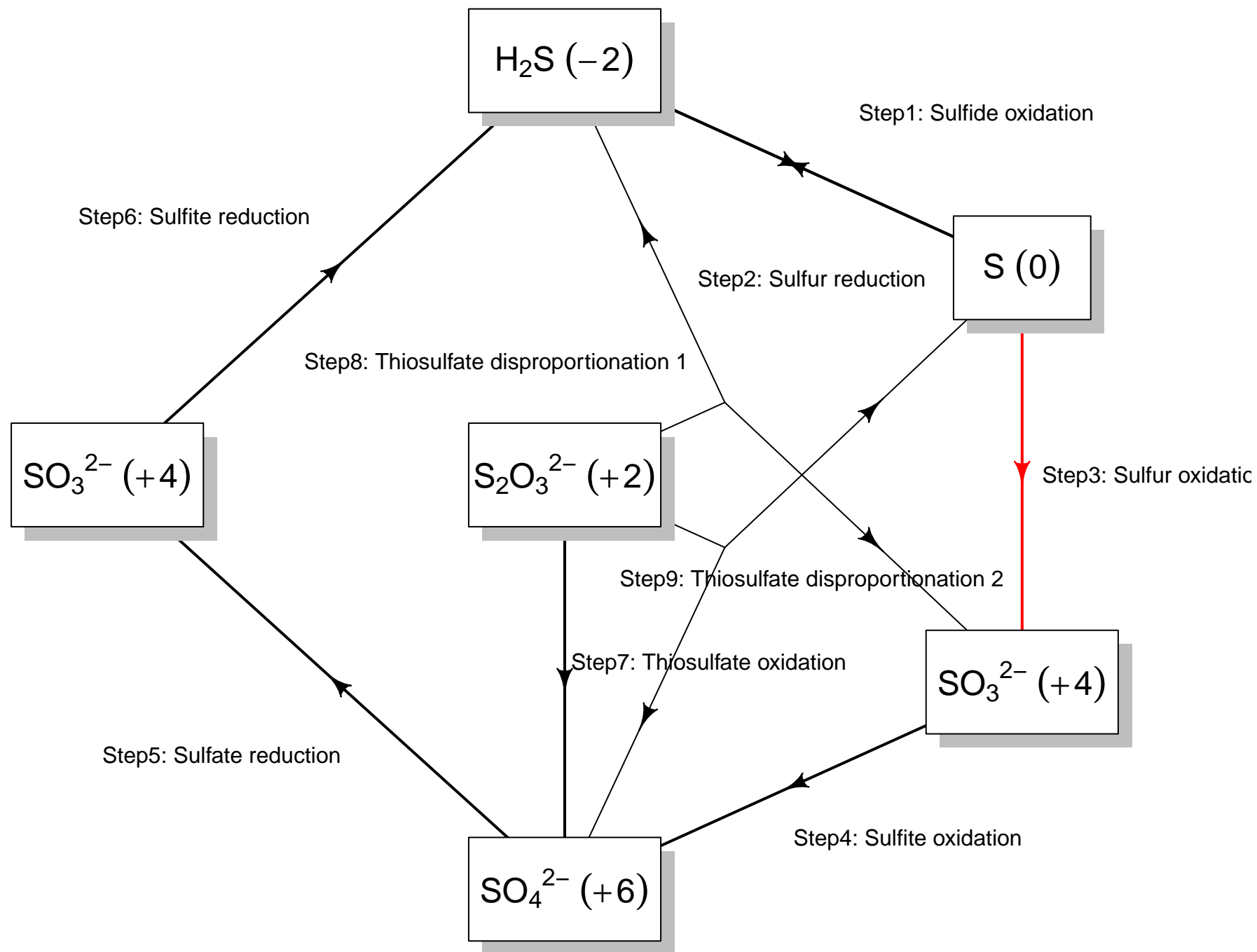

Supplement: Supplementary file 2 — Recovered nutrient cycles including Carbon, Nitrogen, and Sulphur cycles for all the metagenomic assembled genomes given as PDF images, and identifiable through bin numbers. [file 13104_2025_7441_MOESM2_ESM.zip › Supplementary Data2/bin.136.draw_sulfur_cycle_single.pdf]

# Carbon Cycle: bin.26

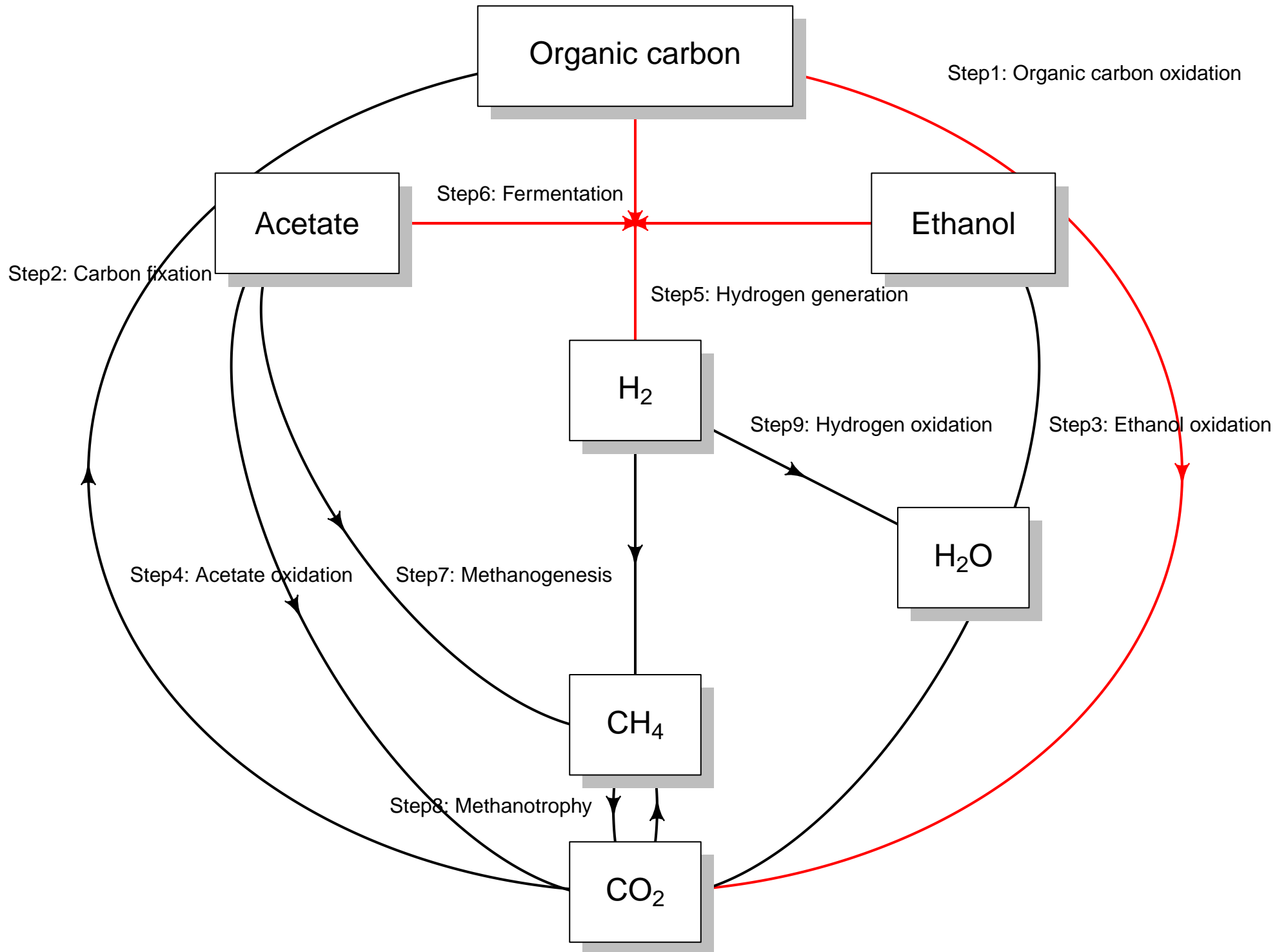

Supplement: Supplementary file 2 — Recovered nutrient cycles including Carbon, Nitrogen, and Sulphur cycles for all the metagenomic assembled genomes given as PDF images, and identifiable through bin numbers. [file 13104_2025_7441_MOESM2_ESM.zip › Supplementary Data2/bin.26.draw_carbon_cycle_single.pdf]

## Nitrogen Cycle: bin.136

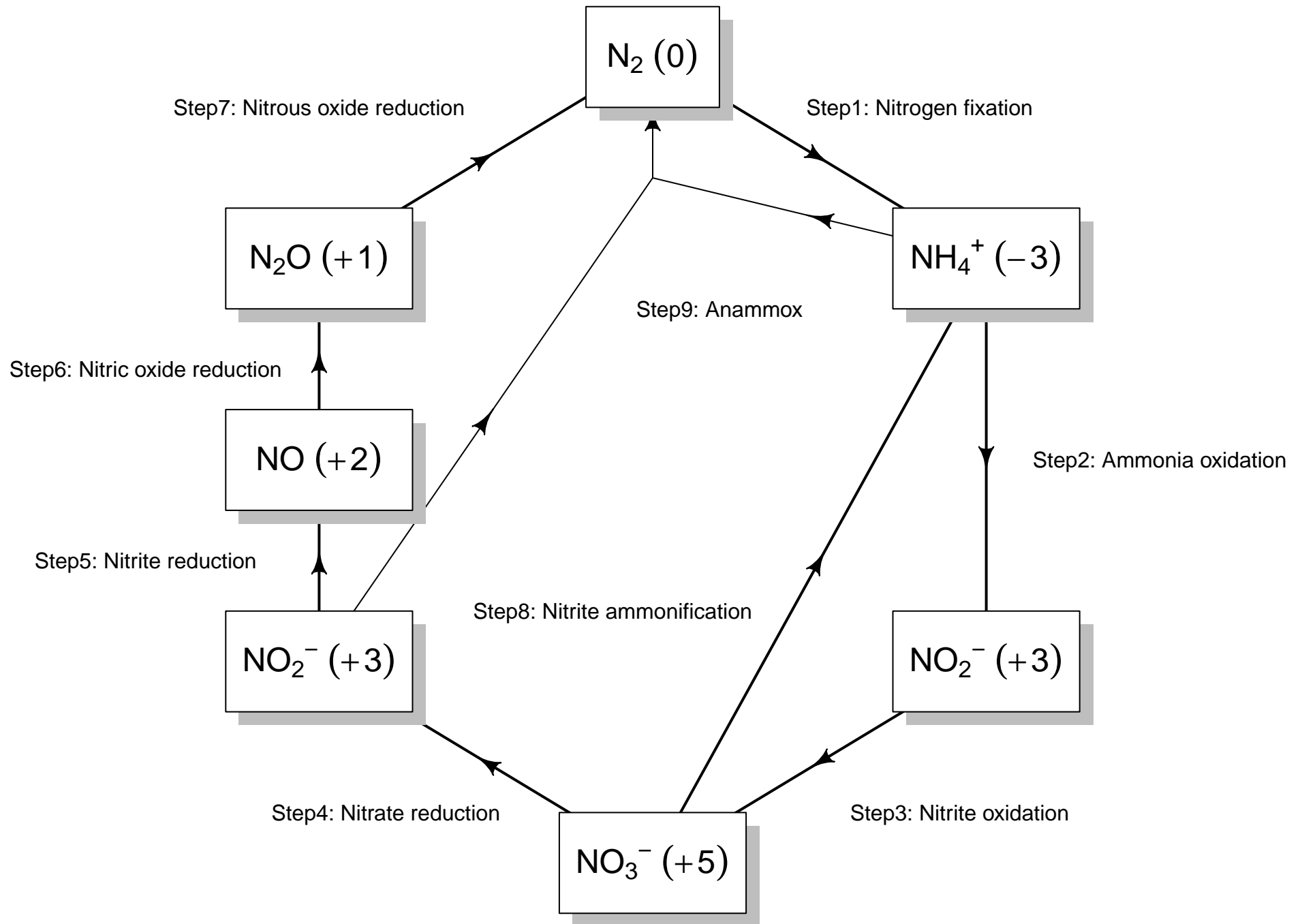

Supplement: Supplementary file 2 — Recovered nutrient cycles including Carbon, Nitrogen, and Sulphur cycles for all the metagenomic assembled genomes given as PDF images, and identifiable through bin numbers. [file 13104_2025_7441_MOESM2_ESM.zip › Supplementary Data2/bin.136.draw_nitrogen_cycle_single.pdf]

# Carbon Cycle: bin.33

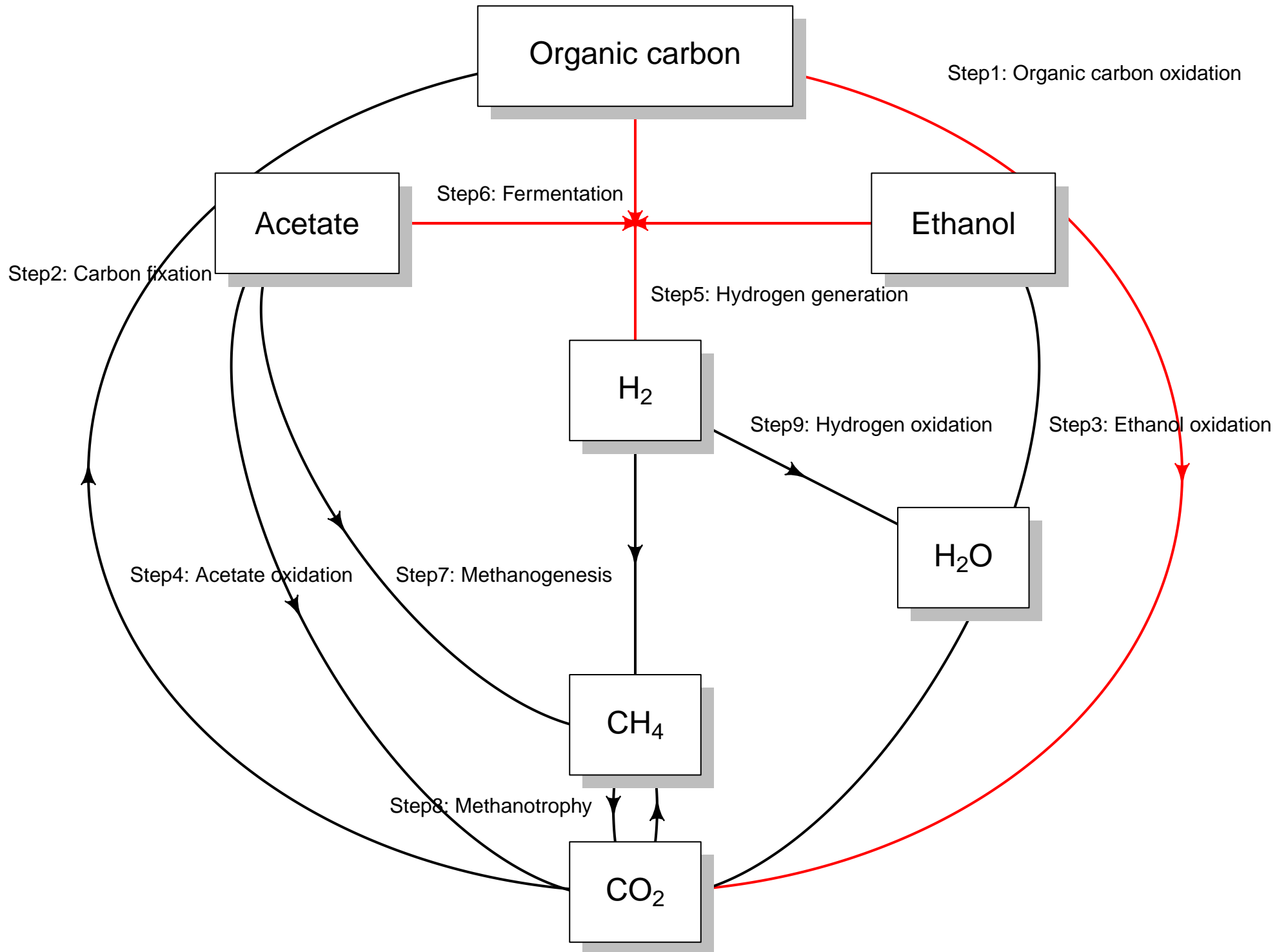

Supplement: Supplementary file 2 — Recovered nutrient cycles including Carbon, Nitrogen, and Sulphur cycles for all the metagenomic assembled genomes given as PDF images, and identifiable through bin numbers. [file 13104_2025_7441_MOESM2_ESM.zip › Supplementary Data2/bin.33.draw_carbon_cycle_single.pdf]

## Sulfur Cycle: bin.123

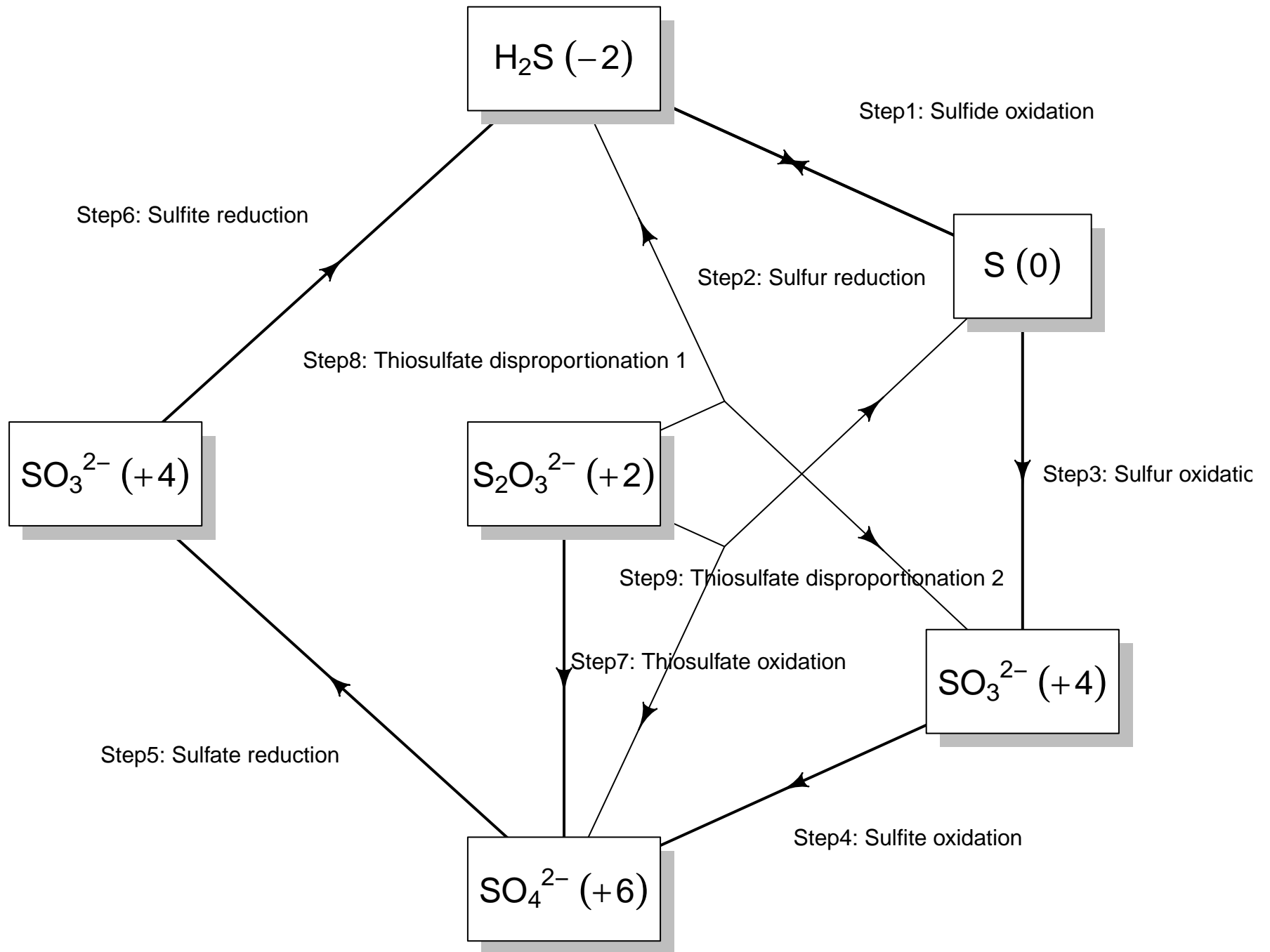

Supplement: Supplementary file 2 — Recovered nutrient cycles including Carbon, Nitrogen, and Sulphur cycles for all the metagenomic assembled genomes given as PDF images, and identifiable through bin numbers. [file 13104_2025_7441_MOESM2_ESM.zip › Supplementary Data2/bin.123.draw_sulfur_cycle_single.pdf]

# Nitrogen Cycle: bin.61

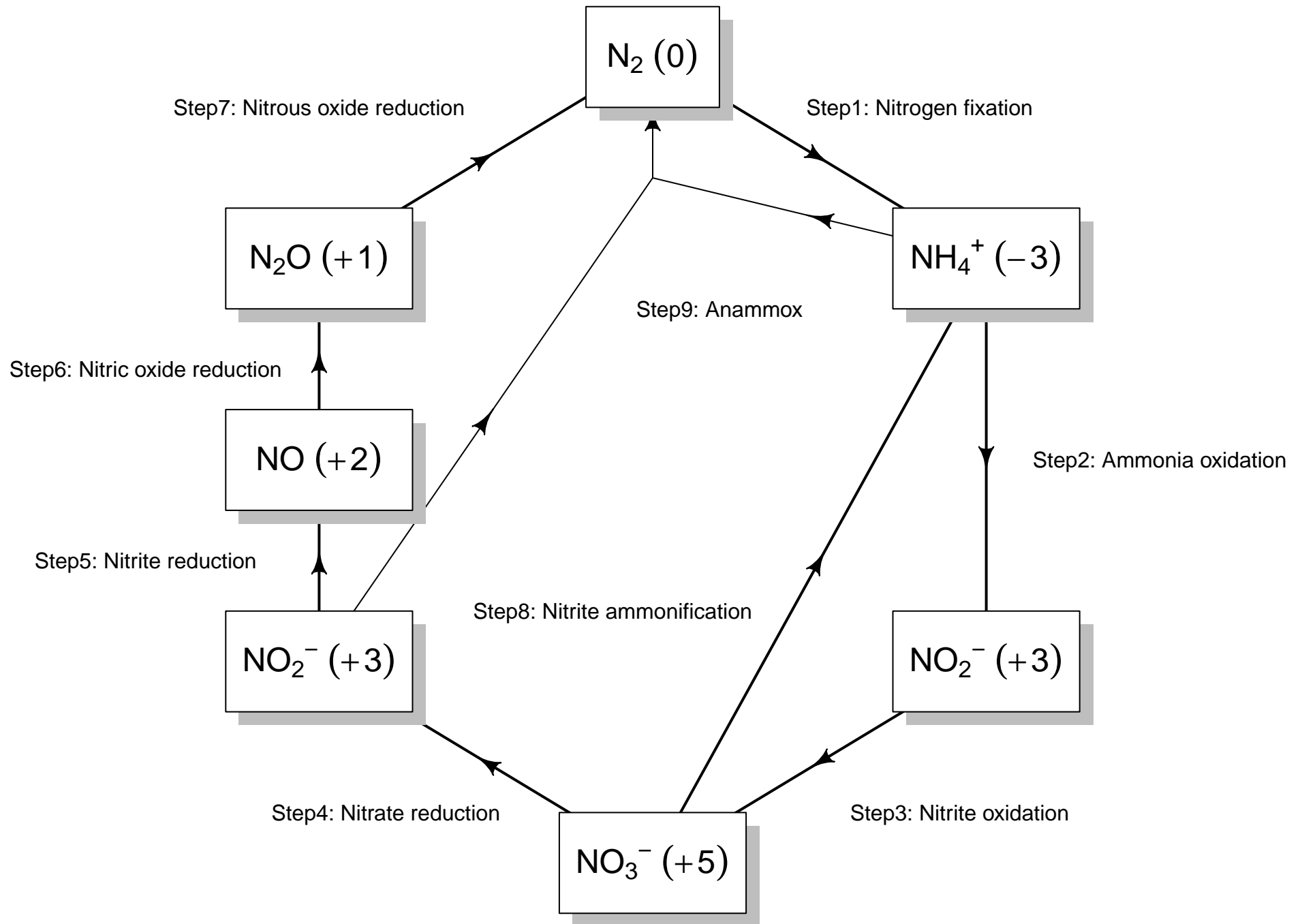

Supplement: Supplementary file 2 — Recovered nutrient cycles including Carbon, Nitrogen, and Sulphur cycles for all the metagenomic assembled genomes given as PDF images, and identifiable through bin numbers. [file 13104_2025_7441_MOESM2_ESM.zip › Supplementary Data2/bin.61.draw_nitrogen_cycle_single.pdf]

# Carbon Cycle: bin.100

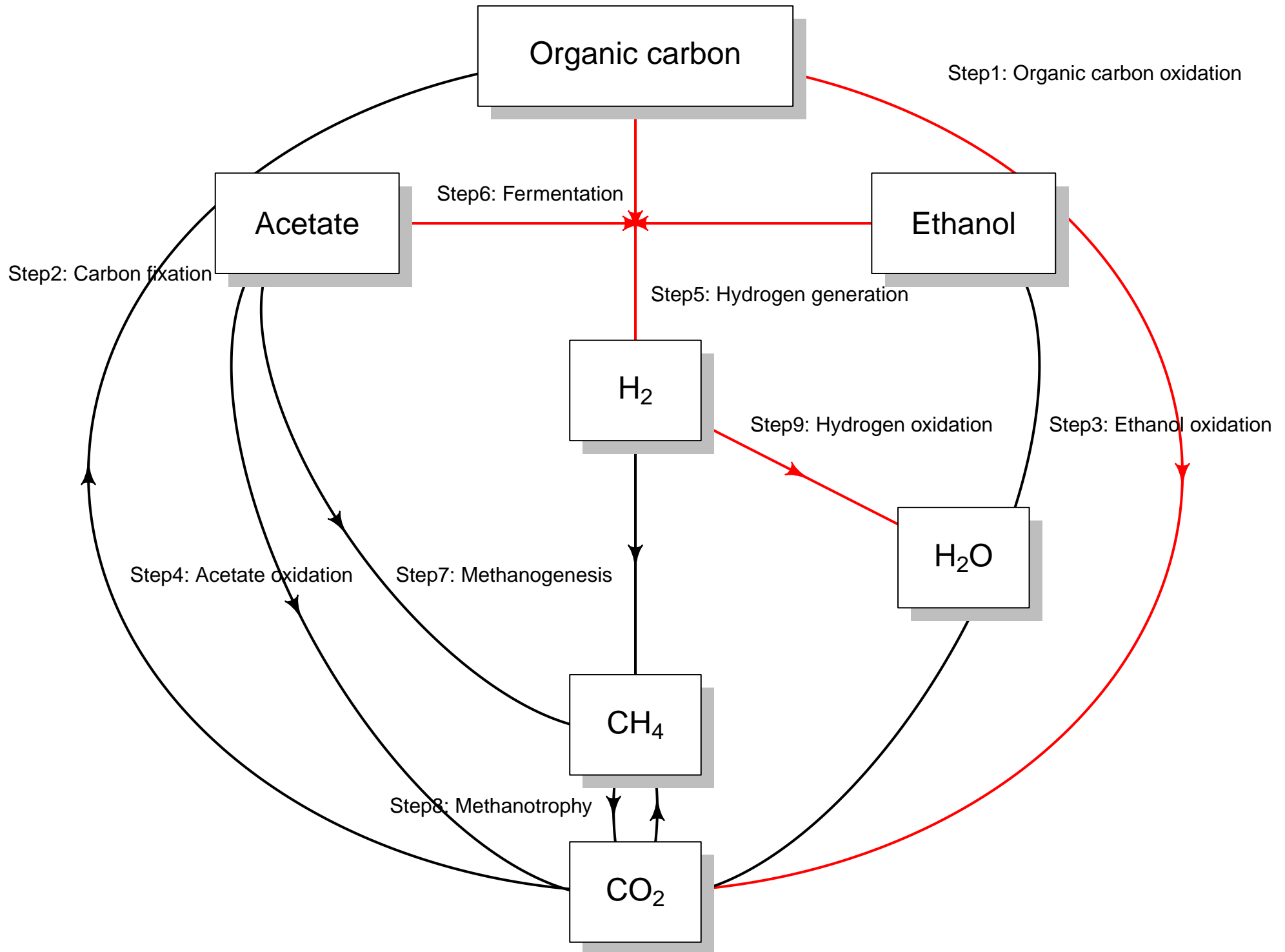

Supplement: Supplementary file 2 — Recovered nutrient cycles including Carbon, Nitrogen, and Sulphur cycles for all the metagenomic assembled genomes given as PDF images, and identifiable through bin numbers. [file 13104_2025_7441_MOESM2_ESM.zip › Supplementary Data2/bin.100.draw_carbon_cycle_single.pdf]

## Sulfur Cycle: bin.88

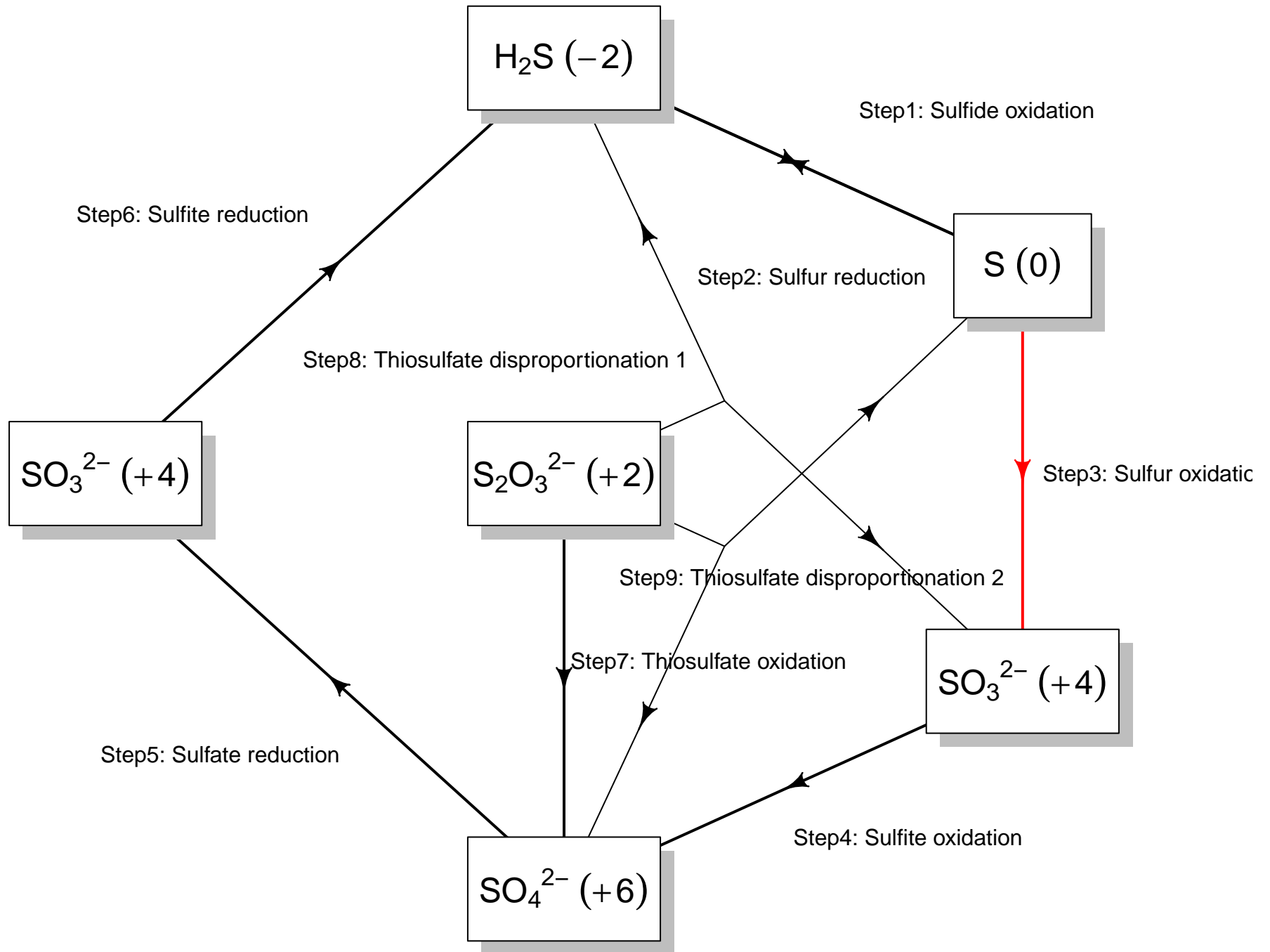

Supplement: Supplementary file 2 — Recovered nutrient cycles including Carbon, Nitrogen, and Sulphur cycles for all the metagenomic assembled genomes given as PDF images, and identifiable through bin numbers. [file 13104_2025_7441_MOESM2_ESM.zip › Supplementary Data2/bin.88.draw_sulfur_cycle_single.pdf]

## Sulfur Cycle: bin.10

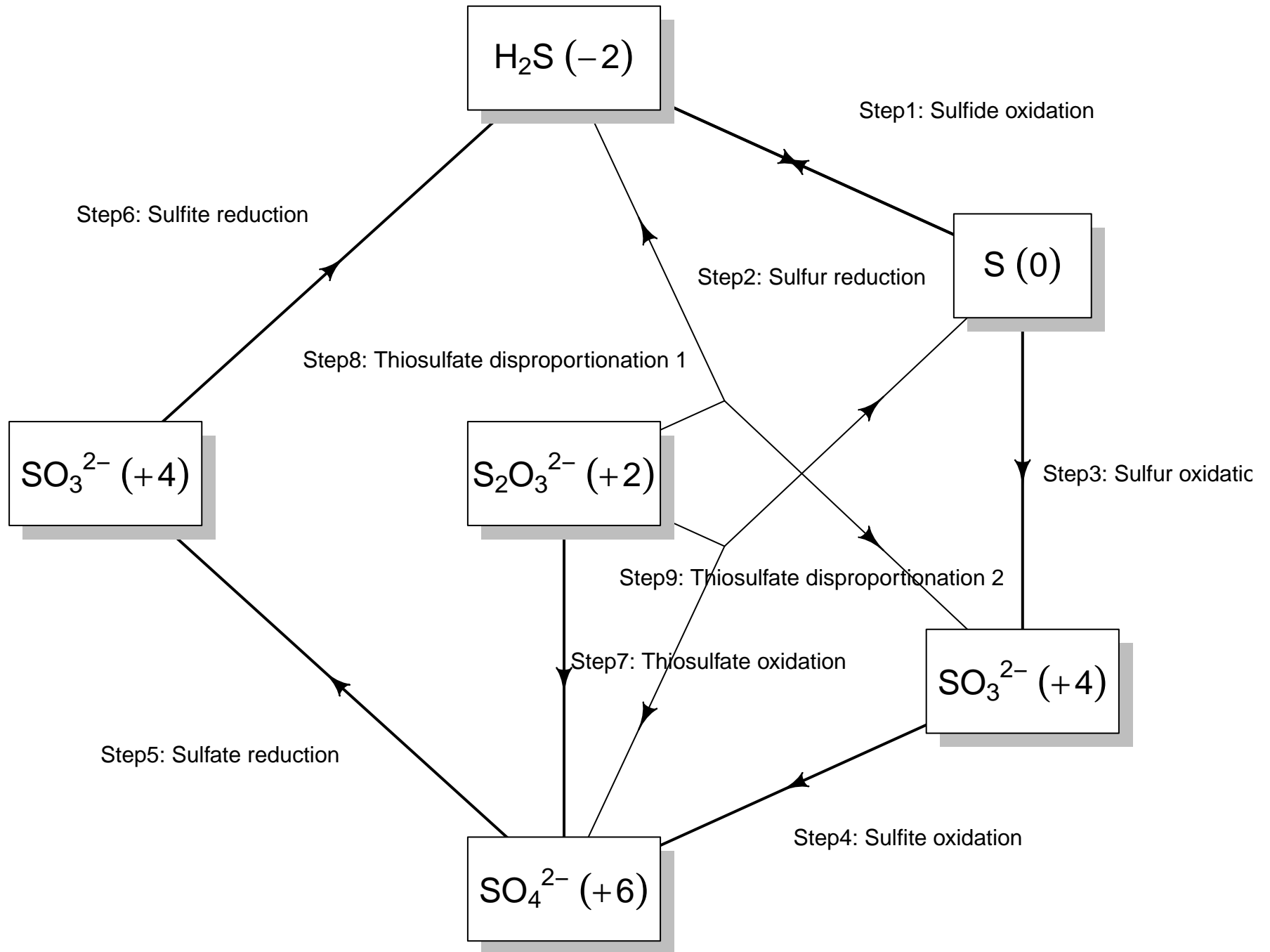

Supplement: Supplementary file 2 — Recovered nutrient cycles including Carbon, Nitrogen, and Sulphur cycles for all the metagenomic assembled genomes given as PDF images, and identifiable through bin numbers. [file 13104_2025_7441_MOESM2_ESM.zip › Supplementary Data2/bin.10.draw_sulfur_cycle_single.pdf]

## Sulfur Cycle: bin.86

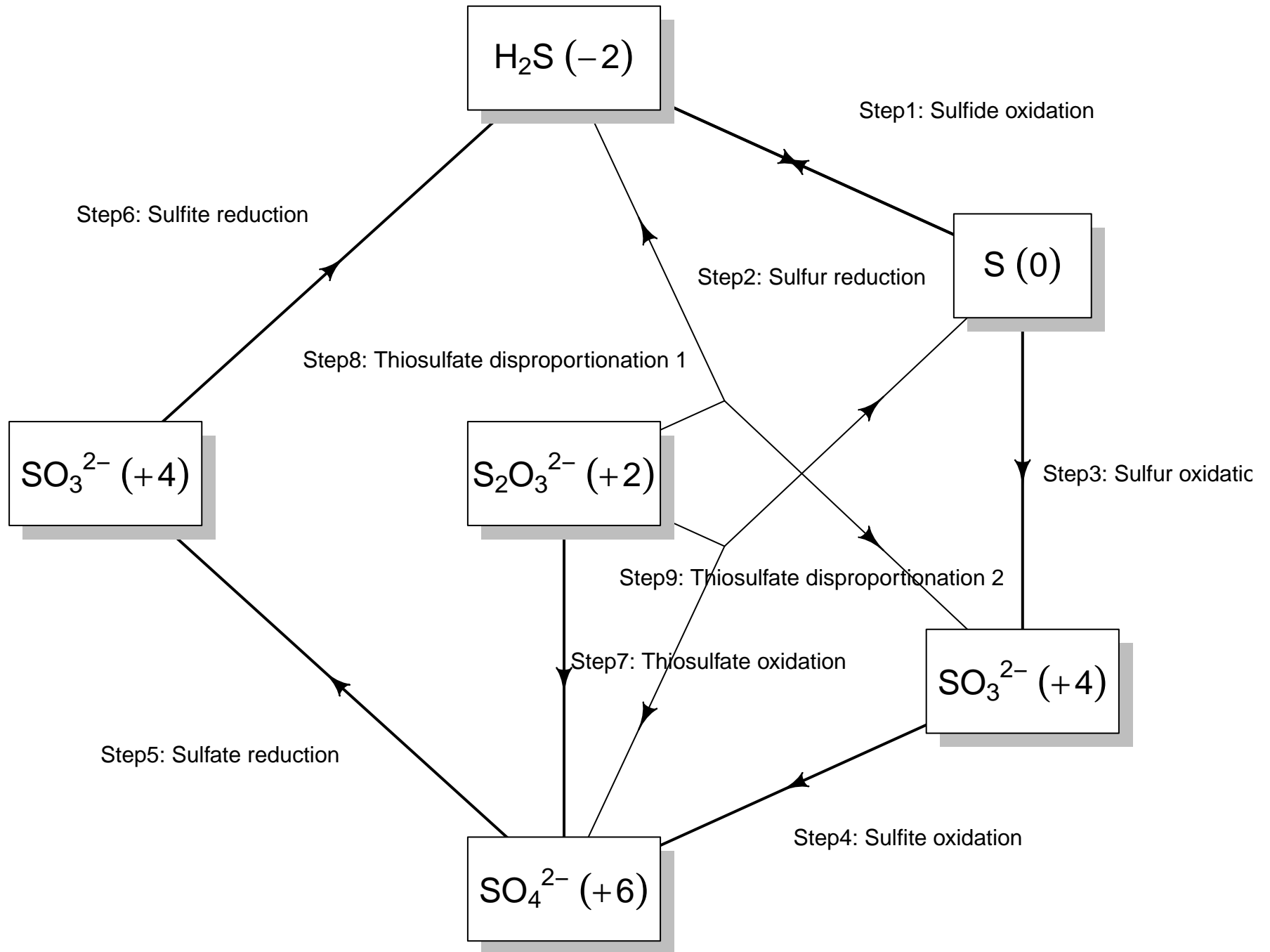

Supplement: Supplementary file 2 — Recovered nutrient cycles including Carbon, Nitrogen, and Sulphur cycles for all the metagenomic assembled genomes given as PDF images, and identifiable through bin numbers. [file 13104_2025_7441_MOESM2_ESM.zip › Supplementary Data2/bin.86.draw_sulfur_cycle_single.pdf]

## Nitrogen Cycle: bin.125

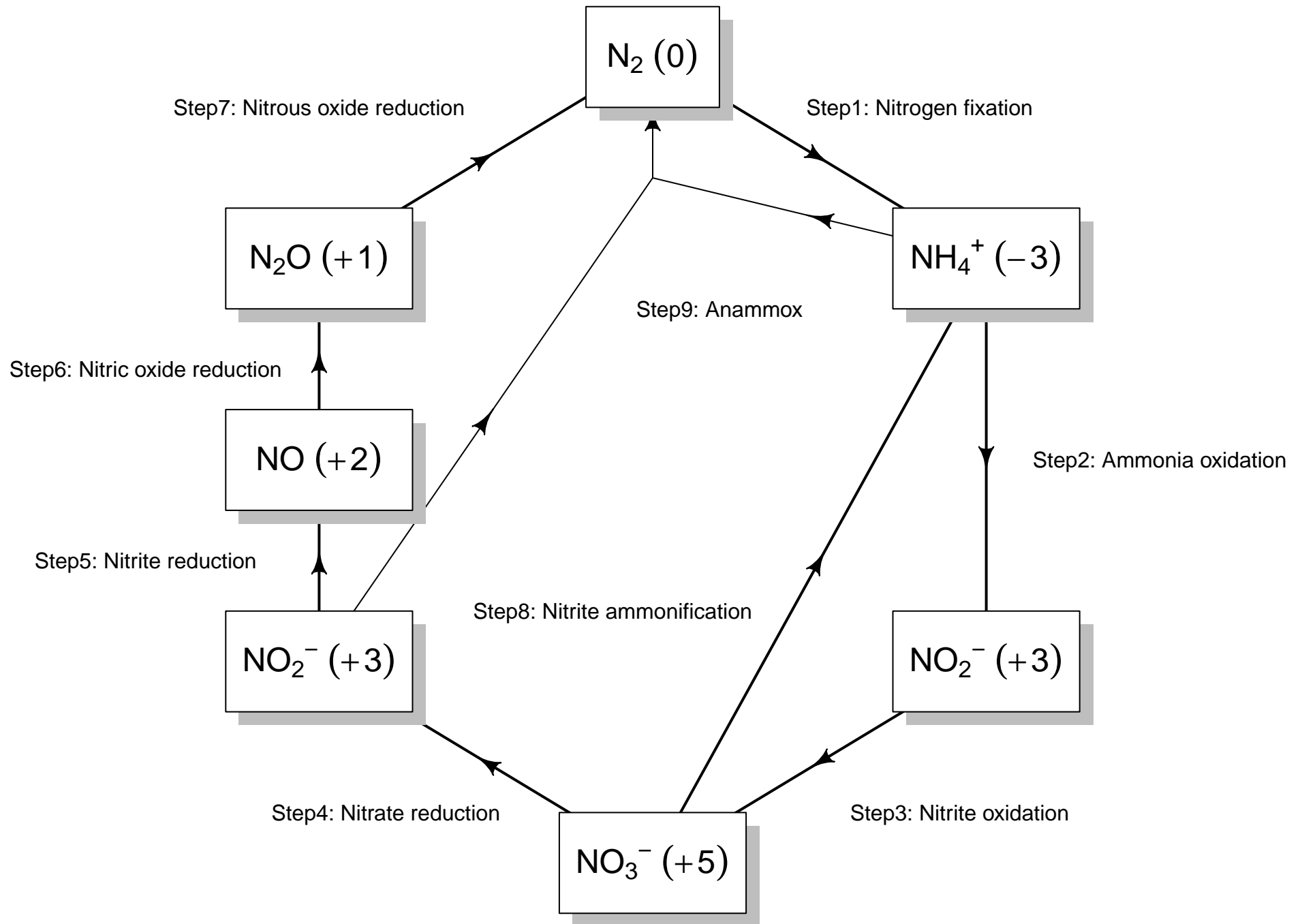

Supplement: Supplementary file 2 — Recovered nutrient cycles including Carbon, Nitrogen, and Sulphur cycles for all the metagenomic assembled genomes given as PDF images, and identifiable through bin numbers. [file 13104_2025_7441_MOESM2_ESM.zip › Supplementary Data2/bin.125.draw_nitrogen_cycle_single.pdf]

## Nitrogen Cycle: bin.2

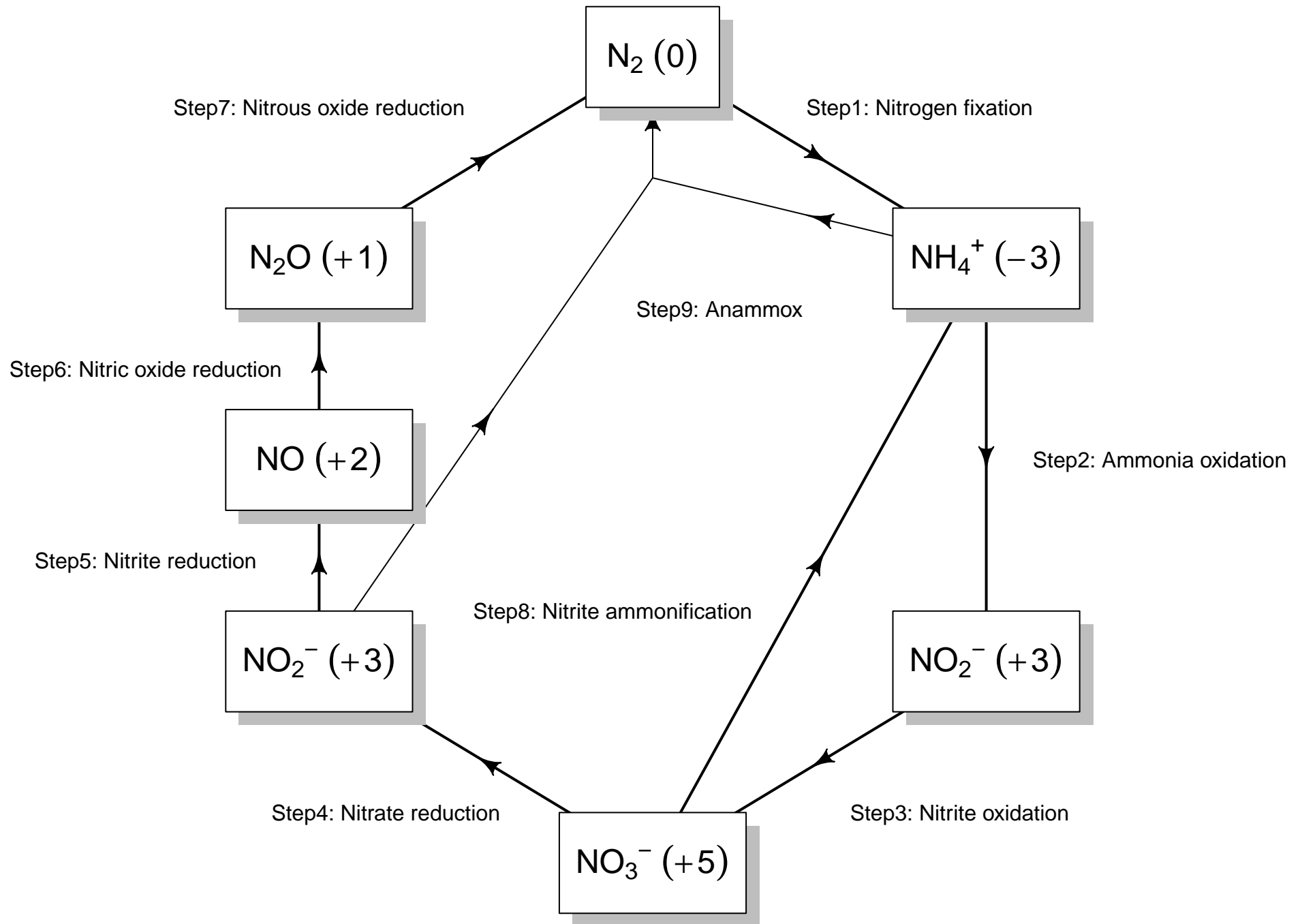

Supplement: Supplementary file 2 — Recovered nutrient cycles including Carbon, Nitrogen, and Sulphur cycles for all the metagenomic assembled genomes given as PDF images, and identifiable through bin numbers. [file 13104_2025_7441_MOESM2_ESM.zip › Supplementary Data2/bin.2.draw_nitrogen_cycle_single.pdf]

## Sulfur Cycle: bin.34

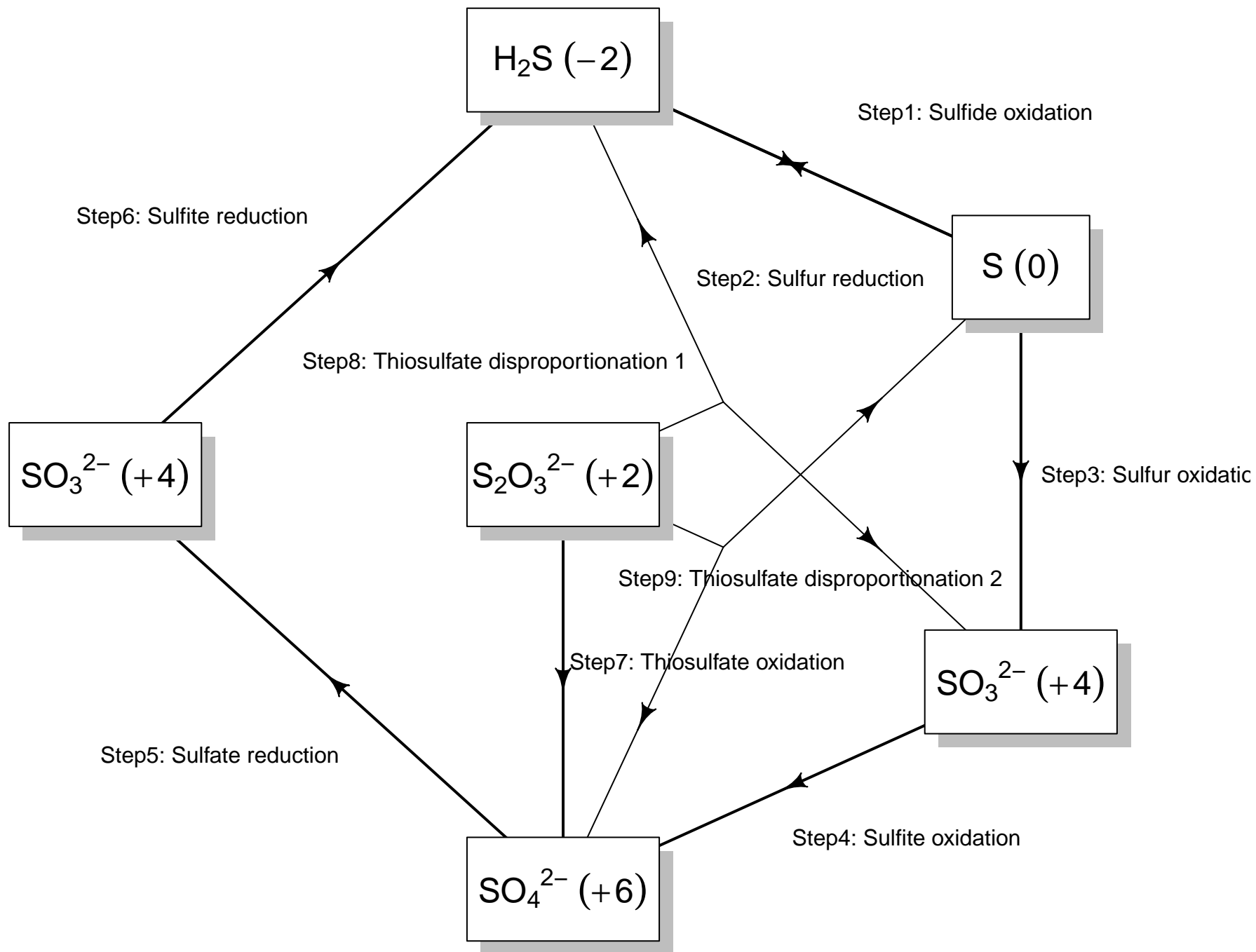

Supplement: Supplementary file 2 — Recovered nutrient cycles including Carbon, Nitrogen, and Sulphur cycles for all the metagenomic assembled genomes given as PDF images, and identifiable through bin numbers. [file 13104_2025_7441_MOESM2_ESM.zip › Supplementary Data2/bin.34.draw_sulfur_cycle_single.pdf]

# Carbon Cycle: bin.124

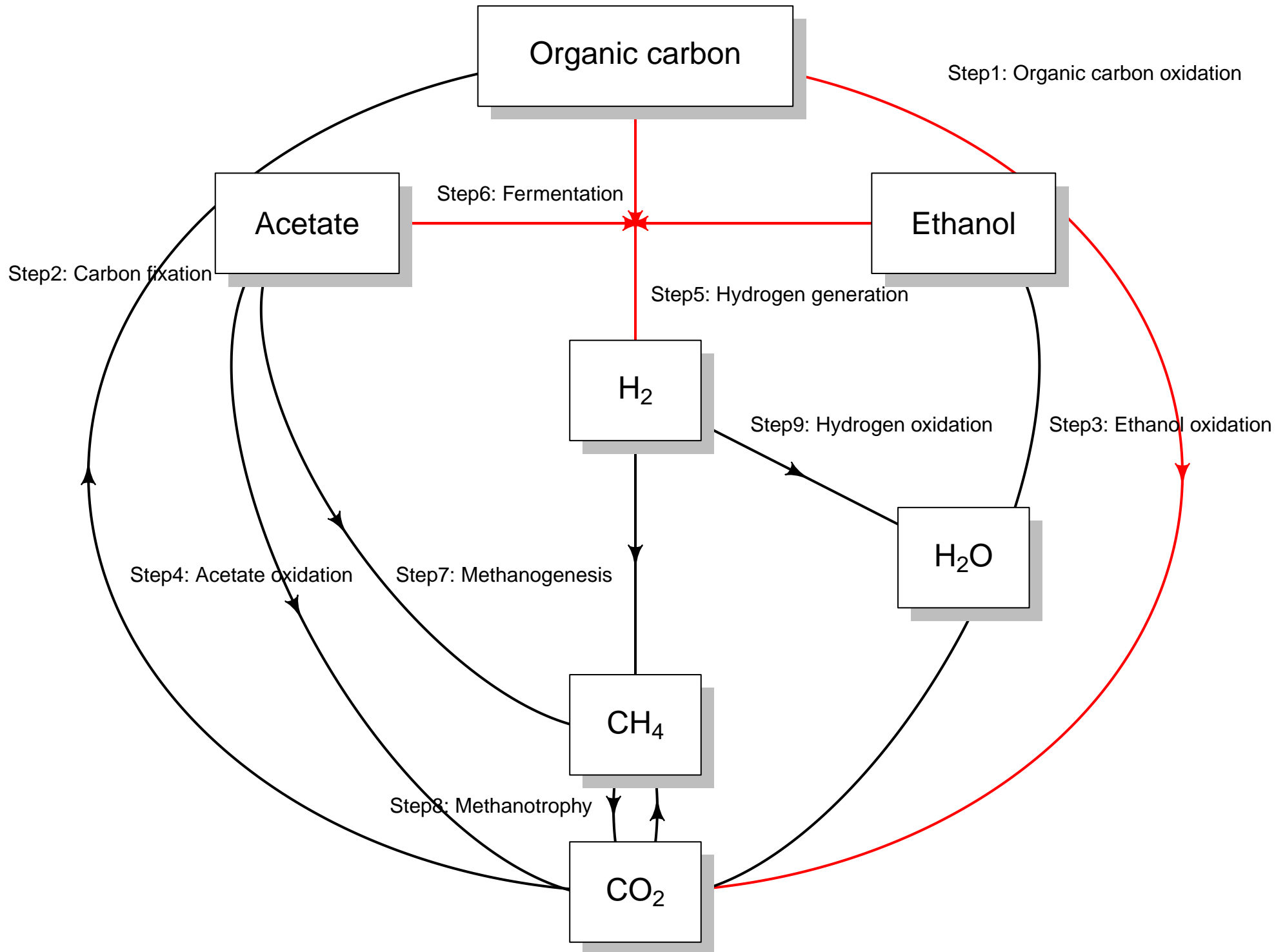

Supplement: Supplementary file 2 — Recovered nutrient cycles including Carbon, Nitrogen, and Sulphur cycles for all the metagenomic assembled genomes given as PDF images, and identifiable through bin numbers. [file 13104_2025_7441_MOESM2_ESM.zip › Supplementary Data2/bin.124.draw_carbon_cycle_single.pdf]

## Sulfur Cycle: bin.107

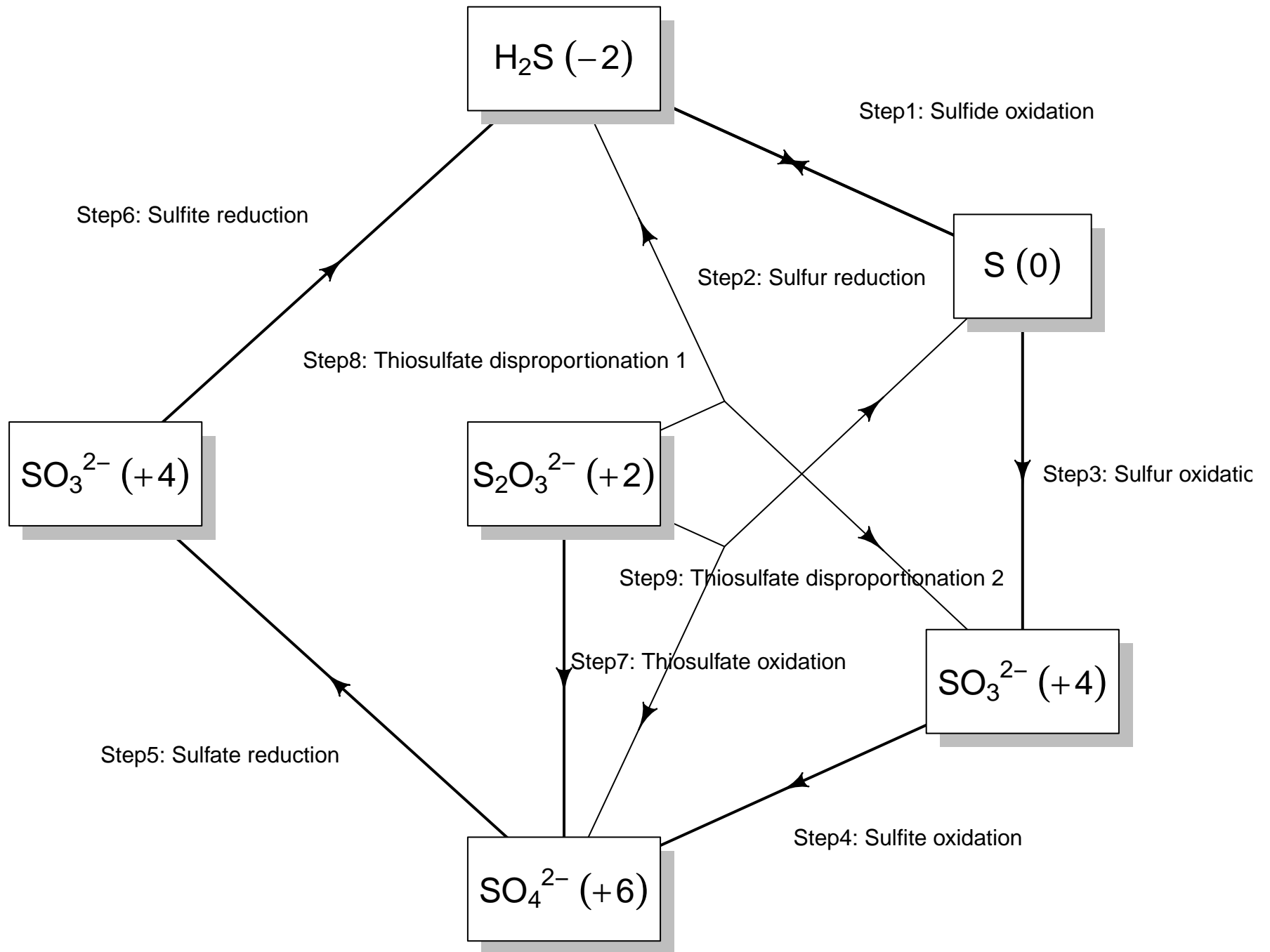

Supplement: Supplementary file 2 — Recovered nutrient cycles including Carbon, Nitrogen, and Sulphur cycles for all the metagenomic assembled genomes given as PDF images, and identifiable through bin numbers. [file 13104_2025_7441_MOESM2_ESM.zip › Supplementary Data2/bin.107.draw_sulfur_cycle_single.pdf]

# Carbon Cycle: bin.17

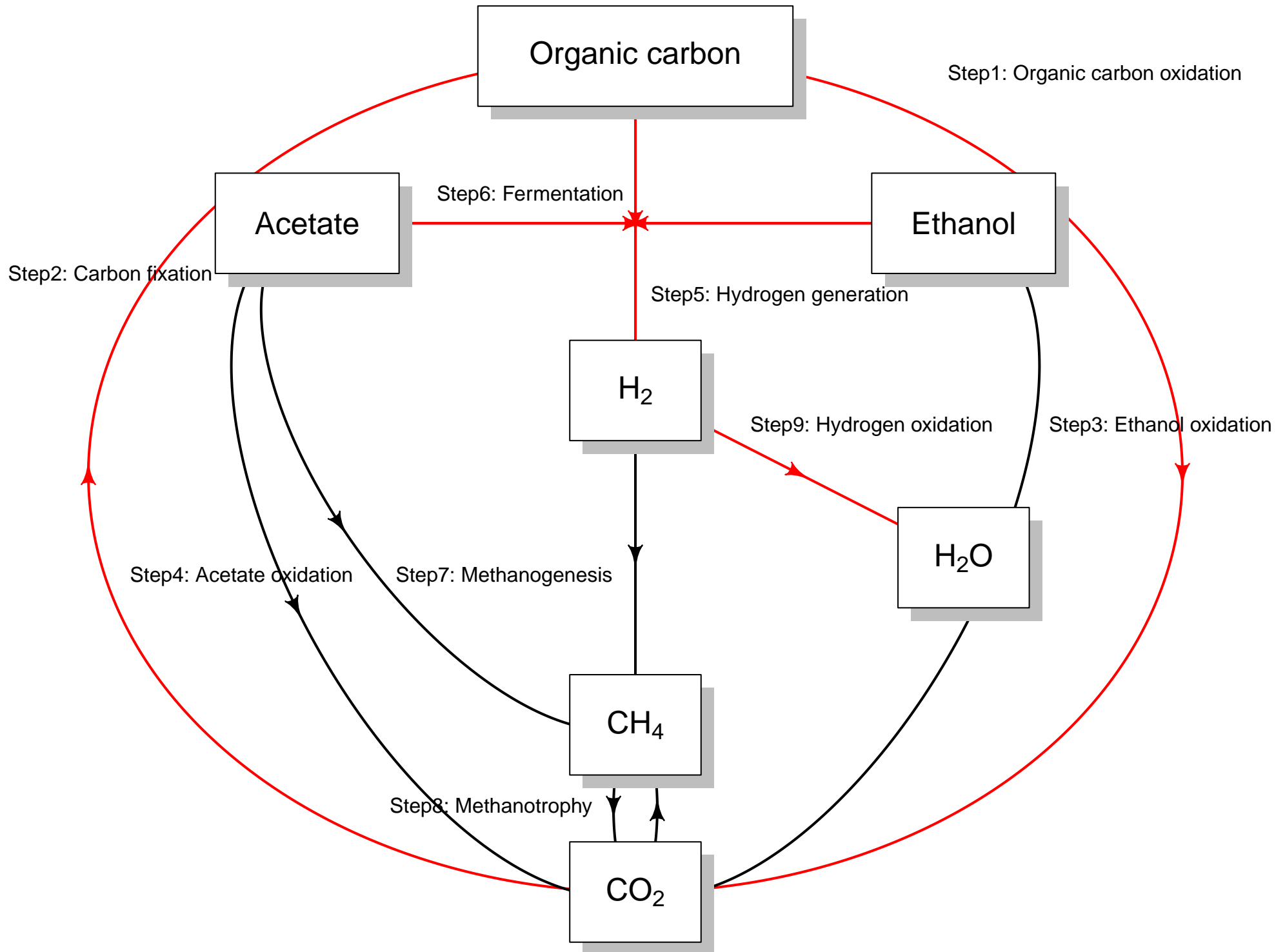

Supplement: Supplementary file 2 — Recovered nutrient cycles including Carbon, Nitrogen, and Sulphur cycles for all the metagenomic assembled genomes given as PDF images, and identifiable through bin numbers. [file 13104_2025_7441_MOESM2_ESM.zip › Supplementary Data2/bin.17.draw_carbon_cycle_single.pdf]

## Sulfur Cycle: bin.109

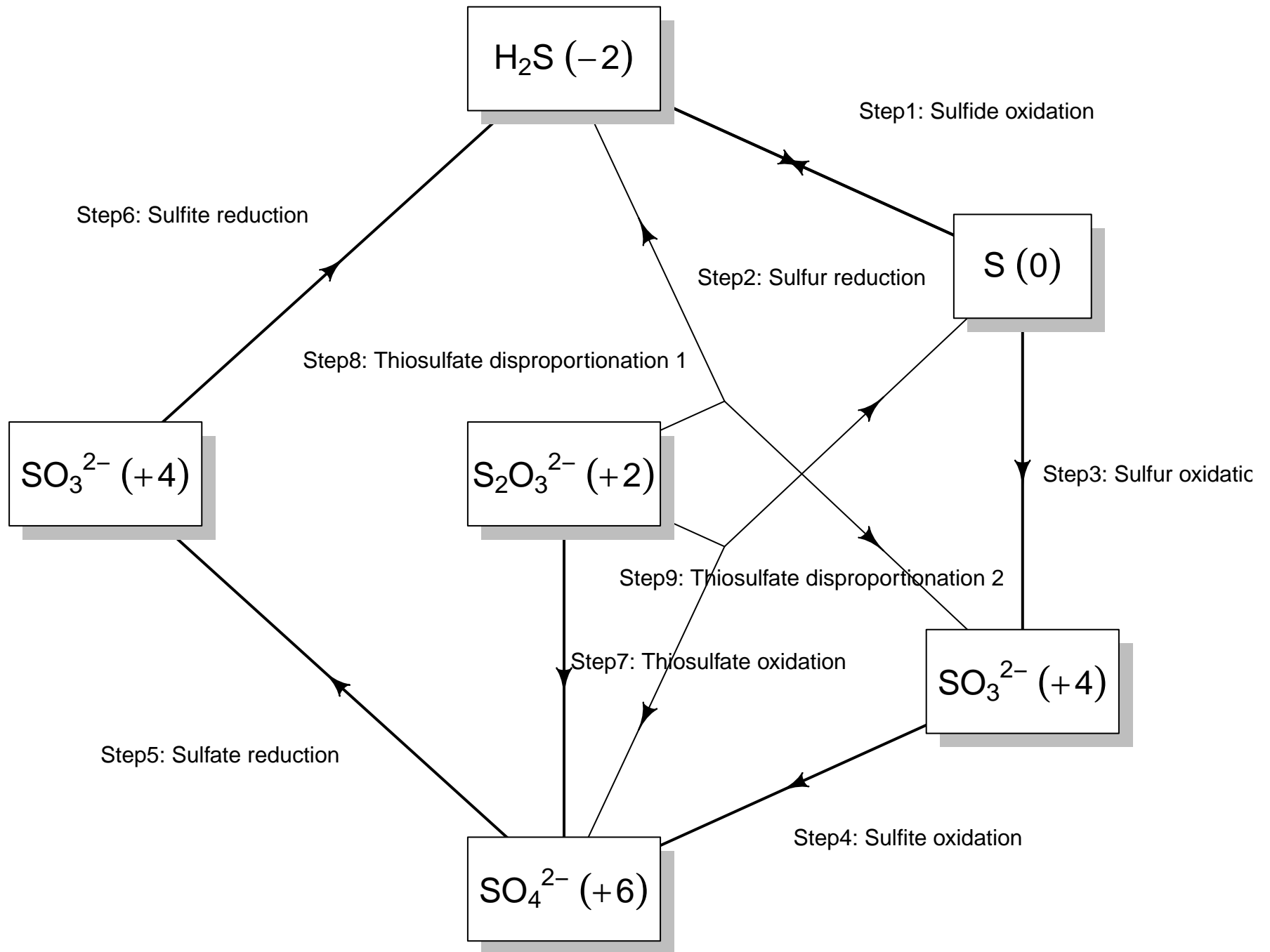

Supplement: Supplementary file 2 — Recovered nutrient cycles including Carbon, Nitrogen, and Sulphur cycles for all the metagenomic assembled genomes given as PDF images, and identifiable through bin numbers. [file 13104_2025_7441_MOESM2_ESM.zip › Supplementary Data2/bin.109.draw_sulfur_cycle_single.pdf]

# Carbon Cycle: bin.19

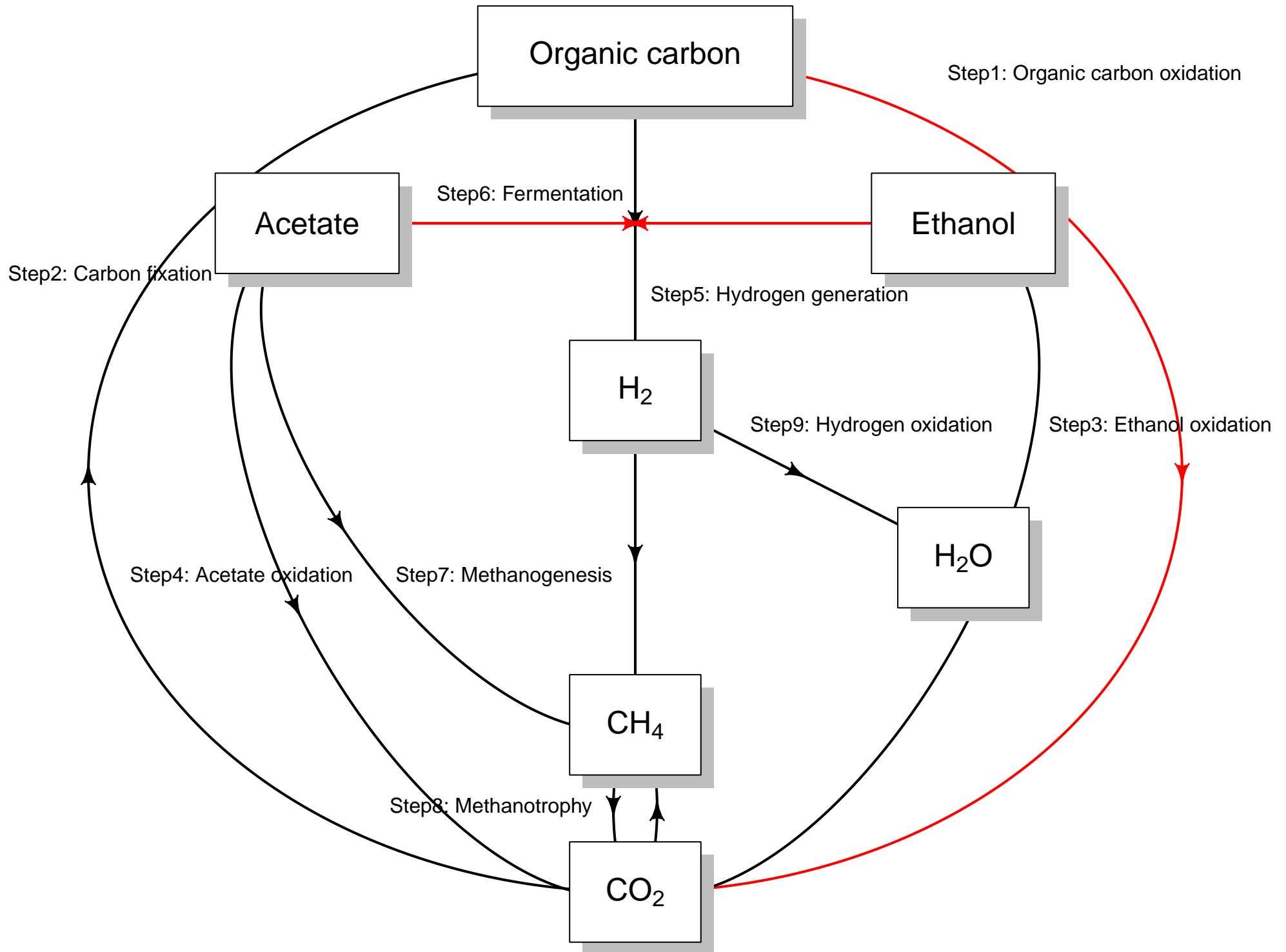

Supplement: Supplementary file 2 — Recovered nutrient cycles including Carbon, Nitrogen, and Sulphur cycles for all the metagenomic assembled genomes given as PDF images, and identifiable through bin numbers. [file 13104_2025_7441_MOESM2_ESM.zip › Supplementary Data2/bin.19.draw_carbon_cycle_single.pdf]

## Nitrogen Cycle: bin.28

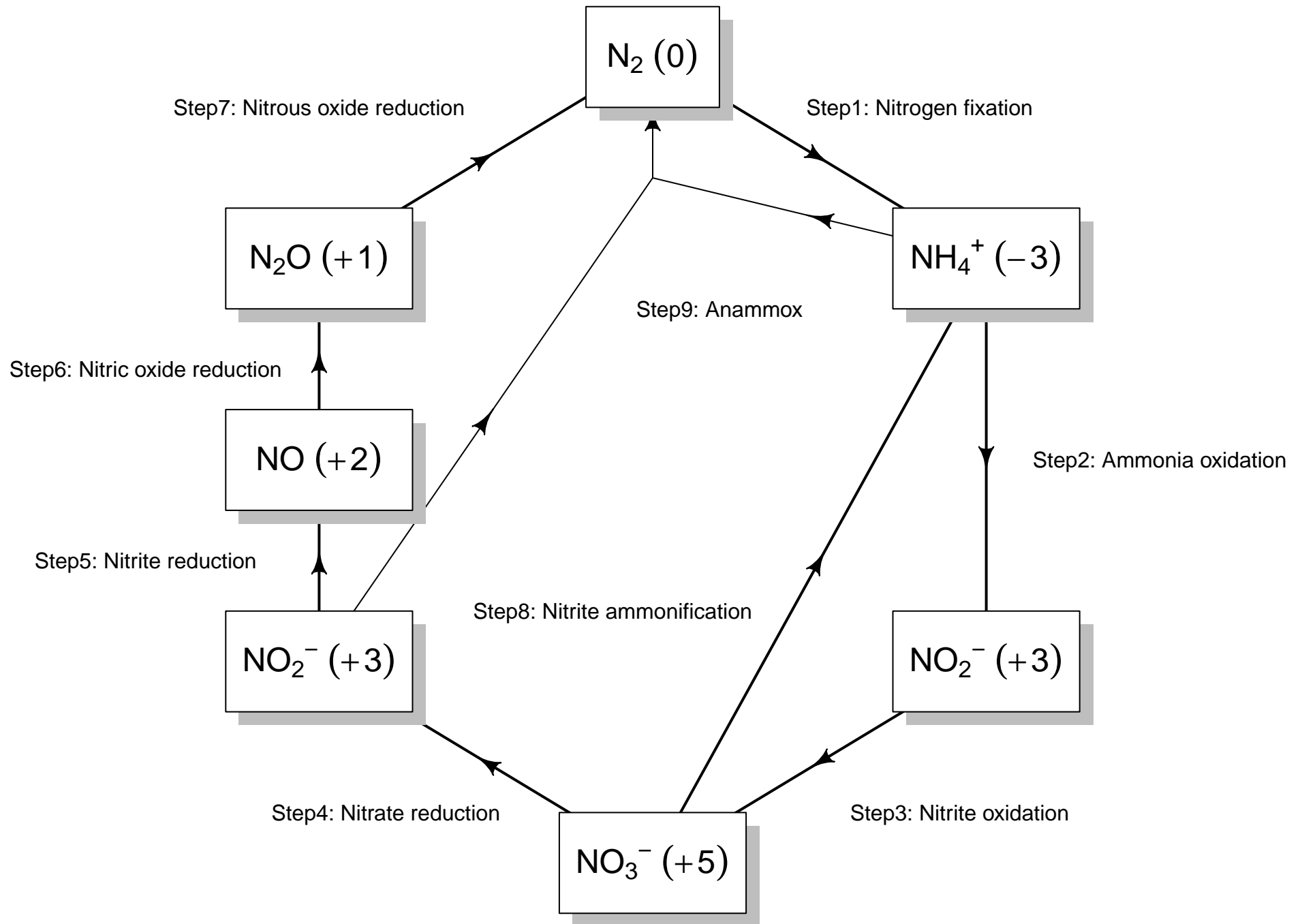

Supplement: Supplementary file 2 — Recovered nutrient cycles including Carbon, Nitrogen, and Sulphur cycles for all the metagenomic assembled genomes given as PDF images, and identifiable through bin numbers. [file 13104_2025_7441_MOESM2_ESM.zip › Supplementary Data2/bin.28.draw_nitrogen_cycle_single.pdf]

## Nitrogen Cycle: bin.123

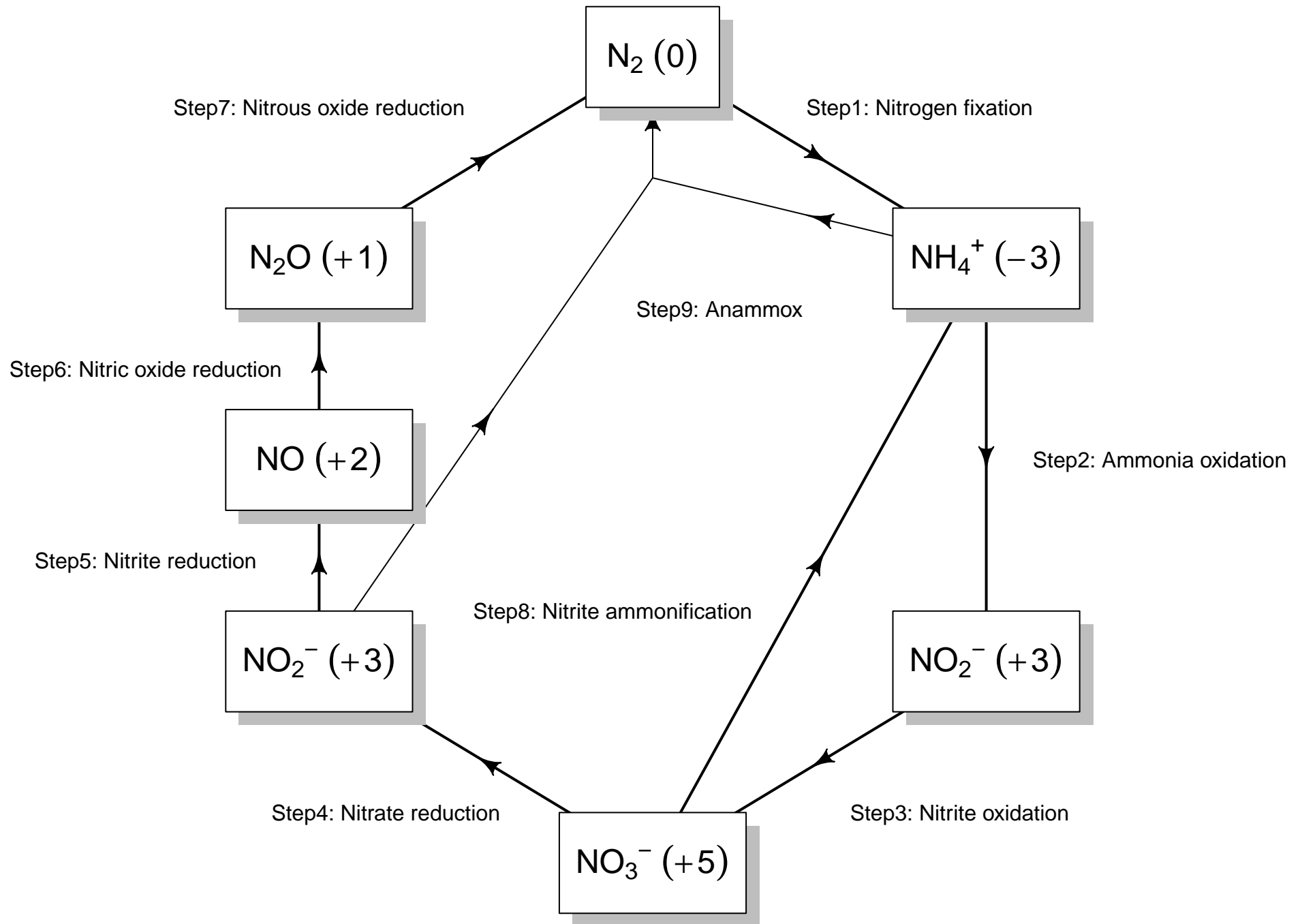

Supplement: Supplementary file 2 — Recovered nutrient cycles including Carbon, Nitrogen, and Sulphur cycles for all the metagenomic assembled genomes given as PDF images, and identifiable through bin numbers. [file 13104_2025_7441_MOESM2_ESM.zip › Supplementary Data2/bin.123.draw_nitrogen_cycle_single.pdf]

# Carbon Cycle: bin.94

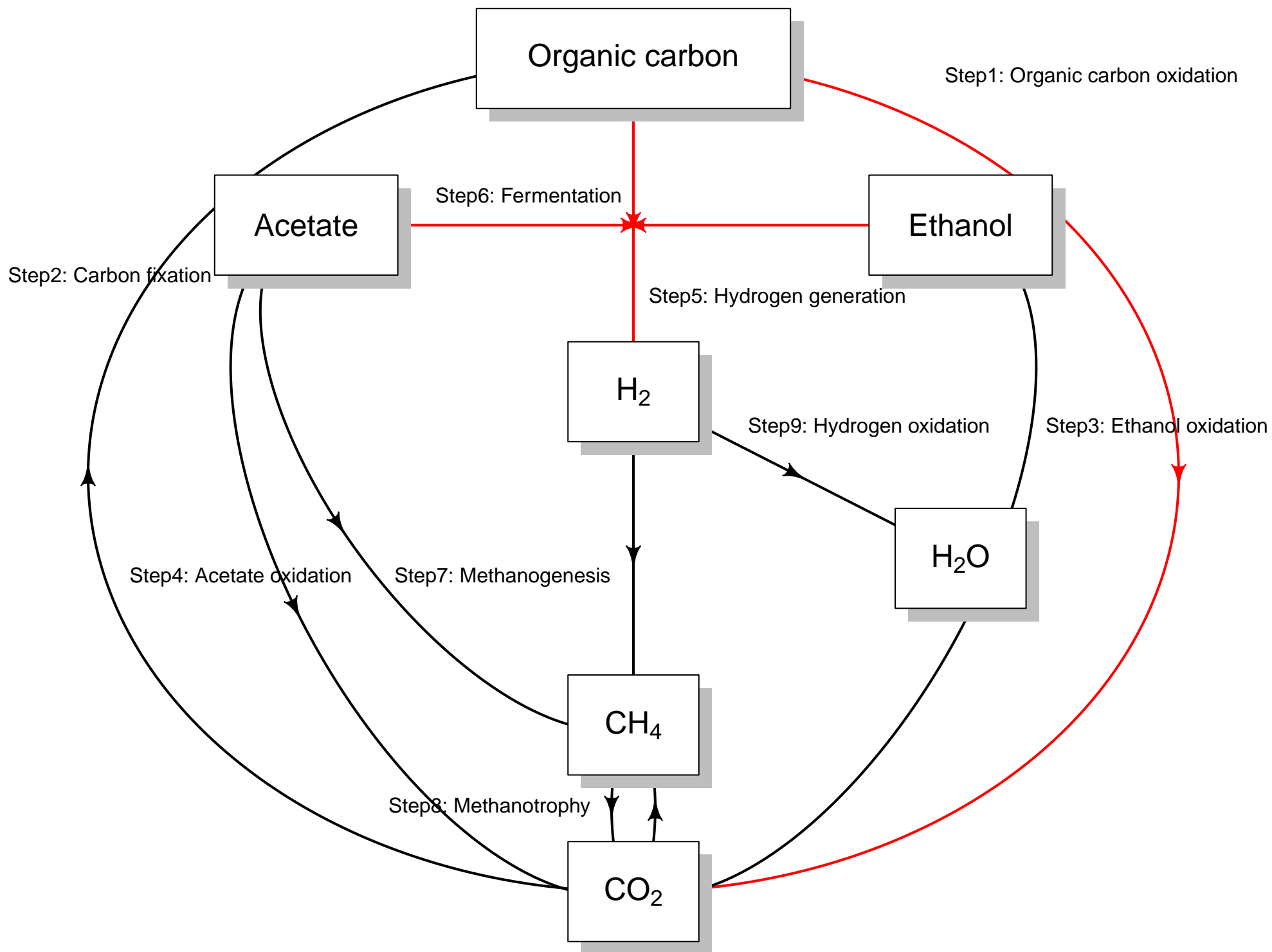

Supplement: Supplementary file 2 — Recovered nutrient cycles including Carbon, Nitrogen, and Sulphur cycles for all the metagenomic assembled genomes given as PDF images, and identifiable through bin numbers. [file 13104_2025_7441_MOESM2_ESM.zip › Supplementary Data2/bin.94.draw_carbon_cycle_single.pdf]

## Nitrogen Cycle: bin.4

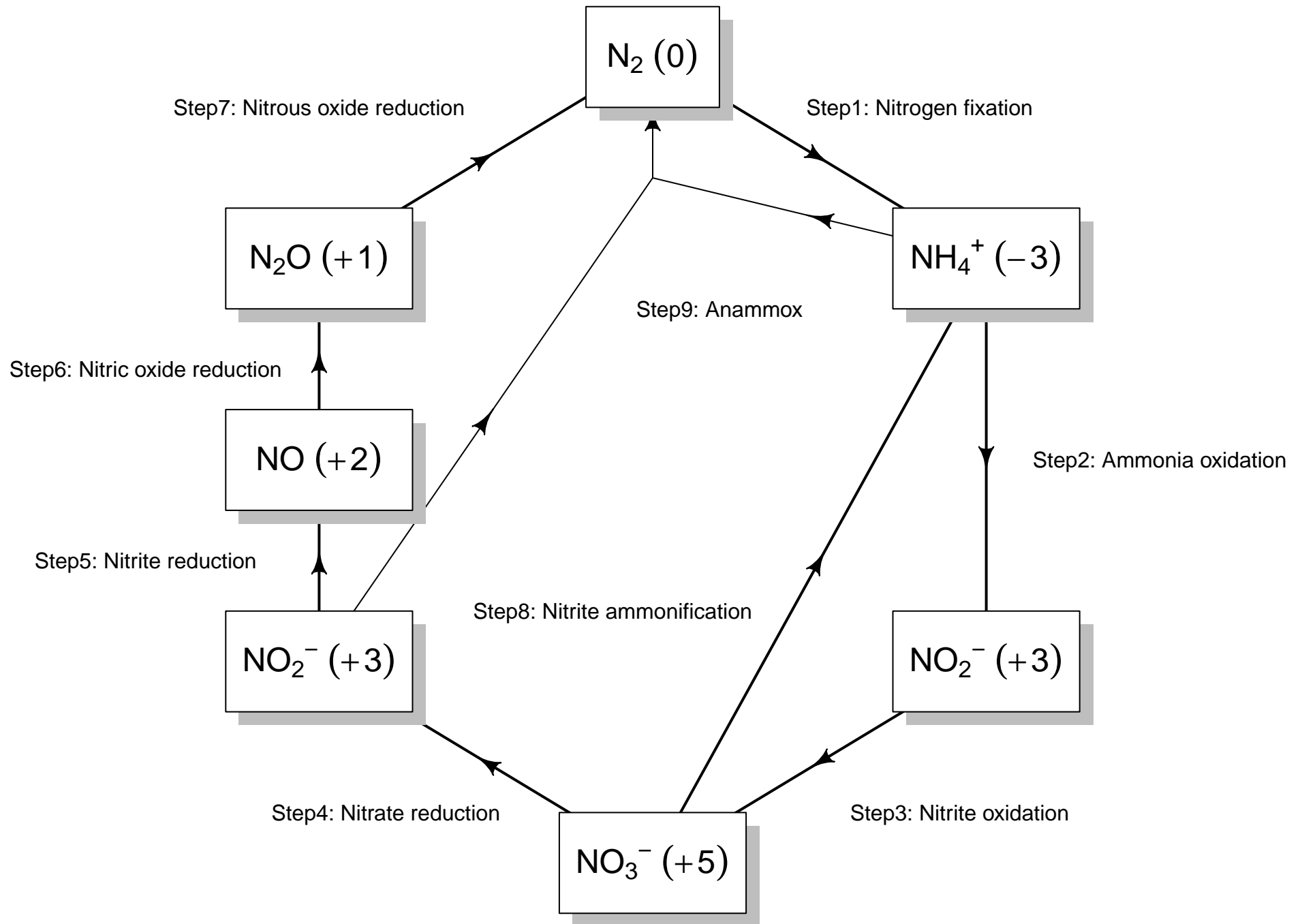

Supplement: Supplementary file 2 — Recovered nutrient cycles including Carbon, Nitrogen, and Sulphur cycles for all the metagenomic assembled genomes given as PDF images, and identifiable through bin numbers. [file 13104_2025_7441_MOESM2_ESM.zip › Supplementary Data2/bin.4.draw_nitrogen_cycle_single.pdf]

## Nitrogen Cycle: bin.67

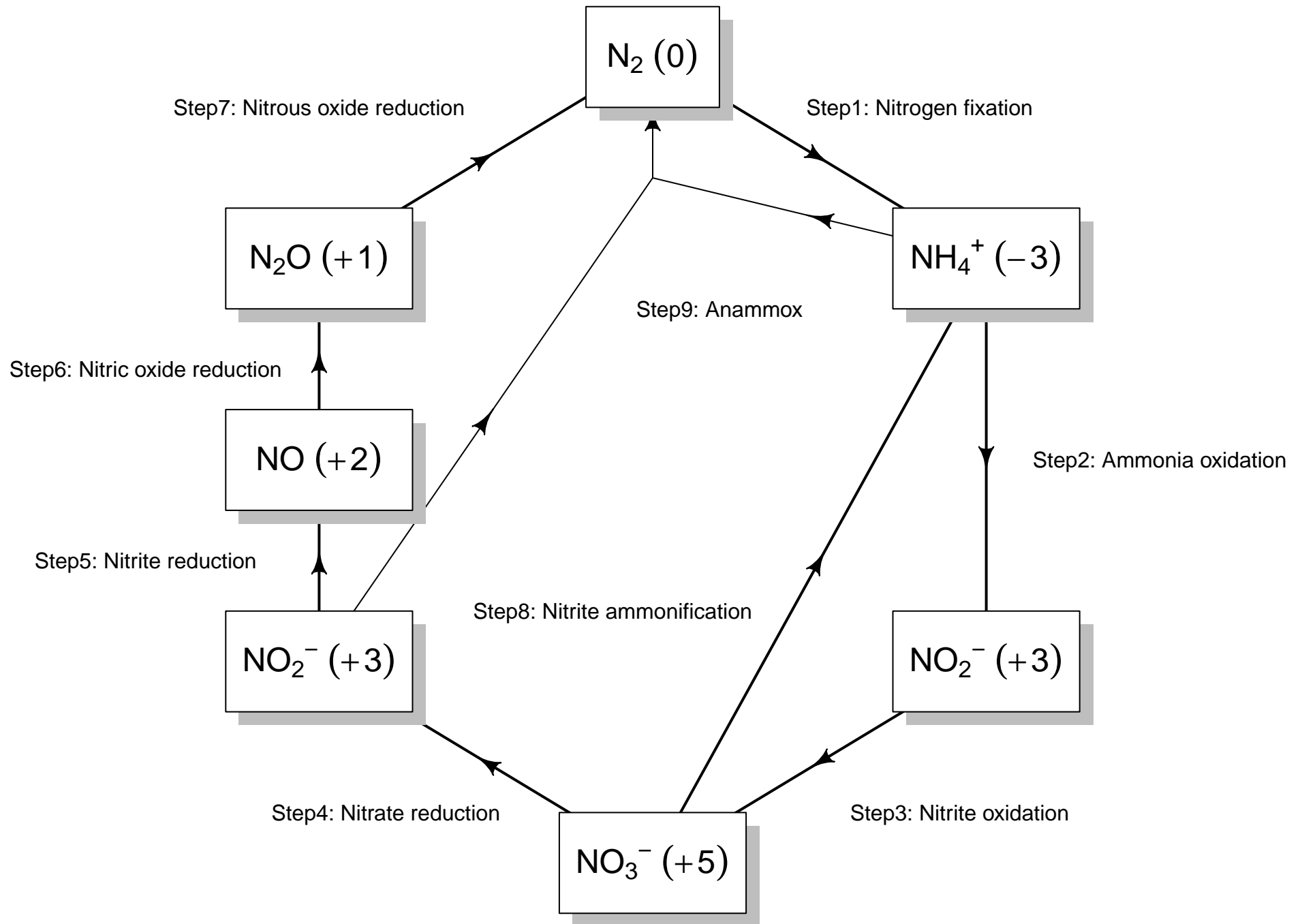

Supplement: Supplementary file 2 — Recovered nutrient cycles including Carbon, Nitrogen, and Sulphur cycles for all the metagenomic assembled genomes given as PDF images, and identifiable through bin numbers. [file 13104_2025_7441_MOESM2_ESM.zip › Supplementary Data2/bin.67.draw_nitrogen_cycle_single.pdf]

## Sulfur Cycle: bin.112

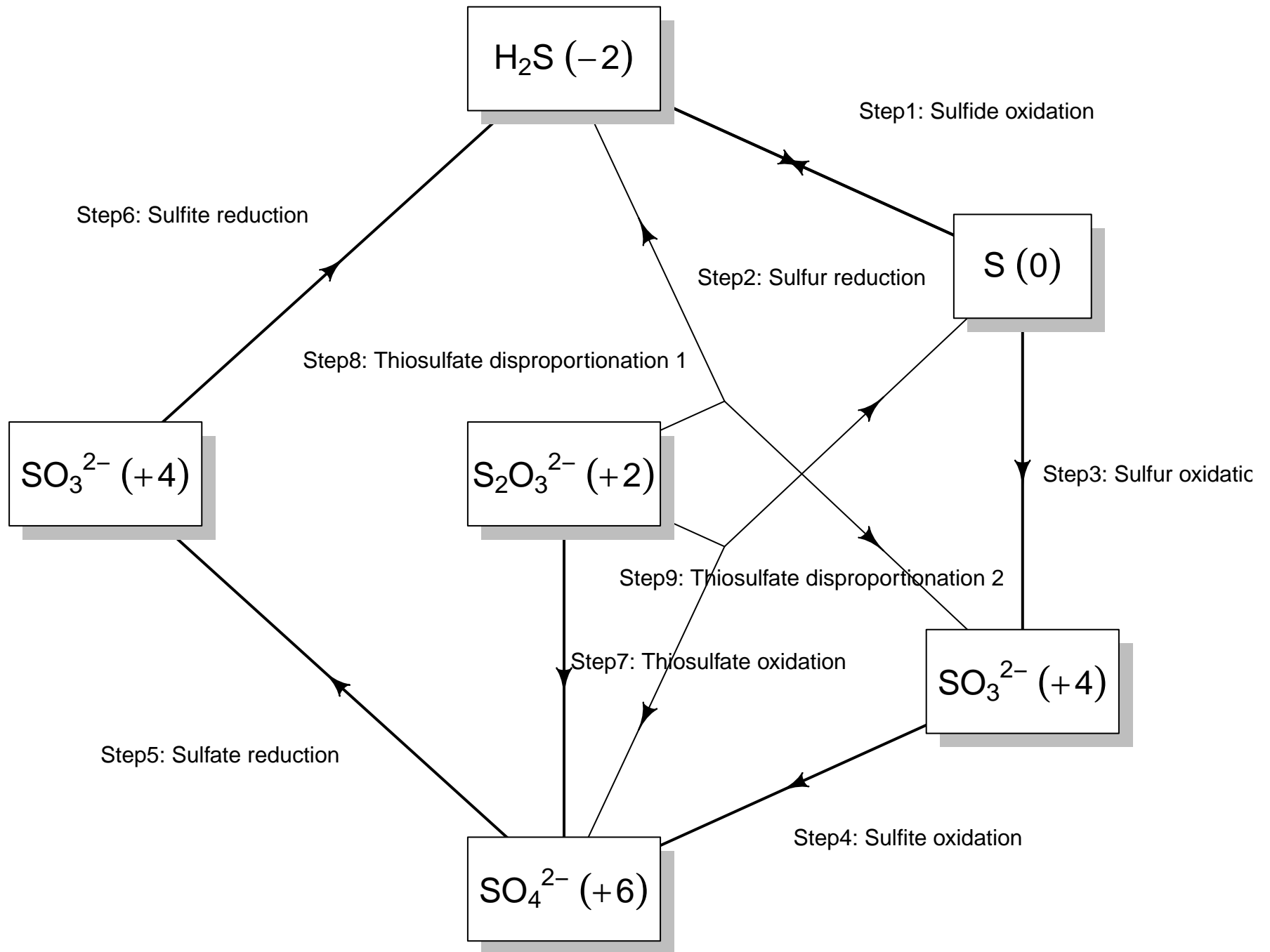

Supplement: Supplementary file 2 — Recovered nutrient cycles including Carbon, Nitrogen, and Sulphur cycles for all the metagenomic assembled genomes given as PDF images, and identifiable through bin numbers. [file 13104_2025_7441_MOESM2_ESM.zip › Supplementary Data2/bin.112.draw_sulfur_cycle_single.pdf]

# Nitrogen Cycle: bin.74

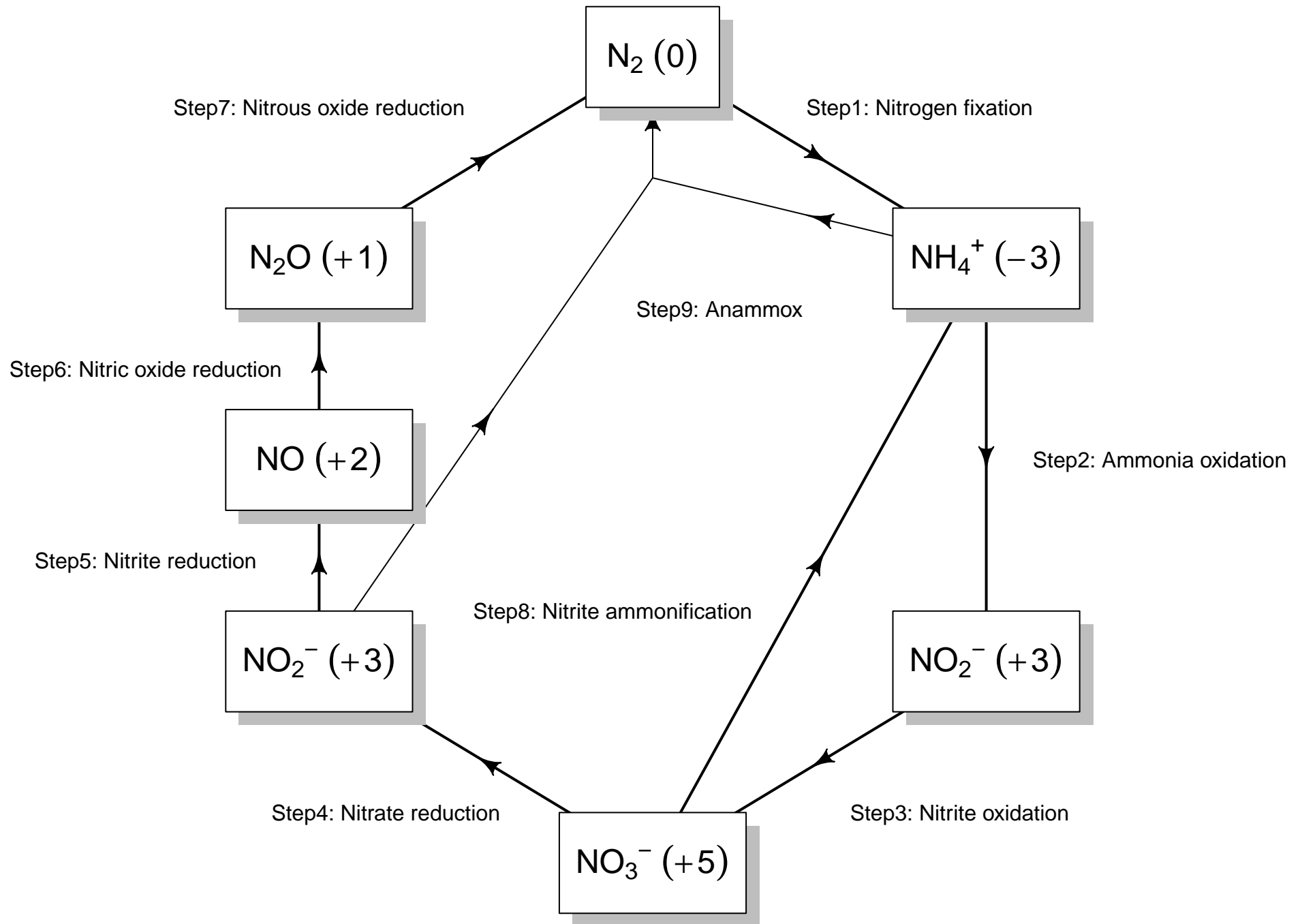

Supplement: Supplementary file 2 — Recovered nutrient cycles including Carbon, Nitrogen, and Sulphur cycles for all the metagenomic assembled genomes given as PDF images, and identifiable through bin numbers. [file 13104_2025_7441_MOESM2_ESM.zip › Supplementary Data2/bin.74.draw_nitrogen_cycle_single.pdf]

# Carbon Cycle: bin.131

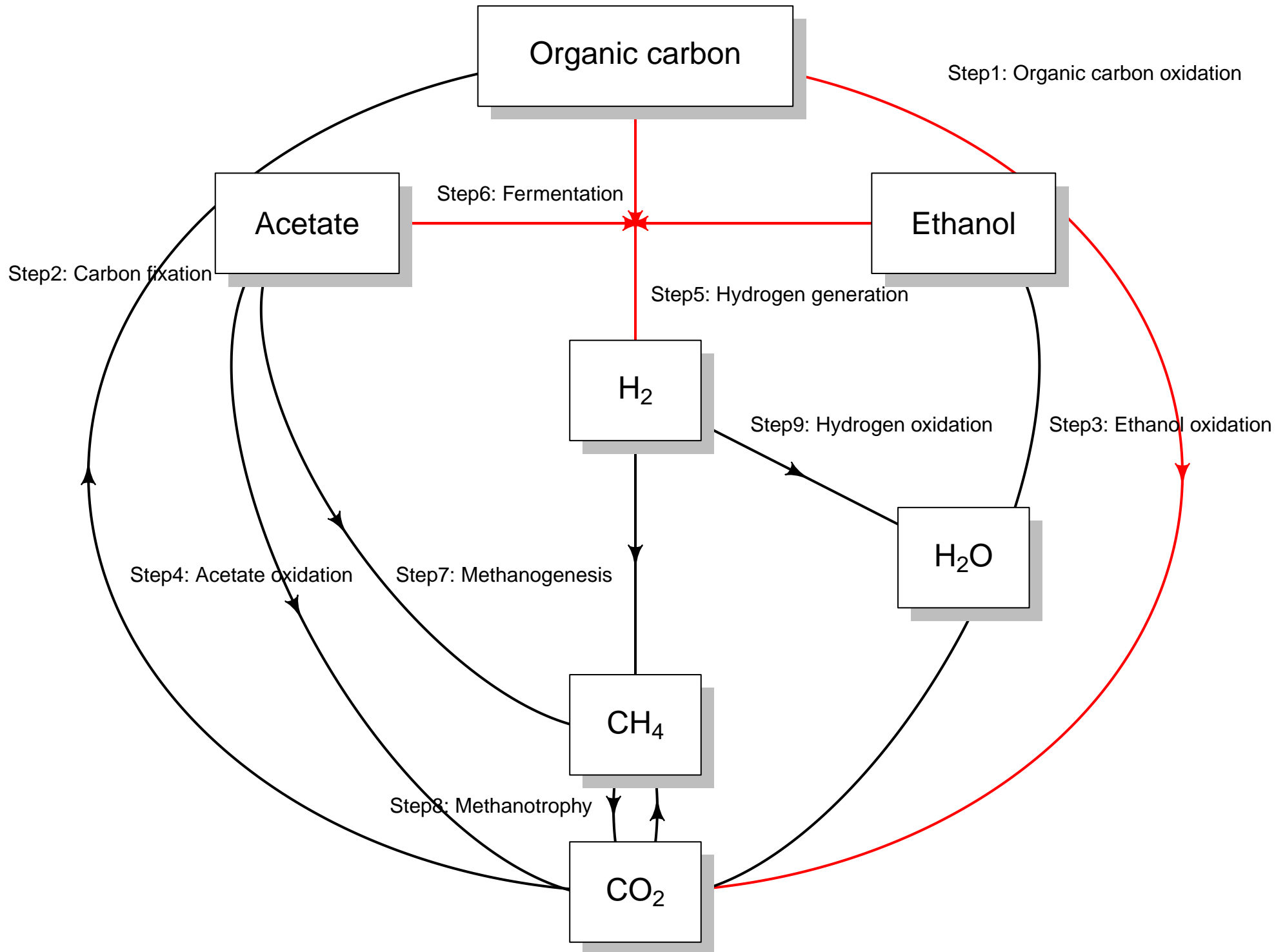

Supplement: Supplementary file 2 — Recovered nutrient cycles including Carbon, Nitrogen, and Sulphur cycles for all the metagenomic assembled genomes given as PDF images, and identifiable through bin numbers. [file 13104_2025_7441_MOESM2_ESM.zip › Supplementary Data2/bin.131.draw_carbon_cycle_single.pdf]

## Carbon Cycle: bin.2

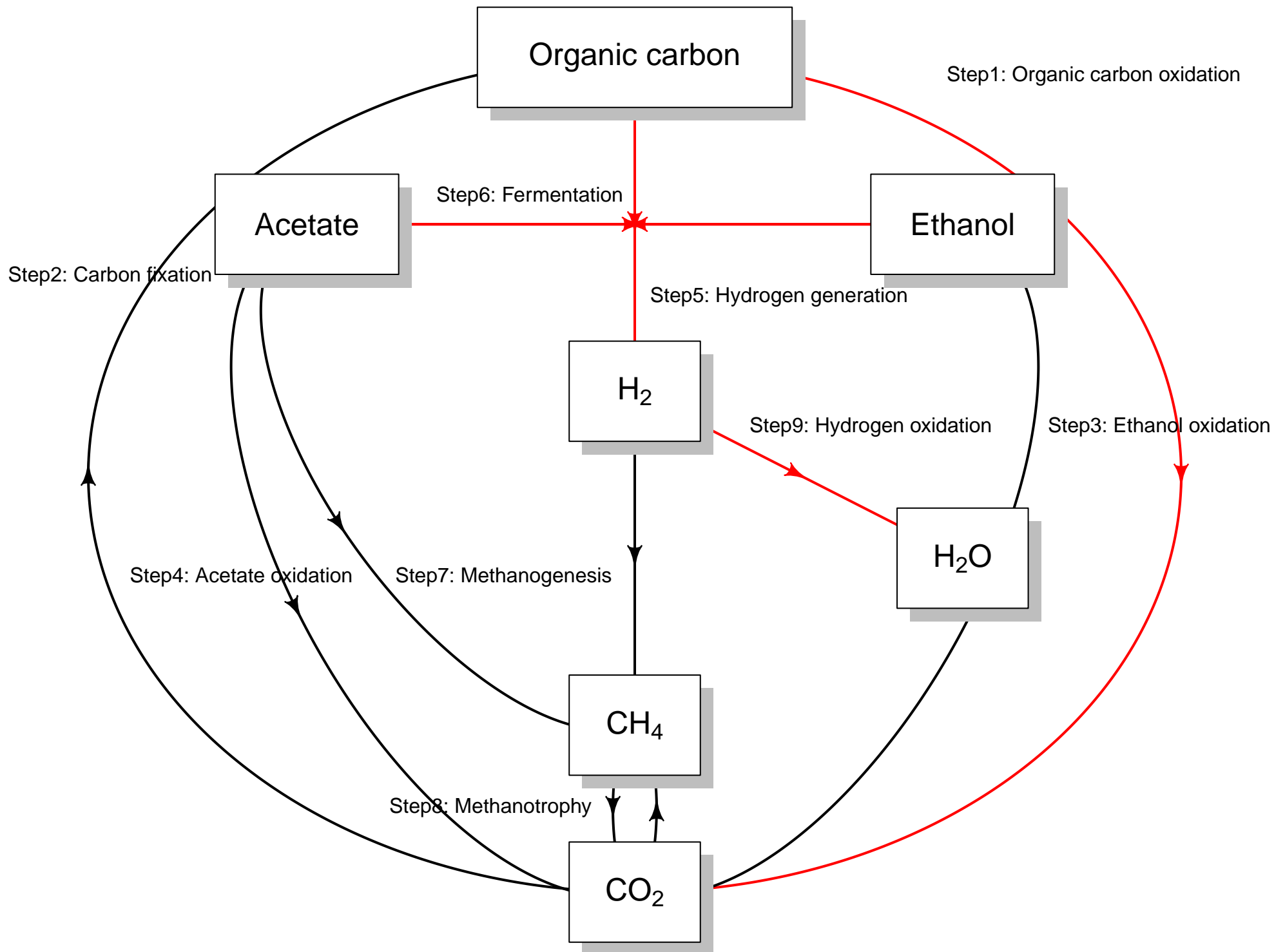

Supplement: Supplementary file 2 — Recovered nutrient cycles including Carbon, Nitrogen, and Sulphur cycles for all the metagenomic assembled genomes given as PDF images, and identifiable through bin numbers. [file 13104_2025_7441_MOESM2_ESM.zip › Supplementary Data2/bin.2.draw_carbon_cycle_single.pdf]

## Nitrogen Cycle: bin.130

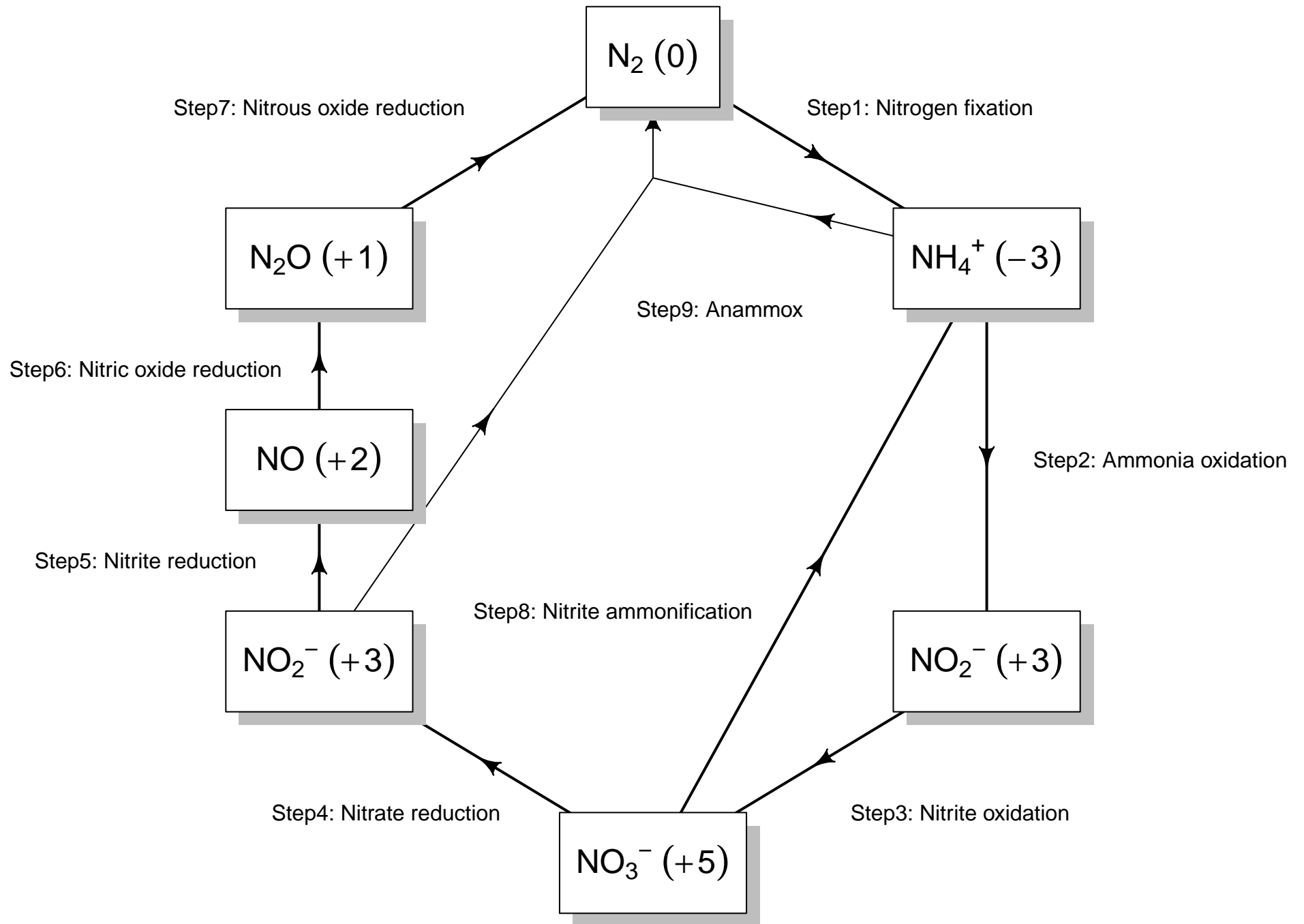

Supplement: Supplementary file 2 — Recovered nutrient cycles including Carbon, Nitrogen, and Sulphur cycles for all the metagenomic assembled genomes given as PDF images, and identifiable through bin numbers. [file 13104_2025_7441_MOESM2_ESM.zip › Supplementary Data2/bin.130.draw_nitrogen_cycle_single.pdf]

## Sulfur Cycle: bin.21

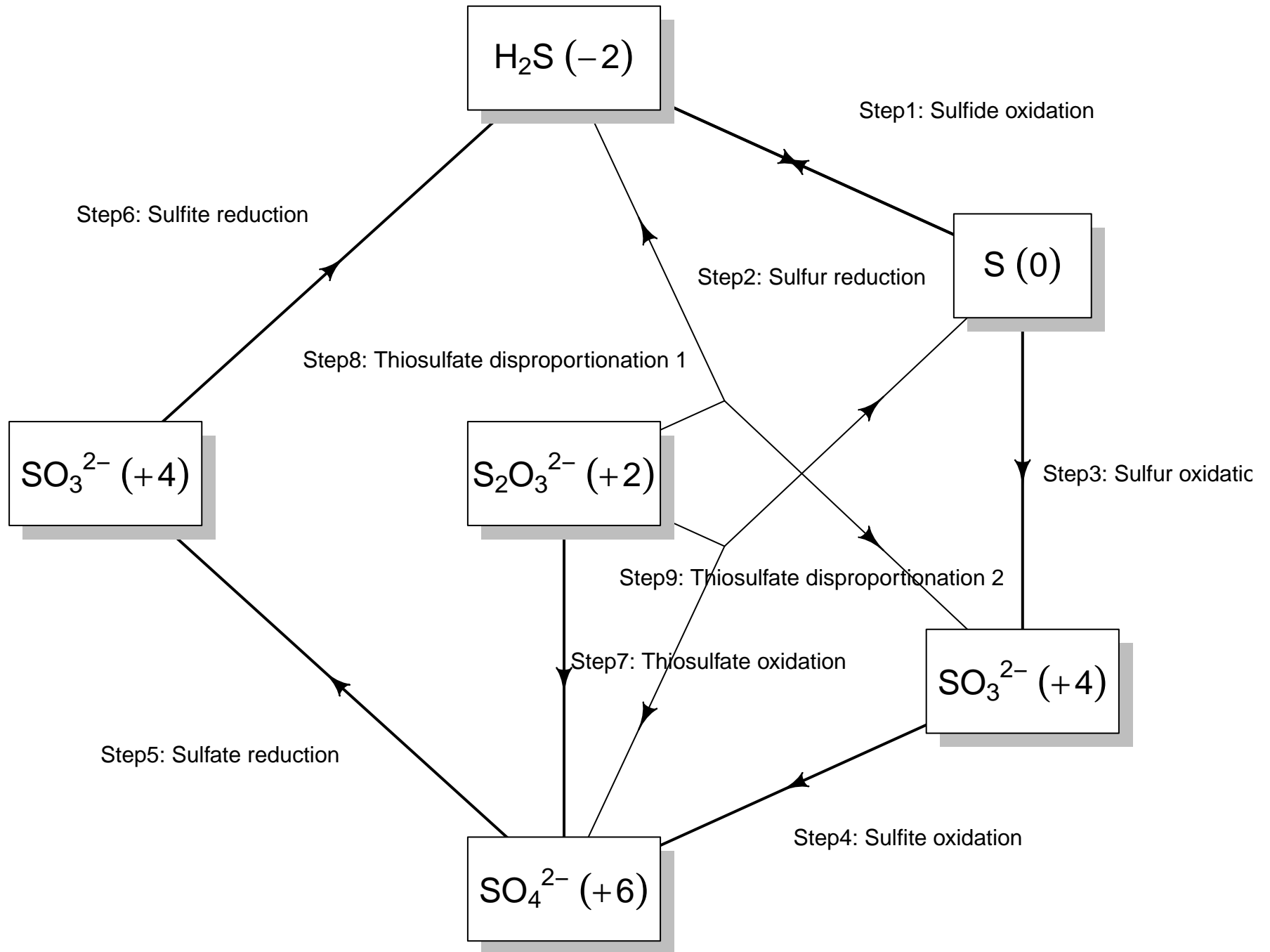

Supplement: Supplementary file 2 — Recovered nutrient cycles including Carbon, Nitrogen, and Sulphur cycles for all the metagenomic assembled genomes given as PDF images, and identifiable through bin numbers. [file 13104_2025_7441_MOESM2_ESM.zip › Supplementary Data2/bin.21.draw_sulfur_cycle_single.pdf]

## Nitrogen Cycle: bin.129

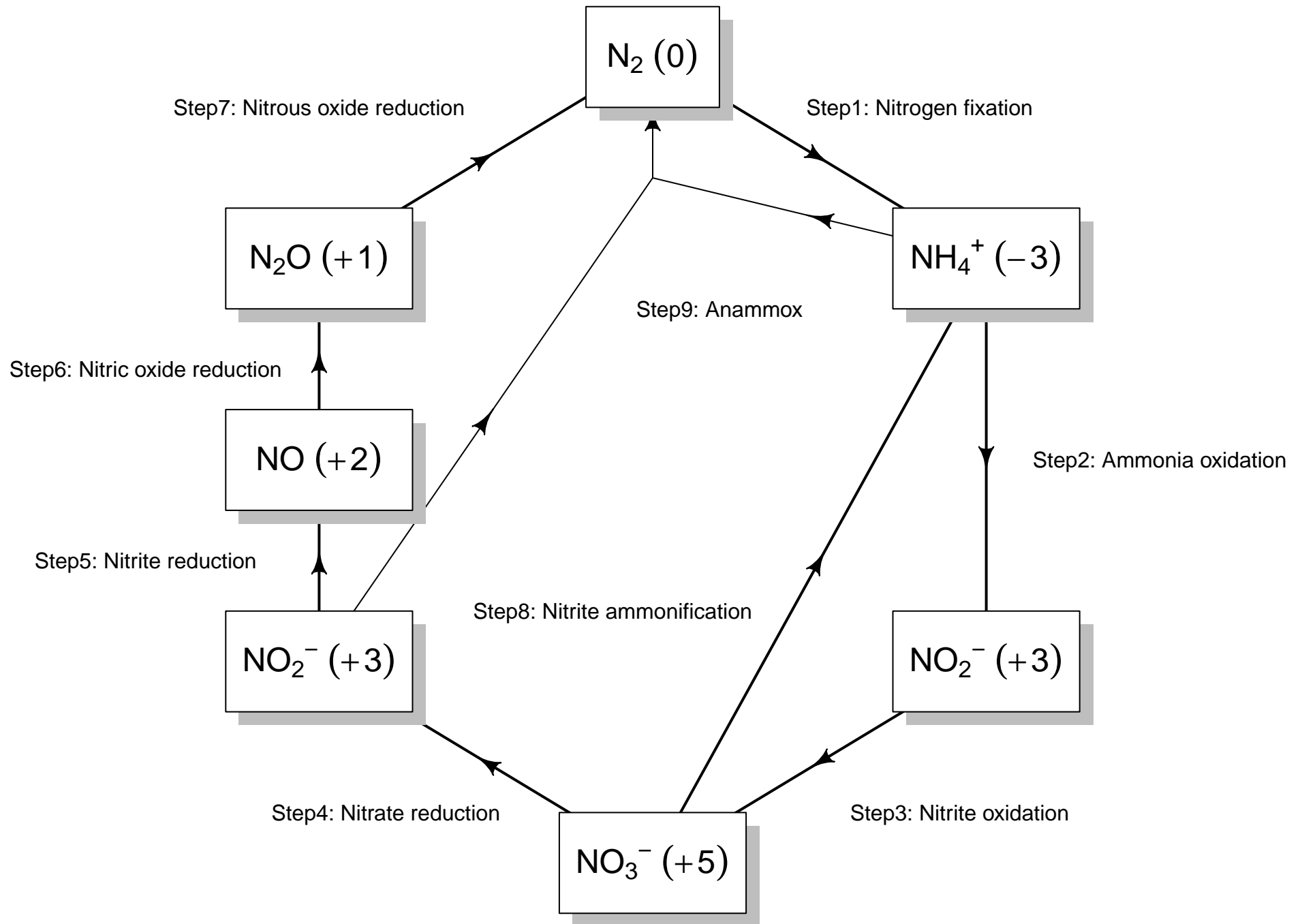

Supplement: Supplementary file 2 — Recovered nutrient cycles including Carbon, Nitrogen, and Sulphur cycles for all the metagenomic assembled genomes given as PDF images, and identifiable through bin numbers. [file 13104_2025_7441_MOESM2_ESM.zip › Supplementary Data2/bin.129.draw_nitrogen_cycle_single.pdf]

# Carbon Cycle: bin.55

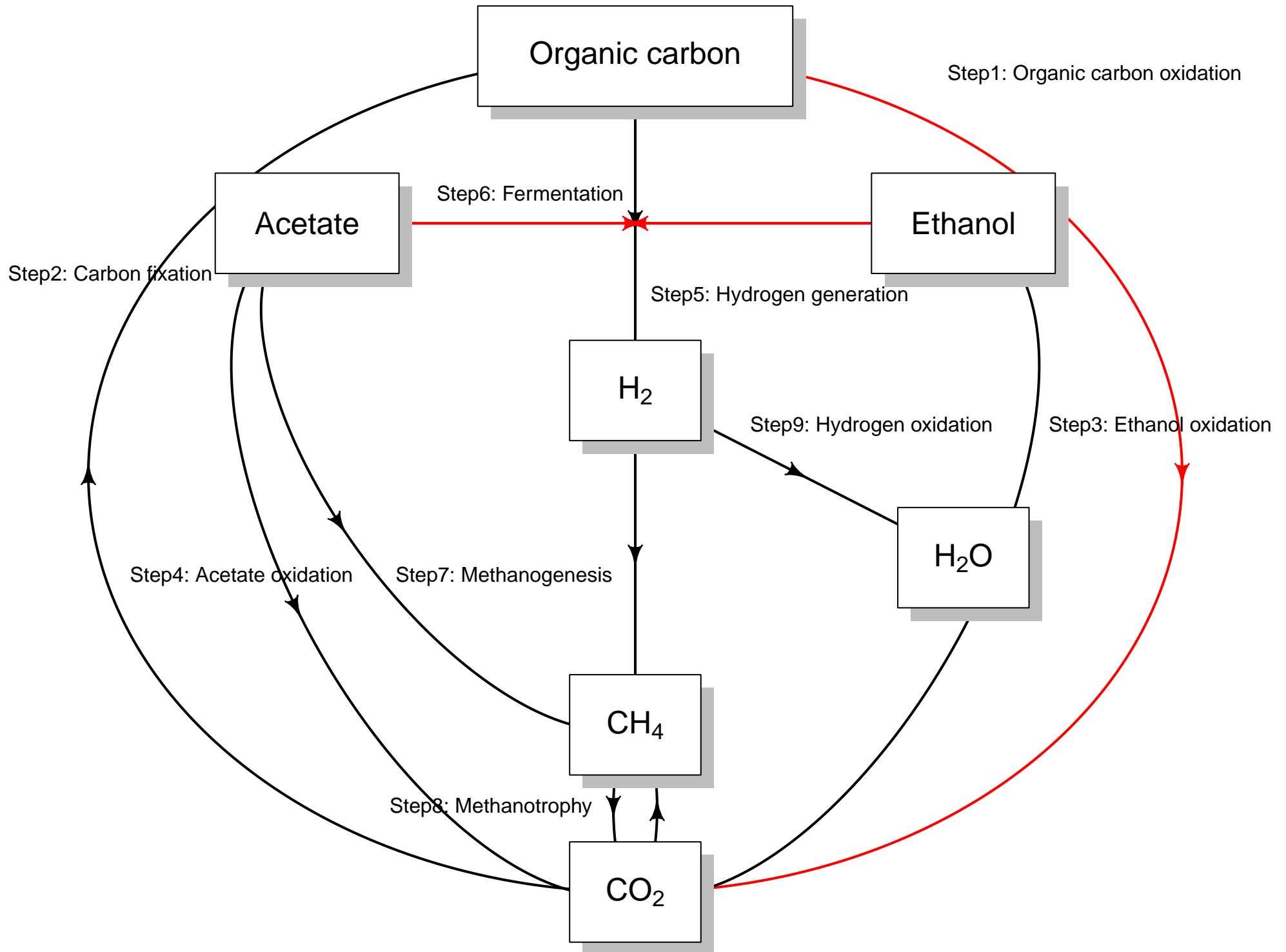

Supplement: Supplementary file 2 — Recovered nutrient cycles including Carbon, Nitrogen, and Sulphur cycles for all the metagenomic assembled genomes given as PDF images, and identifiable through bin numbers. [file 13104_2025_7441_MOESM2_ESM.zip › Supplementary Data2/bin.55.draw_carbon_cycle_single.pdf]

## Nitrogen Cycle: bin.22

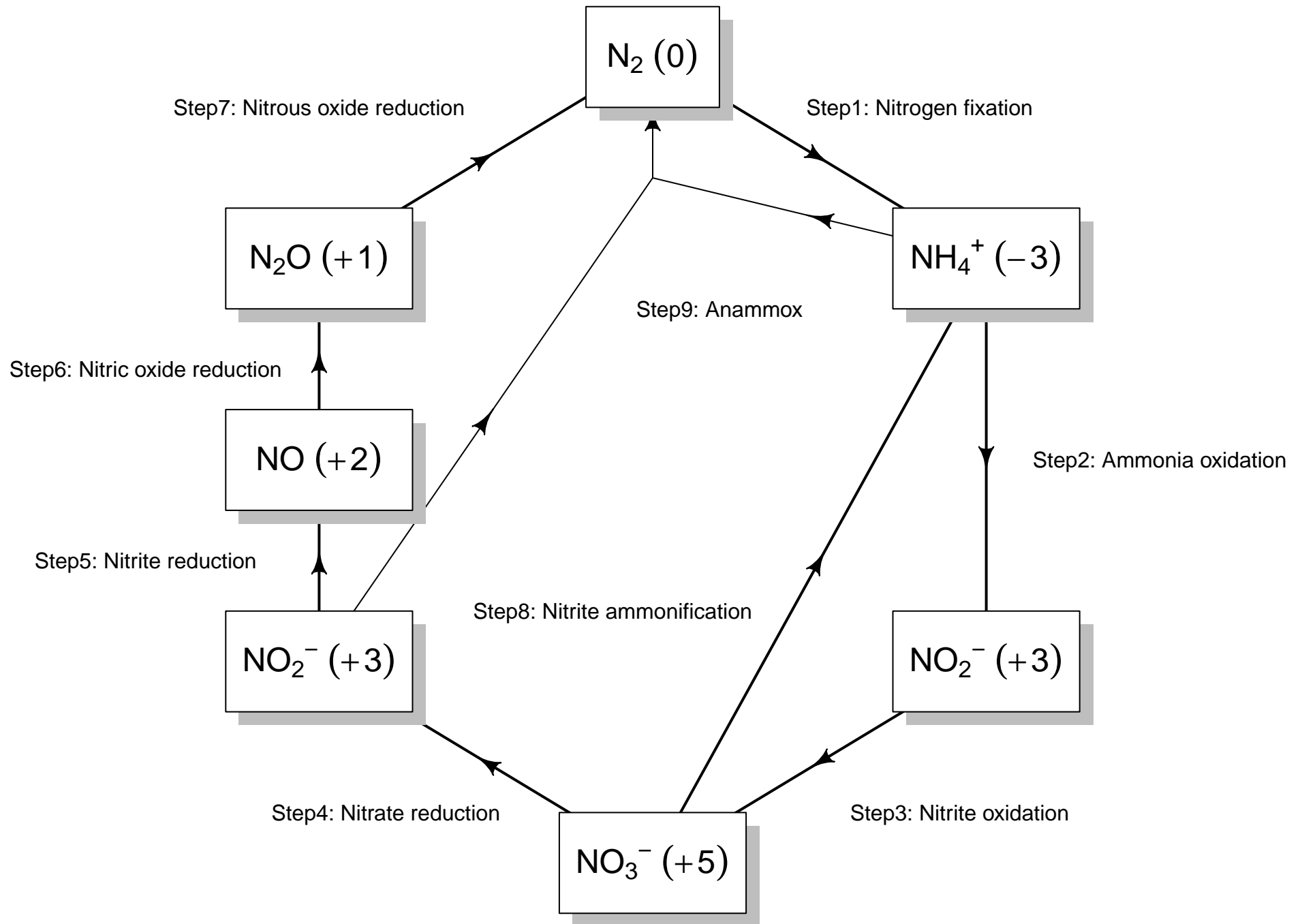

Supplement: Supplementary file 2 — Recovered nutrient cycles including Carbon, Nitrogen, and Sulphur cycles for all the metagenomic assembled genomes given as PDF images, and identifiable through bin numbers. [file 13104_2025_7441_MOESM2_ESM.zip › Supplementary Data2/bin.22.draw_nitrogen_cycle_single.pdf]

## Sulfur Cycle: bin.145

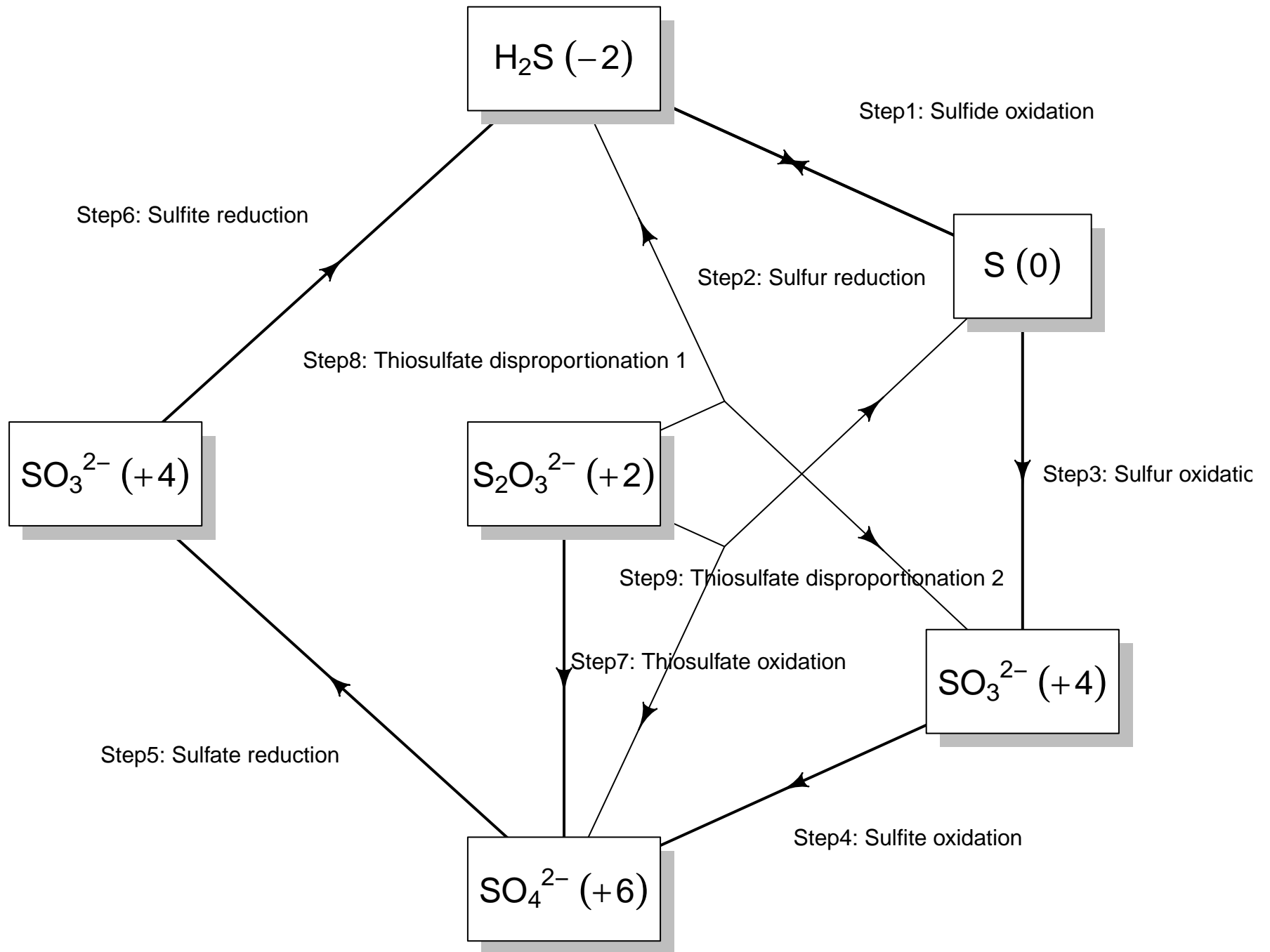

Supplement: Supplementary file 2 — Recovered nutrient cycles including Carbon, Nitrogen, and Sulphur cycles for all the metagenomic assembled genomes given as PDF images, and identifiable through bin numbers. [file 13104_2025_7441_MOESM2_ESM.zip › Supplementary Data2/bin.145.draw_sulfur_cycle_single.pdf]

# Nitrogen Cycle: bin.31

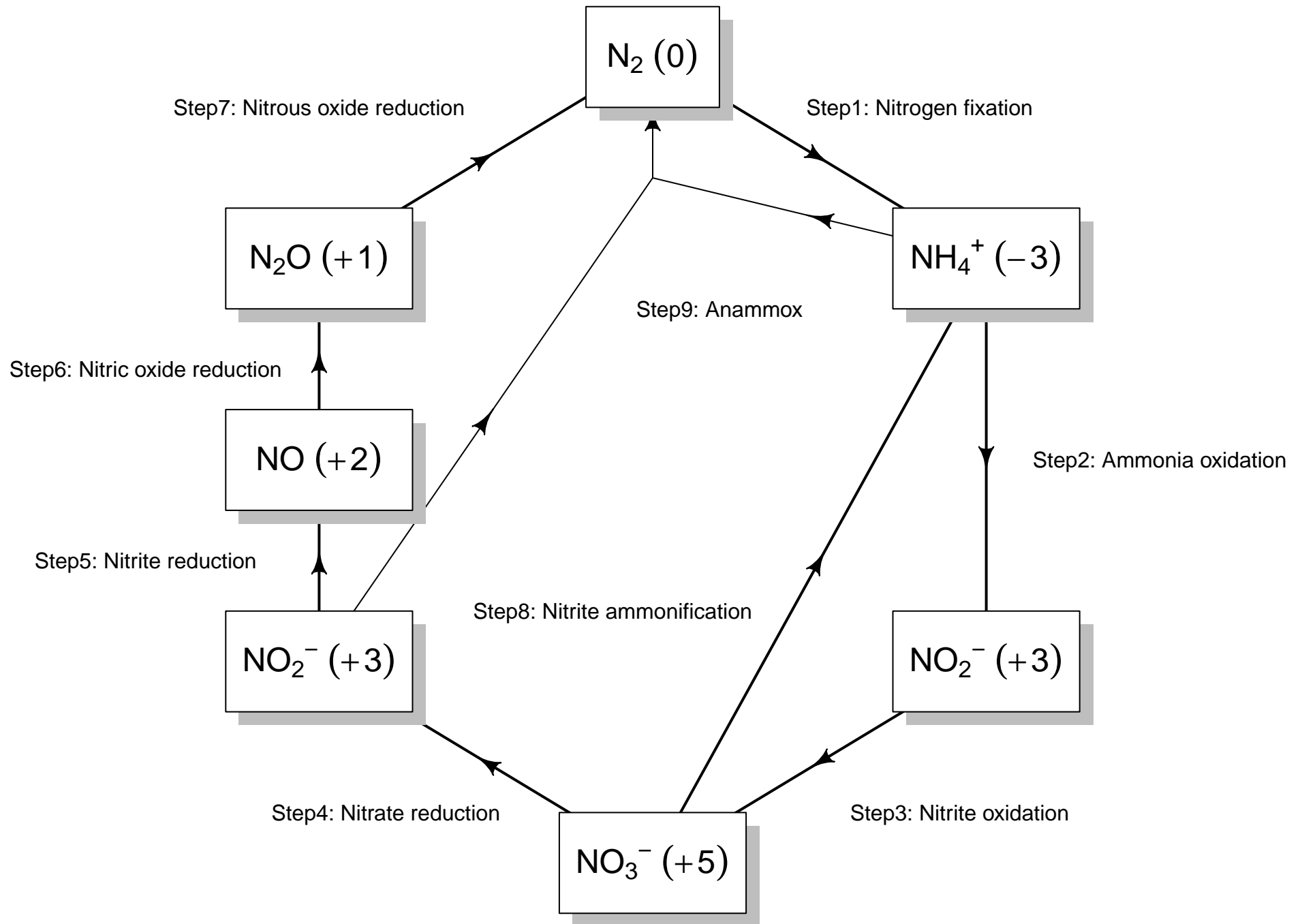

Supplement: Supplementary file 2 — Recovered nutrient cycles including Carbon, Nitrogen, and Sulphur cycles for all the metagenomic assembled genomes given as PDF images, and identifiable through bin numbers. [file 13104_2025_7441_MOESM2_ESM.zip › Supplementary Data2/bin.31.draw_nitrogen_cycle_single.pdf]

## Sulfur Cycle: bin.78

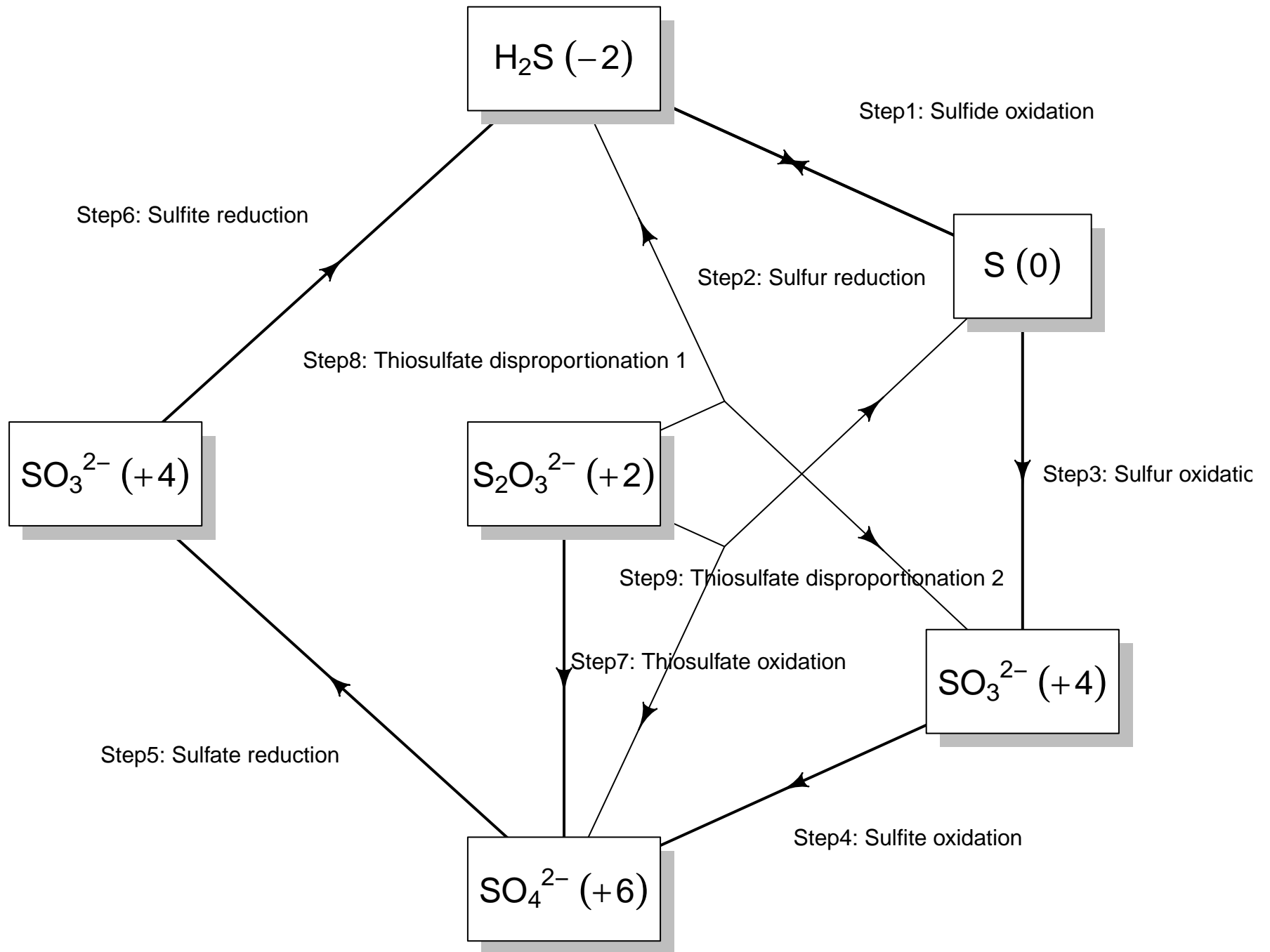

Supplement: Supplementary file 2 — Recovered nutrient cycles including Carbon, Nitrogen, and Sulphur cycles for all the metagenomic assembled genomes given as PDF images, and identifiable through bin numbers. [file 13104_2025_7441_MOESM2_ESM.zip › Supplementary Data2/bin.78.draw_sulfur_cycle_single.pdf]

## Sulfur Cycle: bin.76

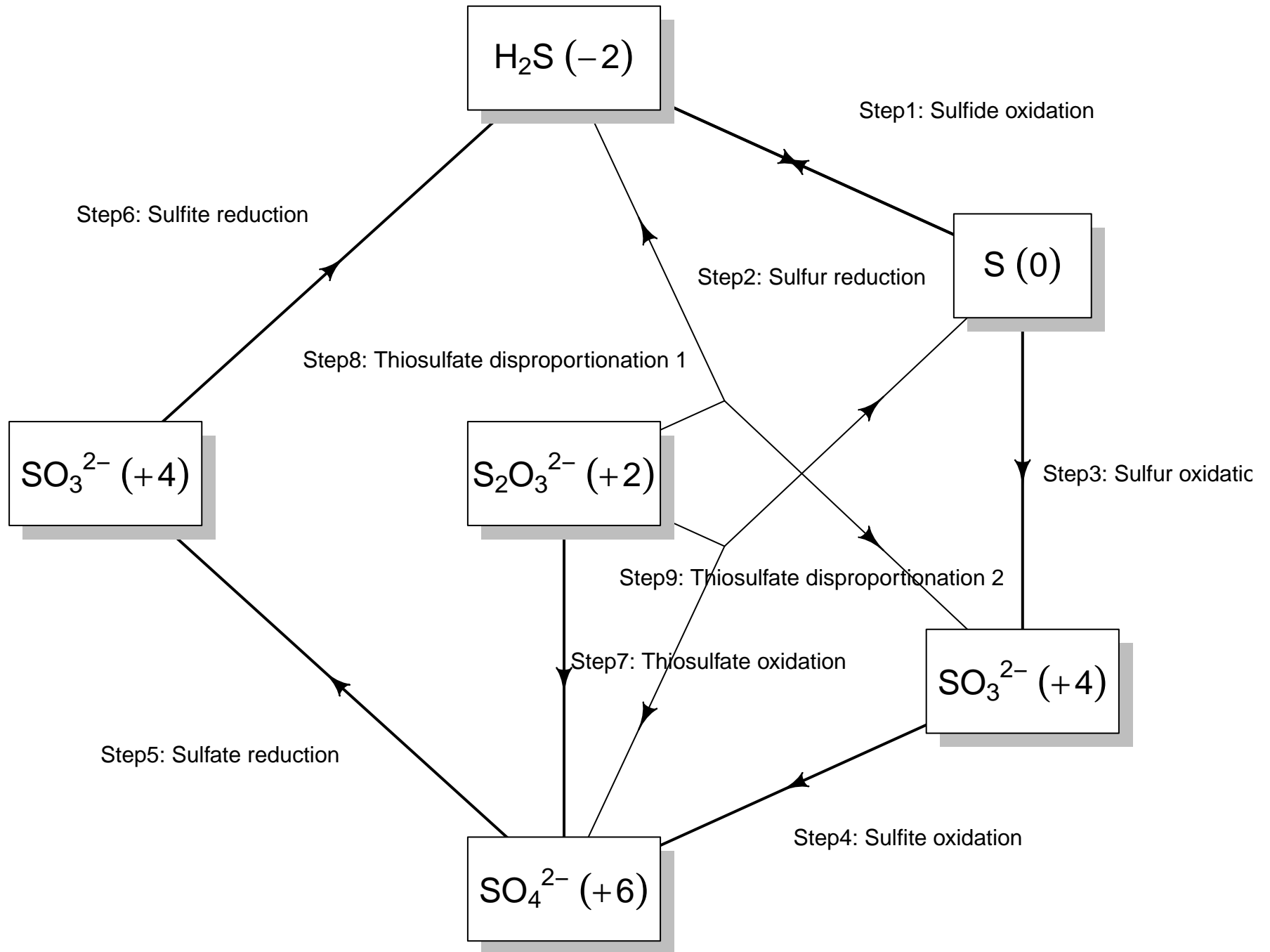

Supplement: Supplementary file 2 — Recovered nutrient cycles including Carbon, Nitrogen, and Sulphur cycles for all the metagenomic assembled genomes given as PDF images, and identifiable through bin numbers. [file 13104_2025_7441_MOESM2_ESM.zip › Supplementary Data2/bin.76.draw_sulfur_cycle_single.pdf]

## Sulfur Cycle: bin.63

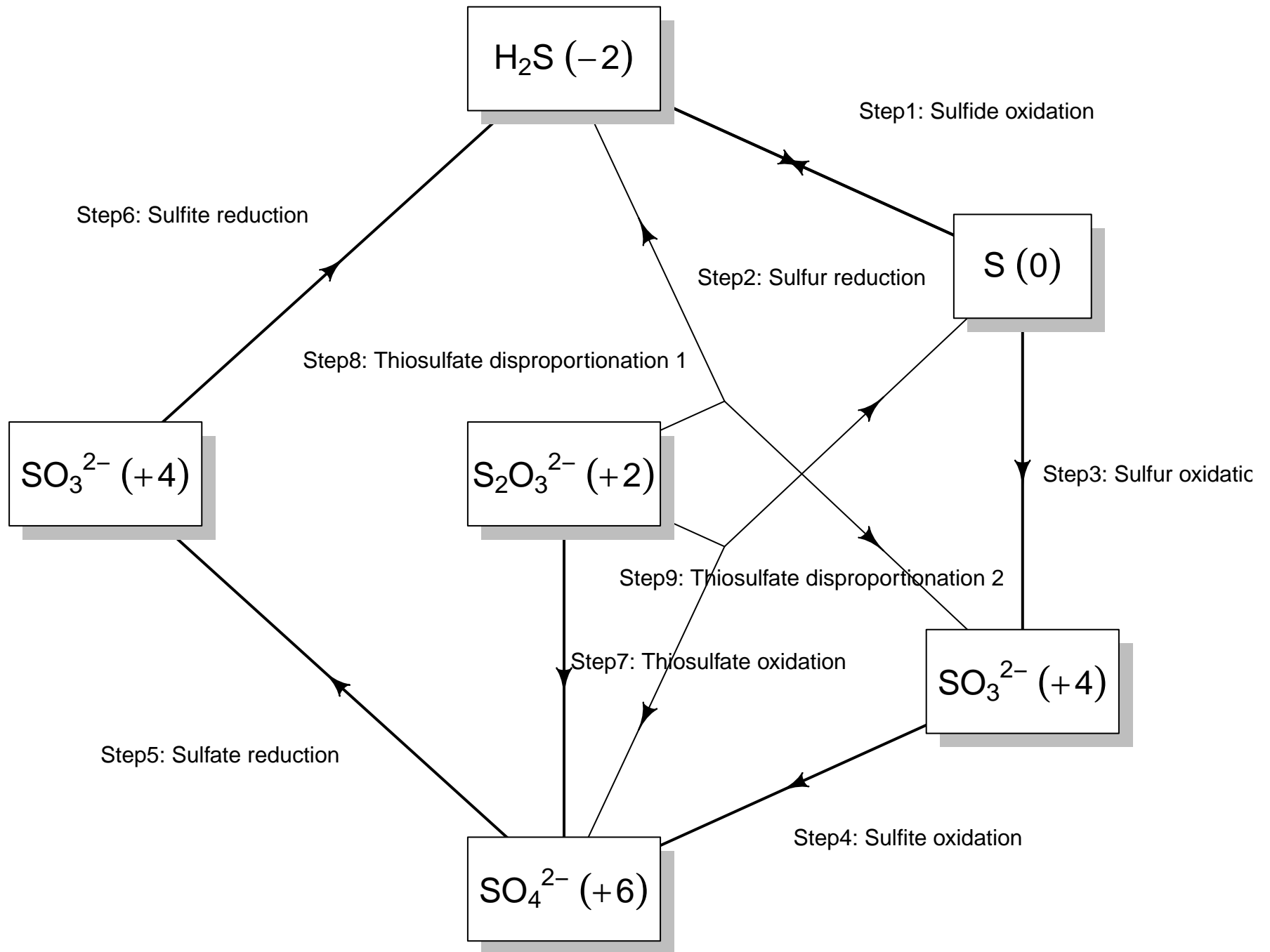

Supplement: Supplementary file 2 — Recovered nutrient cycles including Carbon, Nitrogen, and Sulphur cycles for all the metagenomic assembled genomes given as PDF images, and identifiable through bin numbers. [file 13104_2025_7441_MOESM2_ESM.zip › Supplementary Data2/bin.63.draw_sulfur_cycle_single.pdf]

## Nitrogen Cycle: bin.86

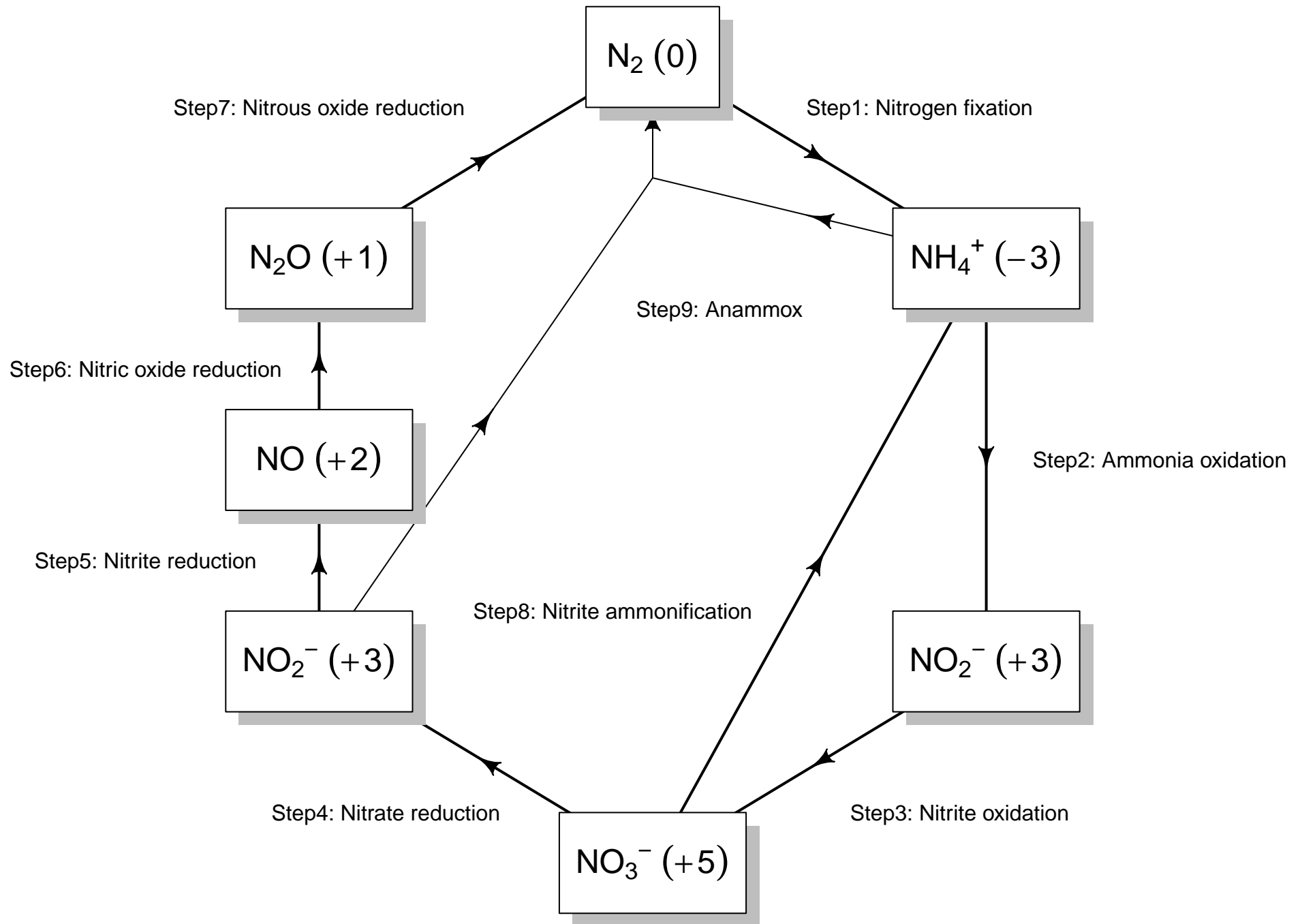

Supplement: Supplementary file 2 — Recovered nutrient cycles including Carbon, Nitrogen, and Sulphur cycles for all the metagenomic assembled genomes given as PDF images, and identifiable through bin numbers. [file 13104_2025_7441_MOESM2_ESM.zip › Supplementary Data2/bin.86.draw_nitrogen_cycle_single.pdf]

## Sulfur Cycle: bin.150

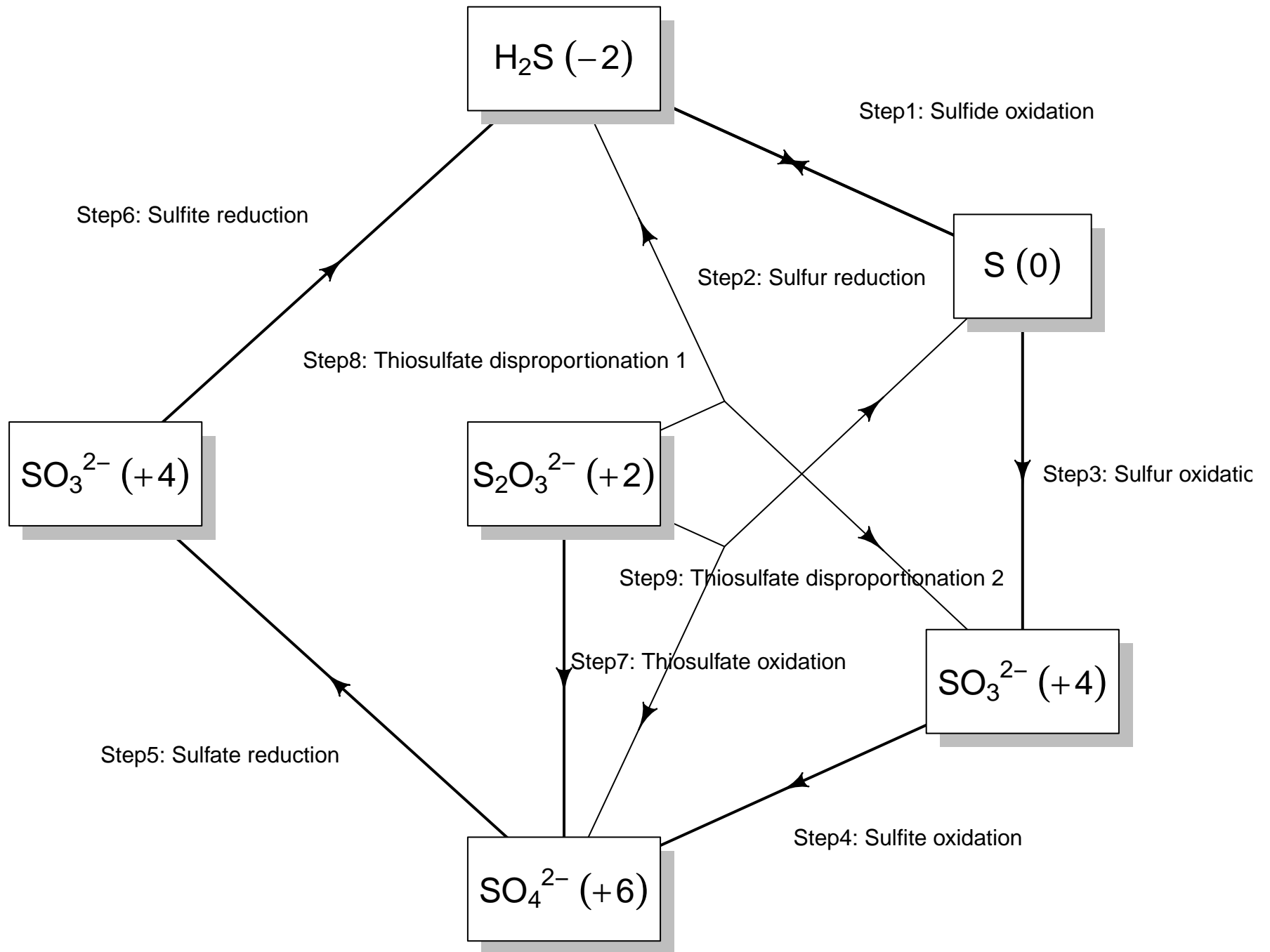

Supplement: Supplementary file 2 — Recovered nutrient cycles including Carbon, Nitrogen, and Sulphur cycles for all the metagenomic assembled genomes given as PDF images, and identifiable through bin numbers. [file 13104_2025_7441_MOESM2_ESM.zip › Supplementary Data2/bin.150.draw_sulfur_cycle_single.pdf]

## Nitrogen Cycle: bin.95

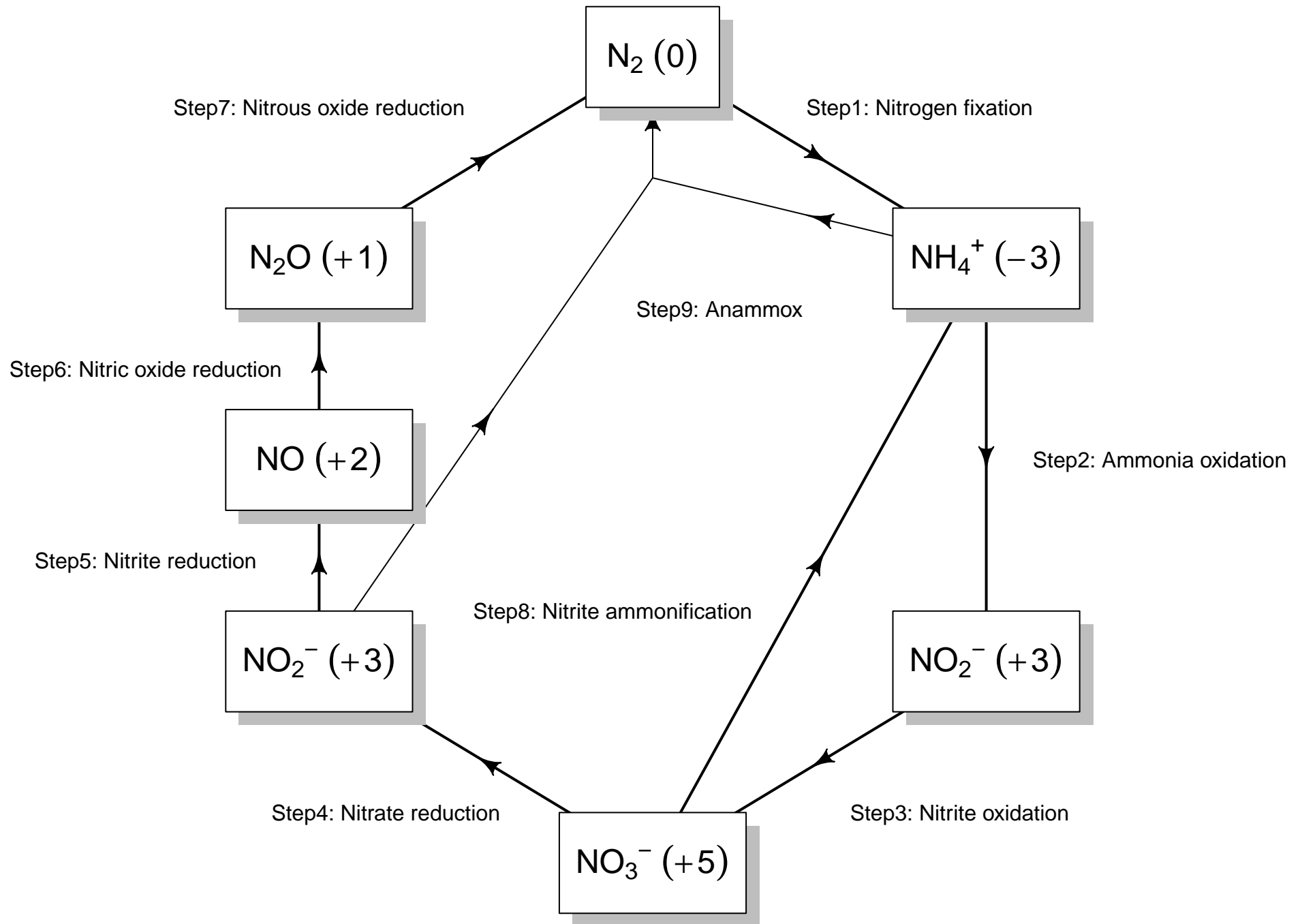

Supplement: Supplementary file 2 — Recovered nutrient cycles including Carbon, Nitrogen, and Sulphur cycles for all the metagenomic assembled genomes given as PDF images, and identifiable through bin numbers. [file 13104_2025_7441_MOESM2_ESM.zip › Supplementary Data2/bin.95.draw_nitrogen_cycle_single.pdf]
