# Supplementary material for: Investigating microbial population structure and function in the chicken caeca and large intestine over time using metagenomics
Source: BMC Res Notes. 2025 Aug 15;18:355. doi: 10.1186/s13104-025-07441-7 (PMC12357378; doi:10.1186/s13104-025-07441-7)
Supplement: Supplementary file 3 — Fig. S1. Geochemical cycles (a-i) recovered from METABOLIC with count showing the number of genomes that have a particular pathway, along with the coverage information for all genomes recovered in this dataset. Associations to parameters (weight gain, age, feed conversion rate, feed intake and body weight) changes are indicated showing the number of genomes with positive association to parameters changes (red), and the number of genomes with negative associations to parameters changes (blue)(e). The arrows (a-c) represent those pathways that were substantially more abundant in the community after the changes. These associations are based on CODA LASSO analysis. [file 13104_2025_7441_MOESM3_ESM.docx]

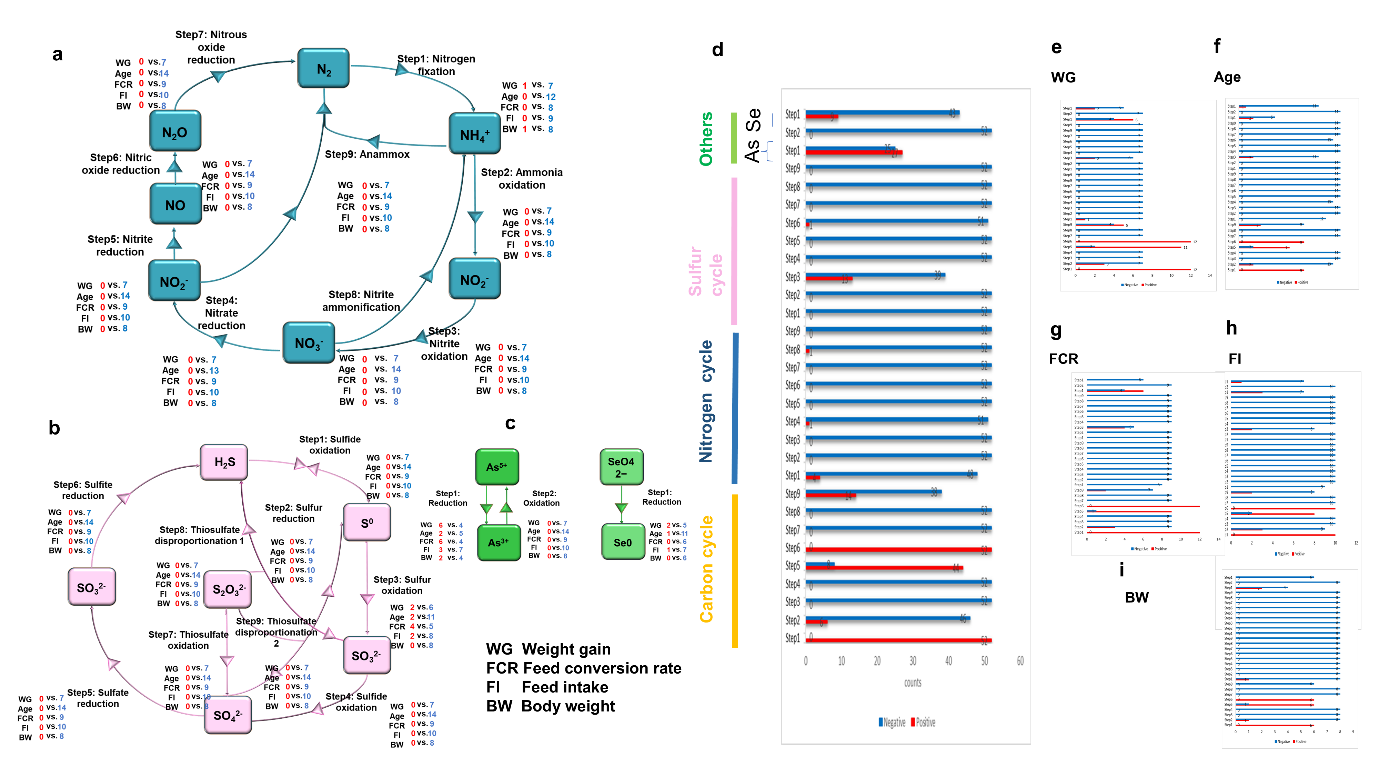


**Fig. S1** Geochemical cycles (a-i) recovered from METABOLIC with the count showing the number of genomes that have a particular pathway, along with the coverage information for all genomes recovered in this dataset. Associations to parameters (weight gain, days, feed conversion rate, feed intake and body weight) changes are indicated showing the number of genomes with positive association to parameters changes (red), and the number of genomes with negative associations to parameters changes (blue). The arrows (a-c) represent those pathways that were substantially more abundant in the community after the changes. These associations are based on CODA LASSO analysis.
