# Supplementary material for: Investigating microbial population structure and function in the chicken caeca and large intestine over time using metagenomics
Source: BMC Res Notes. 2025 Aug 15;18:355. doi: 10.1186/s13104-025-07441-7 (PMC12357378; doi:10.1186/s13104-025-07441-7)
Supplement: Supplementary file 4 — Fig. S2: CODA-LASSO regression of the parameters weight gain, feed intake, feed conversion ratio and body weight regressed against KEGG modules abundances [17] were indicated in (a-d) respectively. Non-zero β-coefficients returned from CODA-LASSO procedure are shown as two disjoint sets (those that are increasing with parameters (positive; green) and those that are decreasing with parameters (negative; red). The insets show prediction quality of fitting with the predictions from CODA-LASSO procedure shown on the x-axis and the actual values of parameters shown on the y-axis. [file 13104_2025_7441_MOESM4_ESM.docx]

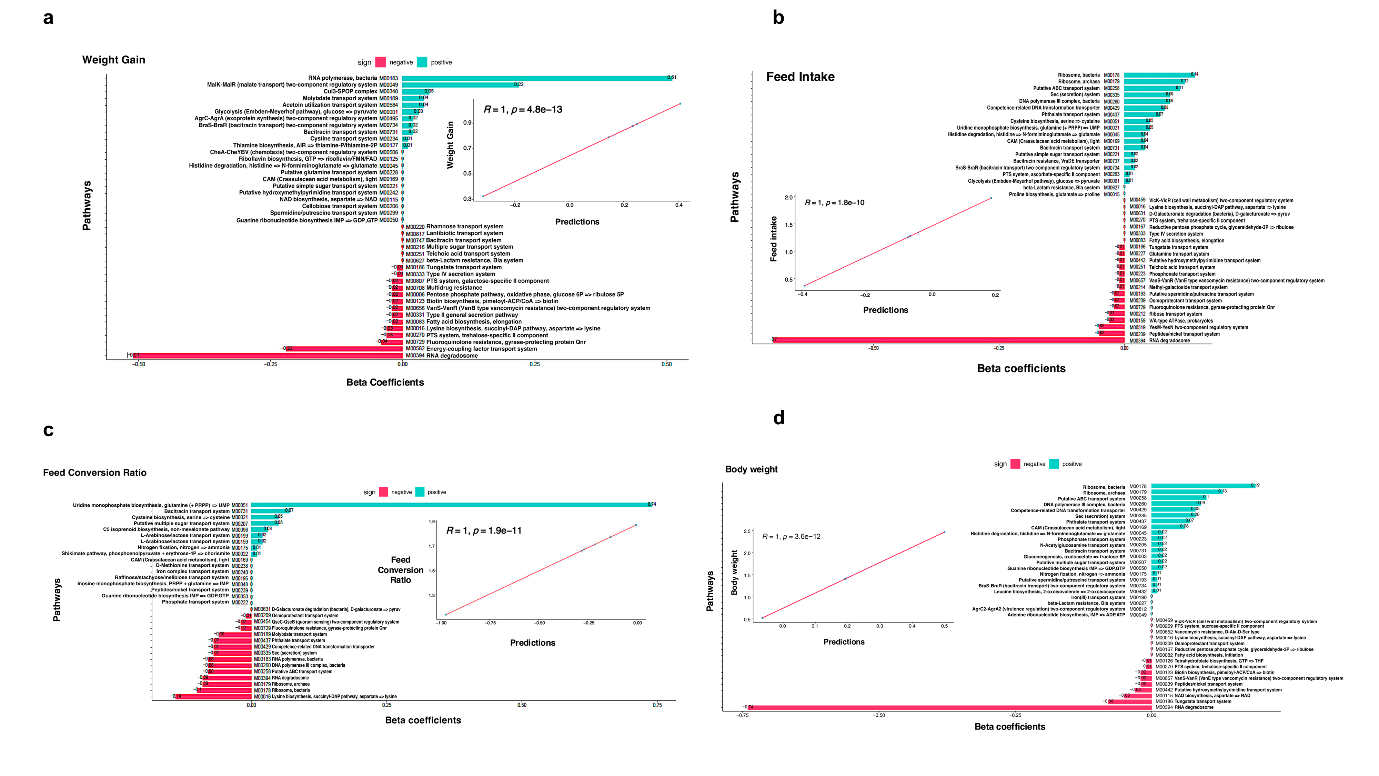


**Fig. S2** CODA-LASSO regression of the parameters weight gain, feed intake, feed conversion ratio and body weight regressed against KEGG modules abundances^38^ were indicated in (a-d) respectively. Non-zero $\beta-$coefficients returned from CODA-LASSO procedure are shown as two disjoint sets (those that are increasing with parameters (positive; green) and those that are decreasing with parameters (negative; red). The insets show prediction quality of fitting with the predictions from CODA-LASSO procedure shown on the x-axis and the actual values of parameters shown on the y-axis.
