# Supplementary material for: Investigating microbial population structure and function in the chicken caeca and large intestine over time using metagenomics
Source: BMC Res Notes. 2025 Aug 15;18:355. doi: 10.1186/s13104-025-07441-7 (PMC12357378; doi:10.1186/s13104-025-07441-7)
Supplement: Supplementary file 5 — Statistical methods used in the study. [file 13104_2025_7441_MOESM5_ESM.pdf]

Statistical analyses

All tests were performed in R v4.1.2[1]. For microbial diversity assessment, the vegan package v2.6-8[2] were employed. For alpha diversity, we estimated rarefied richness (R) and Shannon entropy (H). After confirming their normal distribution, ANOVA was then employed to determine significance differences between treatment groups[1]. For beta diversity, we calculated Bray Curtis dissimilarity index analysis using the vegdist() function of the vegan package, followed by principal component analysis using the Vegan's cmdscale() fucntion[1],[2]. To test whether microbiome profiles are different for the study cohorts considered, permutational analysis of variance (PERMANOVA) through the adonis2() function implemented in R's Vegan package was utilised[2]. To find a minimal subset of features (whether microbes, or functional features) that changed significantly between quantitative outcome (Body weight (BW), Weight gain, Age, Feed intake (FI), and FCR (Feed Conversion Ratio), we have used the CODA LASSO regression[3]. An inherent property of this regression is that it offers feature selection by using a LASSO shrinkage term in the optimization function forcing some of the *β-coefficients* to go to zero (that serve as a weight for individual features), resulting in variable selection. Furthermore, the algorithm returns two sets of non-zero *β-coefficients*, those that are positively associated with the outcome of interest, and those that are negatively associated. We have used coda_glmnet() function from R's coda4microbiome package v0.2.4[4].

Individual CAZyme function categorisation was adapted from dbCAN2 annotated substrate information(5), also utilising the CAZy database and CAZypedia (The CAZypedia Consortium, 2018). Identified enzymes were then matched to KEGG submodules using the KEGG database[6].

R's Complex Heat Map package v2.8.0 was utilised for drawing heatmaps along with average linkage clustering(7). For visualisation of the phylogenetic tree including genomic tracks, R's ggtree package v3.2.1[8] was used. The additional tracks included Guanosine-Cytosine (GC) content, novelty score (PG), and quality score (computed using formula: Completeness % – 5 x Contamination %)[8, 9] .

References

1. Team. RC. R: A language and environment for statistical computing [Computer software]. *R Foundation for Statistical Computing,*. 2022.

2. Dixon P. VEGAN, A Package of R Functions for Community Ecology. *Journal of Vegetation Science,* . 2003;14(6), 927–930.

3. Susin A, Wang Y, KA LC, Calle ML. Variable selection in microbiome compositional data analysis. NAR Genom Bioinform. 2020;2(2):lqaa029.

4. Calle ML, Pujolassos M, Susin A. coda4microbiome: compositional data analysis for microbiome cross-sectional and longitudinal studies. BMC Bioinformatics. 2023;24(1):82.

5. Zhang H, Yohe, T., Huang, L., Entwistle, S., Wu, P., Yang, Z., Busk, P. K., Xu, Y., & Yin, Y. . dbCAN2: A meta server for automated carbohydrate-active enzyme annotation. . *Nucleic Acids Research,*. 2018;46(W1), W95–W101. .

6. Kanehisa M, Goto S. KEGG: kyoto encyclopedia of genes and genomes. *Nucleic Acids Res,*. 2000;28(1):27-30.

7. Gu Z, Eils R, Schlesner M. Complex heatmaps reveal patterns and correlations in multidimensional genomic data. *Bioinformatics,*. 2016;32(18):2847-9.

8. Yu G, Smith, D. K., Zhu, H., Guan, Y., & Lam, T. T.-Y. . ggtree: An r package for visualization and annotation of phylogenetic trees with their covariates and other associated data. *Methods in Ecology and Evolution,* . 2017(8(1), 28–36.).

9. Paradis E, Claude J, Strimmer K. APE: Analyses of Phylogenetics and Evolution in R language. *Bioinformatics,*. 2004;20(2):289-90.
